# Targeting long non-coding RNA *RP11-502I4.3* inhibits the trend of angiogenesis in diabetic retinopathy

**DOI:** 10.1371/journal.pone.0312791

**Published:** 2025-05-14

**Authors:** Lan Zeng, Yuhao Wu, Lijuan Zhu, Junhao He, Yuan Yuan, Xiaocong Wang, Kai Tang, Wei Tan

**Affiliations:** 1 Department of Ophthalmology, The First People’s Hospital of Zunyi (also known as The Third Affiliated Hospital of Zunyi Medical University), Zunyi, China; 2 Zunyi Medical University, Zunyi, China; 3 Scientific Research Center, The First People’s Hospital of Zunyi (also known as The Third Affiliated Hospital of Zunyi Medical University), Zunyi, China; 4 Department of Ophthalmology, Heyou Hospital, Foshan, China.; Eye Foundation Hospital / Eye Foundation Retina Institute, NIGERIA

## Abstract

Diabetic retinopathy (DR) is a leading cause of blindness. We hypothesised that the long non-coding RNA *RP11-502I4.3* may be involved in angiogenesis associated with DR. We investigated the role of *RP11-502I4.3* in DR by examining its regulation of vascular endothelial growth factor (VEGF). We assessed differences in *RP11-502I4.3* expression between the control group and streptozotocin-induced diabetic rats or high glucose (HG)-stimulated human retinal microvascular endothelial cells (HRMECs). VEGF expression was measured with and without lentiviral vectors overexpressing *RP11-502I4.3*. We analysed the structural alterations related to DR after overexpressing *RP11-502I4.3*. Our analysis revealed that *RP11-502I4.3* expression was lower in the retinas of diabetic rats and HG-stimulated HRMECs compared with normal glucose conditions. Overexpressing of *RP11-502I4.3* resulted in decreased VEGF levels. Diabetic rats exhibited retinopathy characterised by thinning of the retinal layer thickness, structural changes in the inner and outer nuclear layers, a reduced count of retinal ganglion cells, and the presence of acellular capillaries. The proliferative activity, migration count, and tube formation ability of HG-treated HRMECs were significantly higher than those of the control group. However, these changes were inhibited by *RP11-502I4.3* overexpression. Overexpression *RP11-502I4.3* might inhibit retinopathy of diabetic rats and HG-induced angiogenesis by downregulating VEGF expression.

## Introduction

Diabetes mellitus (DM) is a significant global public health concern. The worldwide prevalence is projected to increase from 536.6 million in 2021 to 783.2 million by 2045 [1]. Diabetic retinopathy (DR), a neurovascular disorder caused by chronic hyperglycaemia, is a leading cause of blindness worldwide [2]. Proliferative diabetic retinopathy (PDR), the most advanced stage of DR, is characterised by angiogenesis that begins in the retina and can extend into the vitreous, driven by the abnormal production and release of vascular endothelial growth factor (VEGF) [3]. Retinal neovascularisation is prone to bleeding and fibrosis, which may result in vitreous haemorrhage and retinal detachment, ultimately leading to vision loss [4]. Treatment outcomes for PDR are often suboptimal [5]. Therefore, detailed studies on the molecular mechanisms underlying the pathogenesis of PDR and the development of new therapeutic agents are essential.

Long non-coding RNAs (lncRNAs), which are non-coding RNA molecules longer than 200 nucleotides, regulate gene expression and protein production at the epigenetic, transcriptional, and translational levels [6]. LncRNAs have been identified in cells, exosomes, extracellular vesicles, and blood. An increasing number of lncRNAs have been associated with vascular oculopathies and proliferative retinopathy [7,8]. With the advent of new RNA therapeutic technologies, treatments targeting lncRNAs may offer a promising new approach for PDR.

In recent years, high-throughput sequencing has significantly accelerated the discovery of novel lncRNAs and their biological functions. Yan *et al*. aimed to identify lncRNAs involved in early DR and were the first to report that approximately 303 lncRNAs were differentially expressed in the retina, with 89 upregulated and 214 downregulated [9]. Among these, *MALAT1* expression was notably upregulated in a cell model of hyperglycaemia and the aqueous humour and fibrovascular membranes of patients with PDR [9]. Other lncRNAs associated with DR, such as *ANRIL* [10], *MIAT* [11], *MEG3* [12], *H19* [13], *PVT1* [14], *BANCR* [15], and *NEAT1* [16] have also been reported. However, the role of lncRNA in PDR has received little attention.

We analysed a microarray dataset (GSE191210) and found that lncRNA *RP11-502I4.3* expression was significantly lower in the vitreous humour (VH) of patients with PDR compared with those with idiopathic macular hole (IMH) [17]. Based on these findings, we speculated that *RP11-502I4.3* may be involved in PDR by its regulation of VEGF. In this study, we investigated the expression levels and functions of *RP11-502I4.3* in the retinal tissues of streptozotocin (STZ)-induced diabetic rats and human retinal microvascular endothelial cells (HRMECs) exposed to high glucose (HG). VEGF expression was measured with and without lentiviral vectors overexpressing *RP11-502I4.3*.

## Materials and methods

### Animals

All rats were handled following the Association for Research in Vision and Ophthalmology Statement for the Use of Animals in Ophthalmic and Vision Research. The animal study was approved by the Ethics Review Committee of First People’s Hospital of Zunyi (Zunyi, China; project number 2021-2-31).

Adult male Sprague-Dawley (SD) rats, weighing 250–300 g, were housed under specific pathogen-free conditions at the Animal Experimental Centre of Zunyi Medical University. The rats were divided into four experimental groups: control group (right and left eyes of eight rats), DM group (right and left eyes of eight rats), DM + overexpression (OE)-normal control (NC) group (left eyes of 16 rats), and DM + OE-*P11-502I4.3* group (right eyes of 16 rats).

The rats were fed with a high-fat and high-sugar (HFHS) diet for four weeks to induce DM. After fasting for 16 hours, the rats received an intraperitoneal injection of STZ (Solarbio, China; 40 mg/kg in citrate buffer, pH 4.3), whereas the DM + OE-NC group received equivalent volumes of citrate buffer. After one week of STZ induction, diabetes was confirmed by blood glucose levels exceeding 16.7 mmol/L. All animals were monitored for changes in body weight and blood glucose levels per week. In addition, *RP11-502I4.3* overexpression models were established. Twelve weeks after diabetes induction, tissues were collected from all rats.

### Intravitreal injection

The lentiviral vector (LV) was designed and synthesised by Hejin Company (Guizhou, China). Diabetic rats were anaesthetised with an intramuscular injection of ketamine at a dose of 30 mg/kg. Tropicamide eye drops were then applied to dilate the pupils. Using a 33G microsyringe (Thermo, USA), we injected 1 µL of *RP11-502I4.3* overexpression virus into the right vitreous cavity once the needle tip was visible through the pupil. Similarly, the control virus was injected into the left vitreous cavity of these rats. Finally, successful *RP11-502I4.3* overexpression was confirmed using quantitative real-time polymerase chain reaction (qRT-PCR).

### qRT‑PCR

TRIzol reagent (Thermo Scientific, USA) was used to extract RNA from the retinas of rats and HRMECs. The RNA concentration was measured using a NanoDrop LITE spectrophotometre (Thermo Scientific, USA). Total RNA was reverse transcribed using the PrimeScript™ RT reagent Kit Reverse Transcription System (TaKaRa, Japan). Transcript levels were determined using the 2 × SYBR Green qPCR Mix (Solarbio, China) and a CFX96 real-time quantitative PCR detection system (Bio-Rad, USA). The specificity of the qRT-PCR products was assessed using a dissociation curve. Relative gene expression was calculated using the 2^-ΔΔCt^ method, with β-actin serving as the internal control. The primer pairs used are listed in Table 1.

**Table 1:** Primer sequence for quantitative real-time polymerase chain reaction.

Primer name	Primer Sequences
***RP11-502I4.3* (rat)**	F: 5’- GCATTGTTGATGAGGATTCGCC- 3’
	R: 5’- CACACCCACATGGACACCA- 3’
***VEGF* (rat)**	F: 5’- GTTGTGGAAGGTCAGTTCAGGATGG - 3’
	R: 5’- AGTGGTAGGGCAGACAGAAAGGG - 3’
***β*-actin (rat)**	F: 5’- ACAGCAACAGGGTGGTGGAC - 3’
	R: 5’- TTTGAGGGTGCAGCGAACTT - 3’
***RP11-502I4.3* (human)**	F: 5’- GAGACTGGTCTTGCTCGCTC - 3’
	R: 5’- ACAAAGTGGGGATAACCGGG - 3’
***VEGF* (human)**	F: 5’- CACCATGCAGATTATGCGGA - 3’
	R: 5’- ACCAACGTACACGCTCCA - 3’
***β*-actin (human)**	F: 5’- CCTCTATGCCAACACAGTGC- 3’
	R: 5’- CATCGTACTCCTGCTTGCTG - 3’

*VEGF*, *vascular endothelial growth factor*; F, Forward; R, Reverse

### Haematoxylin and eosin (H&E) staining

The eyeballs of the autopsied rats were placed into clean 1.5 mL Eppendorf tubes and fixed in 4% paraformaldehyde (Solarbio, China) at 37°C for more than 24 hours. The corneas, lenses, vitreous, and extraocular muscles were then removed. After washing in phosphate-buffered saline (PBS; Solarbio, China) and dehydrating in an alcohol gradient, the eyeballs were cleared in xylene I, II, and III, followed by immersion in paraffin, embedding, sectioning (5 μm), and baking. The paraffin sections were deparaffinised in xylene I and II, rinsed in anhydrous alcohol, and placed in an alcohol gradient. The subsequent steps were performed following a routine H&E staining procedure (Solarbio, China). Retinal thickness and the number of retinal ganglion cells (RGCs) were measured at 0.8 mm from the optic nerve.

### Periodic acid-Schiff (PAS) staining

At autopsy, each eyeball was fixed as previously described. The retinas were isolated under a dissecting microscope (Olympus, Japan) and thoroughly washed. The retinas were then incubated in a 3% trypsin solution (Solarbio, China). Isolated retinal vessels were stained with PAS (Solarbio, China) and visualised.

### Fluorescence in situ hybridisation (FISH)

Tissue fixation, dehydration, sectioning, and deparaffinisation were performed as previously described. Antigen retrieval was conducted in sodium citrate antigen retrieval solution (Solarbio, China) at 100°C for 15 minutes. After cooling, the sections were rinsed in PBS three times and incubated with the pre-hybridisation solution (RiboBio, China) in a moisture box at 37°C for one hour. Subsequently, the pre-hybridisation solution was removed, and the sections were incubated with the hybridisation solution (RiboBio, China) at 37°C overnight. The hybridisation solution was then rinsed off. Following counterstaining with DAPI (Beyotime, China), images were obtained using a fluorescence microscope (Olympus, Japan).

### Cells

In this study, HRMECs were purchased from Guangzhou Ginio Biological Corporation (Guangzhou, China). The sequence used to overexpress *RP11-502I4.3* was designed and synthesised by Hejin Company (Guizhou, China). The cells were divided into four groups: control group (no intervention), HG group (30 mmol/L glucose added to the medium and cultured for 48 hours), HG + OE-NC group, and HG + OE-*RP11-502I4.3* group. HRMECs were cultivated in a complete medium, which contained 500 mL of endothelial cell medium (ECM; ScienCell, USA), 25 mL of foetal bovine serum (ScienCell, USA), 5 mL of penicillin/streptomycin (ScienCell, USA), and 5 mL of endothelial cell growth supplement (ScienCell, USA) in an incubator (5% CO_2_ at 37°C). The cells were inoculated into new six-well plates and cultured in an incubator for approximately 24 hours. Transfections were performed according to the instructions of the manufacturer (Hejin, China). The transfection efficiency reached 70–75% after 72 hours, as assessed using fluorescence microscopy (Olympus, Japan). After 48 hours, HRMECs stably overexpressed *RP11-502I4.3*, and a complete medium was added to finish the culture.

### Cell counting kit-8 (CCK-8) assay

The cells were seeded into a 96-well plate at a density of 2 × 10^5^ cells/well. After 48 hours of incubation, CCK-8 reagent (Beyotime, China) was added to each well at a ratio of 10:1. The plates were then incubated for one hour. Cell viability was assessed by measuring the absorbance of the cells at 450 nm using an i3*x* multifunctional enzyme labelling detector (Molecular Devices, USA).

### Transwell assay

HRMECs were digested with TripLE Express (Gbico, USA), diluted to a density of 4 × 10^5^ cells/mL in complete ECM, and inoculated into the chambers of a 24-well Transwell plate. 500 µL of complete ECM was added to the lower chambers, and the cells were cultured for 48 hours. After this incubation, the upper chambers were fixed for 20 minutes. A 0.1% crystal violet solution (Beyotime, China) was then added to the chambers for 25 minutes for staining. The stained cells were counted using an upright microscope (Olympus, Japan).

### Tube formation assay

A 50 µL/well Matrigel matrix (Corning, USA) was added to a 96-well plate, taking care to avoid bubble formation. The plate was then placed in a cell incubator (37°C, 5% CO_2_, 95% humidity) for one hour to allow for solidification. Cells from the four groups were then inoculated at a density of 4 × 10^5^ cells/mL into the 96-well plate. After incubation, the plate was removed and observed under a microscope (Olympus, Japan).

### Statistical analysis

We used Image J 1.8.0 software for data analysis and GraphPad Prism 8 software for statistical analysis and graphing of the experimental data. The numeric variables were first tested for normality of distribution using the Shapiro–Wilk normality test. Comparisons between two groups were performed using independent-samples *t*-tests. For comparisons involving more than two groups, a one-way analysis of variance was employed. When the data were not normally distributed, the rank-sum test was used. Statistical significance was set at *p* < 0.05.

## Results

### Differences in *RP11-502I4.3* expression in a diabetic rat model

**Fig 1:**
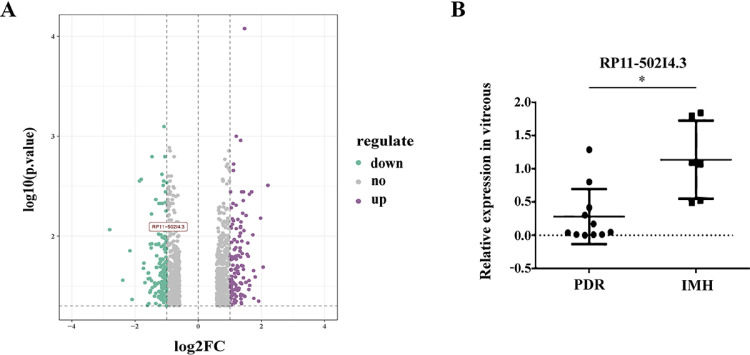
Expression levels of *RP11-502I4.3* in the human vitreous humor (VH) of patients with proliferative diabetic retinopathy (PDR). (A) Gene Ontology enrichment analysis on the expression of *RP11-502I4.3* of PDR patients compared with idiopathic macular hole (IMH) patients. (B) VH samples were collected from patients with PDR (n = 11) and IMH (n = 6) to analyse the expression of *RP11-502I4.3* using quantitative real-time polymerase chain reaction. * *p* < 0.05.

In a previous study [17], we performed microarray-based analysis to compare the lncRNA expression profiles between patients with PDR and those with IMH (Fig 1A). The expression levels of RP11-502I4.3 in the VH of patients with PDR were significantly lower, as confirmed by qRT-PCR (Fig 1B).

**Fig 2:**
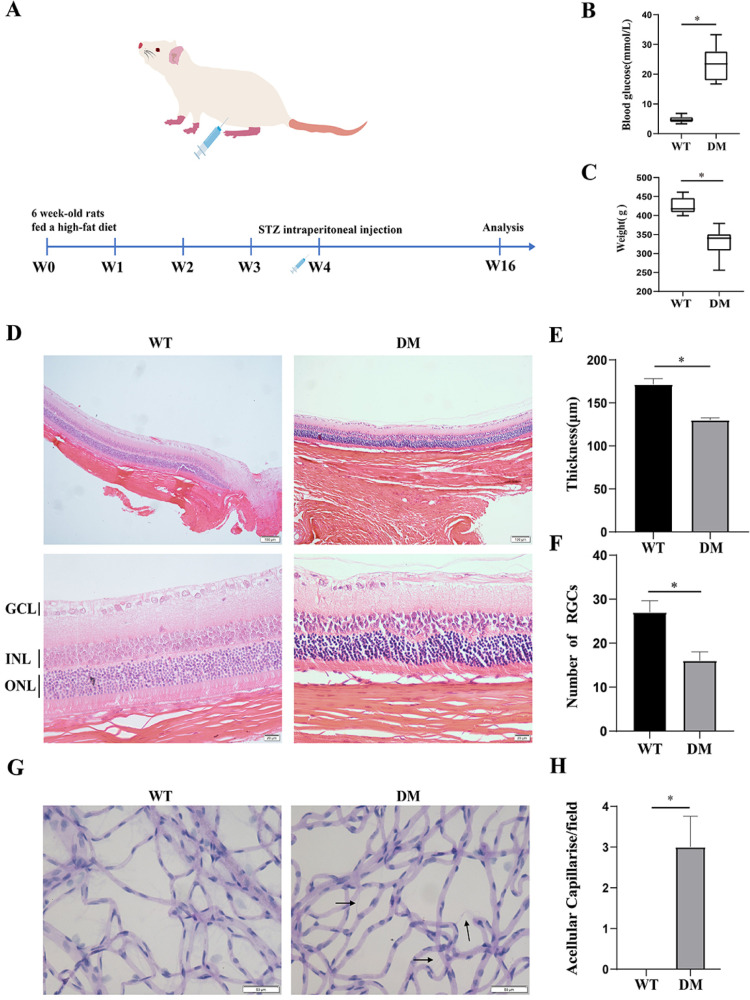
Establishment of a diabetic rat model. (A) Experimental setting for the streptozotocin (STZ)-induced diabetic rat model. Rats were treated with 40 mg/kg STZ (diabetes mellitus group, DM group) or sodium citrate buffer vehicle (control group). (B) Blood glucose and (C) body weight of rats at 12 weeks after the STZ injection (n = 8 samples for the control group and n = 24 samples for the DM group). * p < 0.05. (D) Hematoxylin and eosin staining of retinal sections of the control group and DM group. The ganglion cell layer (GCL), inner nuclear layer (INL), and outer nuclear layer (ONL) are shown in the figure. Changes of (E) retinal layer thickness and (F)the number of retinal ganglion cells (RGCs) in diabetic rats. The above experiments were repeated three times in parallel. * p < 0.05. (G) Periodic acid-Schiff staining of retinas of the control group and DM group. The acellular capillaries were shown by black arrows. (H) Change of acellular capillary in diabetic rats. The experiment was repeated three times in parallel. * p < 0.05.VEGF is a key factor in the pathogenesis of DR [19]. The abnormal production and release of VEGF promote the proliferation and migration of endothelial cells (ECs), which are critical drivers of retinal neovascularization [19]. Accordingly, we measured VEGF gene expression and found that it was significantly increased in the retinas of diabetic rats (Fig 3A). In addition, RP11-502I4.3 expression was decreased in the retinas of diabetic rats compared with non-diabetic rats (Fig 3B), and it was predominantly localised in the cytoplasm (Fig 3C).

However, the involvement of RP11-502I4.3 in PDR remains unclear. To investigate the potential role of RP11-502I4.3 in the pathogenesis of DR, we established a diabetic rat model to verify its expression. After being fed a HFHS diet for four weeks, adult SD rats were injected intraperitoneally with STZ (Fig 2A). We measured rats’ blood glucose levels and body weights after STZ injection. The blood glucose levels in the DM group were significantly higher than those in the control group, and body weight was notably reduced (Fig 2B and 2C). Indicators of retinopathy in diabetic rats included reduced retinal layer thickness (RLT) and retinal cell count [18]. In this study, we observed thinning of the RLT, structural changes in the inner nuclear layer (INL) and outer nuclear layer (ONL), and a decreased count of RGCs in diabetic rats compared with non-diabetic rats (Fig 2D–2F). Acellular capillaries (ACs) were also present in the retinas of diabetic rats (Fig 2G and 2H).

**Fig 3:**
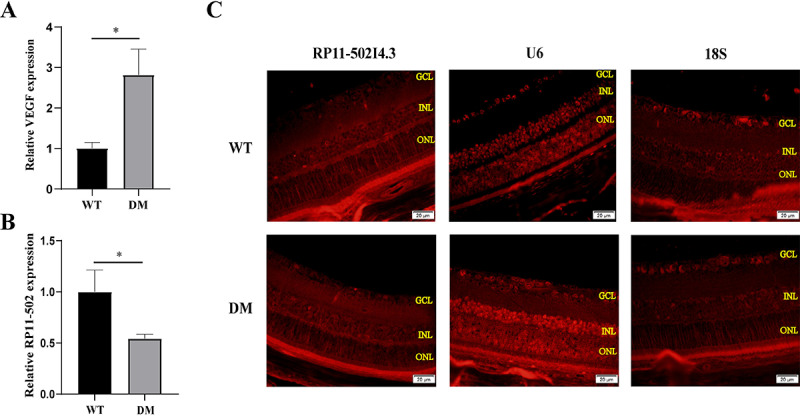
Detection of changes in *vascular endothelial growth factor* (*VEGF*) and *RP11-502I4.3* expression levels. The expression of (A) *VEGF* and (B) *RP11-502I4.3* in control and diabetes mellitus (DM) groups were analyzed by quantitative real-time polymerase chain reaction. The above experiments were repeated times in parallel. * *p* < 0.05. (C) The fluorescence in situ hybridization was performed to detect the fluorescence intensity of *RP11-502I4.3*. *RP11-502I4.3* in DM group showed high expression in U6. U6 represents cytoplasm, 18S represents nucleus. The ganglion cell layer (GCL), inner nuclear layer (INL), and outer nuclear layer (ONL) are shown in the figure. The experiment was repeated three times in parallel.

### Establishment of *RP11-502I4.3* overexpression models

**Fig 4:**
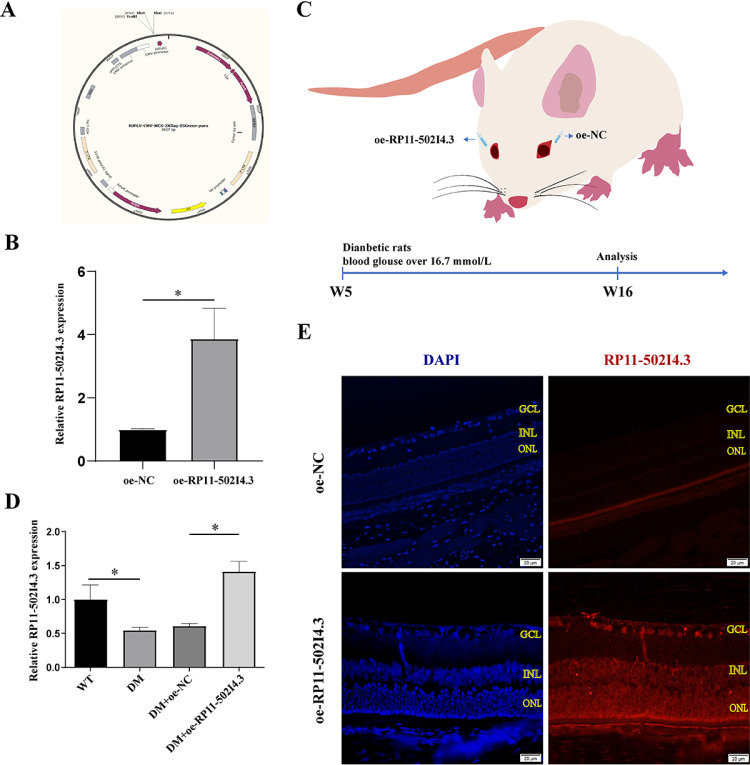
Establishment of overexpressing *RP11-502I4.3* model in rats. (A) Schematic of lentiviral vector (LV) to overexpress *RP11-502I4.3* model in rats. (B) Verification of transfection efficiency of *RP11-502I4.3* using quantitative real-time polymerase chain reaction (qRT-PCR). The experiment was repeated three times in parallel. * *p* < 0.05. (C) Schematic of transient and local overexpression (OE) of retinal *RP11-502I4.3* by single intravitreal injection of OE-*RP11-502I4.3* (LV of overexpressing *RP11-502I4.3*) or OE-normal control (NC) (LV of overexpressing nonsense sequence) into diabetic rats. (D) Expression of *RP11-502I4.3* using qRT-PCR among four groups. The four groups are the control group, diabetes mellitus (DM) group, DM + OE-NC group, and DM + OE-*RP11-502I4.3* group. The experiment was repeated three times in parallel. * *p* < 0.05. (E) The fluorescence in situ hybridization was performed to compare the fluorescence intensity of *RP11-502I4.3* between DM + OE-NC and DM + OE-*RP11-502I4.3* in retinas of rats. The experiment was repeated three times in parallel.

*In vivo* experiments, an overexpression model of *RP11-502I4.3* was established using a LV to investigate the relevance of *RP11-502I4.3* downregulation in neovascularisation (Fig 4A and 4B). Diabetic rats were selected for intravitreal injection one week after STZ induction (Fig 4C). Verification was conducted using qRT-PCR and FISH (Fig 4D and 4E). The expression levels of *RP11-502I4.3* in the DM + OE-*RP11-502I4.3* group were significantly higher than those in the DM + OE-NC group (Fig 4D).

**Fig 5:**
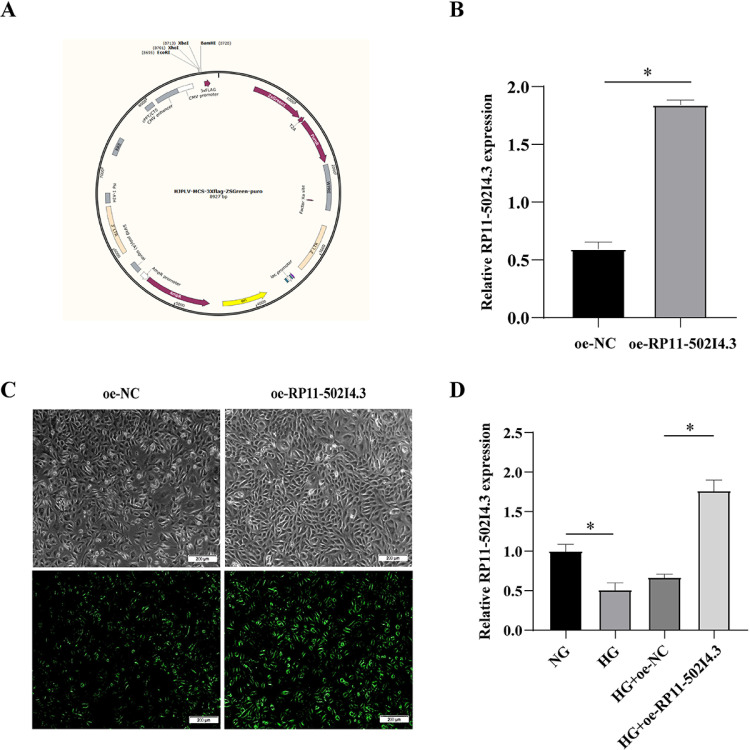
Establishment of overexpressing *RP11-502I4.3* model in human retinal microvascular endothelial cells (HRMECs). (A) Schematic of lentiviral vector (LV) to overexpress *RP11-502I4.3* model in HRMECs. (B) Verification of transfection efficiency of *RP11-502I4.3* using quantitative real-time polymerase chain reaction (qRT-PCR). The experiment was repeated three times in parallel. * *p* < 0.05. (C) Transfection efficiency of LV in HRMECs. The experiment was repeated three times in parallel. (D) Expression of *RP11-502I4.3* using qRT-PCR among four groups. The four groups are the control group, high glucose (HG) group, HG + overexpression (OE)-normal control (NC) (LV of overexpressing nonsense sequence) group, and HG with OE-*RP11-502I4.3* (LV of overexpressing *RP11-502I4.3*) group. The experiment was repeated three times in parallel. * *p* < 0.05.

Next, we developed a cell model for *RP11-502I4.3* overexpression (Fig 5A and 5B). The transfection efficiency of overexpression was evaluated 72 hours after lentivirus-mediated transfection. Both the HG + OE-*RP11-502I4.3* and HG + OE-NC groups were assessed for the expression of green fluorescent protein, with transfection efficiency exceeding 70% (Fig 5C). The verification process was performed similarly (Fig 5D).

### *RP11-502I4.3* overexpression inhibited the trend of retinopathy in the diabetic rat model and angiogenesis HG-treated HRMECs model

**Fig 6:**
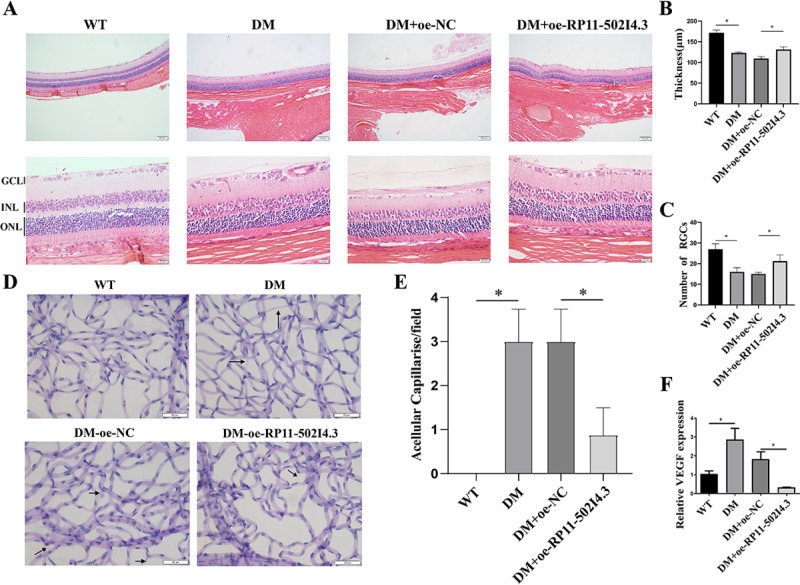
Overexpressing *RP11-502I4.3* inhibited the trend of angiogenesis in rats. (A) Hematoxylin and eosin staining of retinal sections of the control group, diabetes mellitus (DM) group, DM + overexpression (OE)-normal control (NC) group, and DM + OE-*RP11-502I4.3* group. (B) Retinal layer thickness and (C) The number of retinal ganglion cells (RGCs) of four groups. The above experiments were repeated three times in parallel. * *p* < 0.05. (D) Periodic acid-Schiff staining of retinas of four groups. The acellular capillaries are shown by black arrows. (E) Change of acellular capillary of four groups. The experiment was repeated three times in parallel. * *p* < 0.05. (F) The expression of *vascular endothelial growth factor (VEGF)* in rats after overexpressing the *RP11-502I4.3* was analyzed by quantitative real-time polymerase chain reaction. The experiment was repeated three times in parallel. * *p* < 0.05.

Using H&E staining, we observed the occurrence of retinopathy in diabetic rats, including thinning of the RLT, structural changes in the INL and ONL, and a decreased count of RGCs. In contrast, diabetic rats overexpressing *RP11-502I4.3* showed inhibited progression of these changes (Fig 6A–6C). The number of ACs in diabetic rats with *RP11-502I4.3* overexpression was significantly lower than that in diabetic rats without *RP11-502I4.3* overexpression (Fig 6D and 6E). In addition, *VEGF* gene expression was significantly downregulated in diabetic rats after *RP11-502I4.3* overexpression (Fig 6F).

**Fig 7:**
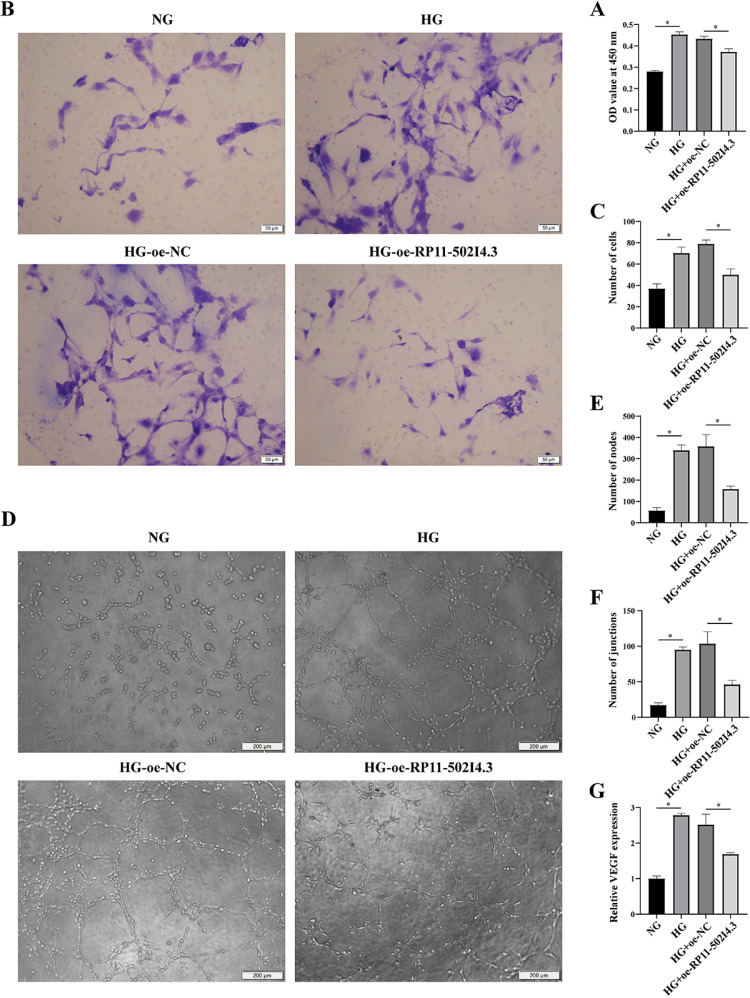
Overexpressing *RP11-502I4.3* inhibited the trend of angiogenesis in human retinal microvascular endothelial cells (HRMECs). (A) Cell Counting Kit-8 assay was performed to determine the cell viability of the control group, high glucose (HG) group, HG + overexpression (OE)-normal control (NC) group, and HG + OE-*RP11-502I4.3* group. The experiment was repeated three times in parallel. * *p* < 0.05. (B) The migrated cells were fixed and stained after the transwell migration assay. (C) The number of migrated cells in each group. The experiment was repeated three times in parallel. * *p* < 0.05. (D) The images of tube-like structures were observed under an inverted microscope after 24 hours of incubation. (E) The number of tube-forming nodes in each group. (F) The number of tube-forming branches in each group. The above experiments were repeated three times in parallel. * *p* < 0.05. (G) The expression of *vascular endothelial growth factor* (*VEGF*) in human retinal microvascular endothelial cells (HRMECs) after overexpressing the *RP11-502I4.3* was analyzed by quantitative real-time polymerase chain reaction. The experiment was repeated three times in parallel. * *p* < 0.05.

*In vitro* experiments demonstrated that the proliferation activity (Fig 7A), migration count (Fig 7B and 7C), and tube formation ability (Fig 7D–7F) of HG-induced HRMECs were increased compared with normal glucose conditions. However, these changes were inhibited following *RP11-502I4.3* overexpression. Similarly, *VEGF* expression was significantly downregulated in HG-induced HRMECs after *RP11-502I4.3* overexpression (Fig 7G).

## Discussion

DR is the leading cause of blindness in the elderly, with its pathogenesis being multifactorial. A detailed understanding of this complexity is essential. In the early stages of DR, pericyte loss and EC apoptosis are triggered by chronic hyperglycaemia, leading to retinal non-perfusion (RNP). As retinal hypoxia increases, VEGF expression rises, becoming a critical driver of PDR. This progression results in further RNP, which in turn enhances VEGF expression, creating a vicious cycle. Anti-VEGF therapy has become a vital component of DR treatment. Frequent anti-VEGF injections can disrupt this cycle, slow the progression of RNP, and alleviate retinopathy [20]. However, the frequency of injections in clinical practice is often low for various reasons, leading to disappointing visual acuity outcomes. Resistance to retinal neovascularisation remains a significant challenge. Gene therapy offers several advantages over long-term anti-VEGF injections and presents a promising, attractive, and cost-effective alternative treatment.

LncRNAs have garnered significant attention in the study of vascular eye diseases and ocular angiogenesis [7,21]. Their functional mechanisms are diverse; some act as competitive endogenous RNAs, whereas others interact with proteins to regulate various angiogenic factors, such as VEGF, and hypoxia-inducible factor 1-alpha, as well as numerous angiogenic and inflammatory signalling pathways, including mitogen-activated protein kinase, phosphoinositide 3-kinase/protein kinase B, and nuclear factor kappa-light-chain-enhancer of activated B cells pathways [7]. LncRNAs are emerging as a novel strategy for the treatment of DR [7,22]. Recent studies suggest that lncRNAs offer additional layers of gene regulation owing to their diverse mechanisms [7]. In the previous study [17], we detected the expression levels of lncRNAs in VH of PDR and IMH patients by gene microarray. There have been systemic expression differences between them. Differentially expressed lncRNAs were sorted according to the absolute value of fold change (FC). Several candidate lncRNAs with multi-variable shear or difficult primer design were excluded. Finally, five target genes were selected for subsequent qRT-PCR analysis. Compared with IMH patients, *RP11-502I4.3*, *RP11-2A4.3*, *RP11-654G14.1*, *RP11-787B4.2*, and *RP11-573J24.1* in VH of PDR patients were decreased expressions. These results are consistent with the gene chip results. *RP11-502I4.3* is the lncRNA with the largest absolute value of FC among them (FC = -2.27). Therefore, we chose it in the next basic experiment. In this study, *RP11-502I4.3* was significantly downregulated in the hyperglycaemic HRMEC model and diabetic rat model. *RP11-502I4.3* may play a role in inhibiting retinal neovascularisation in DR; however, it remains poorly annotated and has yet to be reported in the context of DR or other diseases.

Numerous angiogenic factors have been confirmed to regulate angiogenesis. One of the most extensively studied angiogenic factors is VEGF, which is a critical growth factor specific to ECs [7]. As a pathogenic mediator of retinal blood vessel leakage and abnormal growth, VEGF is a prominent therapeutic target in DR gene therapy research [23]. Various studies have attempted to interfere with the intraocular VEGF pathway both extracellularly and intracellularly. Dysregulated lncRNAs associated with models of ocular angiogenesis and can be categorised into pro-angiogenic lncRNAs (such as *ANRIL*, *H19*, *HDAC4-AS1*, *HIF1A-AS2*, *HOTAIR*, *HOTTIP*, *IPW*, *lncEGFL7OS*, *MALAT1*, *MIAT*, *NEAT1*, *SNHG16*, *TUG1*, *SNHG16*, *HIF1A-AS2*, *HDAC4-AS1*, *Vax2os1* and *Vax2os2*) and anti-angiogenic lncRNAs (such as *MEG3* and *PKNY*) [7]. In our study, *RP11-502I4.3* overexpression downregulated *VEGF* expression. We hypothesise *RP11-502I4.3* downregulated may contribute to the formation of retinal neovascularisation by regulating VEGF.

DR represents a deterioration of the neurovascular unit (NVU), which comprises neurons (ganglion cells, amacrine cells, and horizontal and bipolar cells), glial cells (astrocytes, Müller cells, and microglial cells), and vascular cells (ECs and pericytes). These components engage in intricate functional coupling and contribute to both the early and advanced stages of DR [24]. The molecular mechanisms underlying NVU dysfunction, particularly endothelial dysfunction, in DR are multifactorial [25]. Numerous studies have investigated the factors associated with endothelial dysfunction in DR, including advanced glycosylation end products and their receptors, disruption of peroxisome proliferator-activated receptor-γ, chronic inflammation, leukostasis [26–29], oxidative stress and dysregulated growth factors, cytokines, and non-coding RNA networks [29–32]. These investigations have primarily focused on the connection between ECs and retinal neovascularisation. Angiogenesis is a complex multistep process [33]. Various reviews have indicated that ECs are the primary targets of hyperglycaemic damage [34–38]. Even in the early stages of DR, pericyte loss increases microvascular penetrability and exacerbates damage to the blood-retinal endothelial barrier. Continuous damage to the retinal microvasculature leads to retinal ischaemia and capillary non-perfusion [23]. In the later stages of DR, VEGF upregulation promotes the proliferation, migration, and angiogenesis of ECs [39]. The angiogenic process involves the breakdown of EC junctions, vessel dilation, degradation of the basement membrane, and EC proliferation in response to this angiogenic growth factor [40]. Given the critical role of ECs in angiogenesis [39], we selected HRMECs as the target cells for this study. However, dysregulated lncRNAs can adversely affect various retinal cells [41,42]. In addition to ECs, other retinal cells can produce VEGF under HG conditions [25]. Almost all retinal cells can act as effectors or donors of VEGF and interact with each other through these mechanisms. The complex interactions among multiple factors highlight the intricate and complex nature of DR, indicating that treatment with a single factor is insufficient to reverse its progression [25]. Whether *RP11-502I4* in HRMECs interacts with neighbouring cells, such as pericytes, to induce endothelial dysfunction through intercellular communication and contribute to the pathogenesis of DR remains unknown. Further research on other potential target cells is warranted.

Various *in vivo* models of DR have been developed for experimental research [43]. We selected rats as the animal model because of their small size, short lifespan, and rapid breeding, making them ideal for efficient studies. However, these models do not fully replicate all the features of DR (for example, retinal neovascularization) [44]. Only a few higher-order animal models can mimic the retinopathy observed in the late stages of DR [45]. Despite these limitations, rodent models provide valuable insights into the pathogenesis of DR and have been successfully employed in preclinical studies to identify therapeutic targets and screen drug candidates [44].

Gene therapy is designed to introduce a target gene into patient cells to deliver a therapeutic transgene. Luxturna, a virus-based gene therapy for Leber’s congenital amaurosis, was approved by the US Food and Drug Administration in 2017, marking a significant milestone in ocular gene therapy [46]. Gene therapy for complex polygenic eye diseases, such as glaucoma, age-related macular degeneration, and DR, has garnered increasing interest. Anti-angiogenic proteins or non-coding RNA interference effector molecules, delivered through viral or non-viral vectors, have shown potential in effectively inhibiting the progression of DR [2]. Gene therapy has several avenues of development, including gene augmentation, gene-specific targeting, and genome editing. Currently, gene therapy for DR primarily focuses on gene-specific targeted therapies, which require the construction of viral vectors to overexpress the target gene. Although viral vectors have been used in treatment, several aspects of their use remain under investigation [47]. The development of new molecular-targeted drugs and drug delivery systems is expected to enable more effective drug delivery in the future [47]. Emerging technologies for RNA therapy, such as nanoparticle-conjugated lncRNA overexpression and small interfering RNA-based lncRNA knockdown systems, suggest that lncRNA treatments may soon become a reality.

Our study had certain limitations. Firstly, because of the limited annotation of *RP11-502I4*, we were unable to predict its associated mRNA or signalling pathways using bioinformatics. Consequently, we focused on exploring the regulatory mechanisms of *RP11-502I4* in retinal neovascularisation. Secondly, only ECs were selected as target cells *in vitro* experiments. In the future, further investigation into the role of other retinal cells in this process is needed, which may help elucidate the potential connections and interactions between ECs and neighbouring cells when *RP11-502I4* inhibits retinal neovascularisation. Finally, there are no typical features associated with PDR or vascular leakage in *vivo* experiments. In future experiments, we plan to use oxygen-induced retinopathy (OIR) as an animal model to investigate the relationship between *RP11-573J24.1* and retinal neovascularization and to use ethidium bromide (EB) staining as an observation target to realize the change in vascular permeability after upregulation of *RP11-573J24.1*.

Therefore, we found that *RP11-502I4.3* expression was lower in the retinas of diabetic rats and HG-stimulated HRMECs compared with normal glucose conditions. The dysregulated *RP11-502I4* is involved in the neovascularization of DR. This process may be mediated by VEGF.

## Supporting information

S1 File Original data1. (ZIP)

S2 File Original data2. (ZIP)

## Abbreviations

DM: diabetes mellitus
DR: diabetic retinopathy
PDR: proliferative diabetic retinopathy
VEGF: vascular endothelial growth factor
lncRNAs: long non-coding RNAs
VH: vitreous humour
IMH: idiopathic macular hole
HRMECs: human retinal microvascular endothelial cells
HG: high glucose
STZ: streptozotocin
SD: Sprague-Dawley
OE: overexpression
NC: normal control
HFHS: high-fat and high-sugar
LV: lentiviral vector
qRT-PCR: quantitative real-time polymerase chain reaction
H&E: haematoxylin and eosin
PBS: phosphate-buffered saline
RGCs: retinal ganglion cells
PAS: periodic acid-Schiff
FISH: fluorescence in situ hybridisation
ECM: endothelial cell medium
CCK-8: cell counting kit-8
RLT: retinal layer thickness
INL: inner nuclear layer
ONL: outer nuclear layer
ACs: acellular capillaries
ECs: endothelial cells
RNP: retinal non-perfusion
FC: fold change
NVU: neurovascular unit
OIR: oxygen-induced retinopathy
EB: ethidium bromide
