# Supplementary figures and images for: Targeting long non-coding RNA RP11-502I4.3 inhibits the trend of angiogenesis in diabetic retinopathy (part 1 of 2)
Source: PLoS One. 2025 May 14;20(5):e0312791. doi: 10.1371/journal.pone.0312791 (PMC12077687; doi:10.1371/journal.pone.0312791)

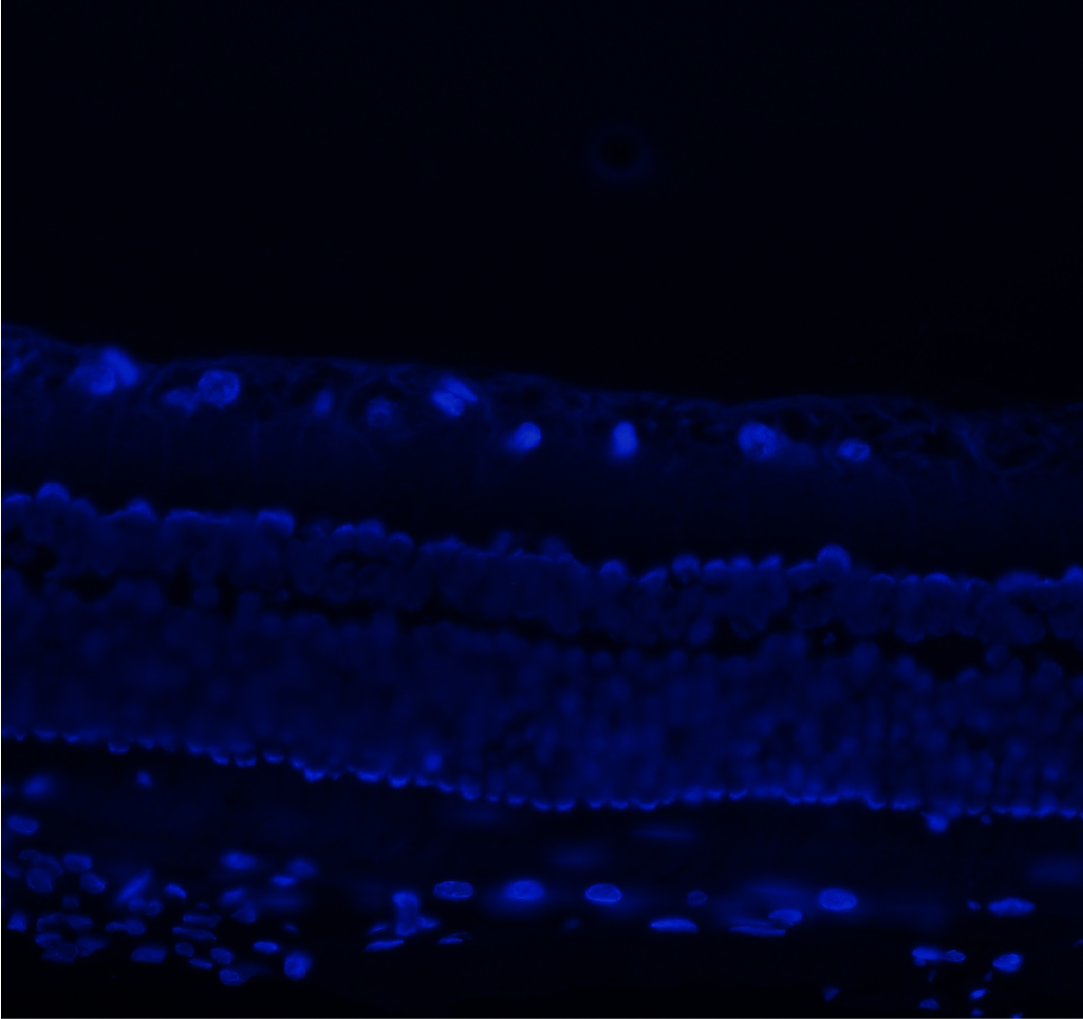

Supplement: S1 File — (ZIP) [file pone.0312791.s001.zip › Fig 4/Fig4 FISH/OE DAPI-1.tif]

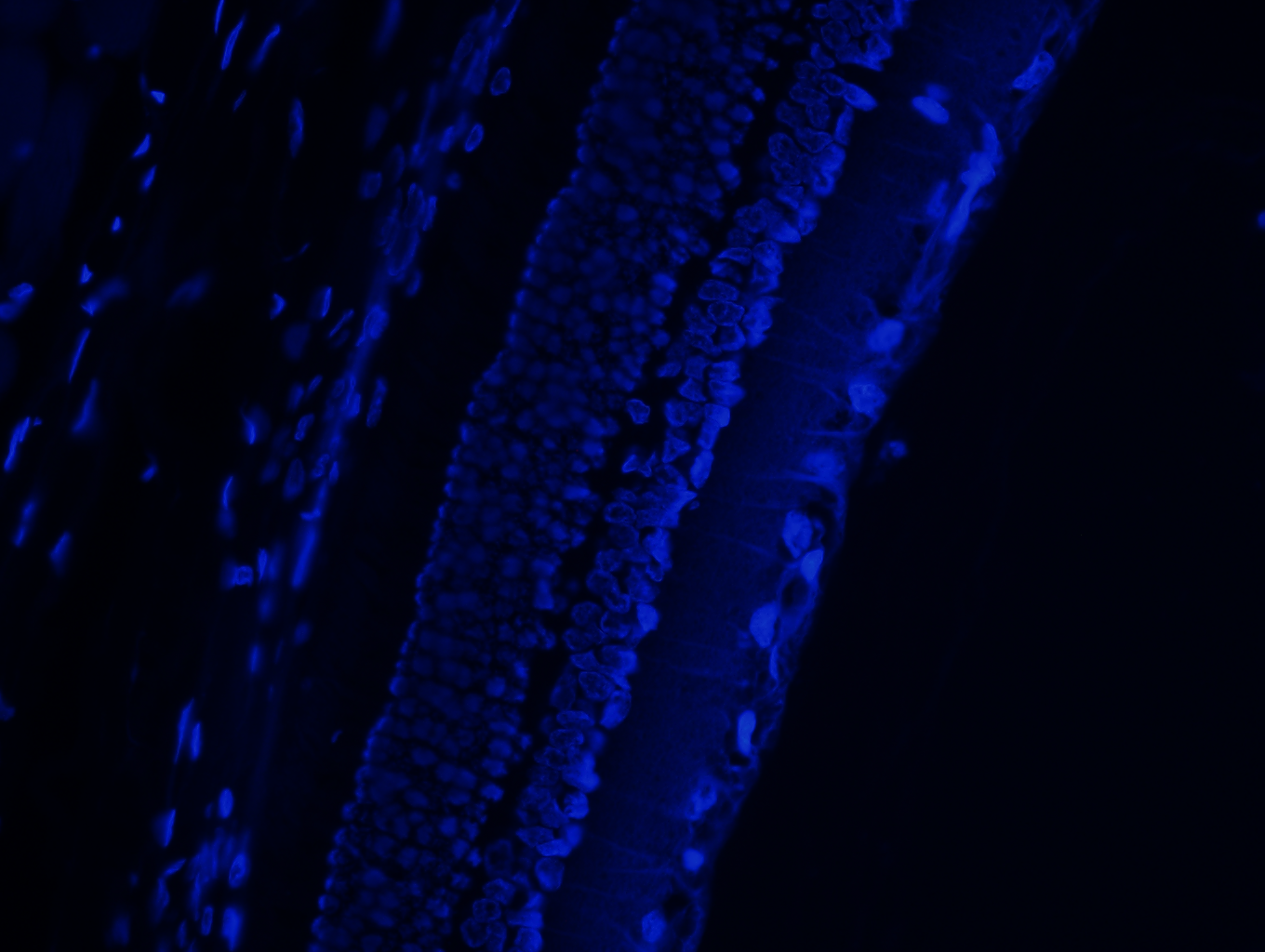

Supplement: S1 File — (ZIP) [file pone.0312791.s001.zip › Fig 4/Fig4 FISH/OE DAPI-2.tif]

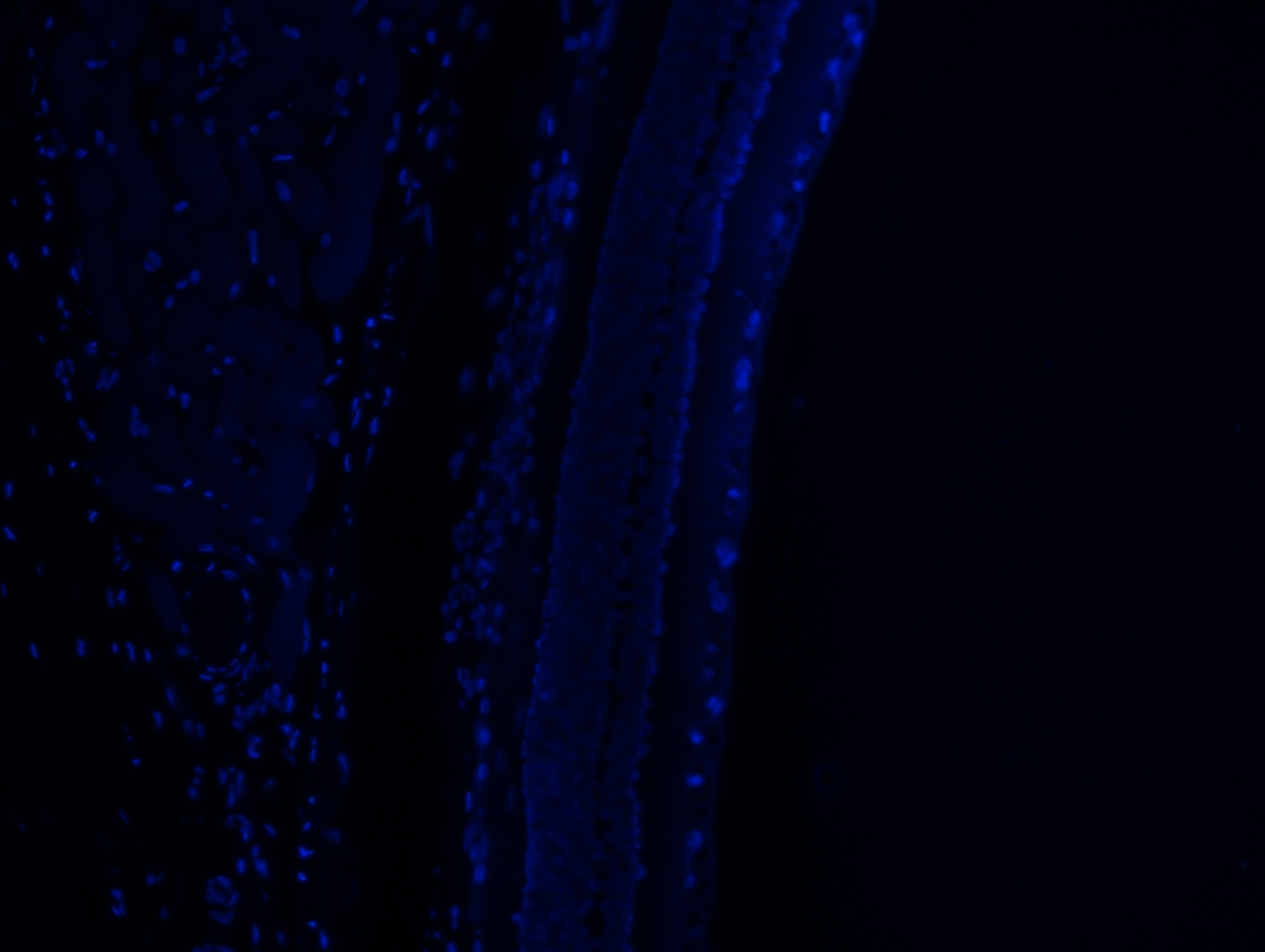

Supplement: S1 File — (ZIP) [file pone.0312791.s001.zip › Fig 4/Fig4 FISH/OE DAPI-3.tif]

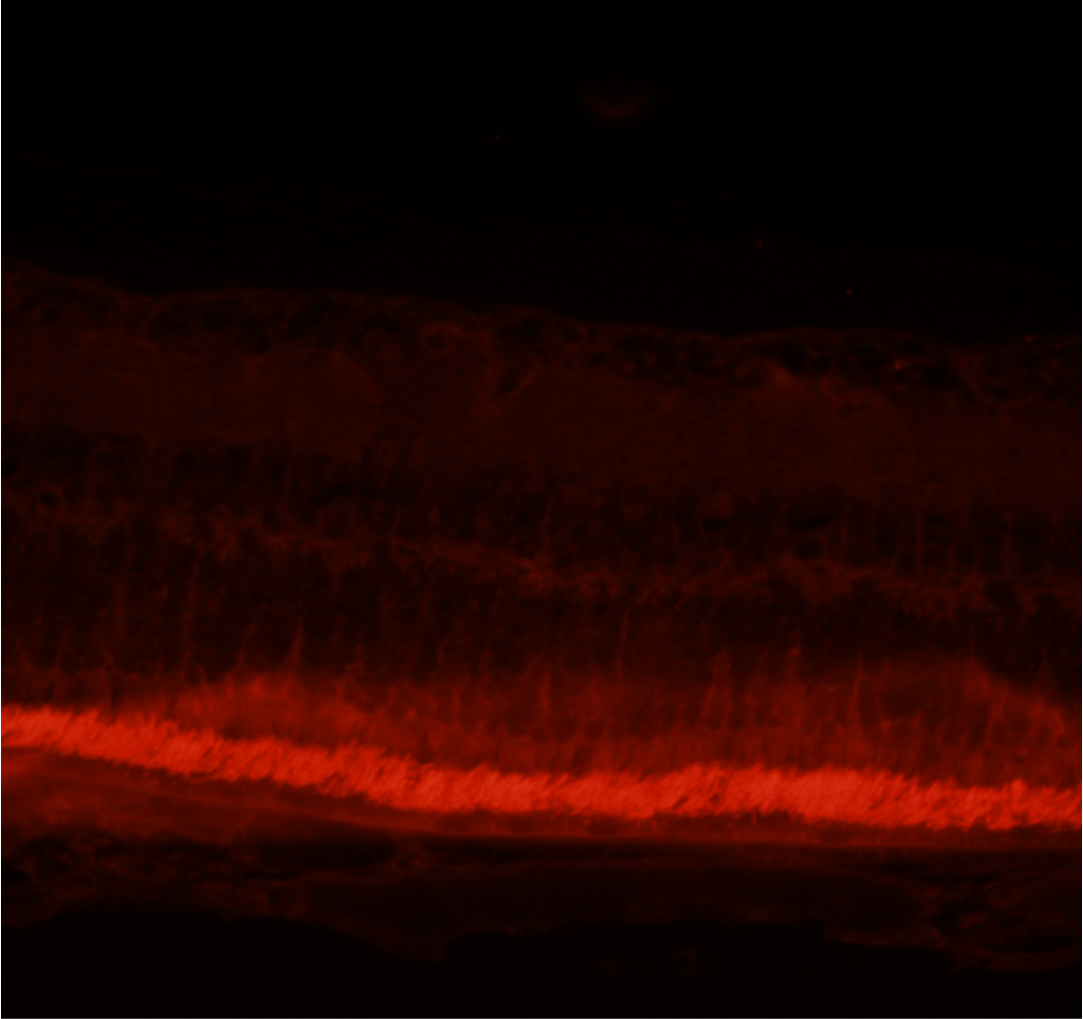

Supplement: S1 File — (ZIP) [file pone.0312791.s001.zip › Fig 4/Fig4 FISH/OE RP11-1.tif]

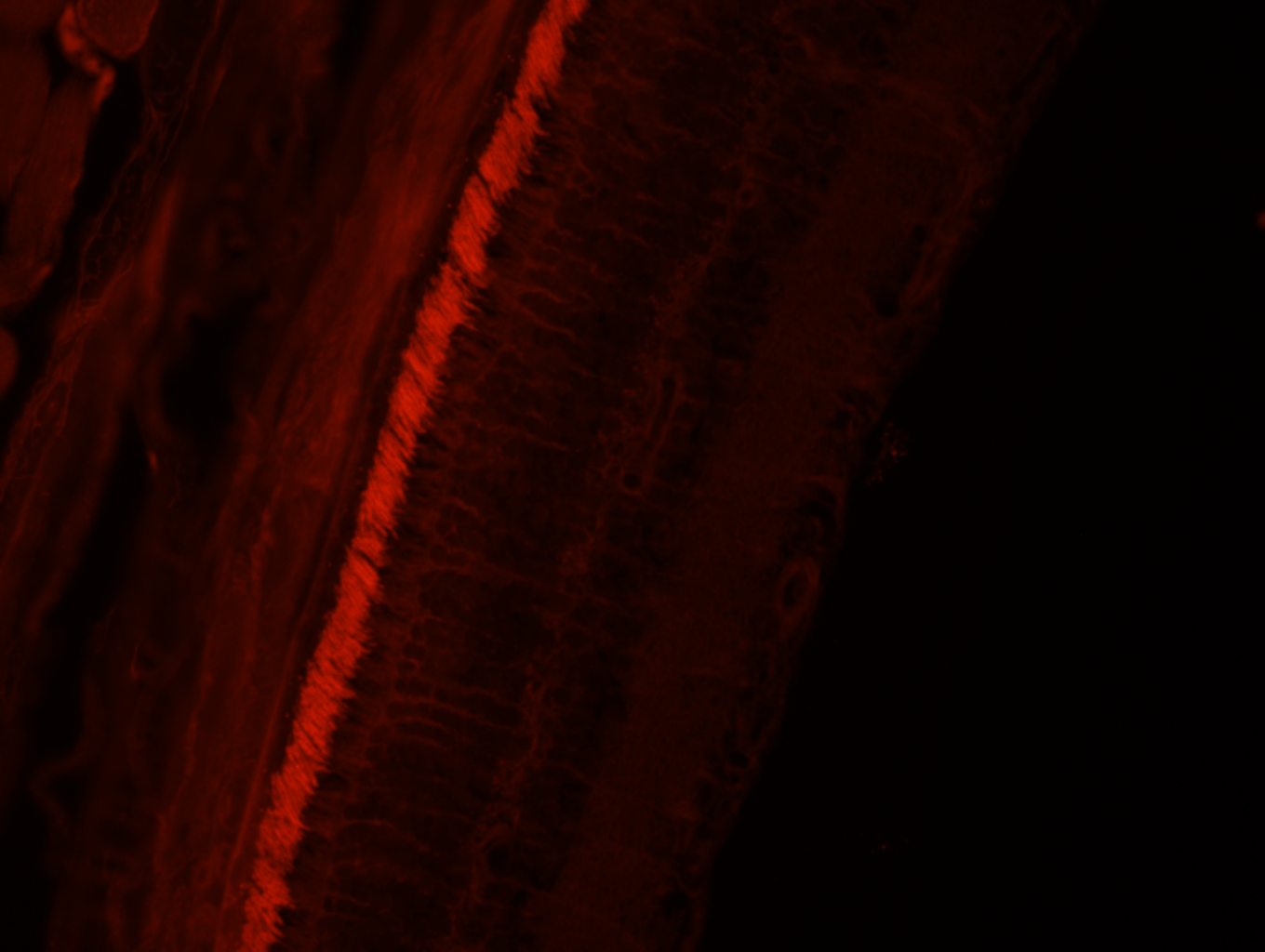

Supplement: S1 File — (ZIP) [file pone.0312791.s001.zip › Fig 4/Fig4 FISH/OE RP11-2.tif]

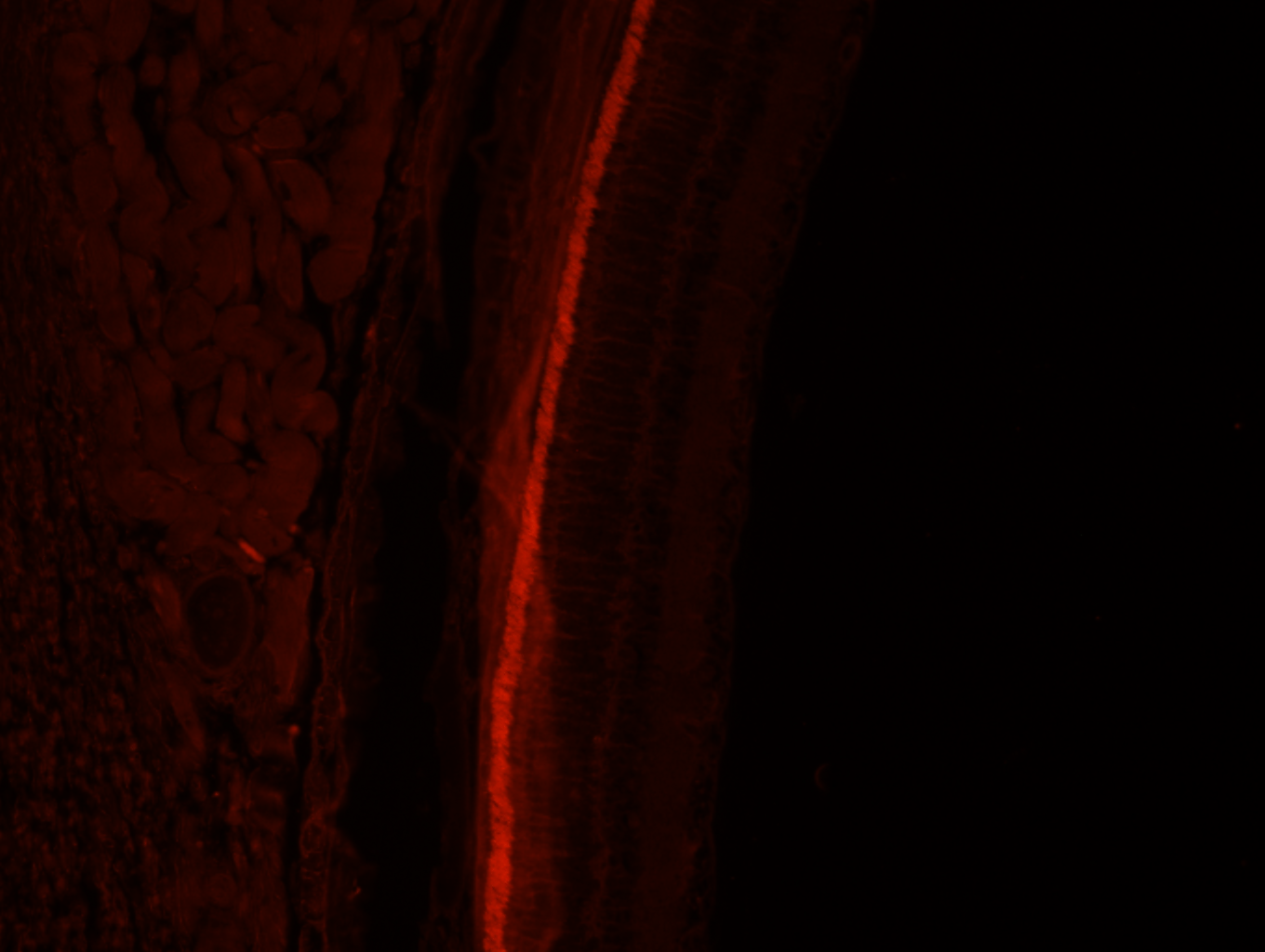

Supplement: S1 File — (ZIP) [file pone.0312791.s001.zip › Fig 4/Fig4 FISH/OE RP11-3.tif]

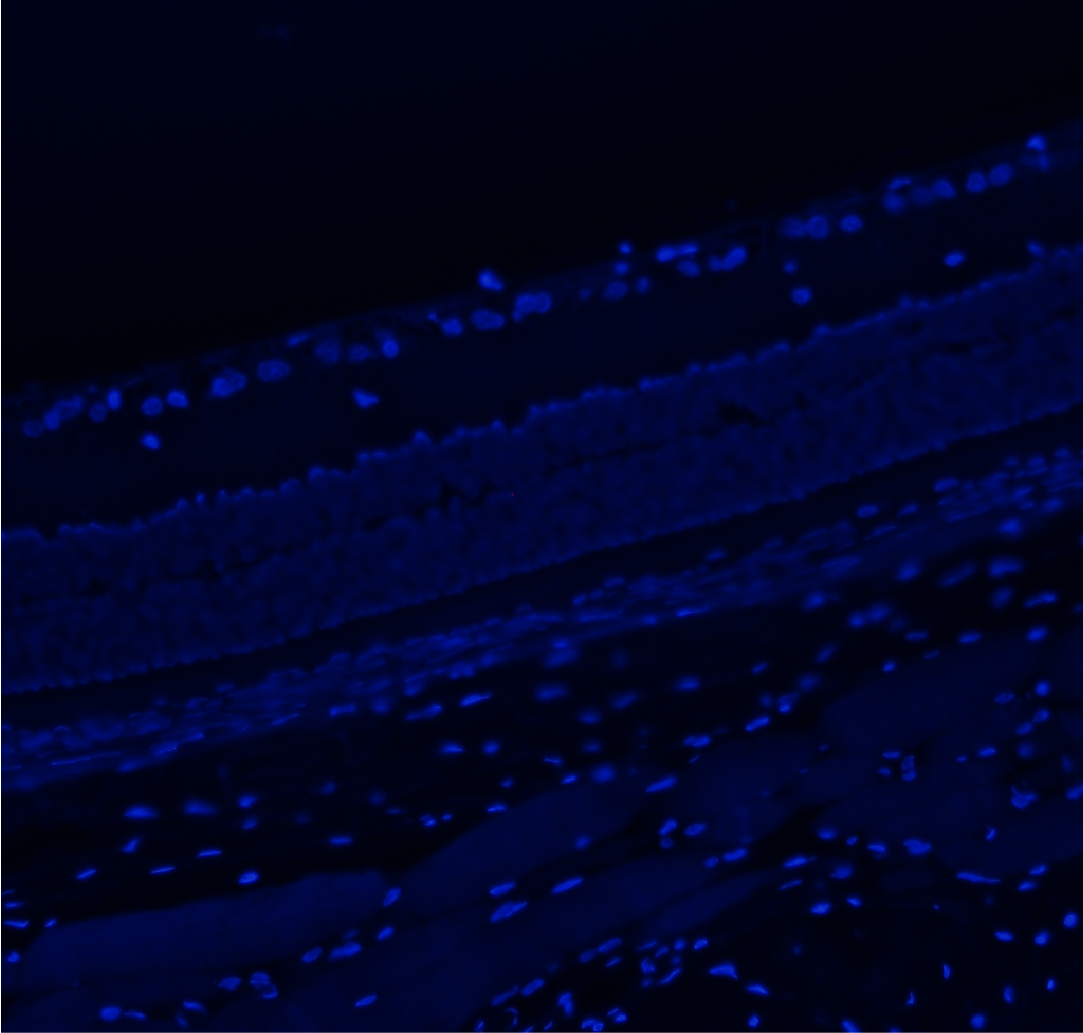

Supplement: S1 File — (ZIP) [file pone.0312791.s001.zip › Fig 4/Fig4 FISH/OENC DAPI-1.tif]

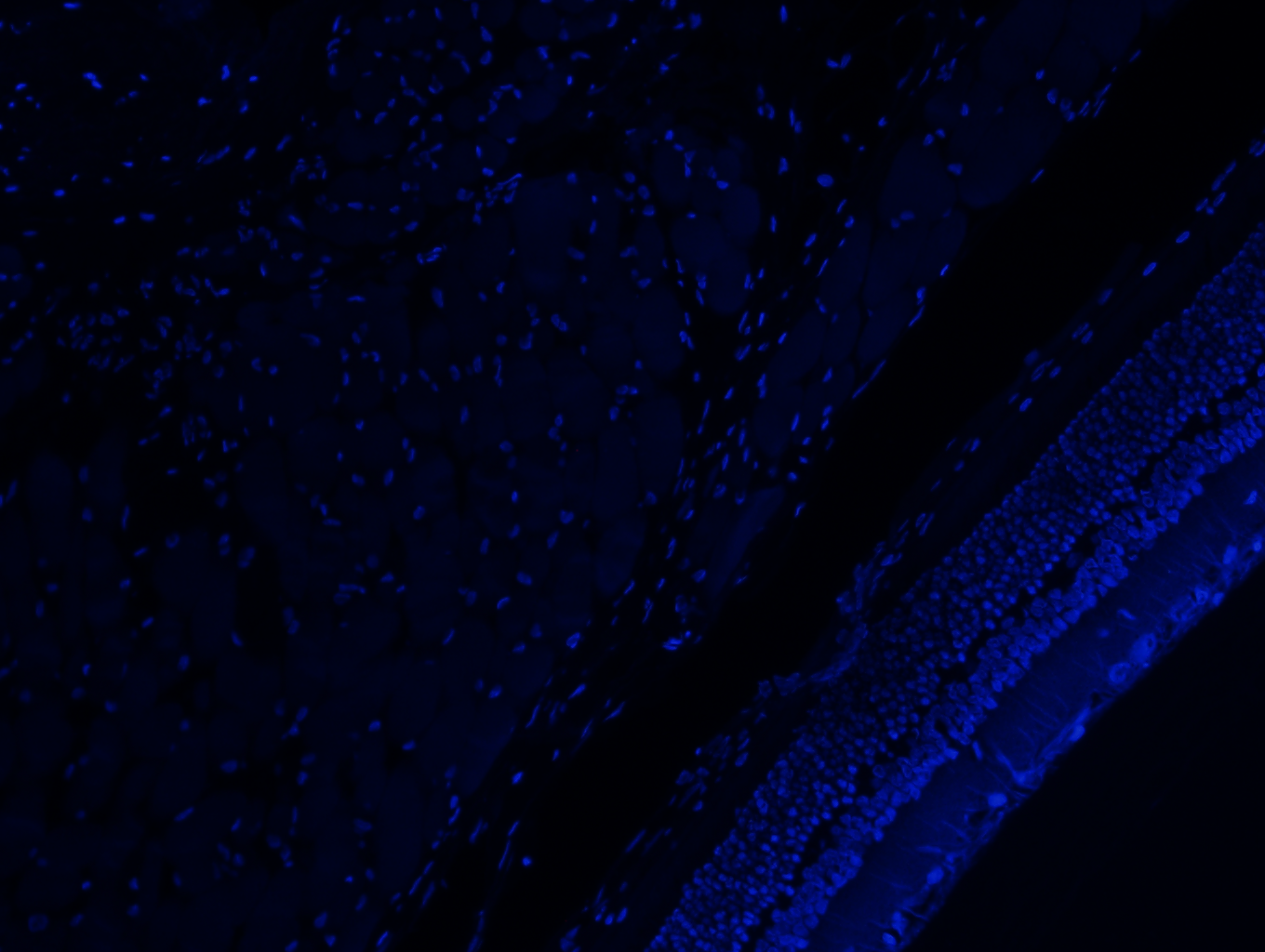

Supplement: S1 File — (ZIP) [file pone.0312791.s001.zip › Fig 4/Fig4 FISH/OENC DAPI-2.tif]

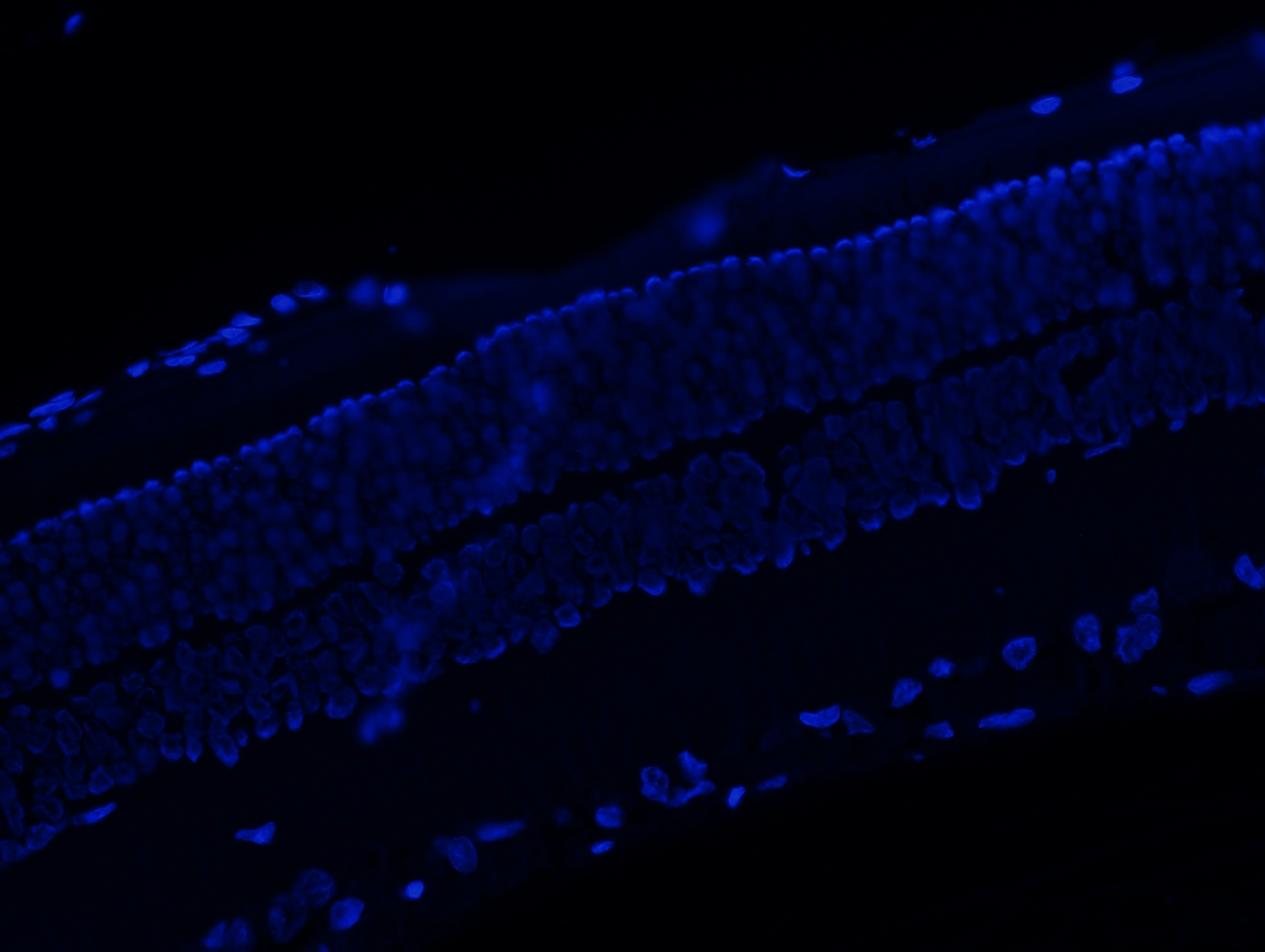

Supplement: S1 File — (ZIP) [file pone.0312791.s001.zip › Fig 4/Fig4 FISH/OENC DAPI-3.tif]

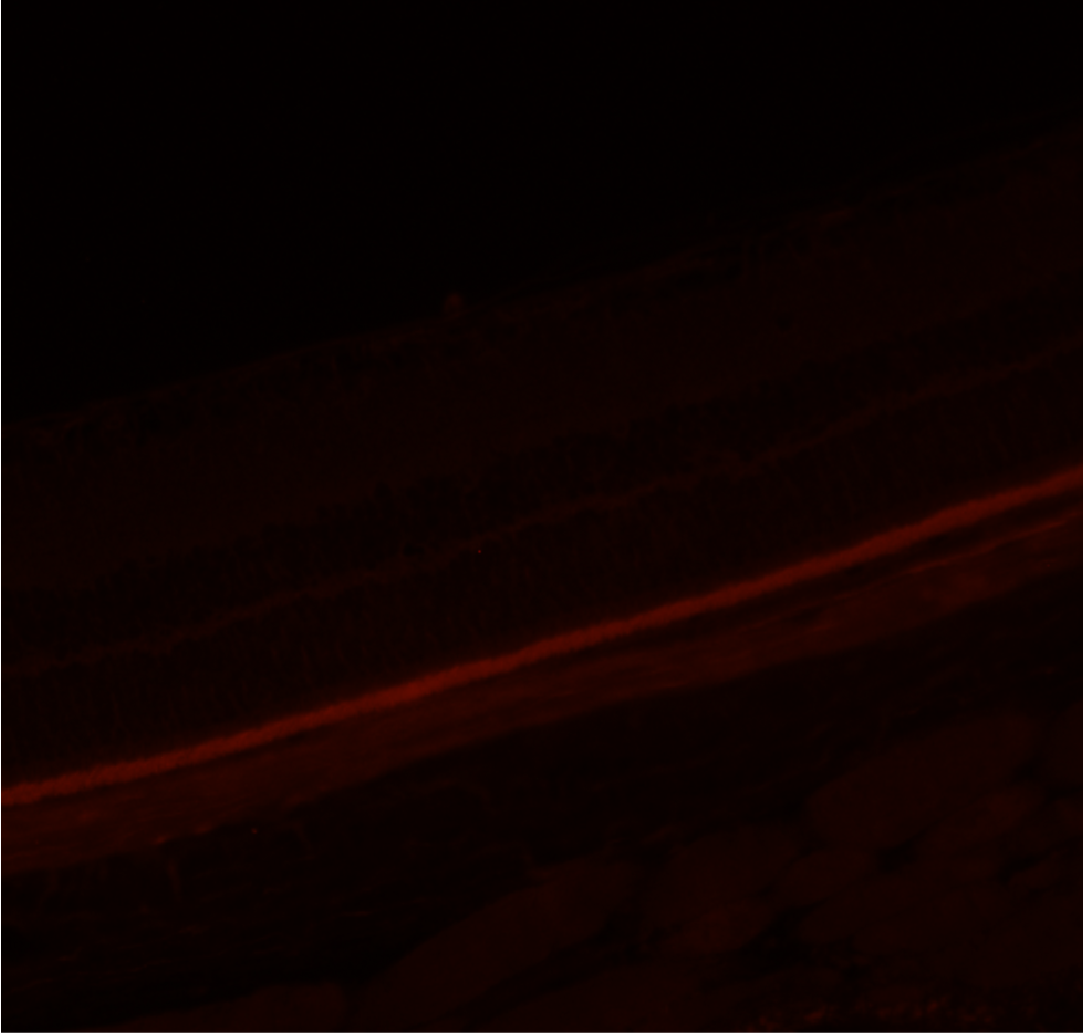

Supplement: S1 File — (ZIP) [file pone.0312791.s001.zip › Fig 4/Fig4 FISH/OENC RP11-1.tif]

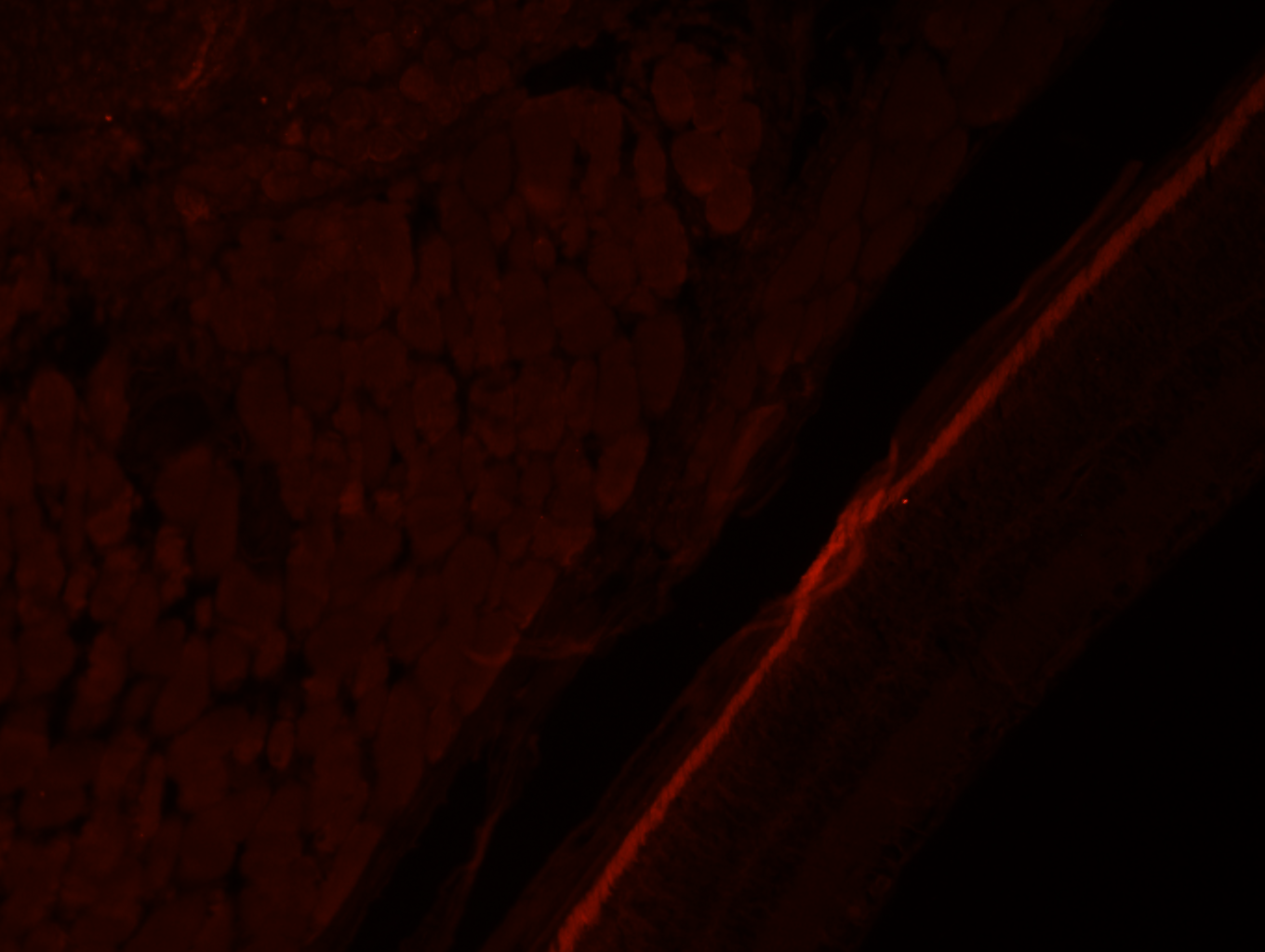

Supplement: S1 File — (ZIP) [file pone.0312791.s001.zip › Fig 4/Fig4 FISH/OENC RP11-2.tif]

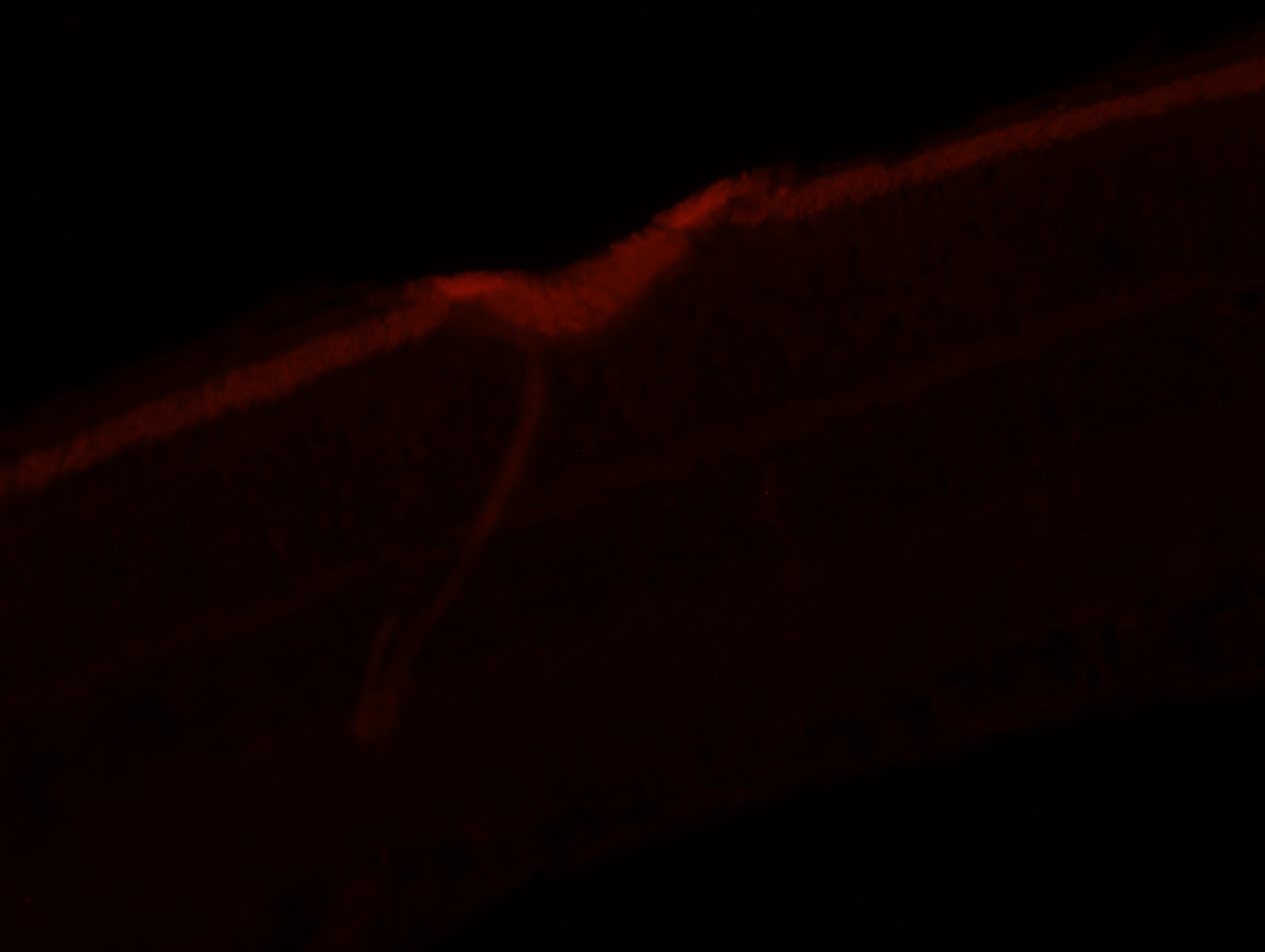

Supplement: S1 File — (ZIP) [file pone.0312791.s001.zip › Fig 4/Fig4 FISH/OENC RP11-3.tif]

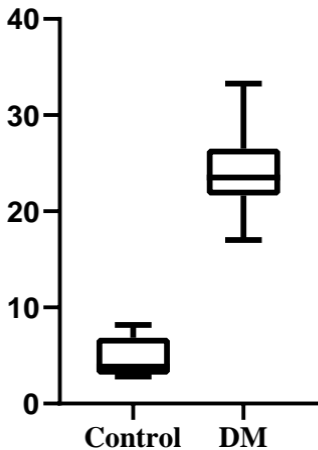

Supplement: S1 File — (ZIP) [file pone.0312791.s001.zip › Fig 2/blood glucose and weight/BLOOD GLUCOSE.pdf]

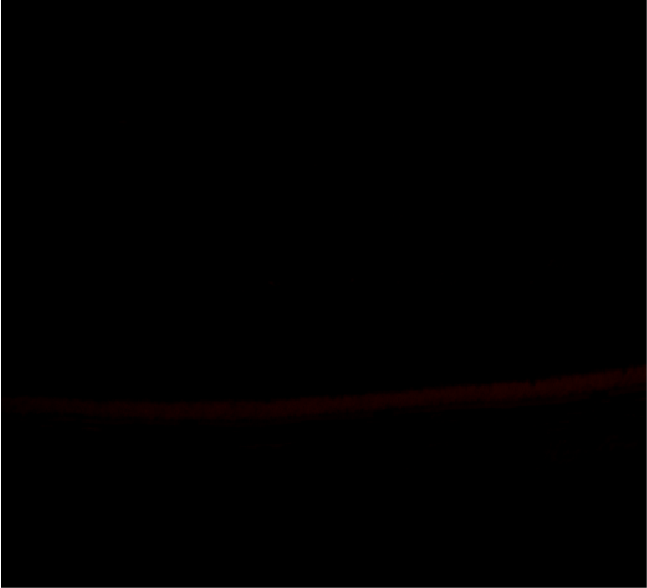

Supplement: S1 File — (ZIP) [file pone.0312791.s001.zip › Fig 2/Fig2 FISH/DM NC-1.tif]

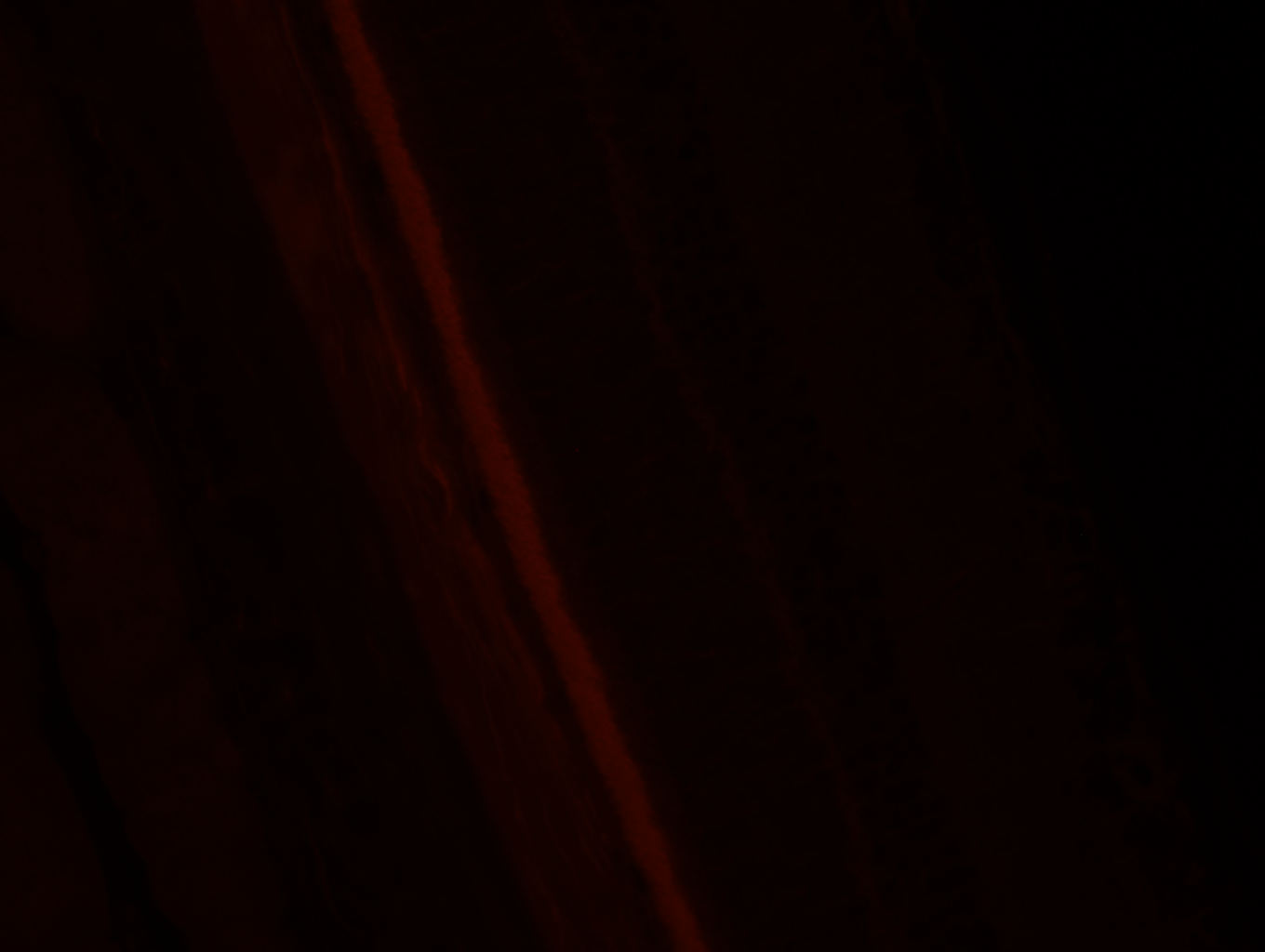

Supplement: S1 File — (ZIP) [file pone.0312791.s001.zip › Fig 2/Fig2 FISH/DM NC-2.tif]

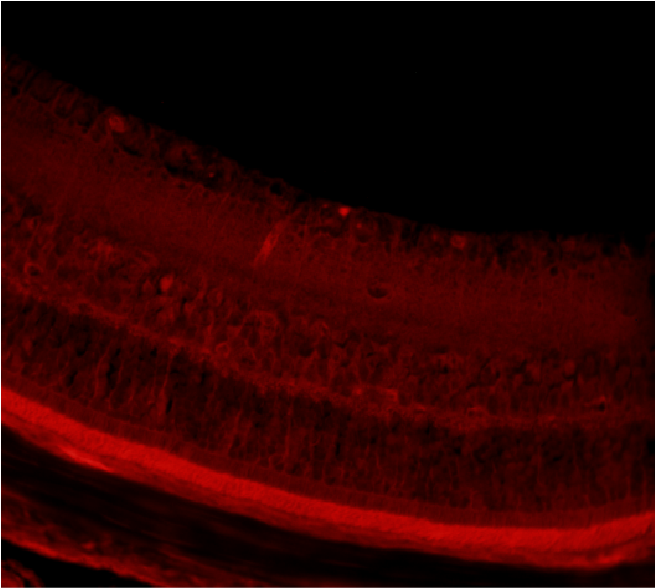

Supplement: S1 File — (ZIP) [file pone.0312791.s001.zip › Fig 2/Fig2 FISH/DM RP11-1.tif]

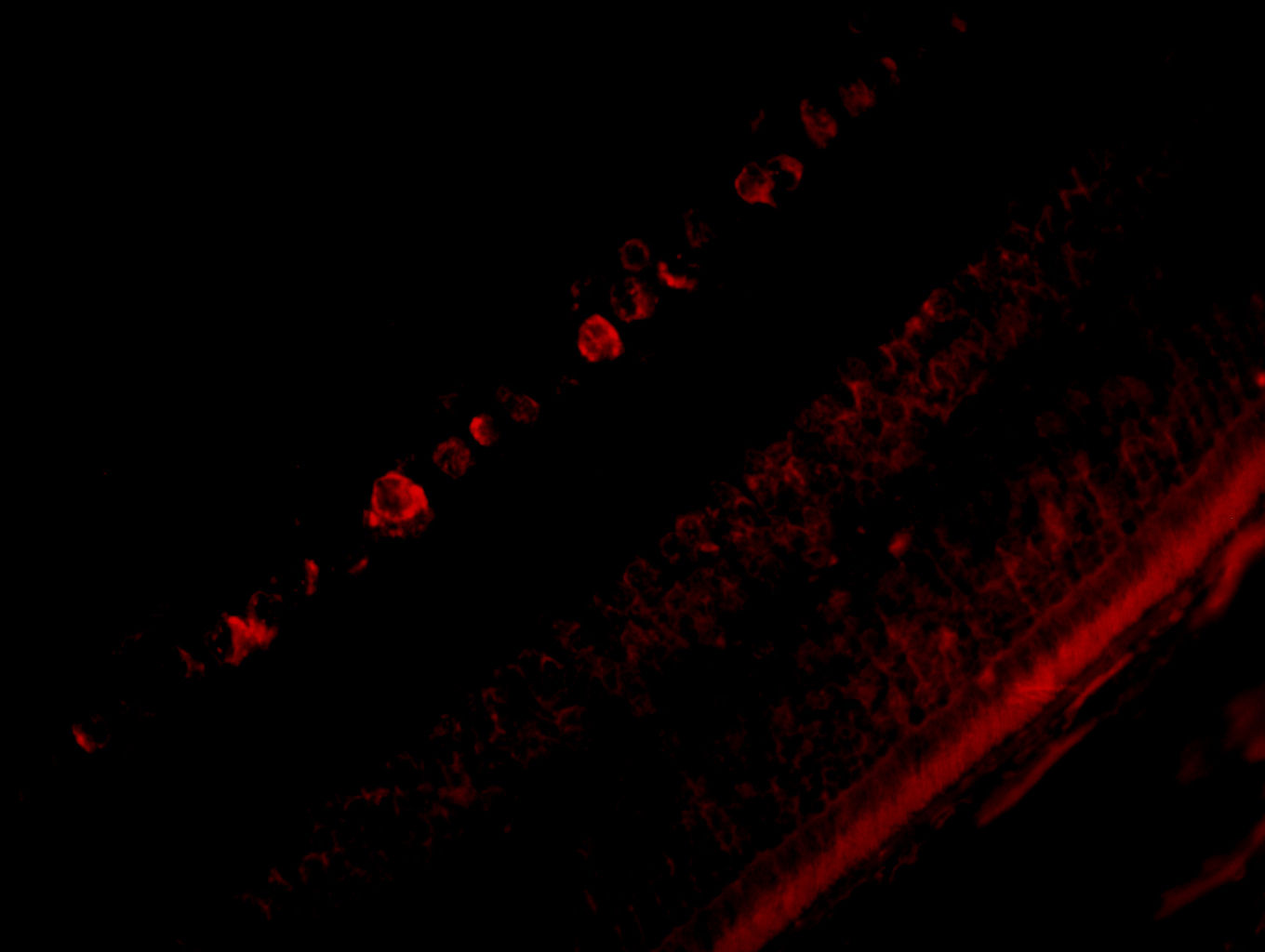

Supplement: S1 File — (ZIP) [file pone.0312791.s001.zip › Fig 2/Fig2 FISH/DM RP11-2.tif]

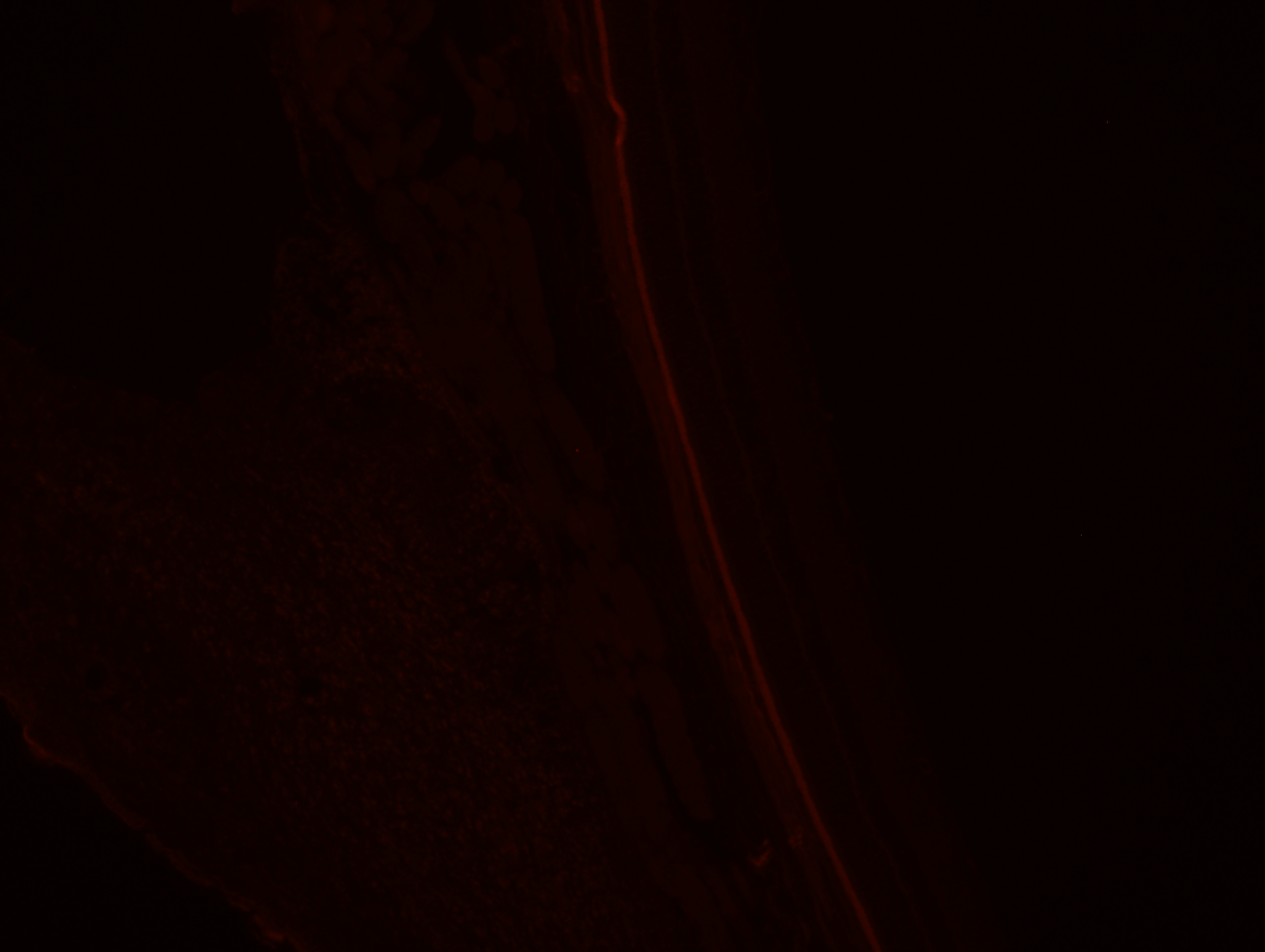

Supplement: S1 File — (ZIP) [file pone.0312791.s001.zip › Fig 2/Fig2 FISH/DM RP11-3.tif]

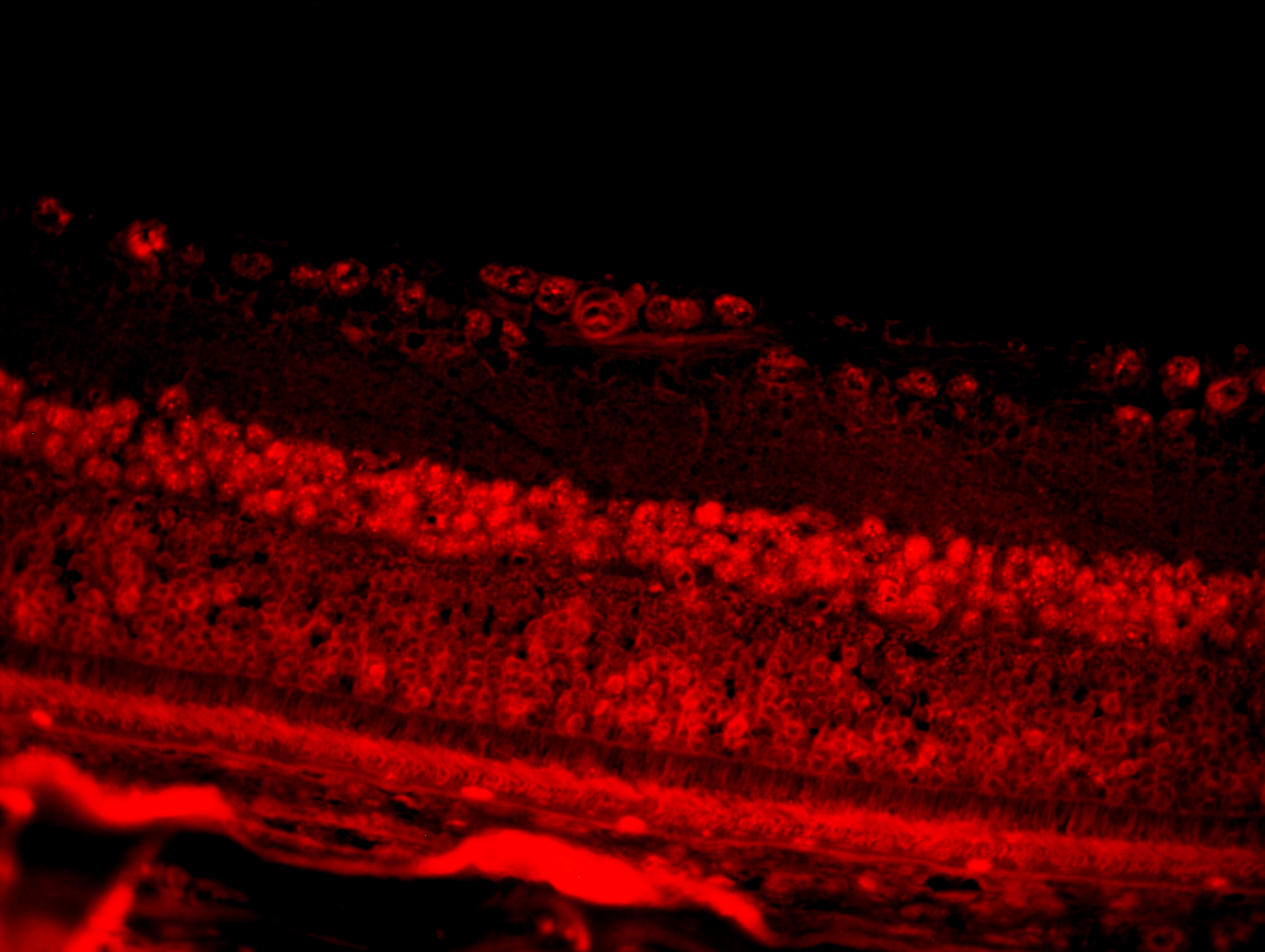

Supplement: S1 File — (ZIP) [file pone.0312791.s001.zip › Fig 2/Fig2 FISH/DM U6.tif]

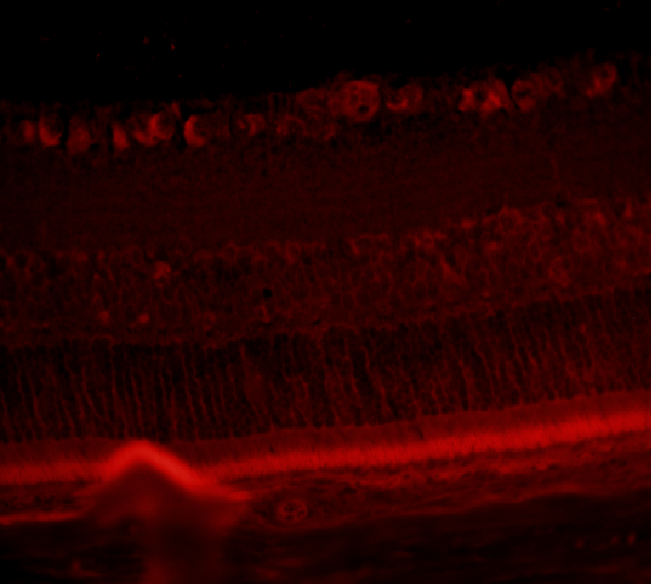

Supplement: S1 File — (ZIP) [file pone.0312791.s001.zip › Fig 2/Fig2 FISH/DM18S.tif]

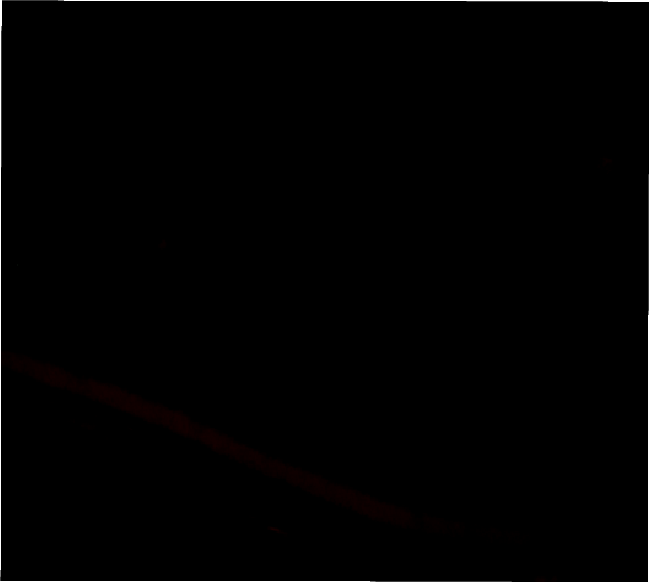

Supplement: S1 File — (ZIP) [file pone.0312791.s001.zip › Fig 2/Fig2 FISH/WT NC-1.tif]

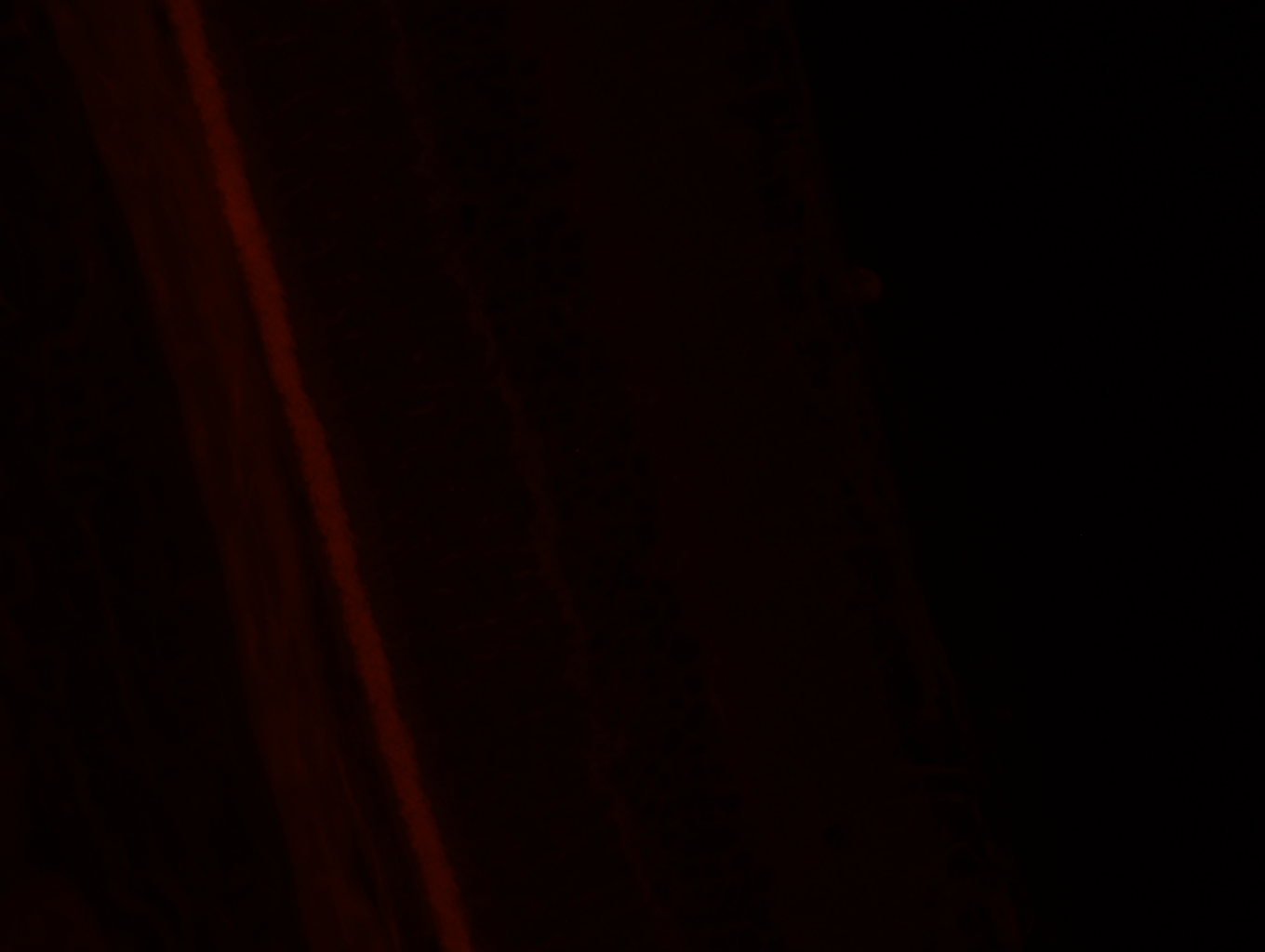

Supplement: S1 File — (ZIP) [file pone.0312791.s001.zip › Fig 2/Fig2 FISH/WT NC-3.tif]

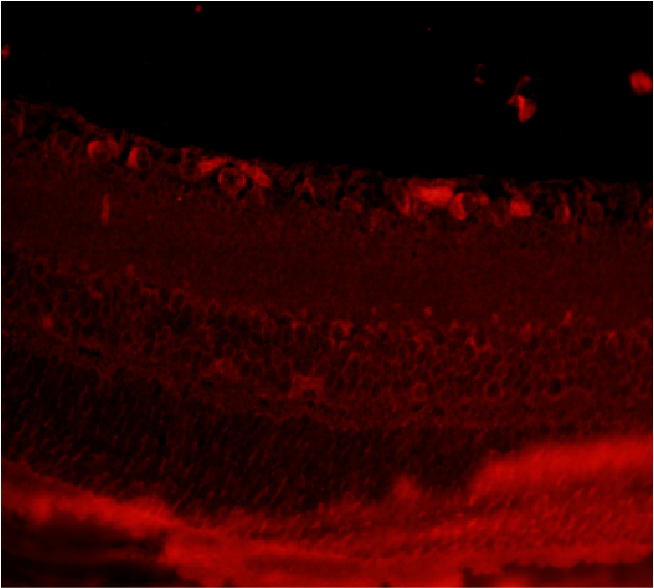

Supplement: S1 File — (ZIP) [file pone.0312791.s001.zip › Fig 2/Fig2 FISH/WT RP11-1.tif]

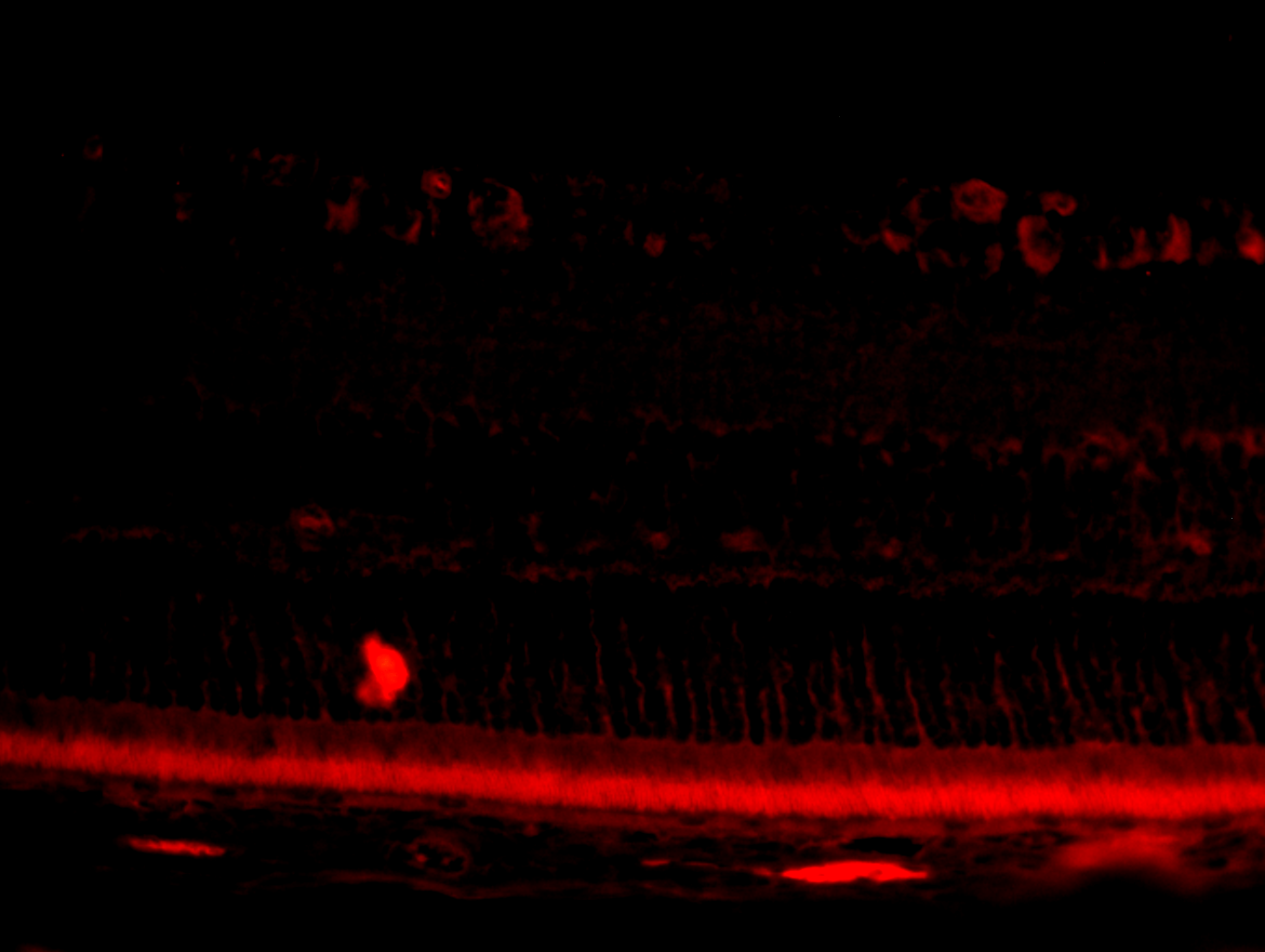

Supplement: S1 File — (ZIP) [file pone.0312791.s001.zip › Fig 2/Fig2 FISH/WT RP11-2.tif]

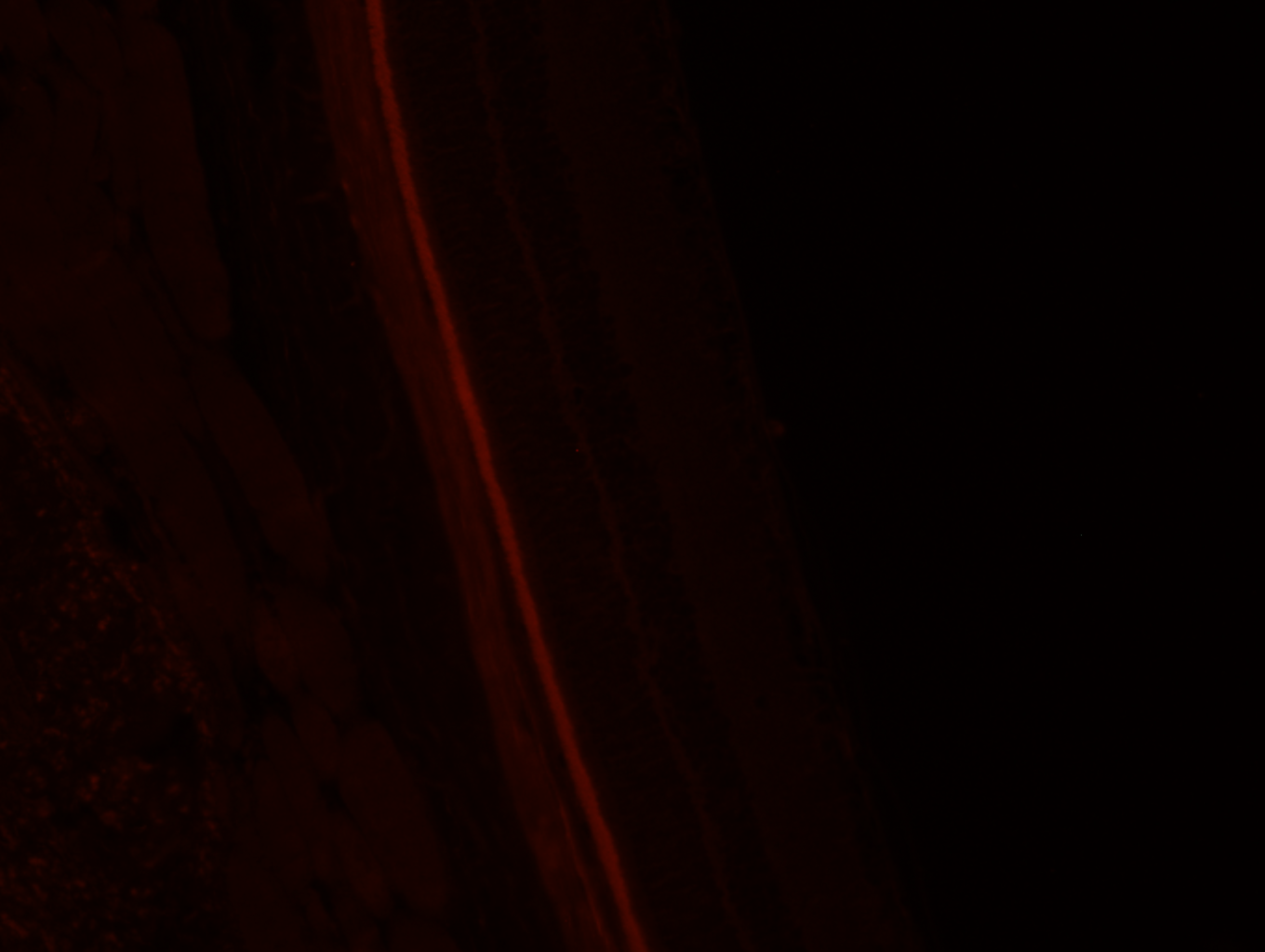

Supplement: S1 File — (ZIP) [file pone.0312791.s001.zip › Fig 2/Fig2 FISH/WT RP11-3.tif]

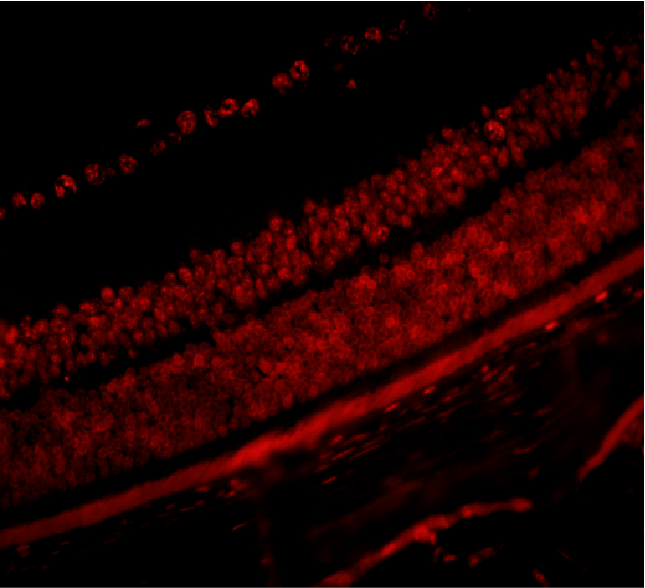

Supplement: S1 File — (ZIP) [file pone.0312791.s001.zip › Fig 2/Fig2 FISH/WT U6.tif]

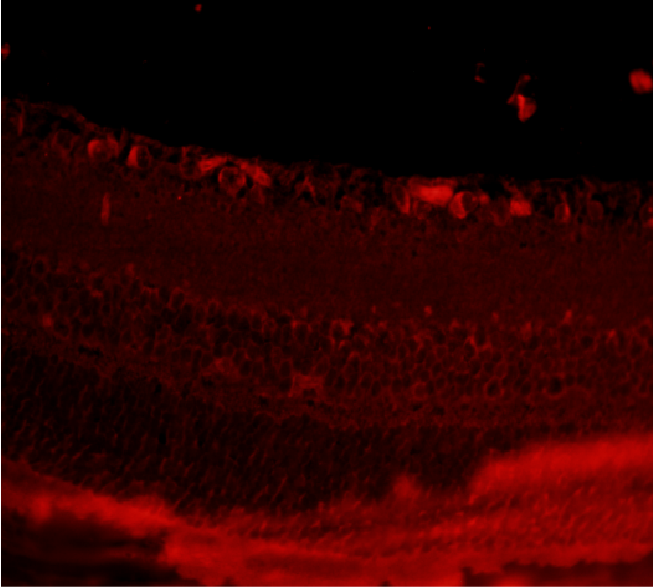

Supplement: S1 File — (ZIP) [file pone.0312791.s001.zip › Fig 2/Fig2 FISH/WT18S.tif]

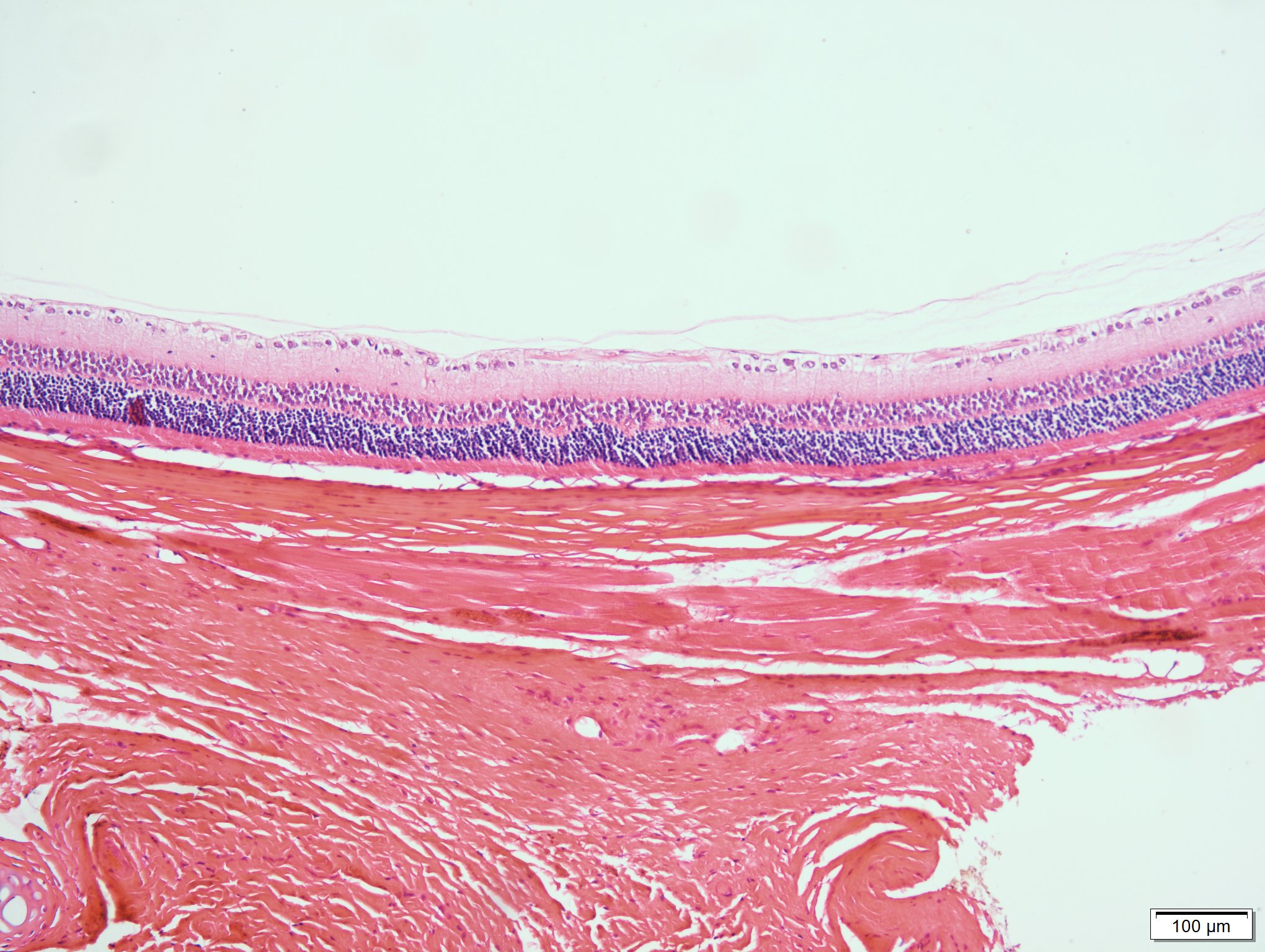

Supplement: S1 File — (ZIP) [file pone.0312791.s001.zip › Fig 2/Fig2 HE/DM1 100um.jpg]

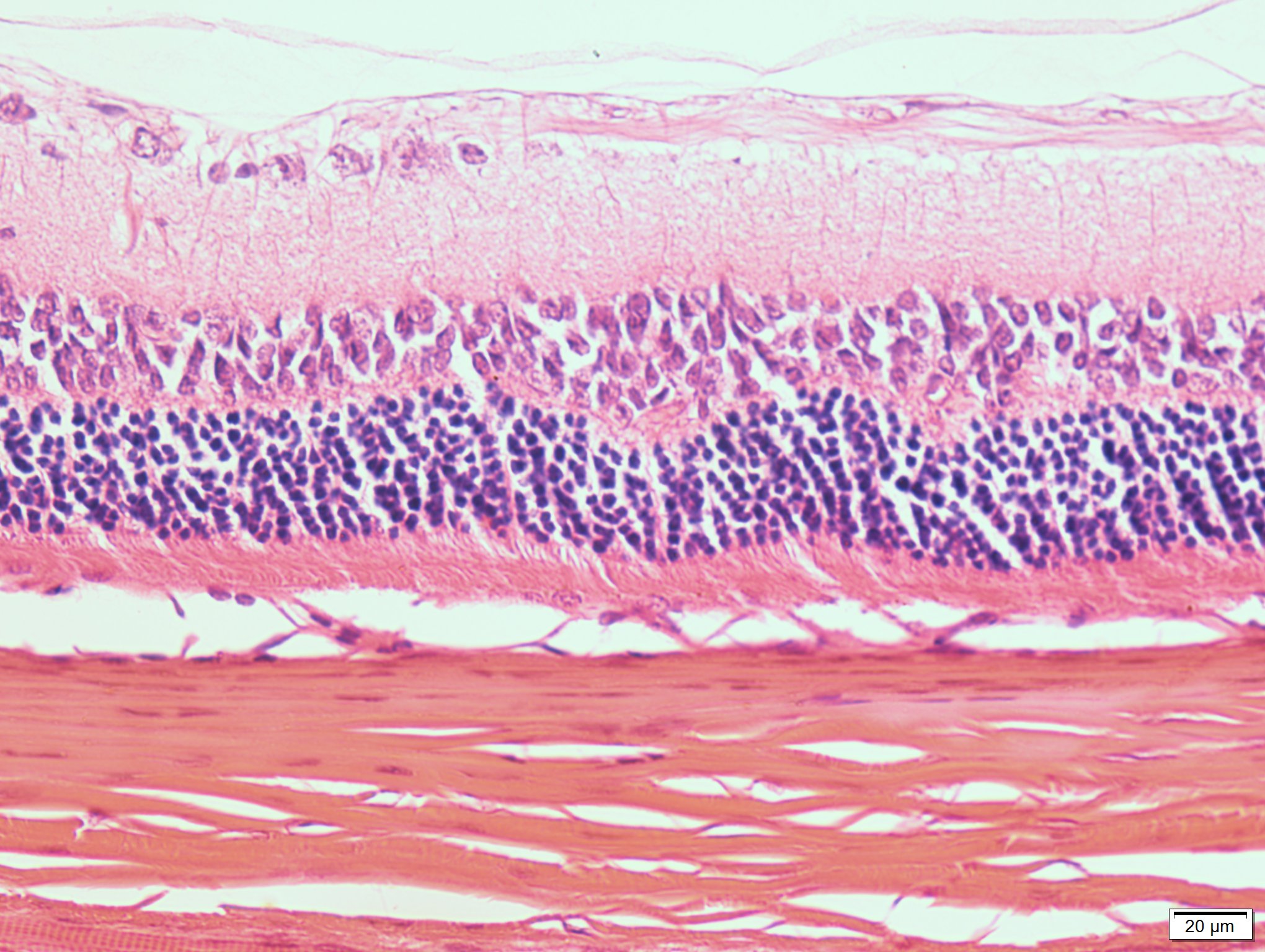

Supplement: S1 File — (ZIP) [file pone.0312791.s001.zip › Fig 2/Fig2 HE/DM1 20um.jpg]

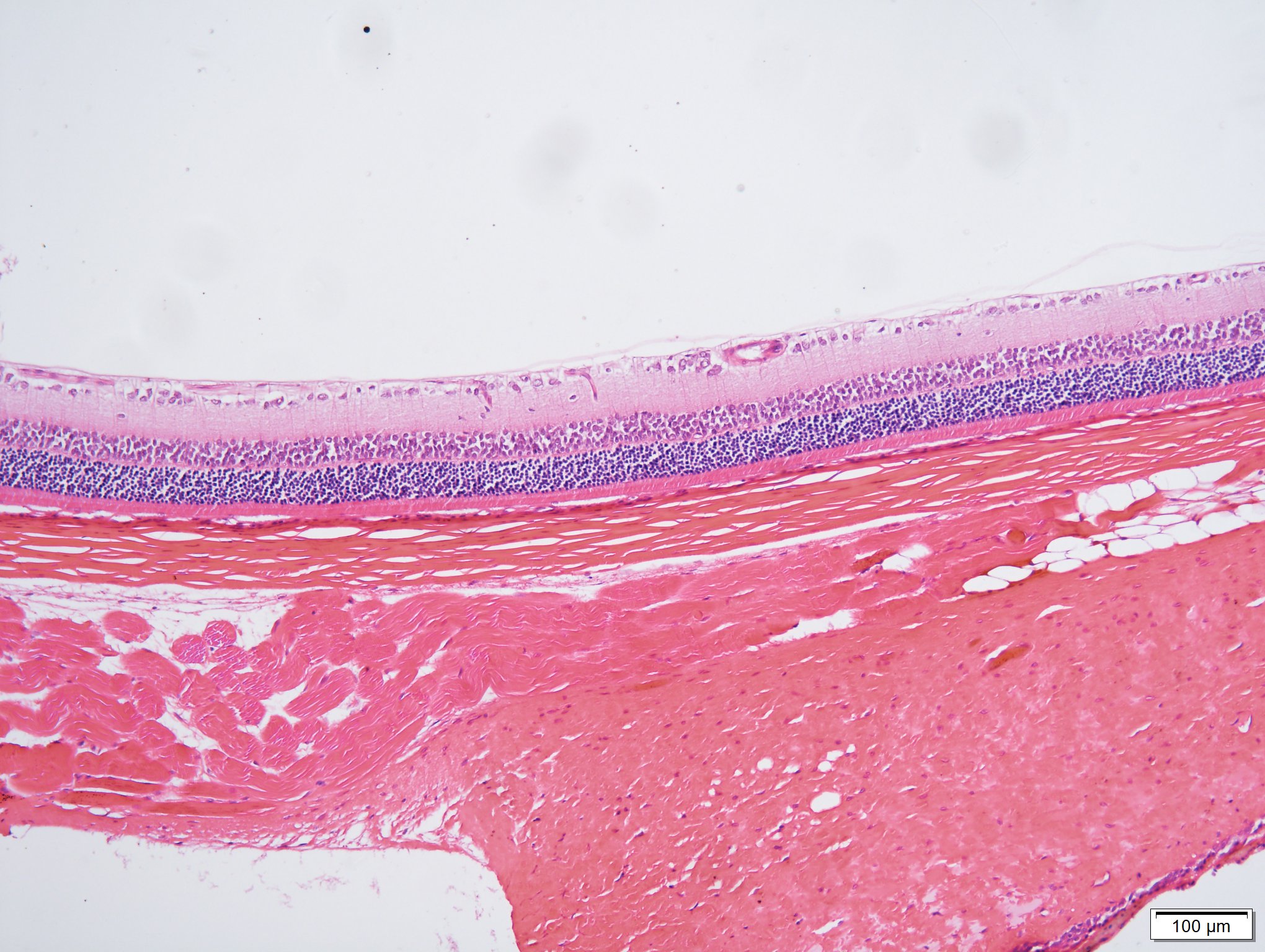

Supplement: S1 File — (ZIP) [file pone.0312791.s001.zip › Fig 2/Fig2 HE/DM2 100um.jpg]

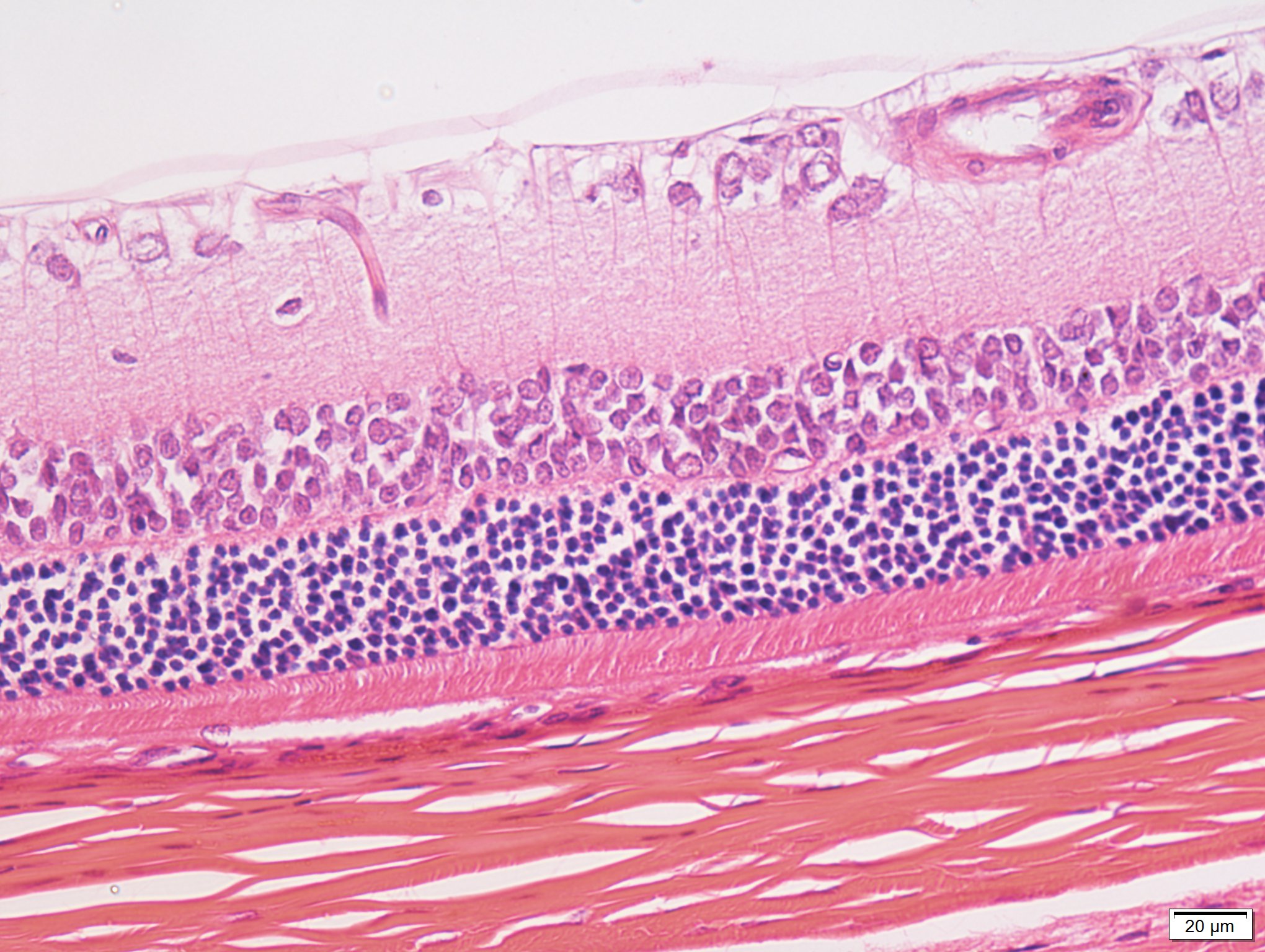

Supplement: S1 File — (ZIP) [file pone.0312791.s001.zip › Fig 2/Fig2 HE/DM2 20um.jpg]

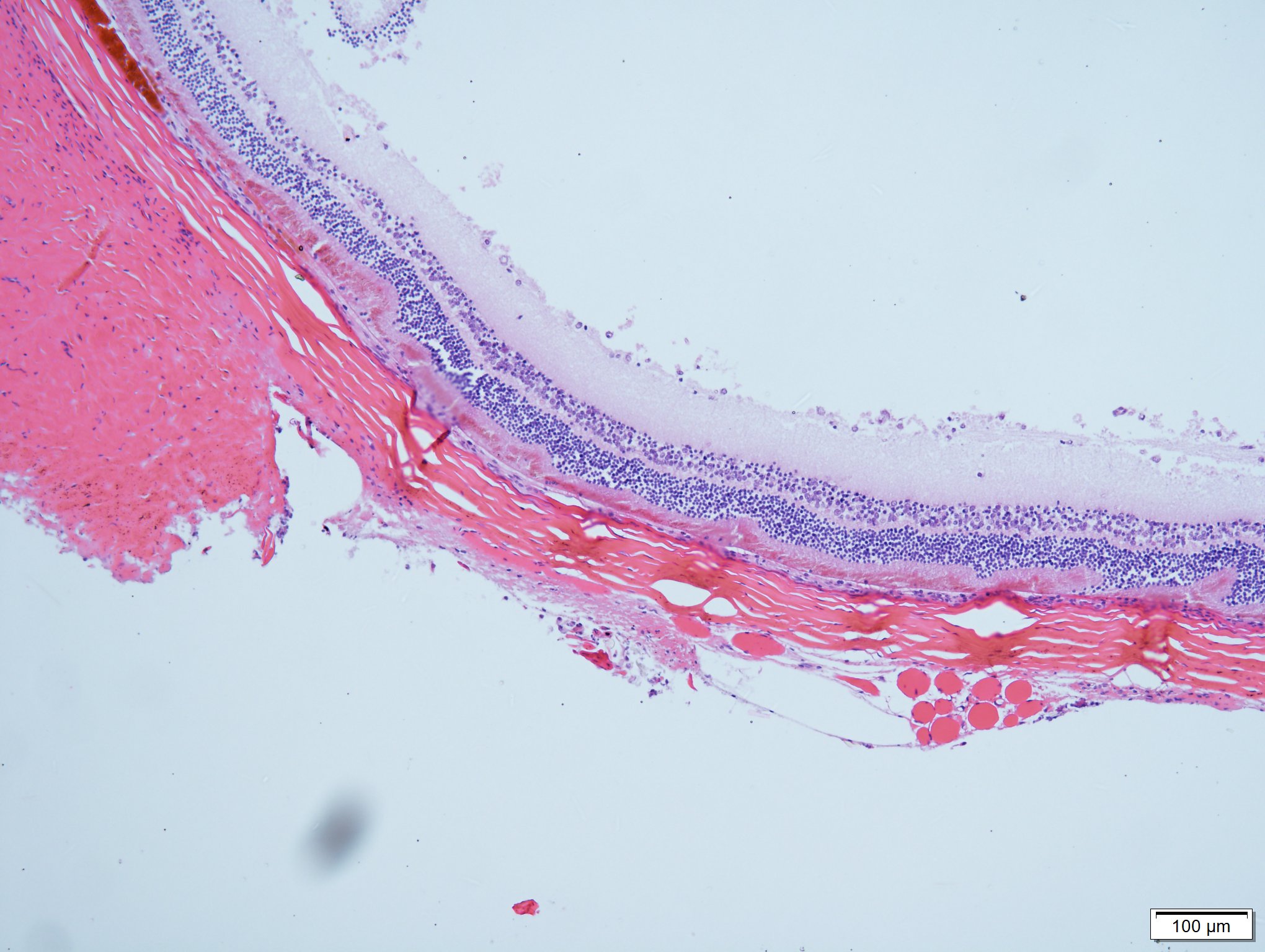

Supplement: S1 File — (ZIP) [file pone.0312791.s001.zip › Fig 2/Fig2 HE/DM3 100um.jpg]

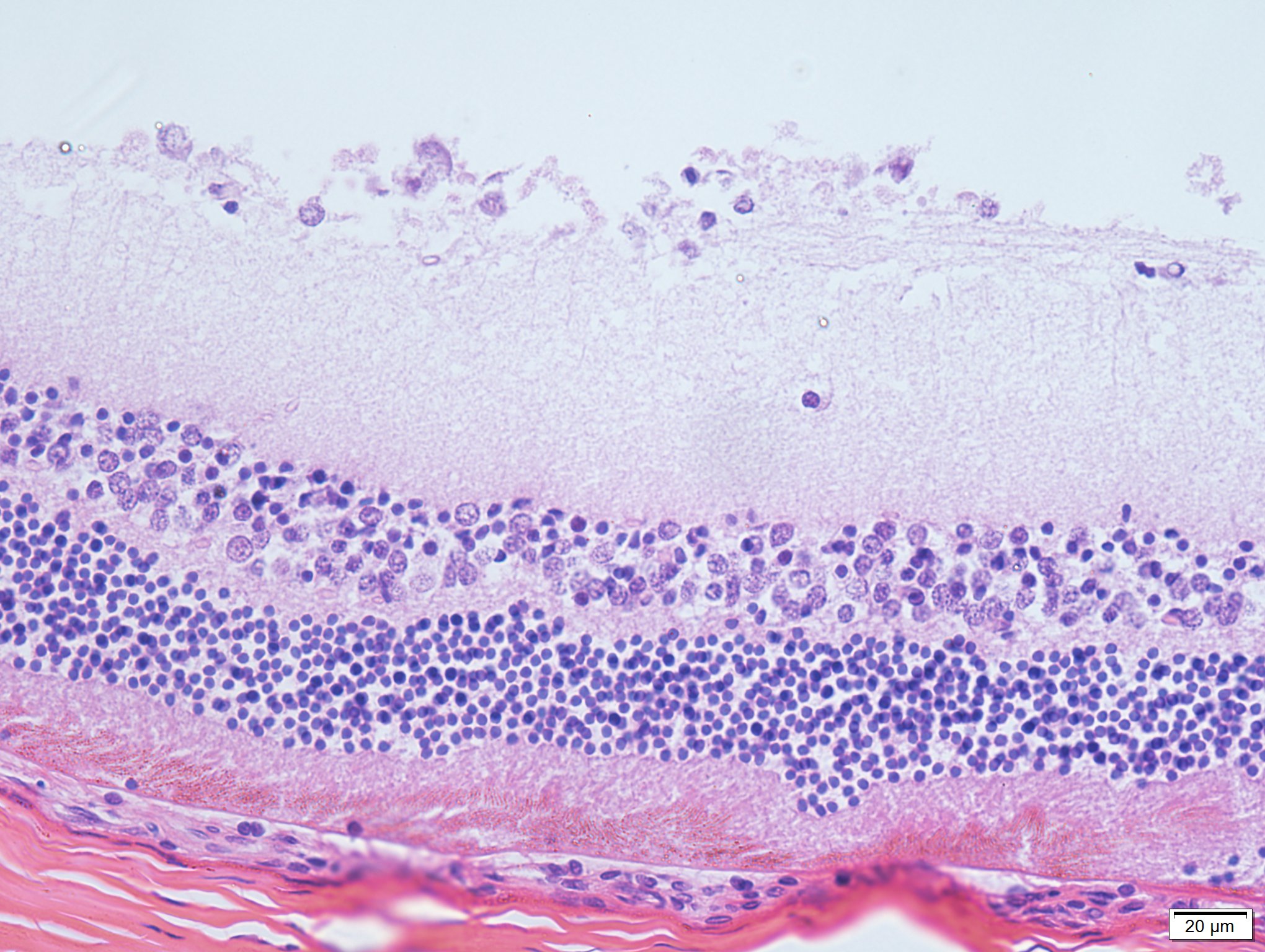

Supplement: S1 File — (ZIP) [file pone.0312791.s001.zip › Fig 2/Fig2 HE/DM3 20um.jpg]

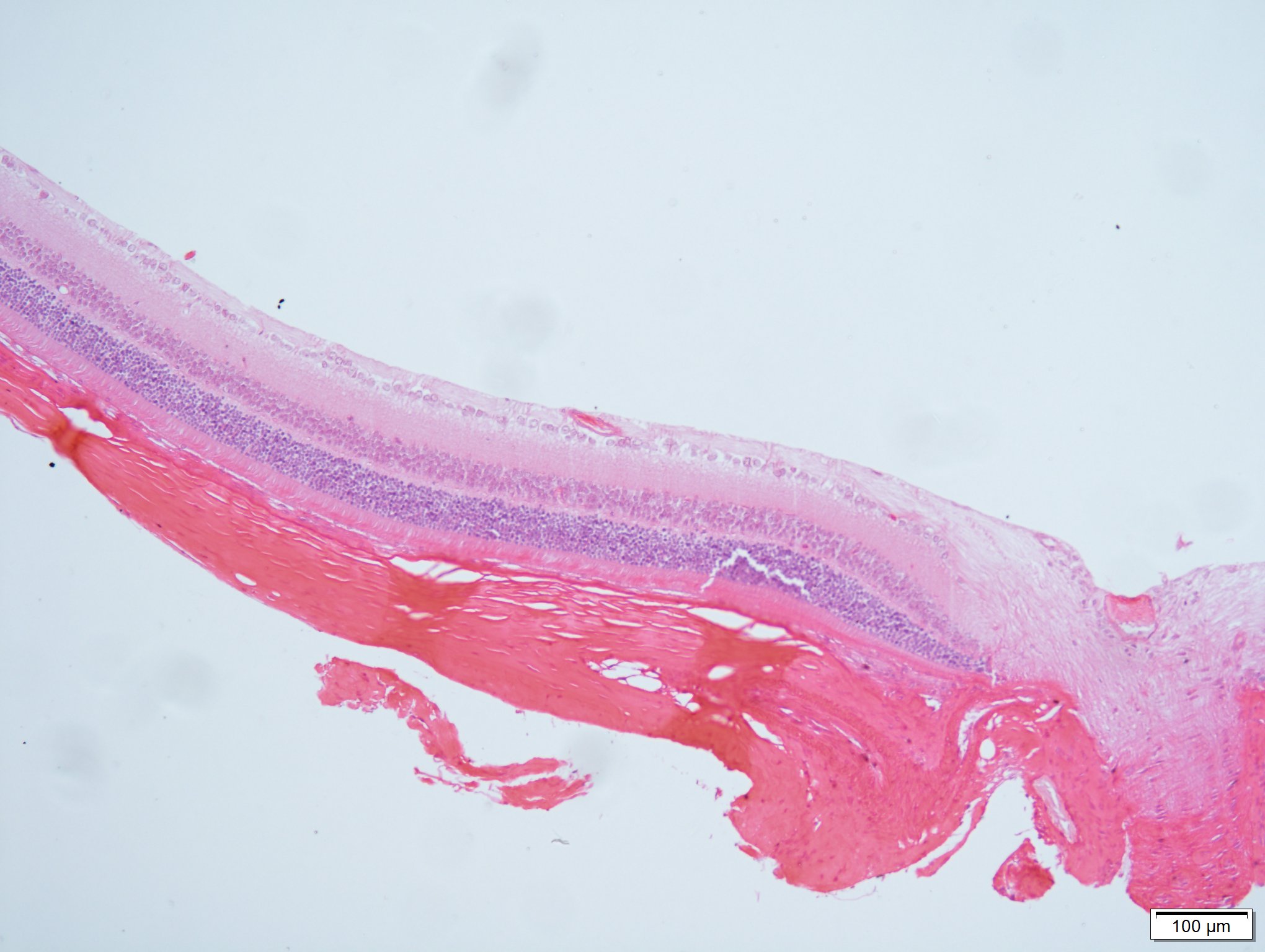

Supplement: S1 File — (ZIP) [file pone.0312791.s001.zip › Fig 2/Fig2 HE/WT1 100um.jpg]

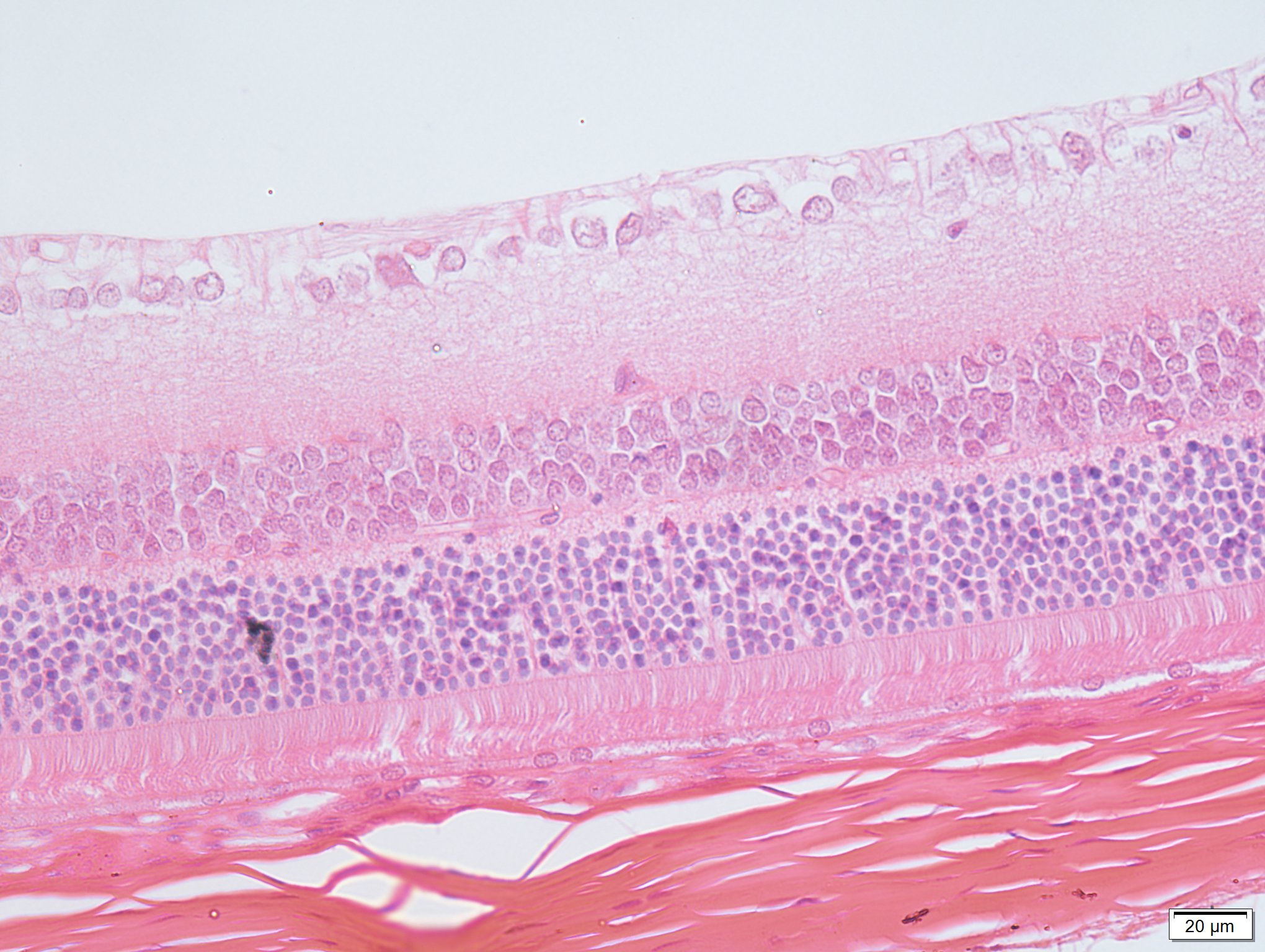

Supplement: S1 File — (ZIP) [file pone.0312791.s001.zip › Fig 2/Fig2 HE/WT1 20um.jpg]

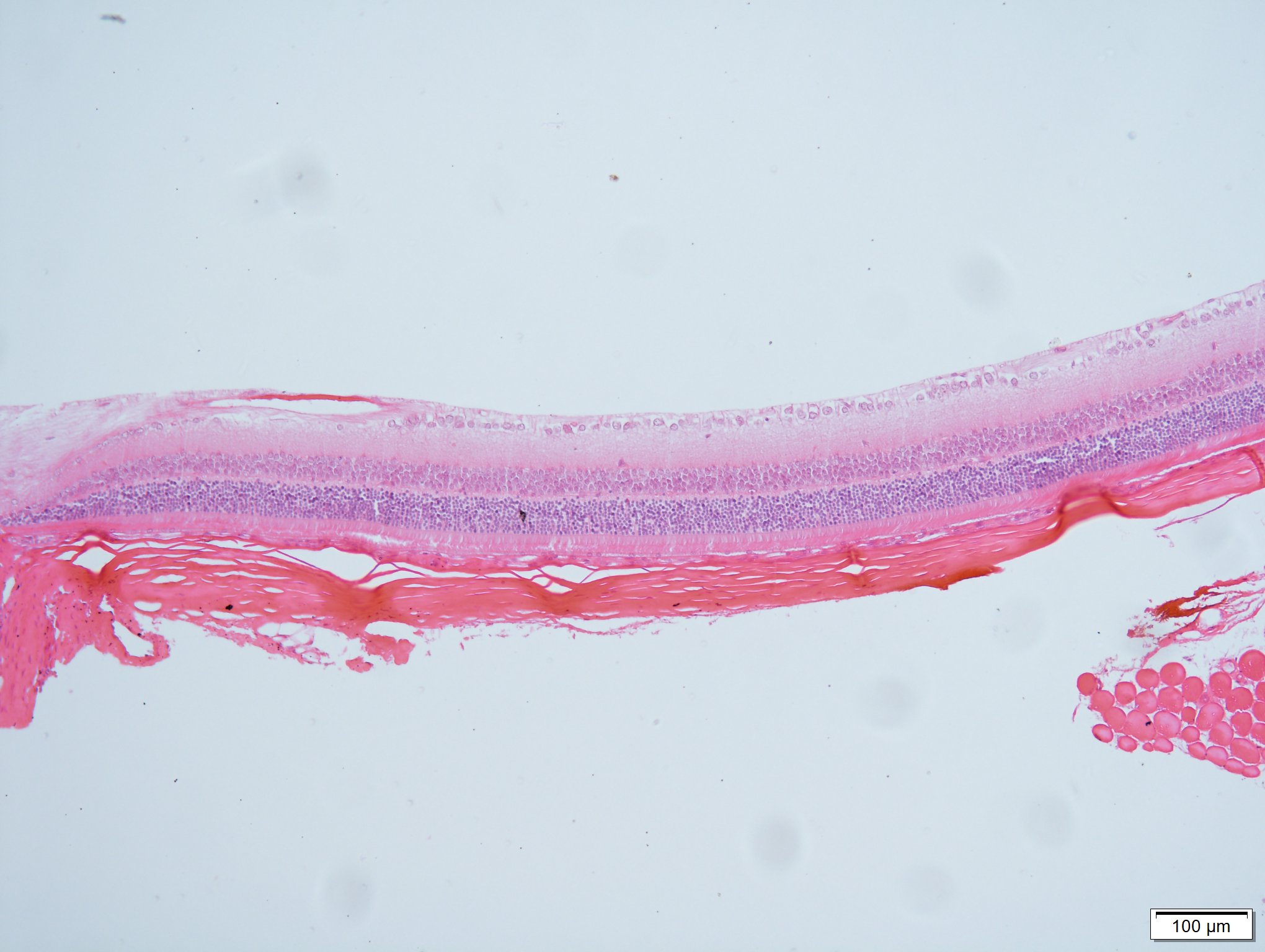

Supplement: S1 File — (ZIP) [file pone.0312791.s001.zip › Fig 2/Fig2 HE/WT2 100um.jpg]

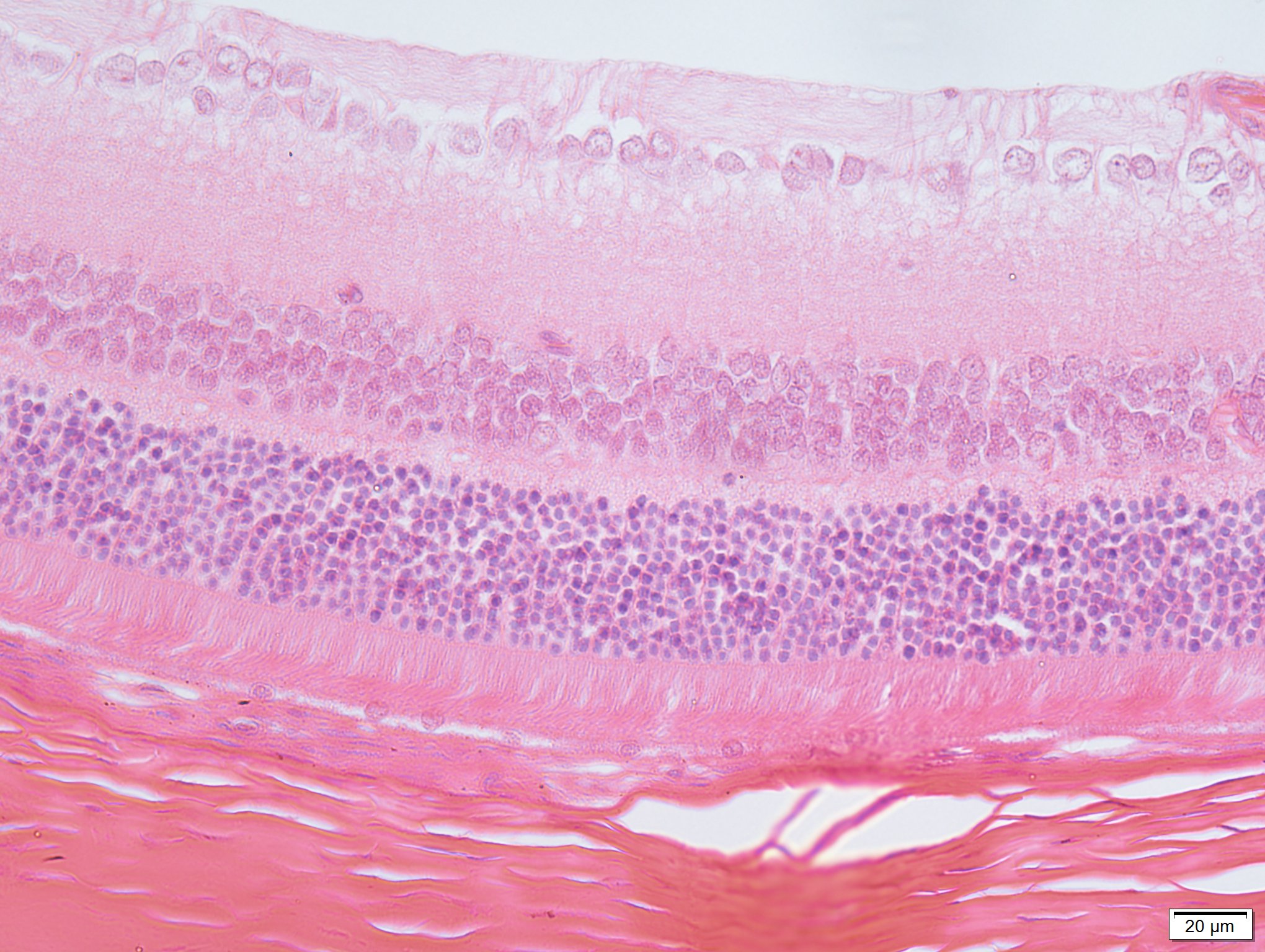

Supplement: S1 File — (ZIP) [file pone.0312791.s001.zip › Fig 2/Fig2 HE/WT2 20um.jpg]

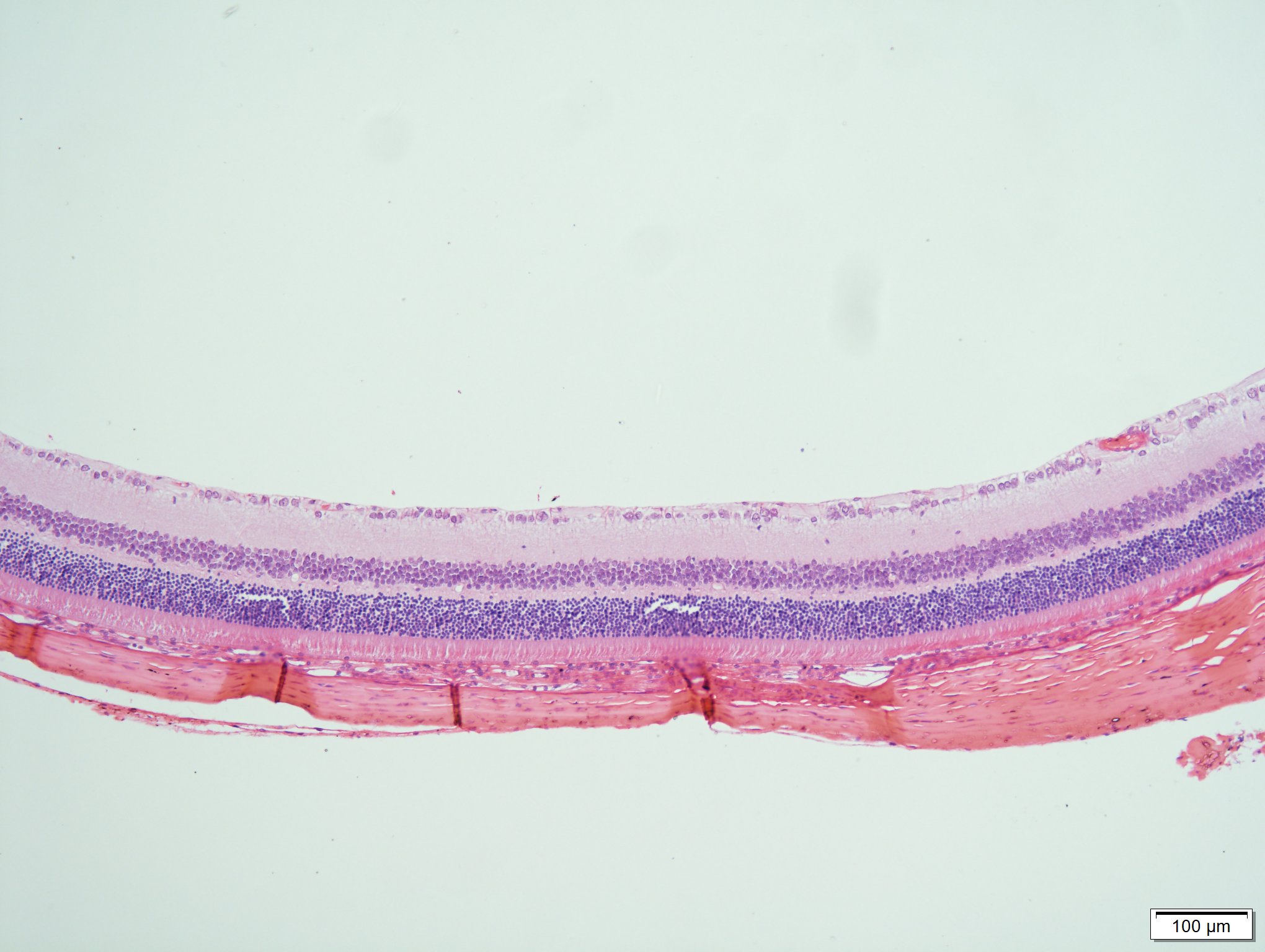

Supplement: S1 File — (ZIP) [file pone.0312791.s001.zip › Fig 2/Fig2 HE/WT3 100um.jpg]

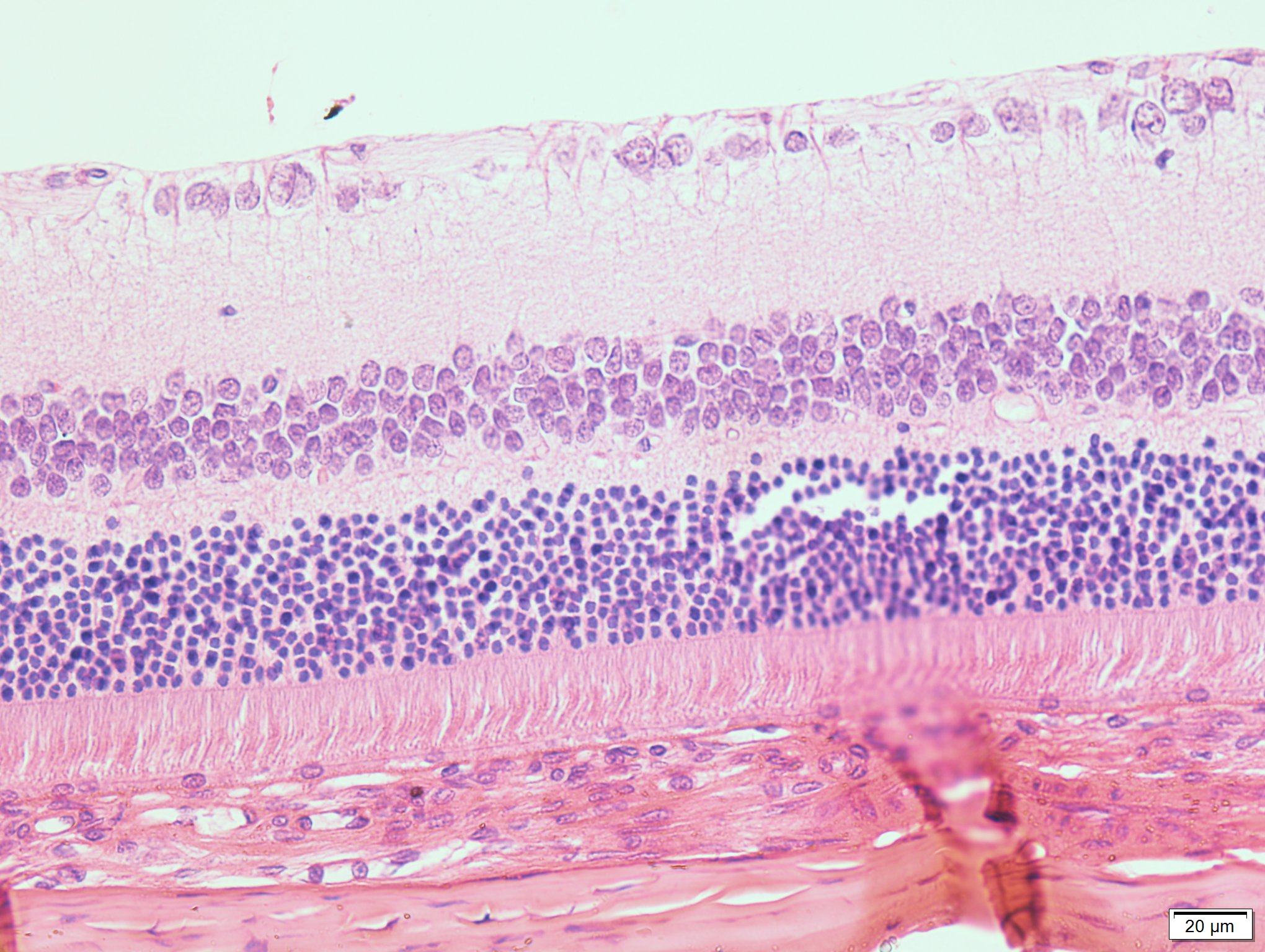

Supplement: S1 File — (ZIP) [file pone.0312791.s001.zip › Fig 2/Fig2 HE/WT3 20um.jpg]

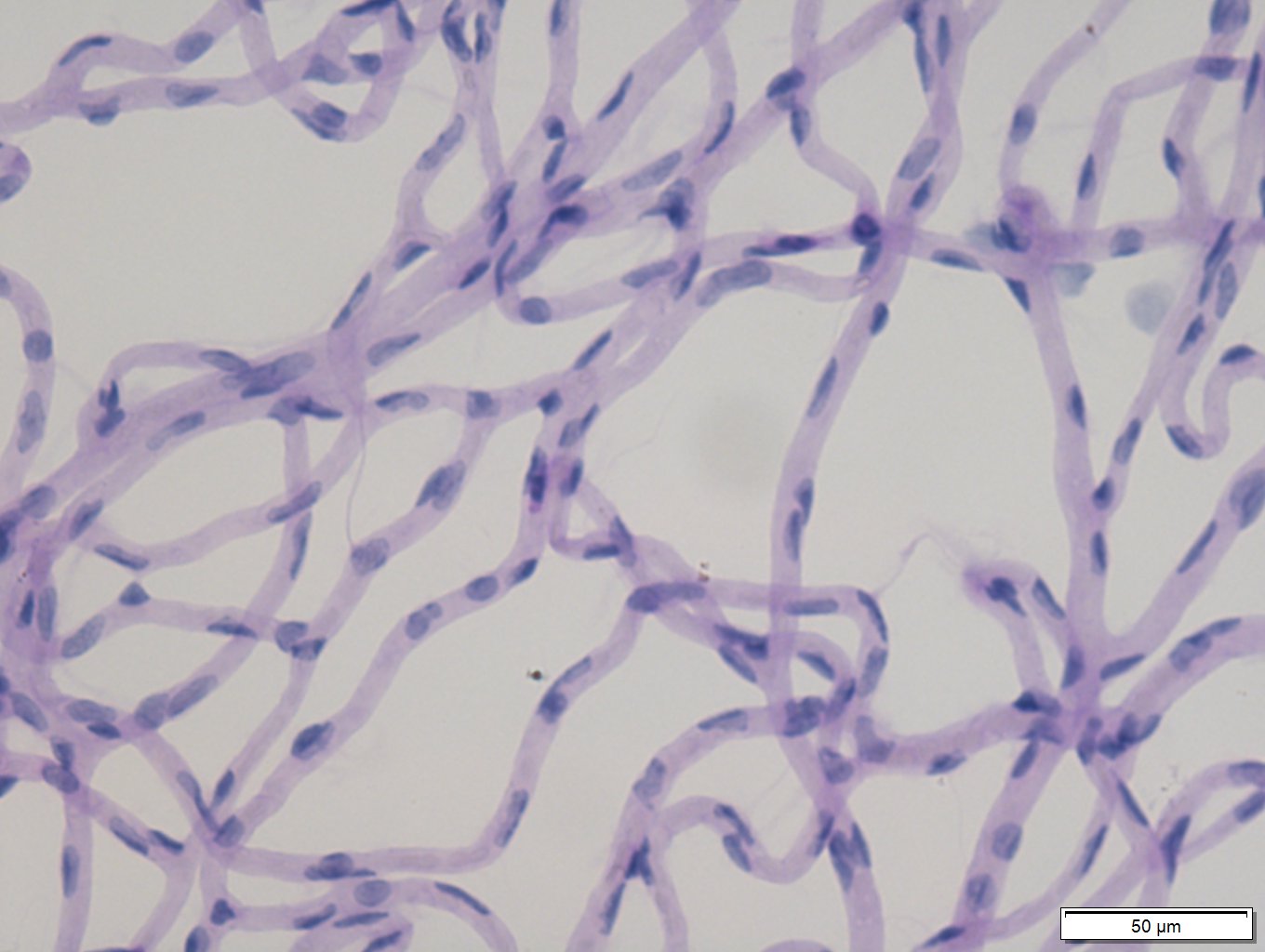

Supplement: S1 File — (ZIP) [file pone.0312791.s001.zip › Fig 2/Fig2 PAS/DM1.jpg]

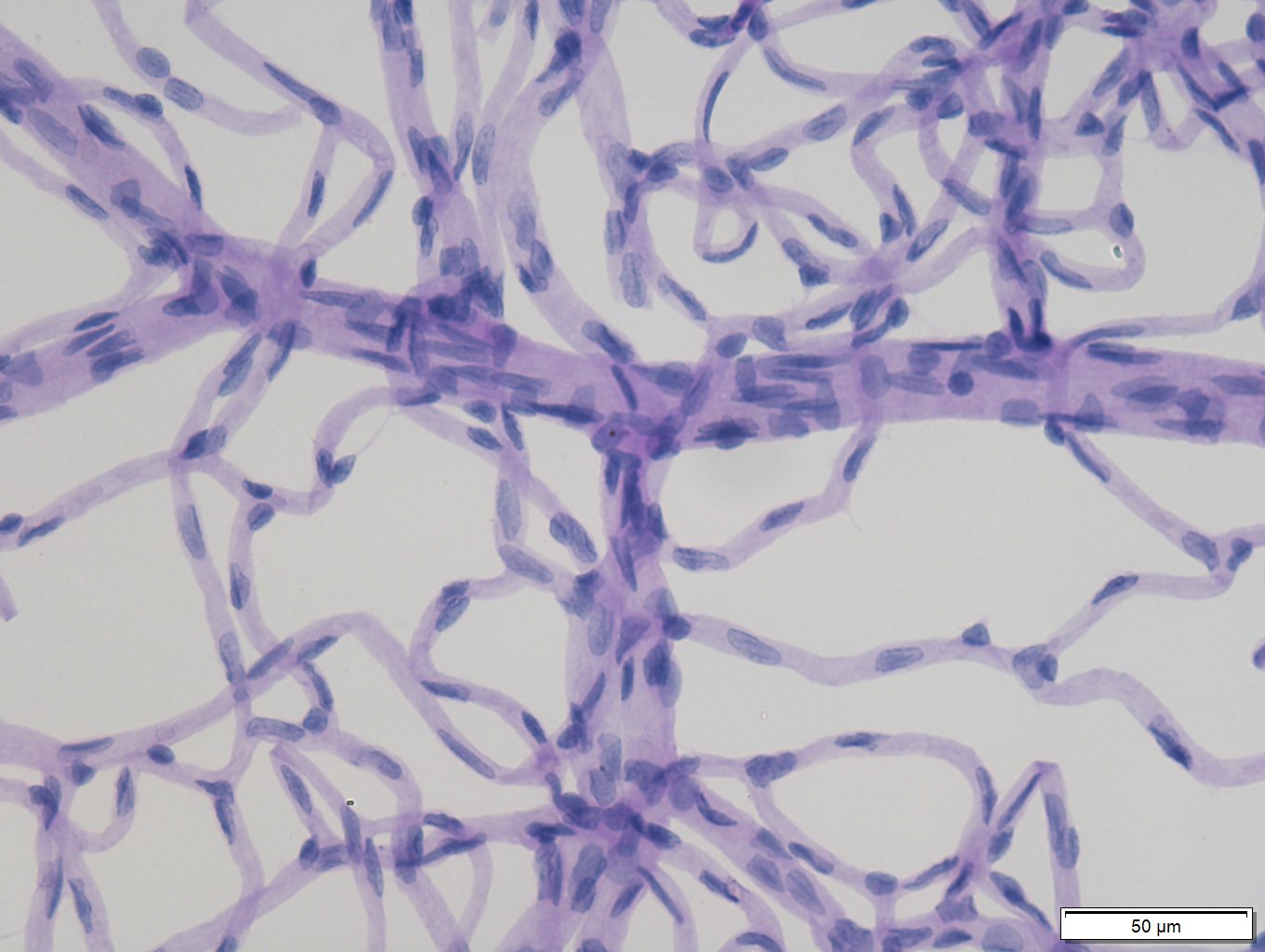

Supplement: S1 File — (ZIP) [file pone.0312791.s001.zip › Fig 2/Fig2 PAS/DM2.jpg]

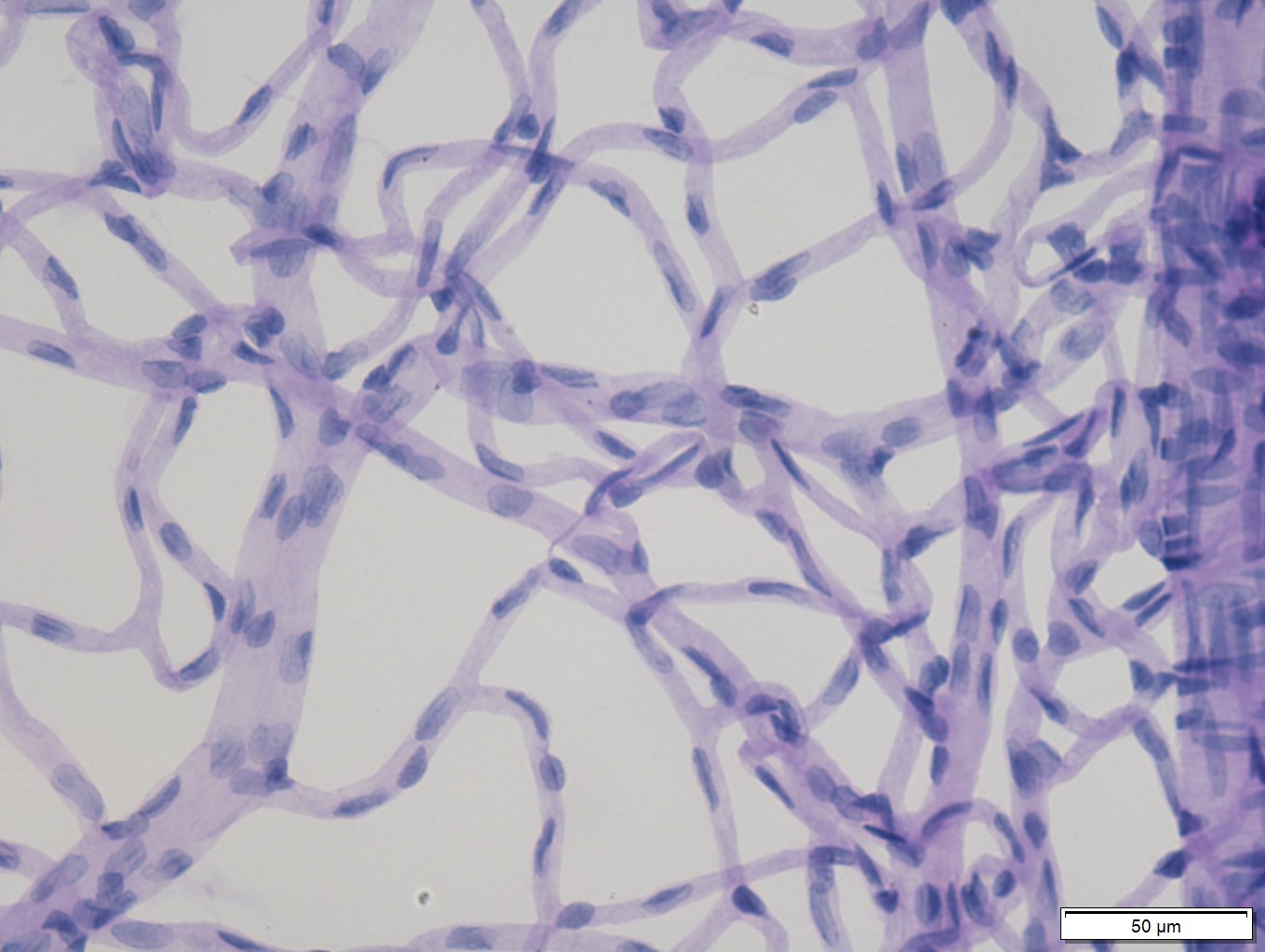

Supplement: S1 File — (ZIP) [file pone.0312791.s001.zip › Fig 2/Fig2 PAS/DM3.jpg]

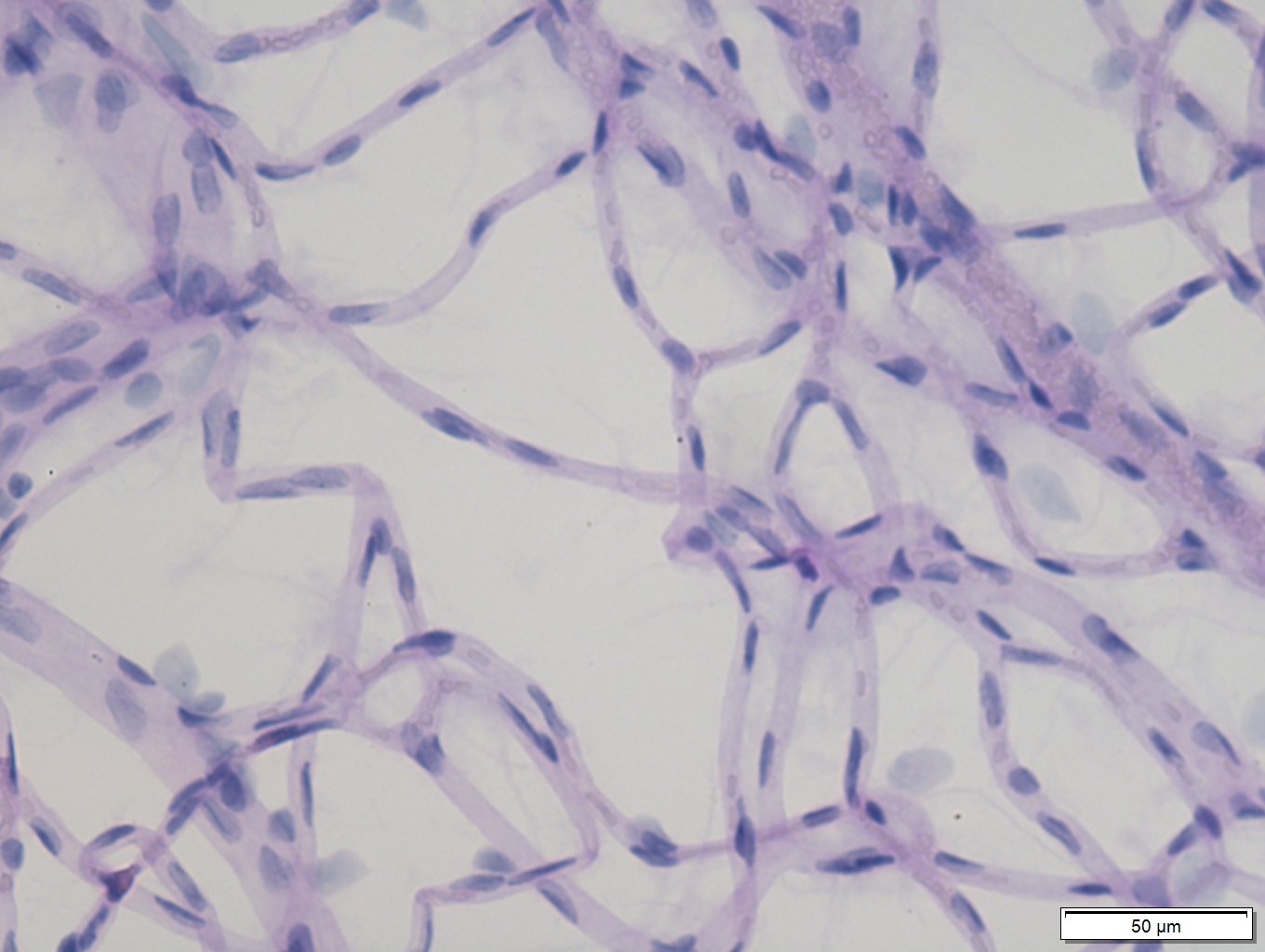

Supplement: S1 File — (ZIP) [file pone.0312791.s001.zip › Fig 2/Fig2 PAS/WT1.jpg]

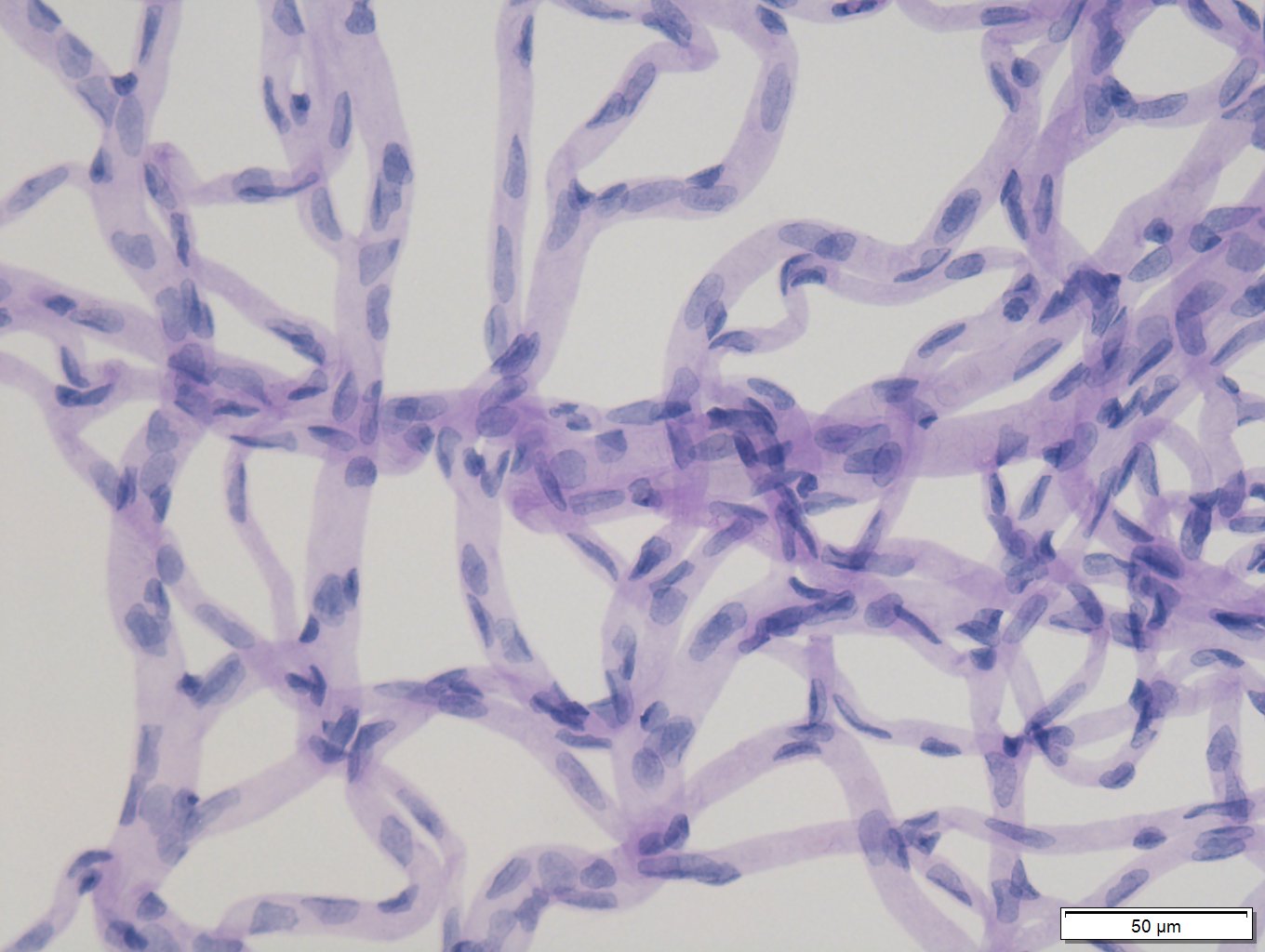

Supplement: S1 File — (ZIP) [file pone.0312791.s001.zip › Fig 2/Fig2 PAS/WT2.jpg]

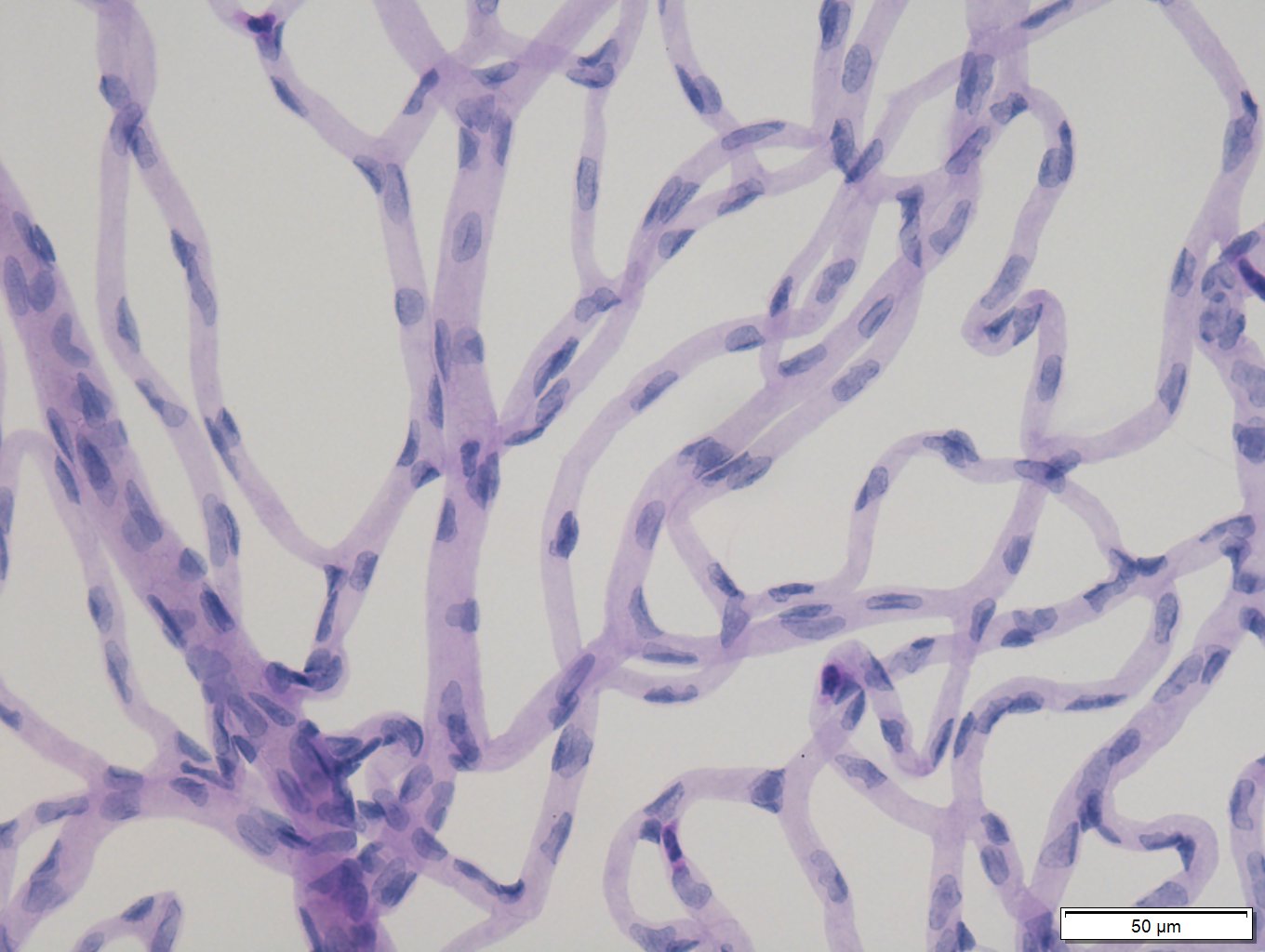

Supplement: S1 File — (ZIP) [file pone.0312791.s001.zip › Fig 2/Fig2 PAS/WT3.jpg]

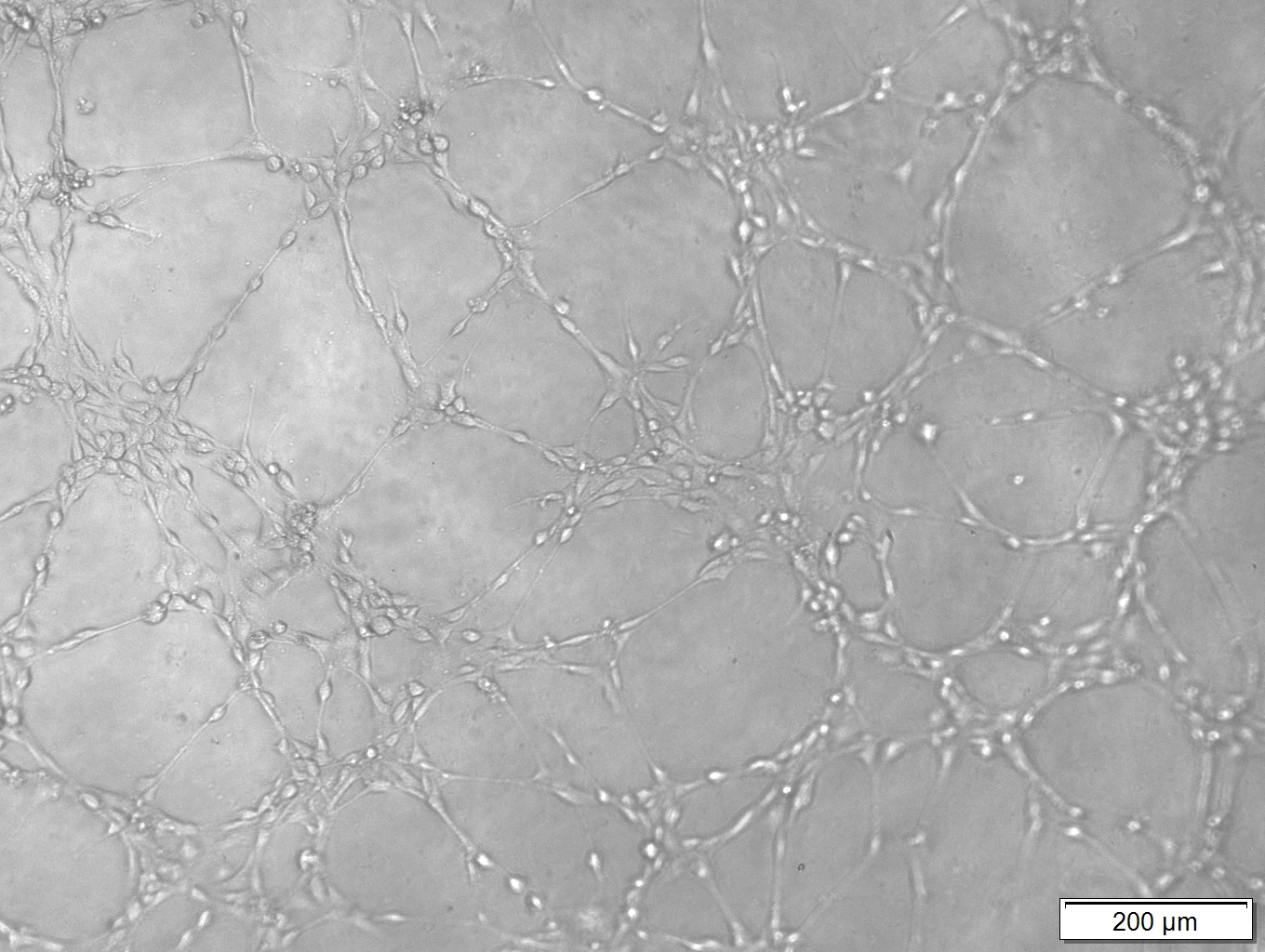

Supplement: S2 File — (ZIP) [file pone.0312791.s002.zip › Fig 7/Fig7 node and junction/HG1.jpg]

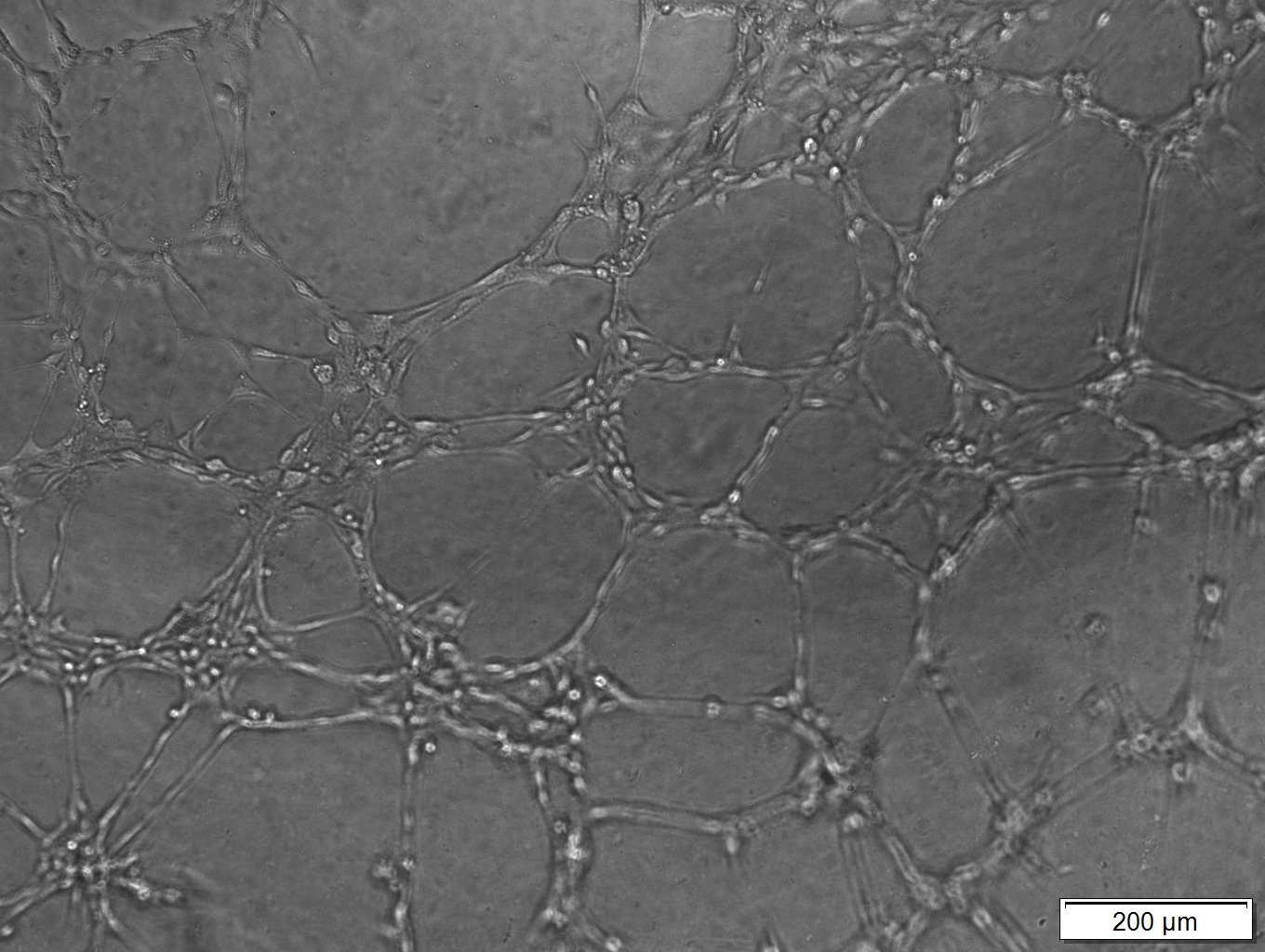

Supplement: S2 File — (ZIP) [file pone.0312791.s002.zip › Fig 7/Fig7 node and junction/HG2.jpg]

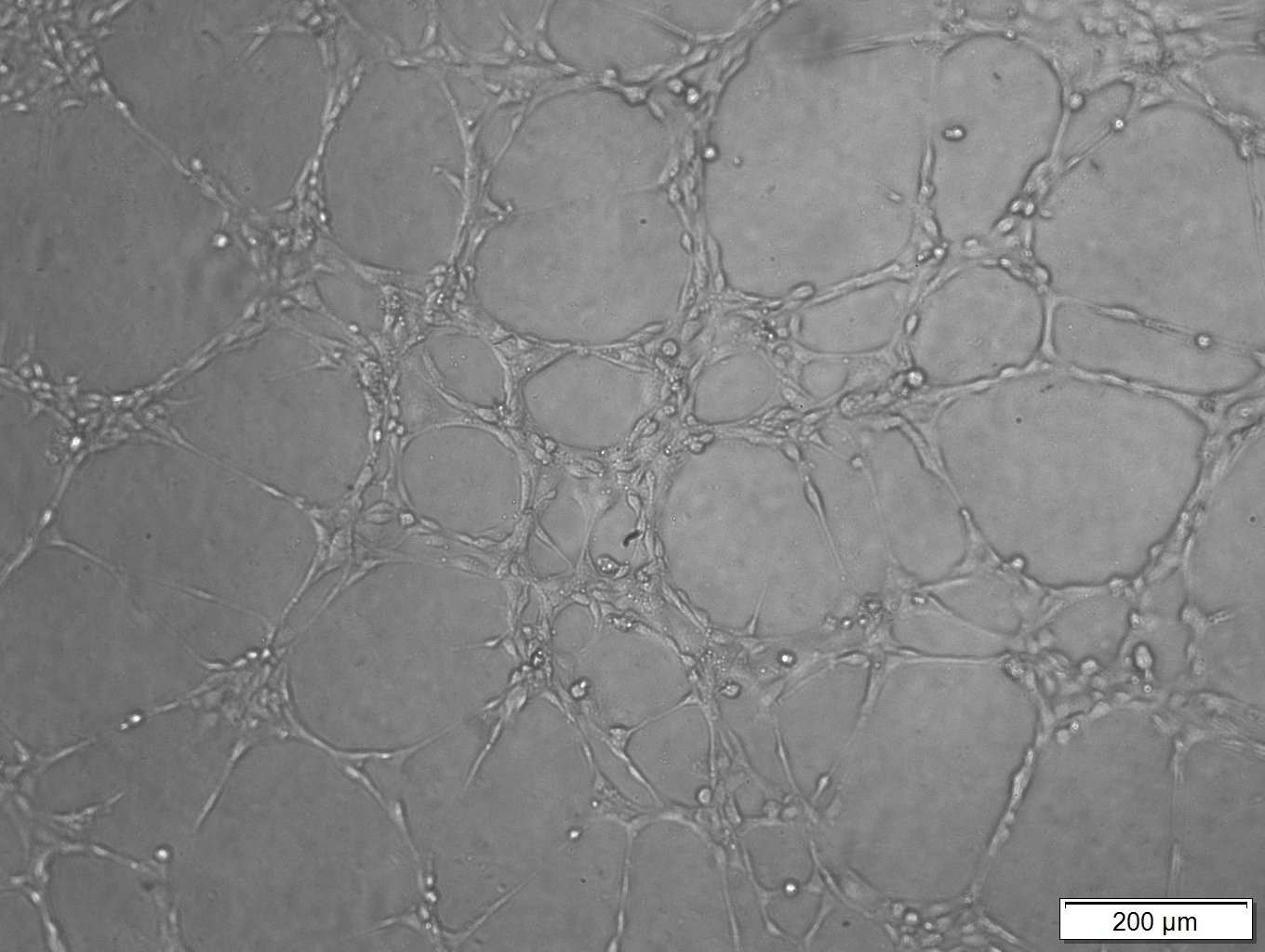

Supplement: S2 File — (ZIP) [file pone.0312791.s002.zip › Fig 7/Fig7 node and junction/HG3.jpg]

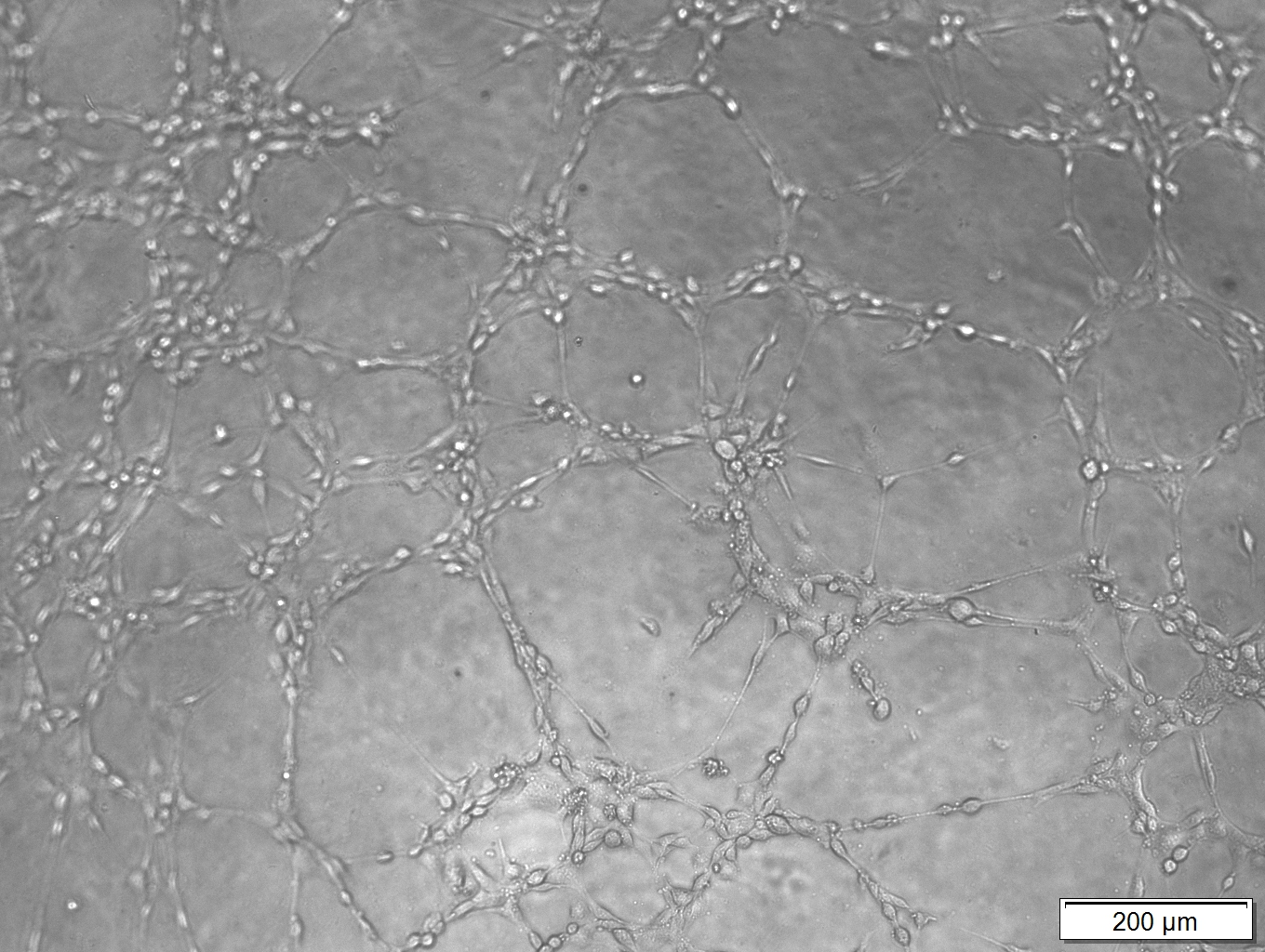

Supplement: S2 File — (ZIP) [file pone.0312791.s002.zip › Fig 7/Fig7 node and junction/NC1.jpg]

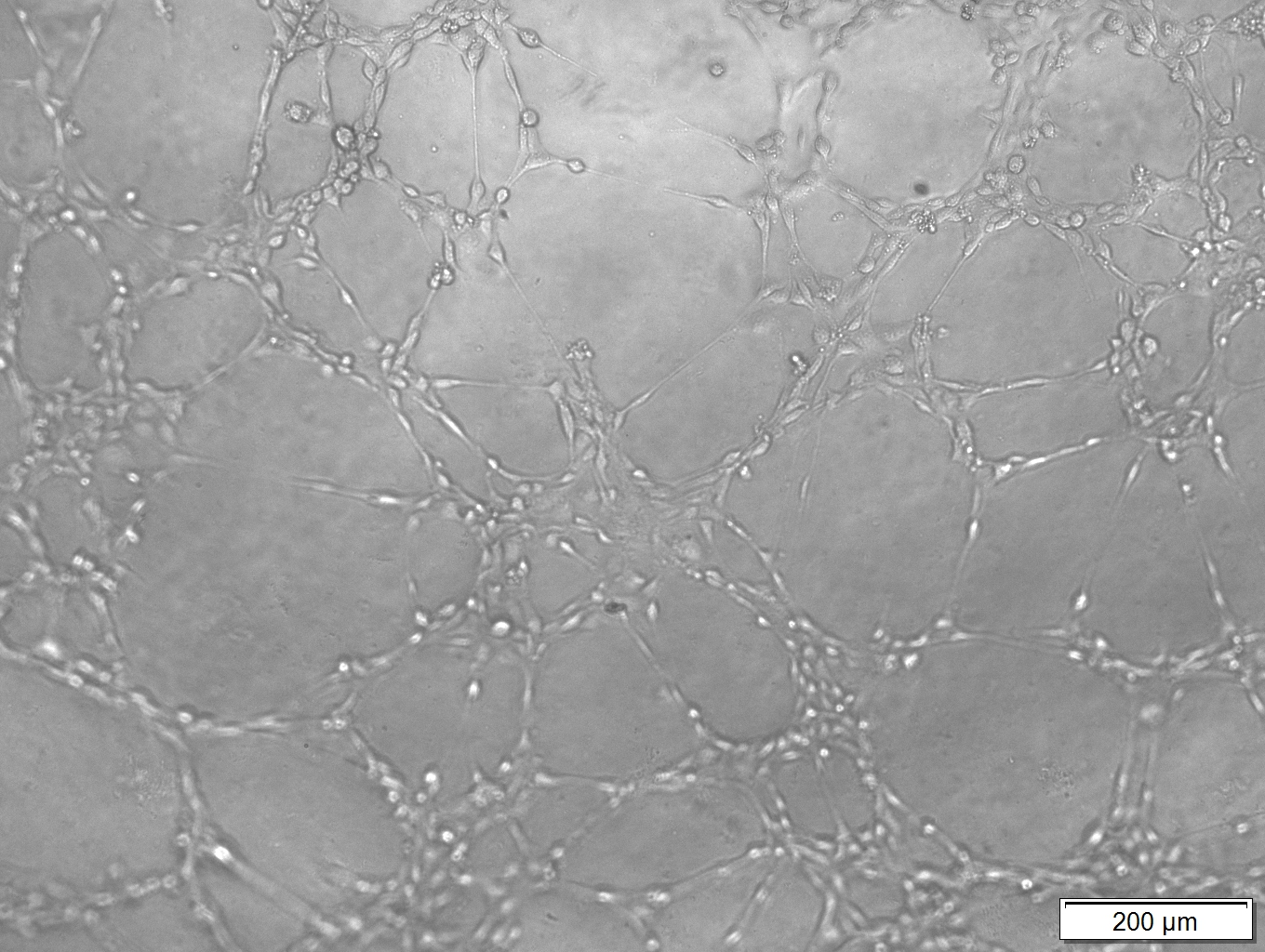

Supplement: S2 File — (ZIP) [file pone.0312791.s002.zip › Fig 7/Fig7 node and junction/NC2.jpg]

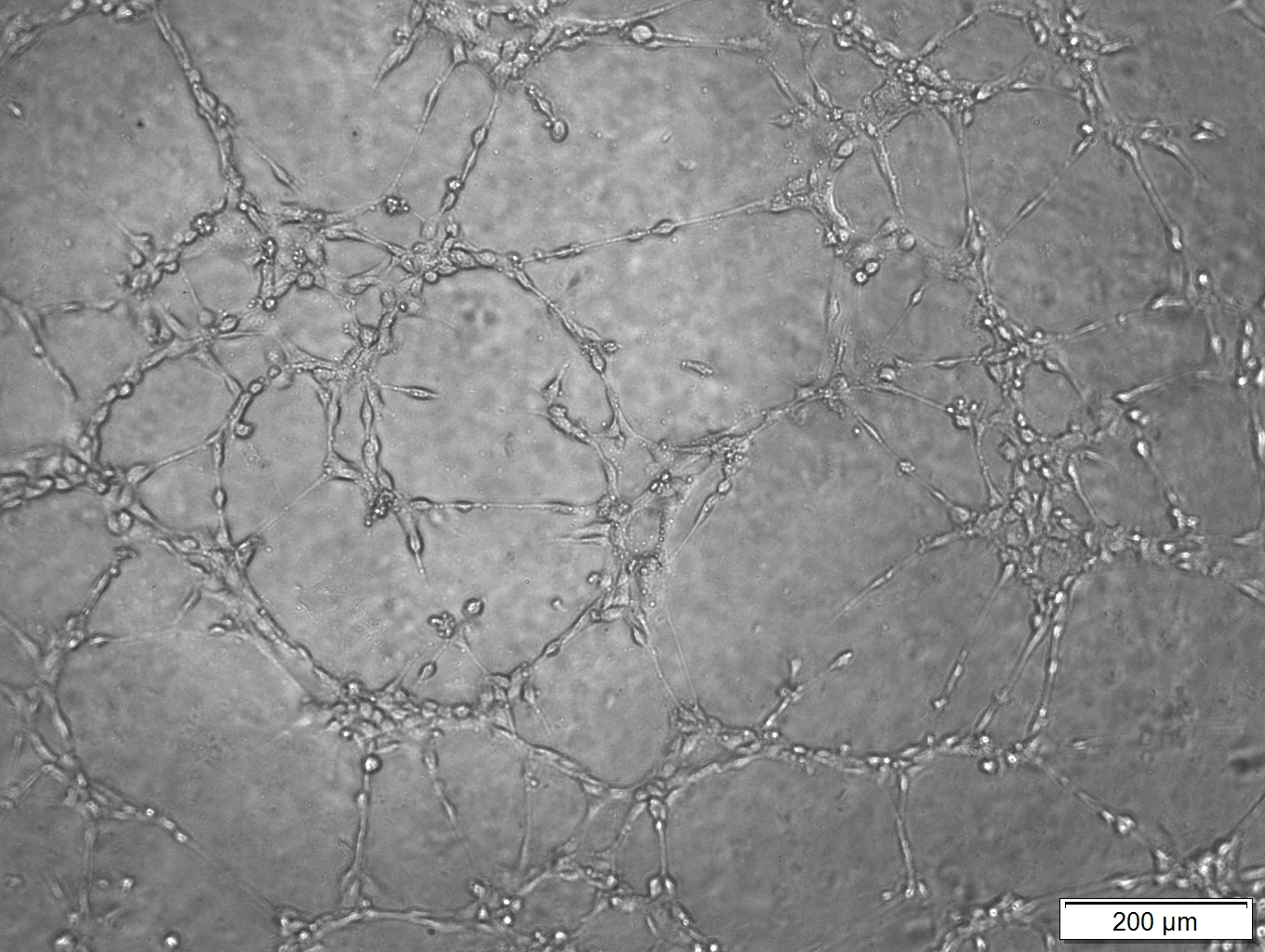

Supplement: S2 File — (ZIP) [file pone.0312791.s002.zip › Fig 7/Fig7 node and junction/NC3.jpg]

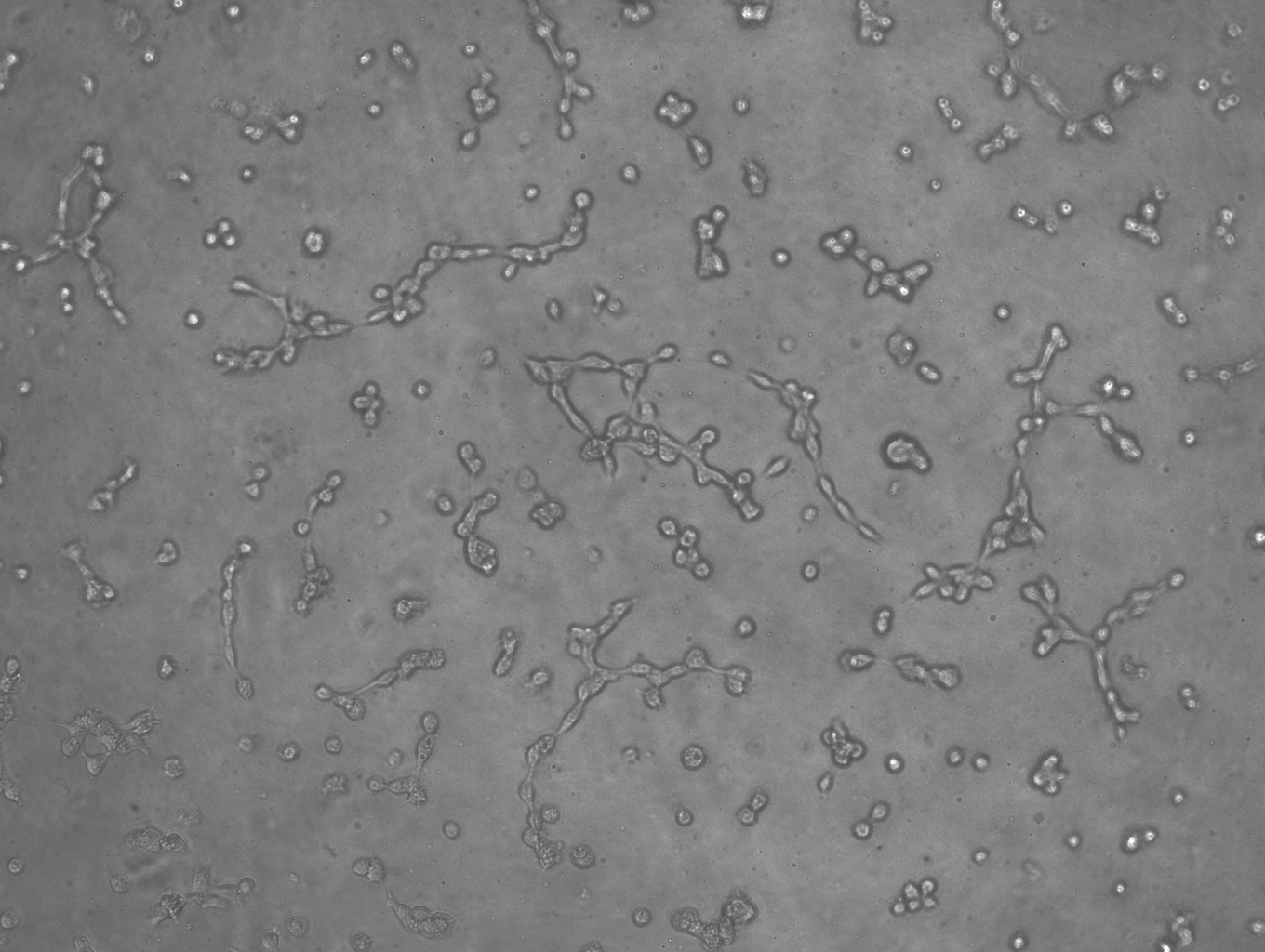

Supplement: S2 File — (ZIP) [file pone.0312791.s002.zip › Fig 7/Fig7 node and junction/NG1.tif]

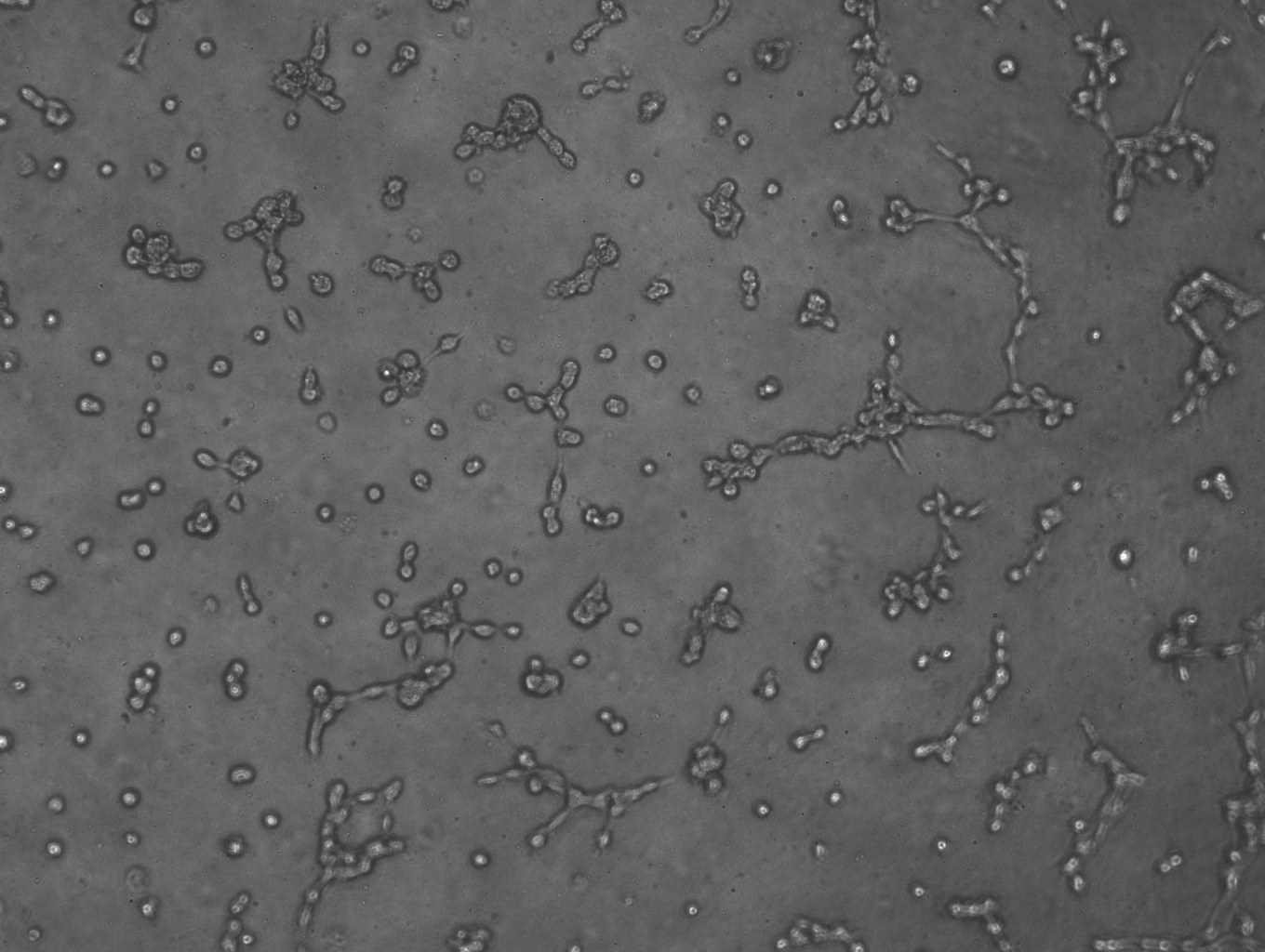

Supplement: S2 File — (ZIP) [file pone.0312791.s002.zip › Fig 7/Fig7 node and junction/NG2.tif]

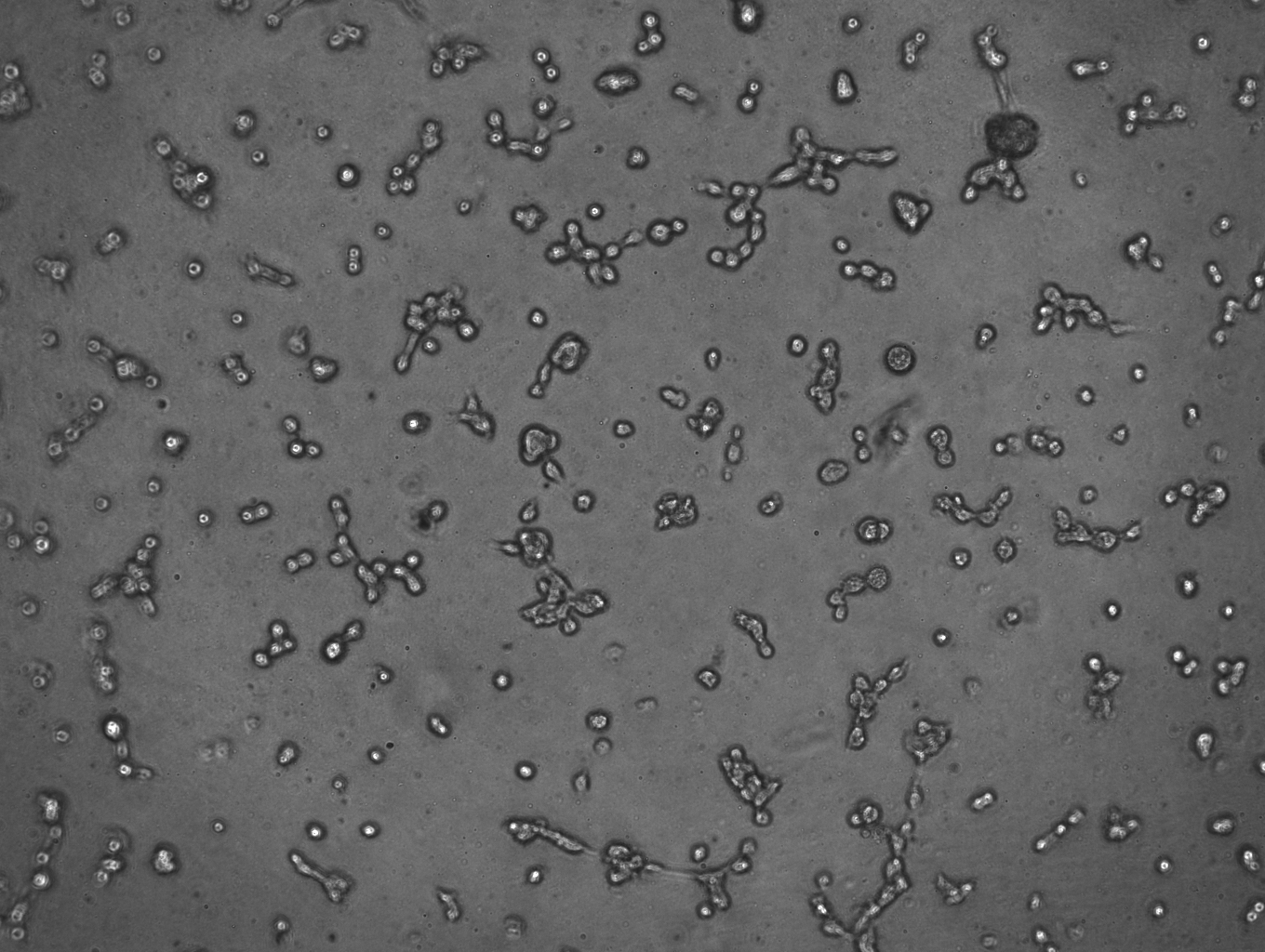

Supplement: S2 File — (ZIP) [file pone.0312791.s002.zip › Fig 7/Fig7 node and junction/NG3.tif]

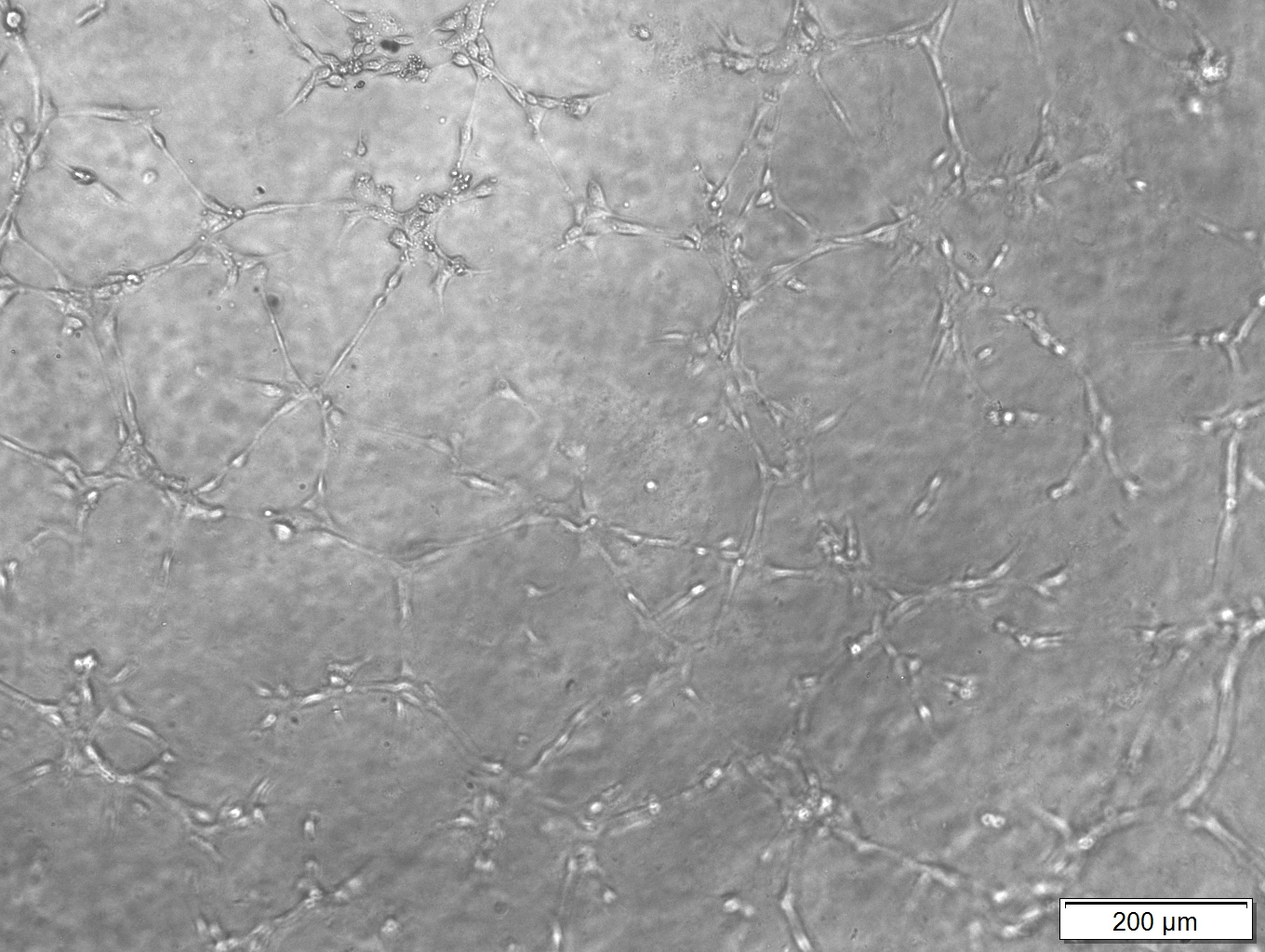

Supplement: S2 File — (ZIP) [file pone.0312791.s002.zip › Fig 7/Fig7 node and junction/RP11-1.jpg]

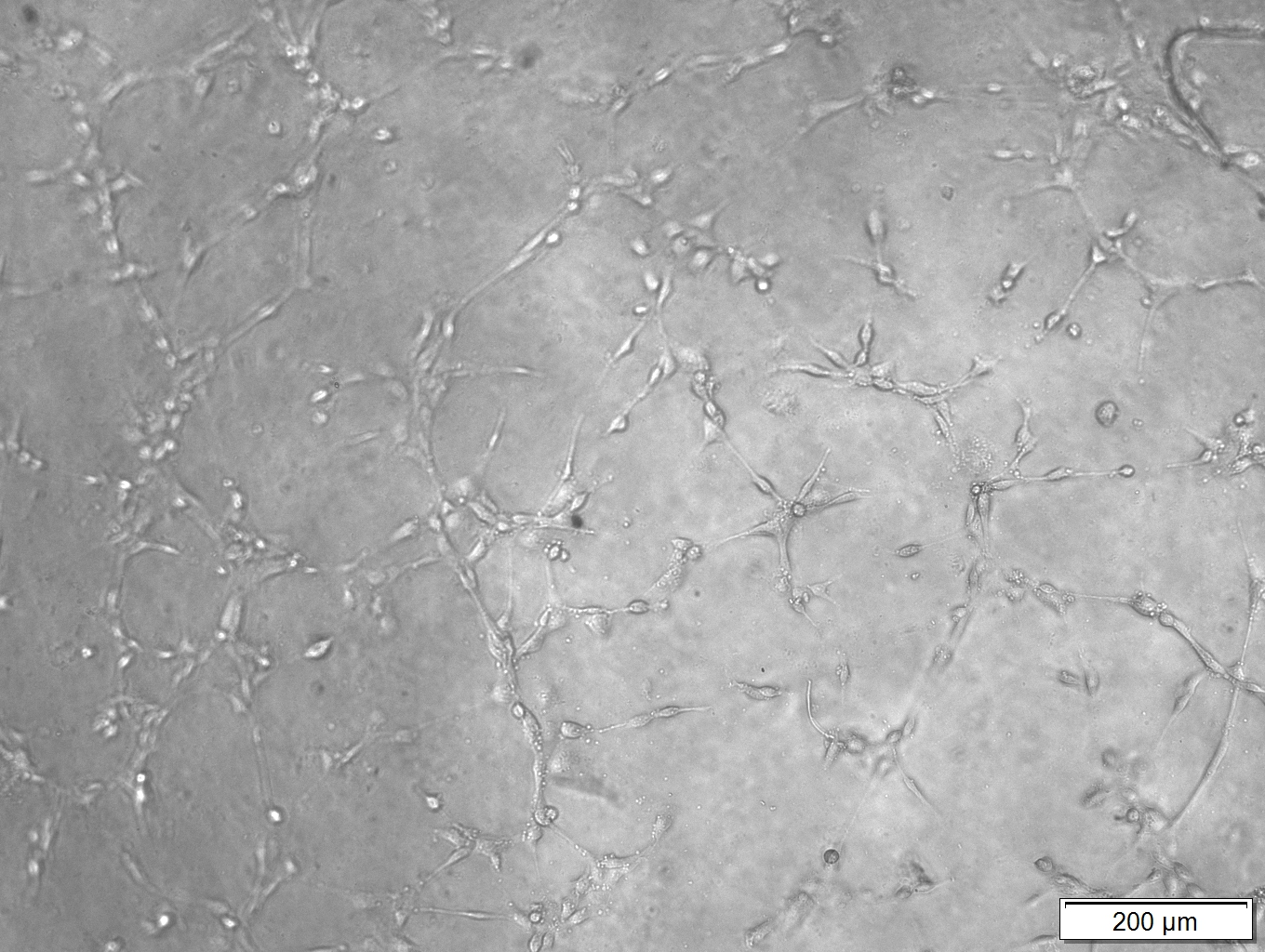

Supplement: S2 File — (ZIP) [file pone.0312791.s002.zip › Fig 7/Fig7 node and junction/RP11-2.jpg]

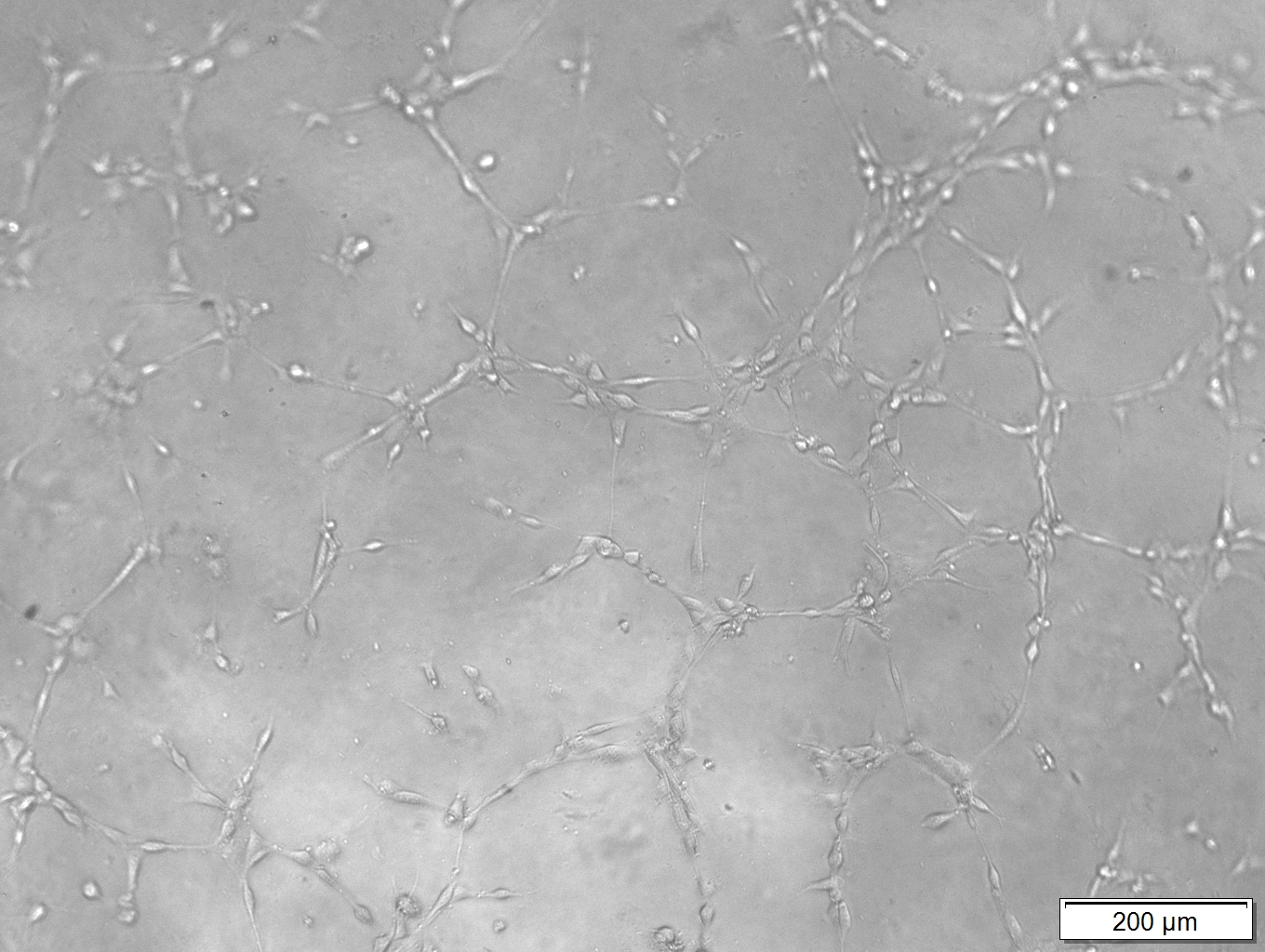

Supplement: S2 File — (ZIP) [file pone.0312791.s002.zip › Fig 7/Fig7 node and junction/RP11-3.jpg]

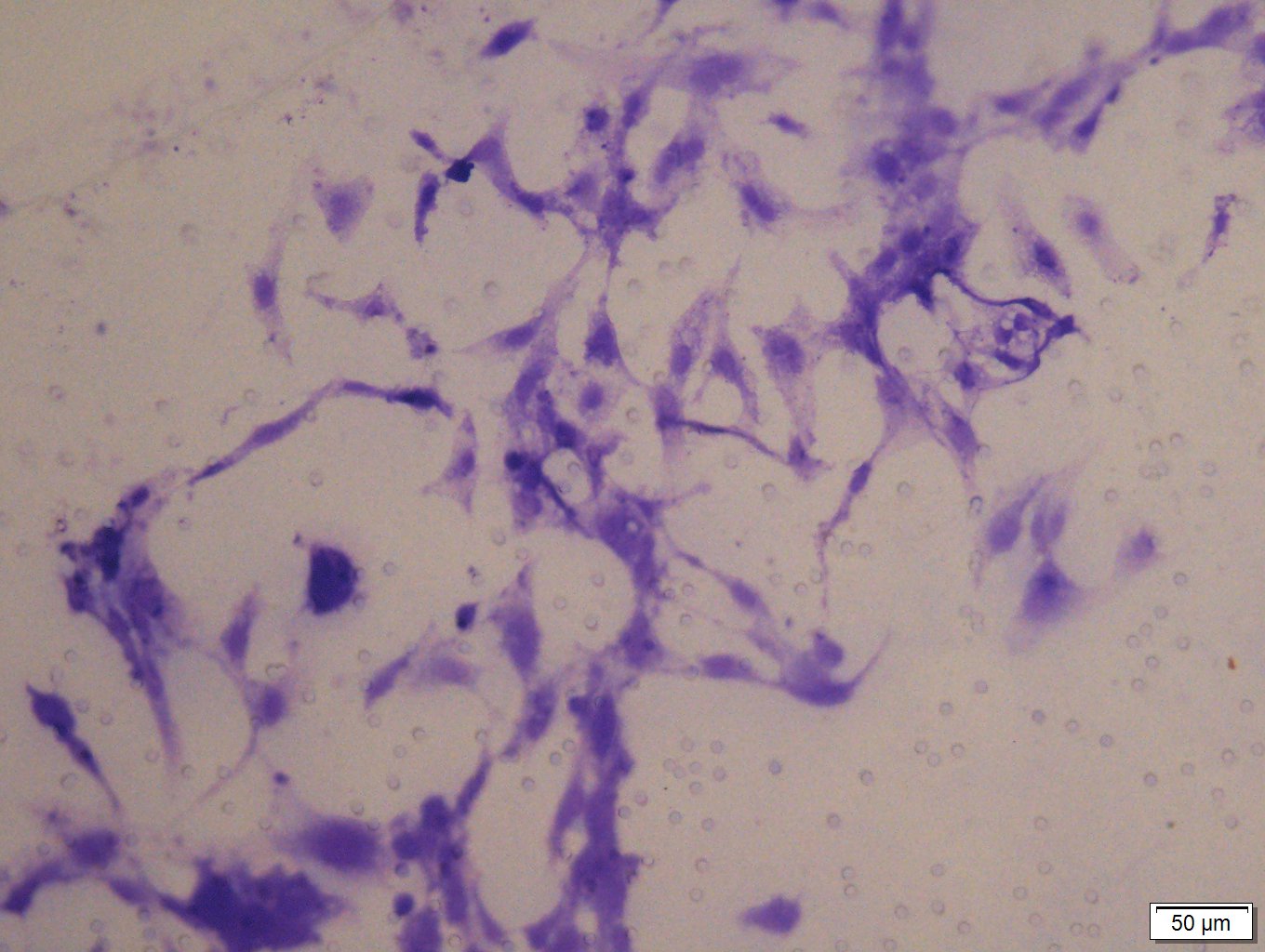

Supplement: S2 File — (ZIP) [file pone.0312791.s002.zip › Fig 7/Fig7 transwell/HG1.jpg]

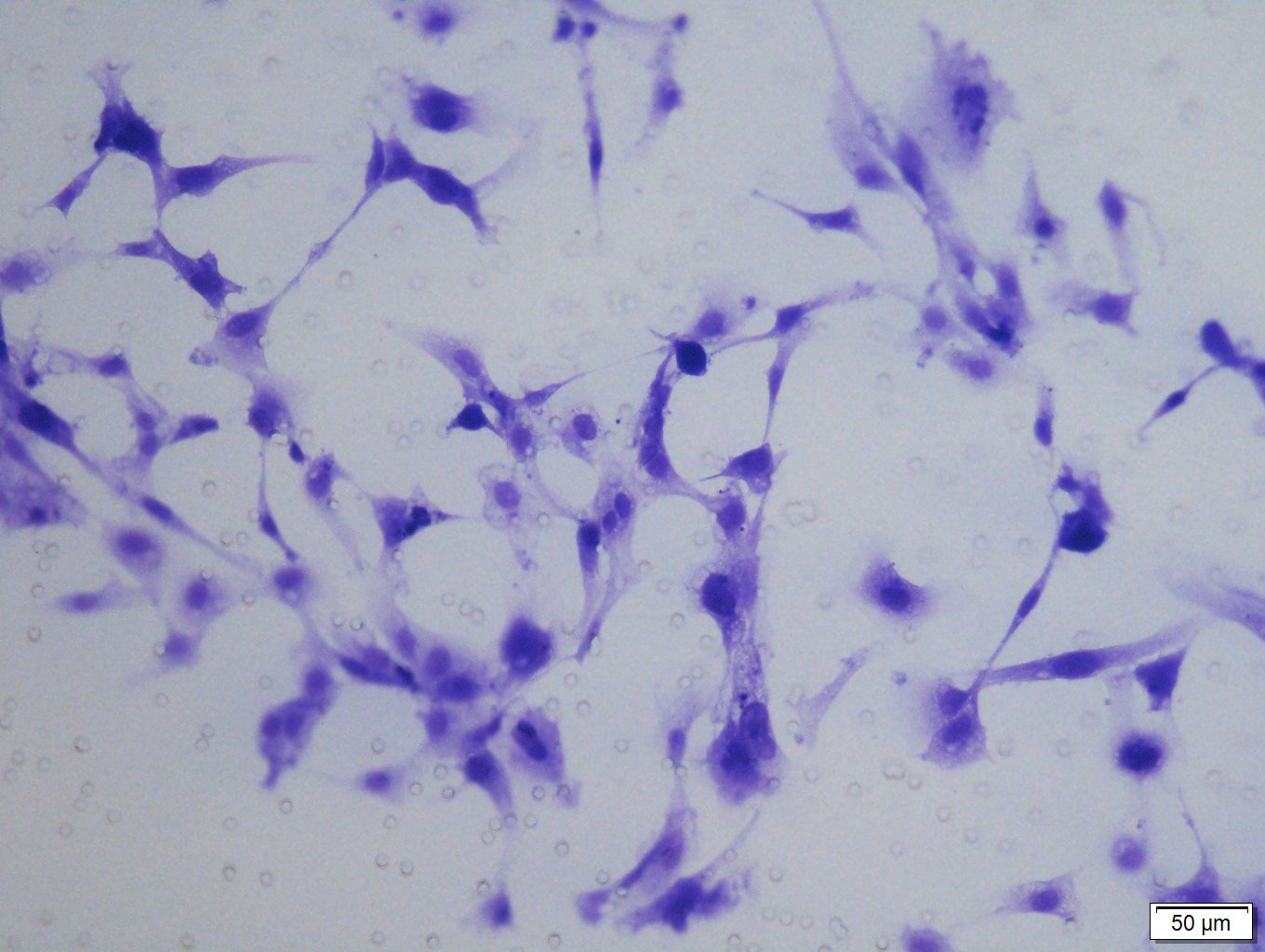

Supplement: S2 File — (ZIP) [file pone.0312791.s002.zip › Fig 7/Fig7 transwell/HG2.jpg]

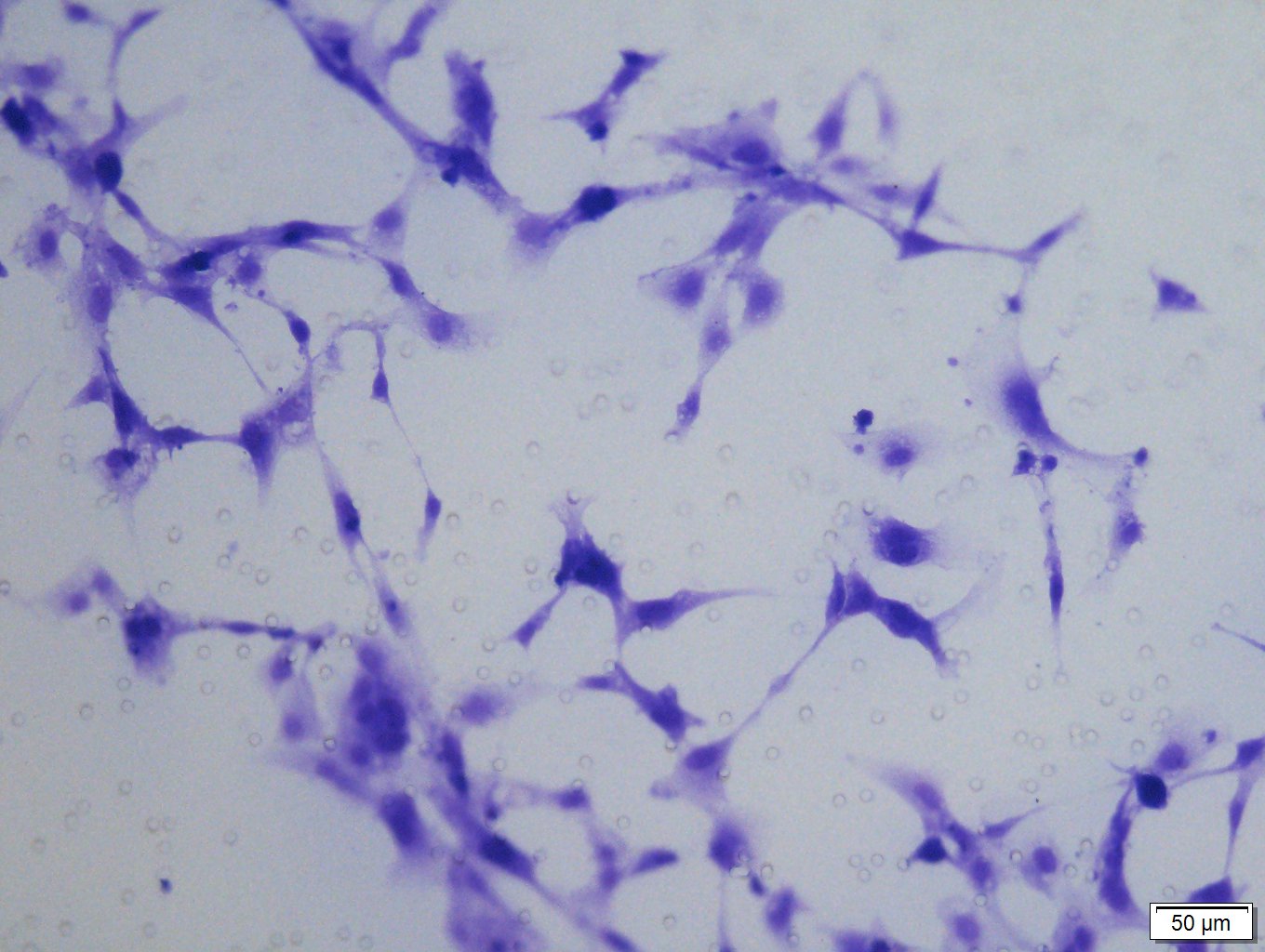

Supplement: S2 File — (ZIP) [file pone.0312791.s002.zip › Fig 7/Fig7 transwell/HG3.jpg]

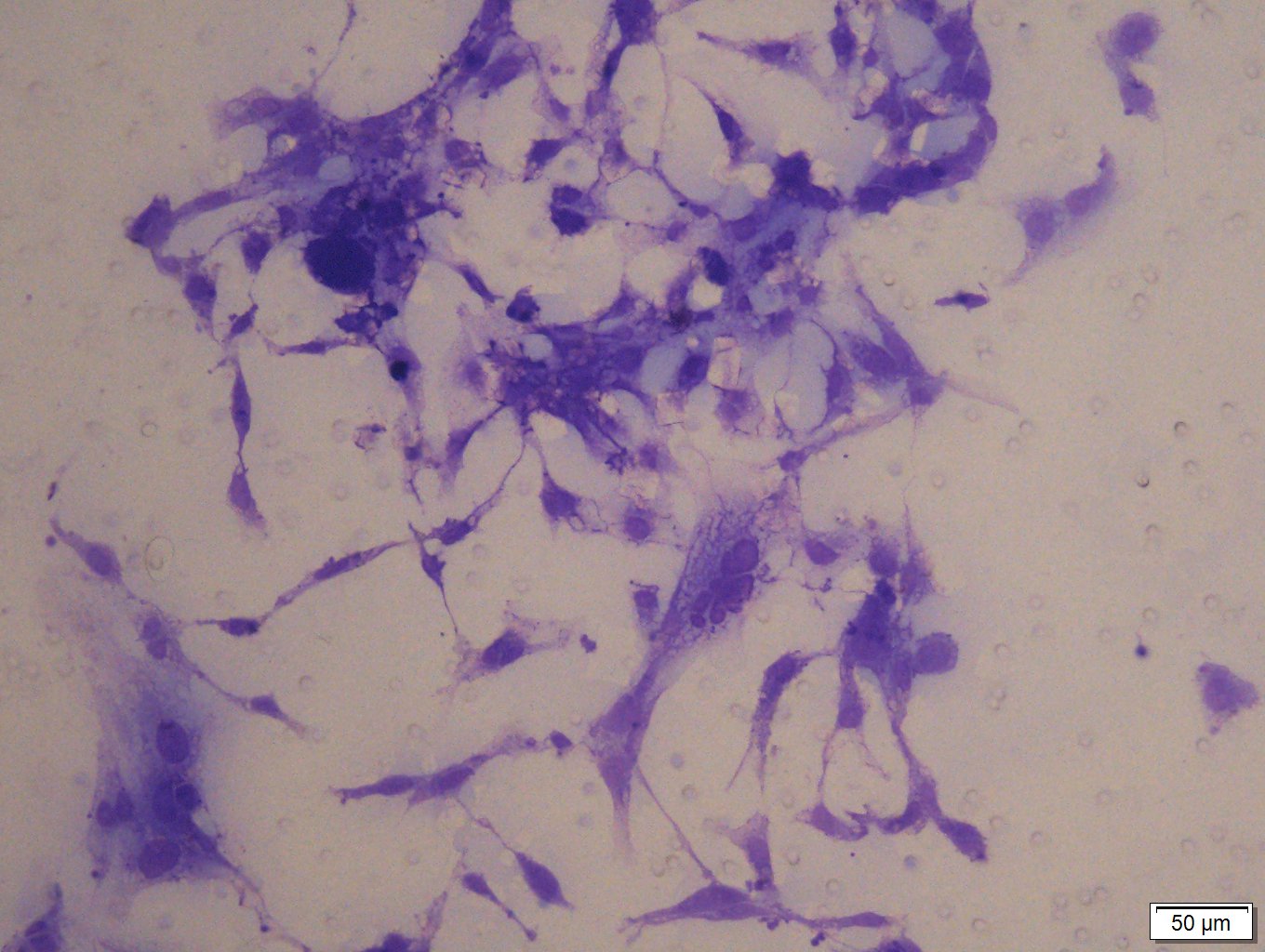

Supplement: S2 File — (ZIP) [file pone.0312791.s002.zip › Fig 7/Fig7 transwell/NC1.jpg]

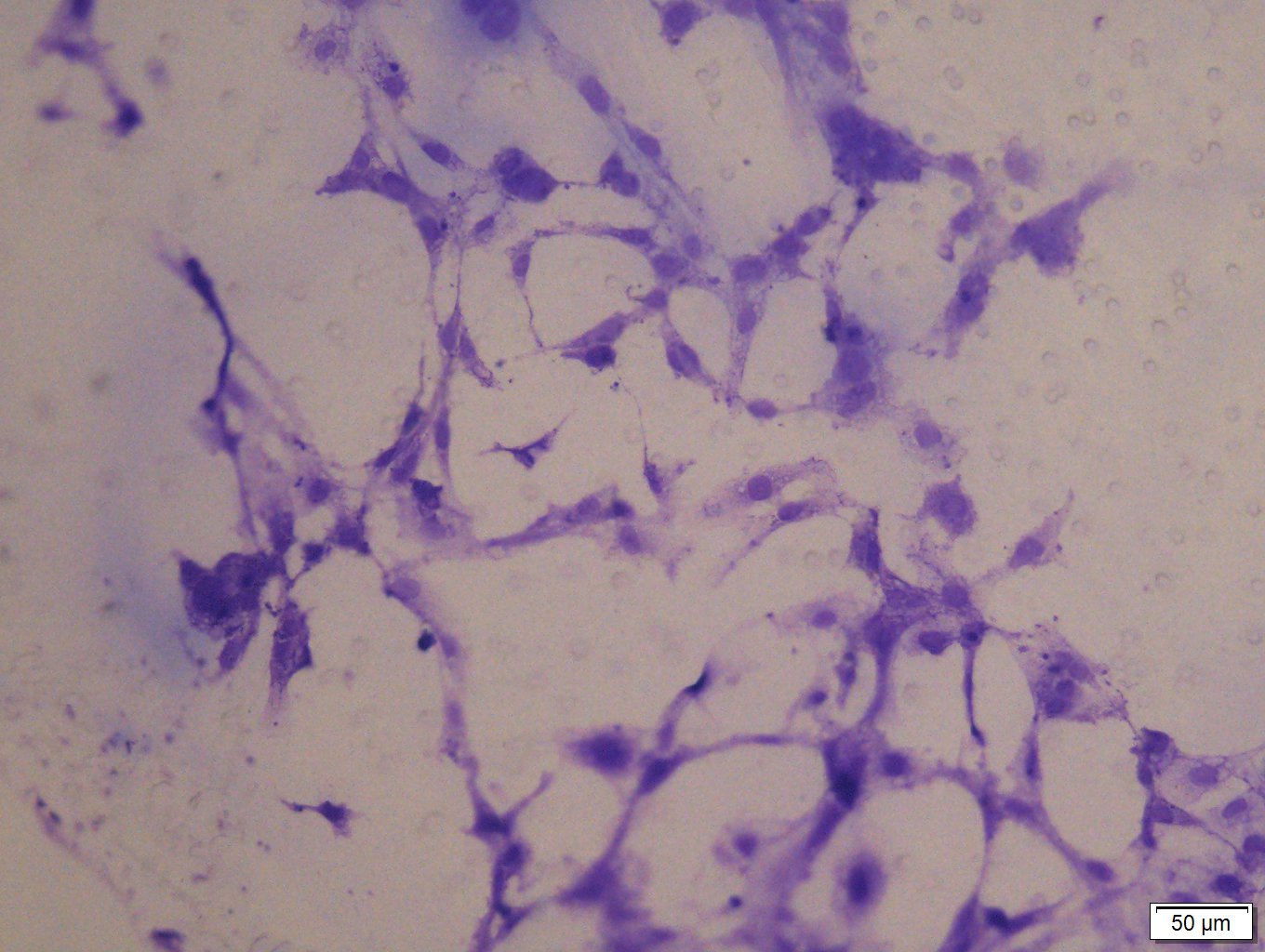

Supplement: S2 File — (ZIP) [file pone.0312791.s002.zip › Fig 7/Fig7 transwell/NC2.jpg]

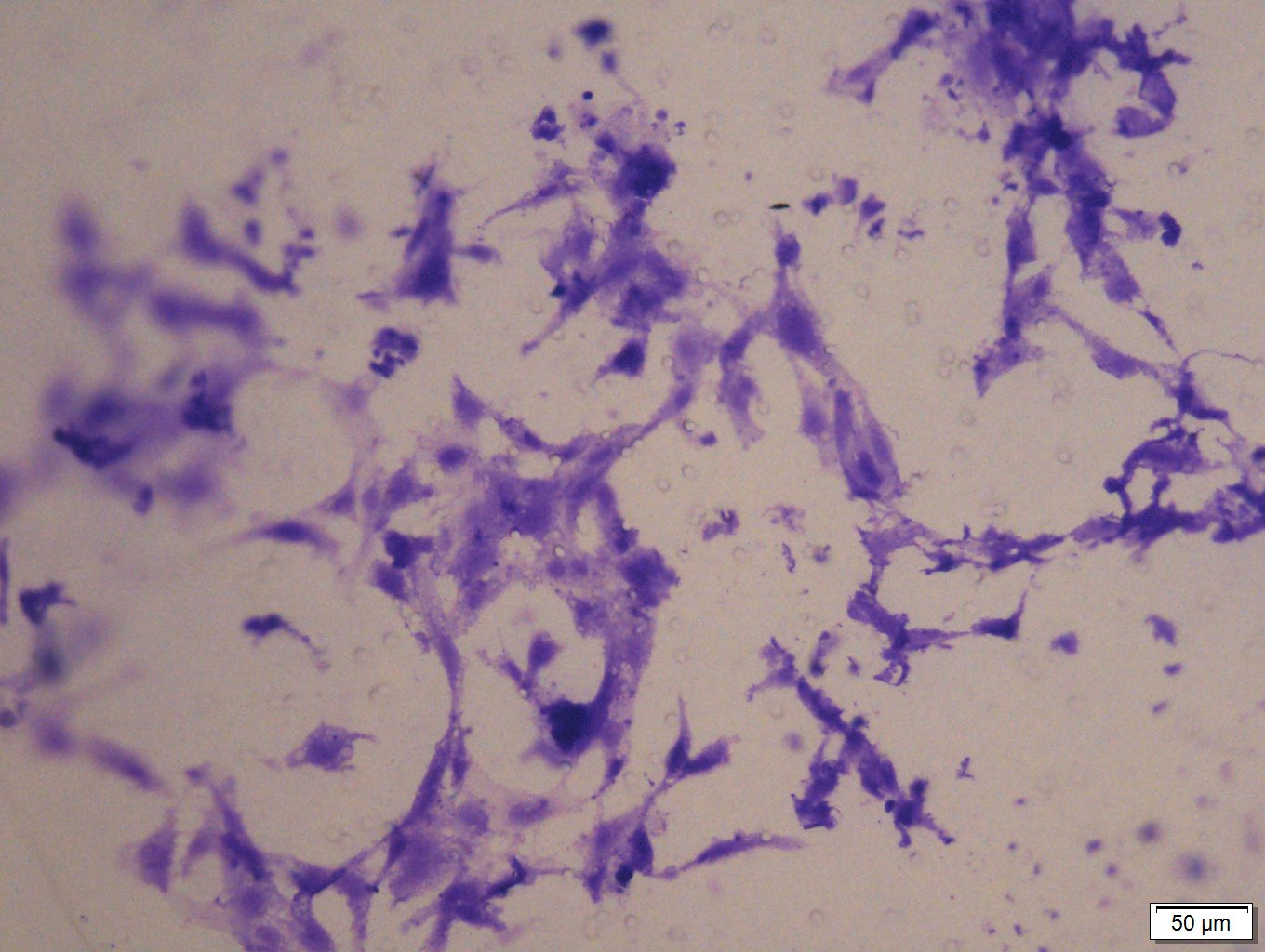

Supplement: S2 File — (ZIP) [file pone.0312791.s002.zip › Fig 7/Fig7 transwell/NC3.jpg]

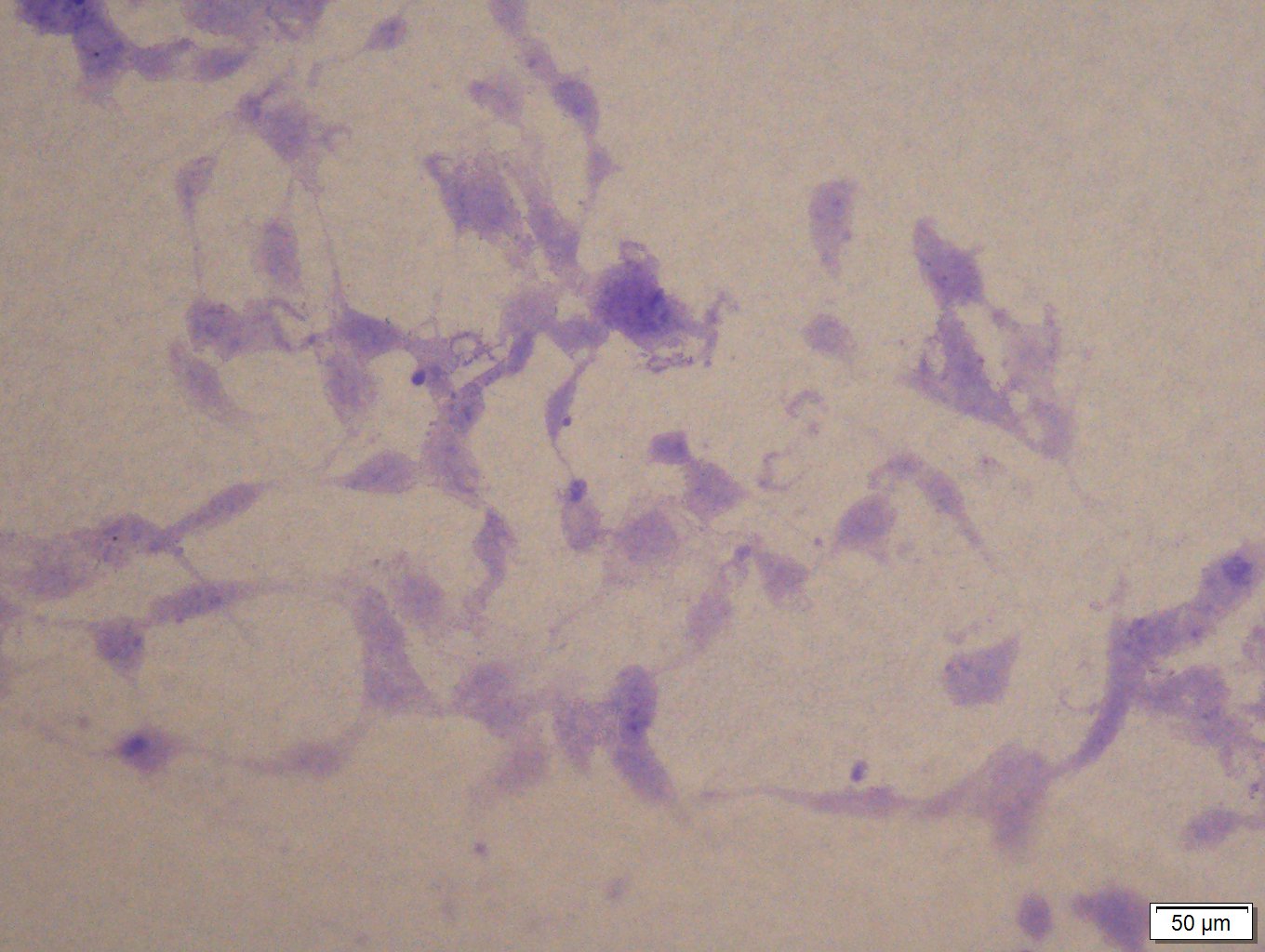

Supplement: S2 File — (ZIP) [file pone.0312791.s002.zip › Fig 7/Fig7 transwell/NG1.jpg]

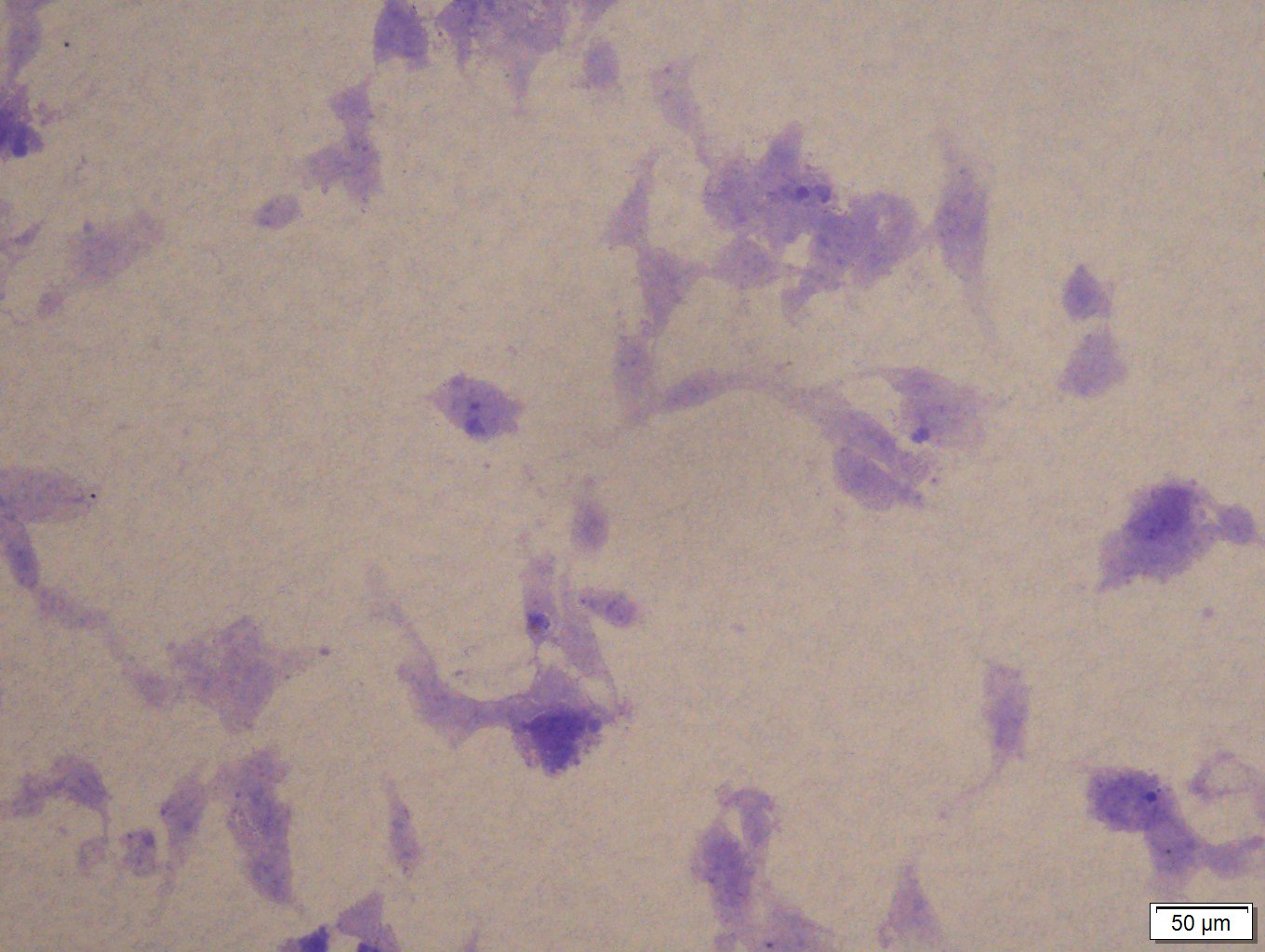

Supplement: S2 File — (ZIP) [file pone.0312791.s002.zip › Fig 7/Fig7 transwell/NG2.jpg]

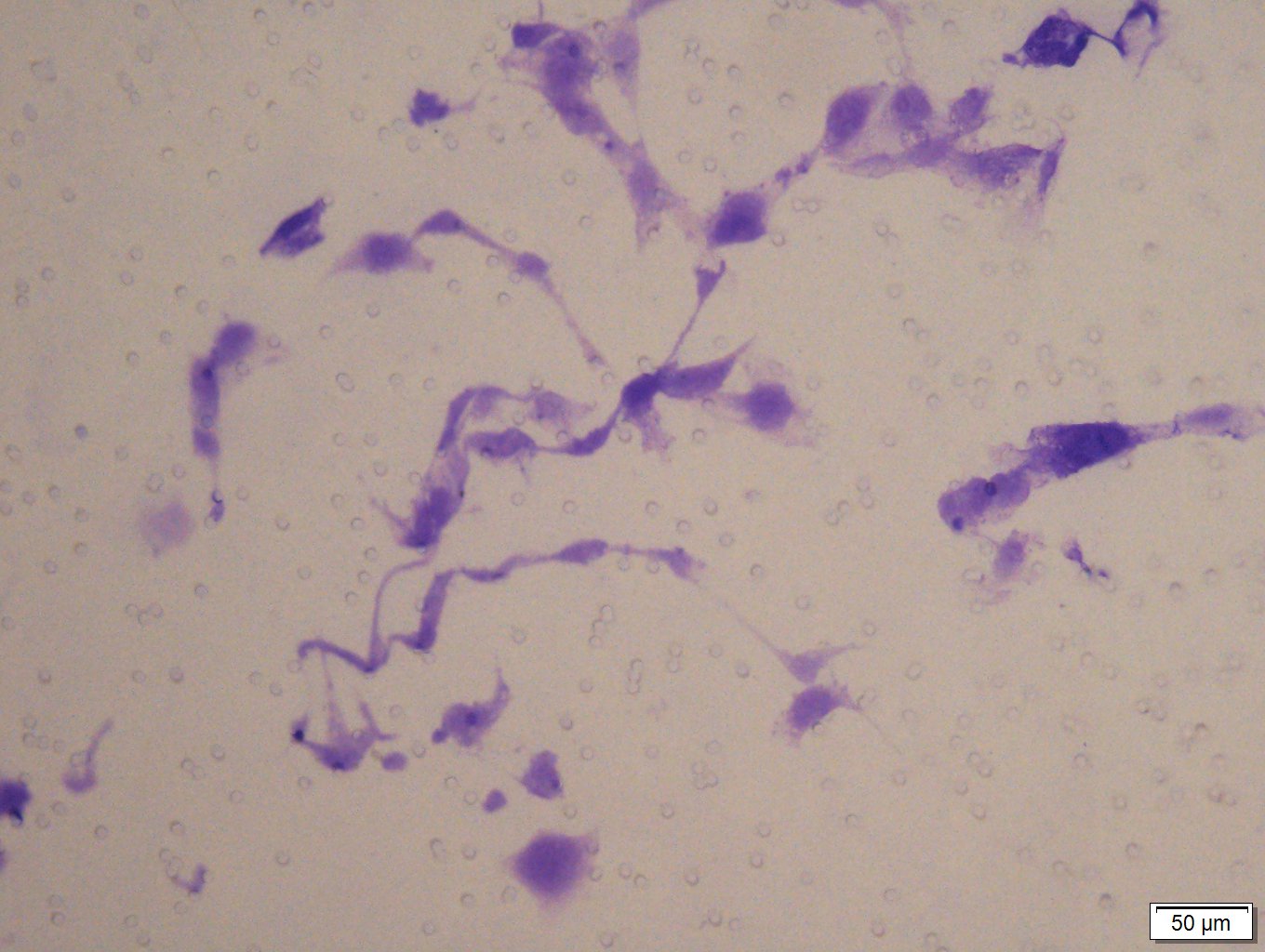

Supplement: S2 File — (ZIP) [file pone.0312791.s002.zip › Fig 7/Fig7 transwell/NG3.jpg]

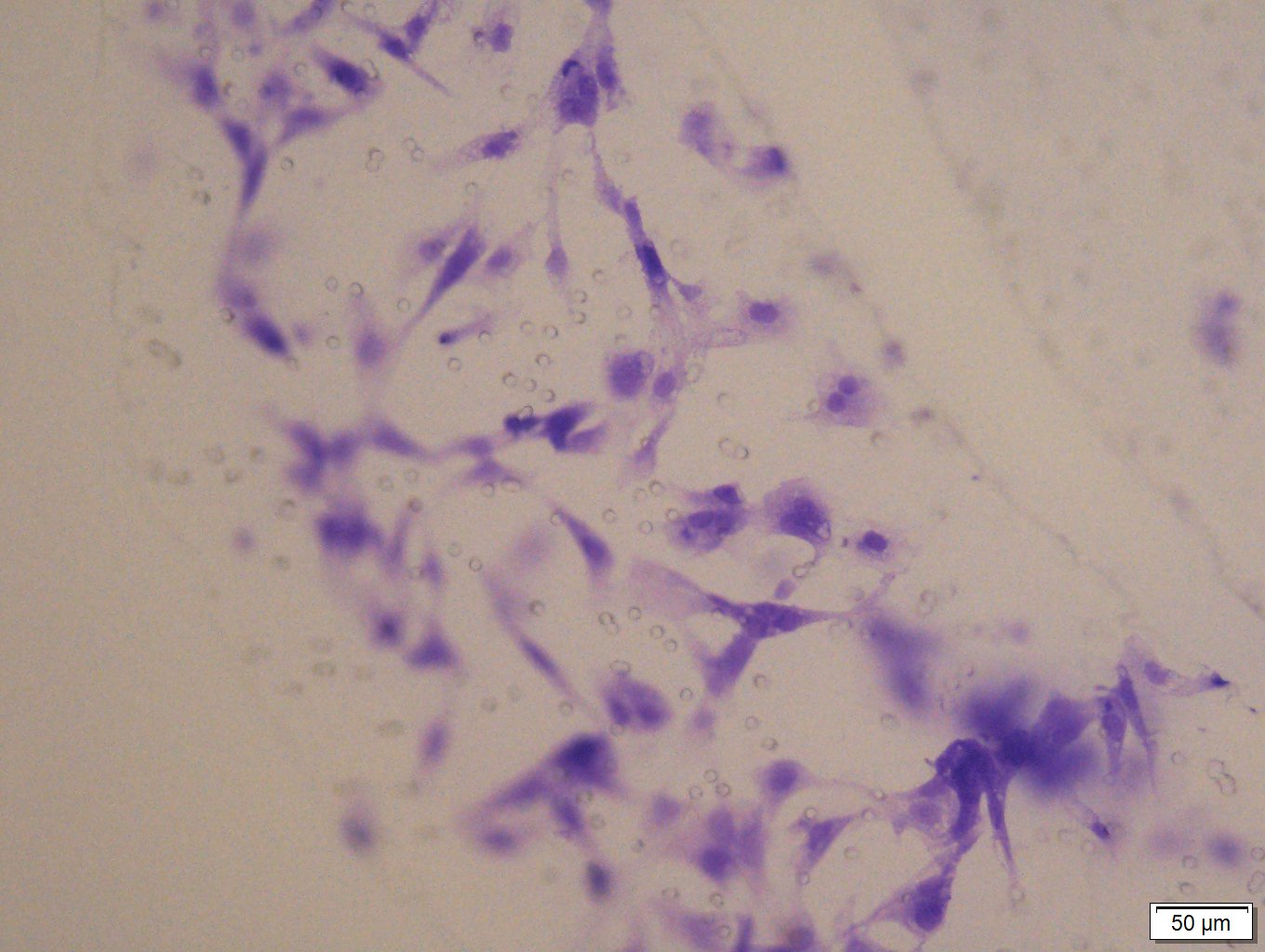

Supplement: S2 File — (ZIP) [file pone.0312791.s002.zip › Fig 7/Fig7 transwell/RP11-1.jpg]

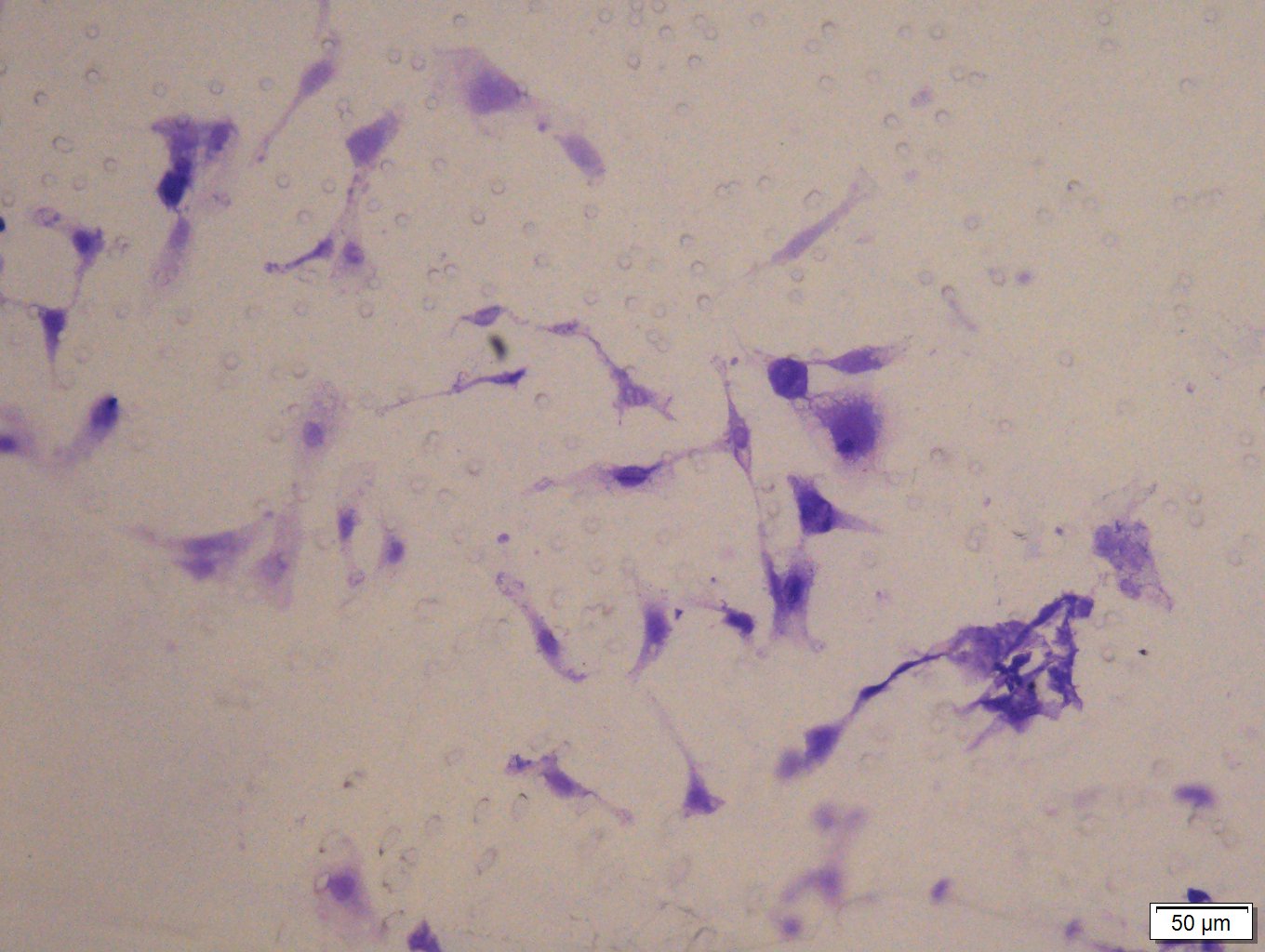

Supplement: S2 File — (ZIP) [file pone.0312791.s002.zip › Fig 7/Fig7 transwell/RP11-2.jpg]

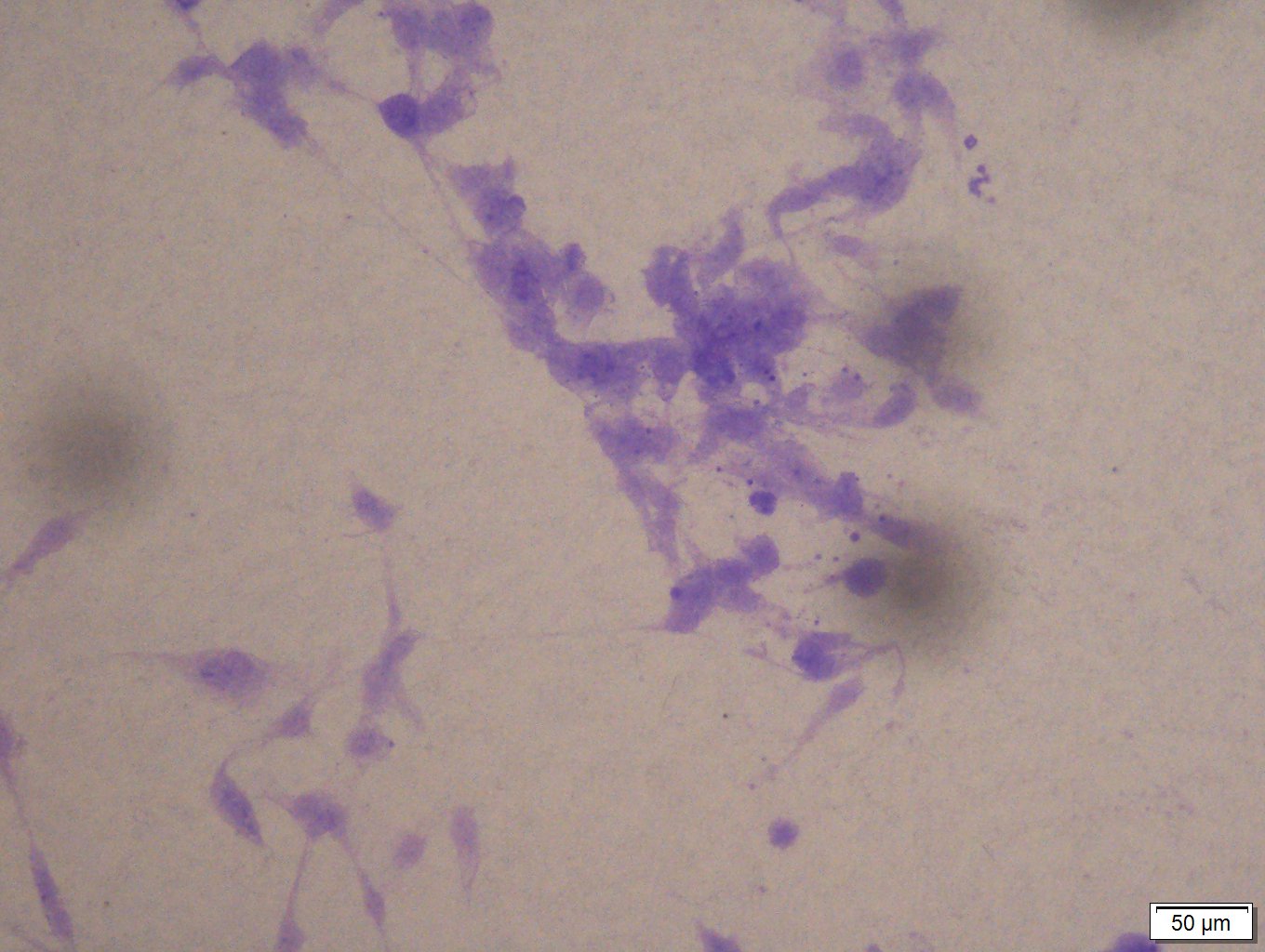

Supplement: S2 File — (ZIP) [file pone.0312791.s002.zip › Fig 7/Fig7 transwell/RP11-3.jpg]

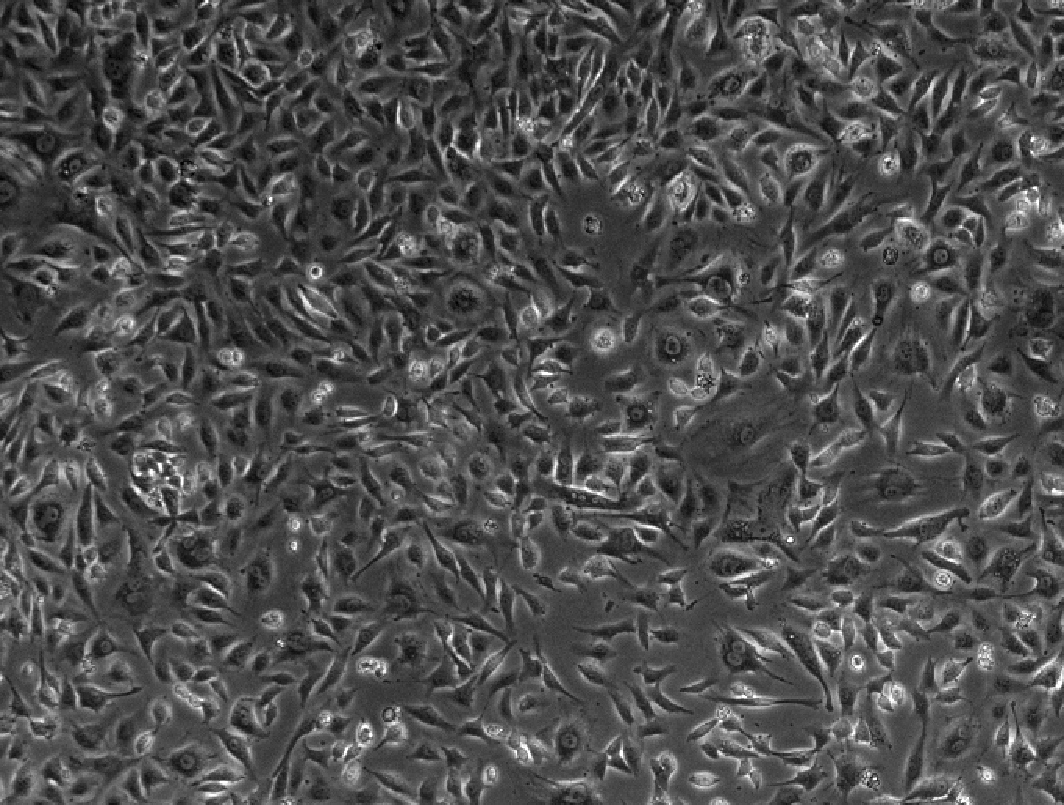

Supplement: S2 File — (ZIP) [file pone.0312791.s002.zip › Fig 5/Fig5 RP11 transfection efficiency/NC1 BF.tif]

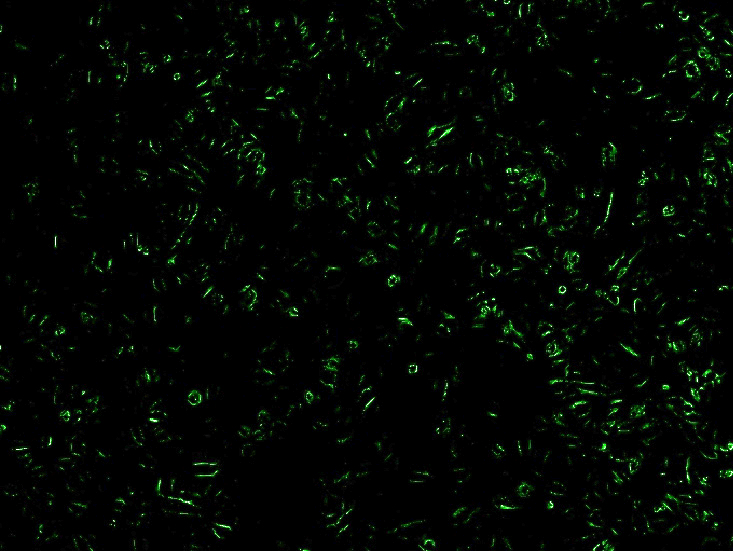

Supplement: S2 File — (ZIP) [file pone.0312791.s002.zip › Fig 5/Fig5 RP11 transfection efficiency/NC1 IF.tif]

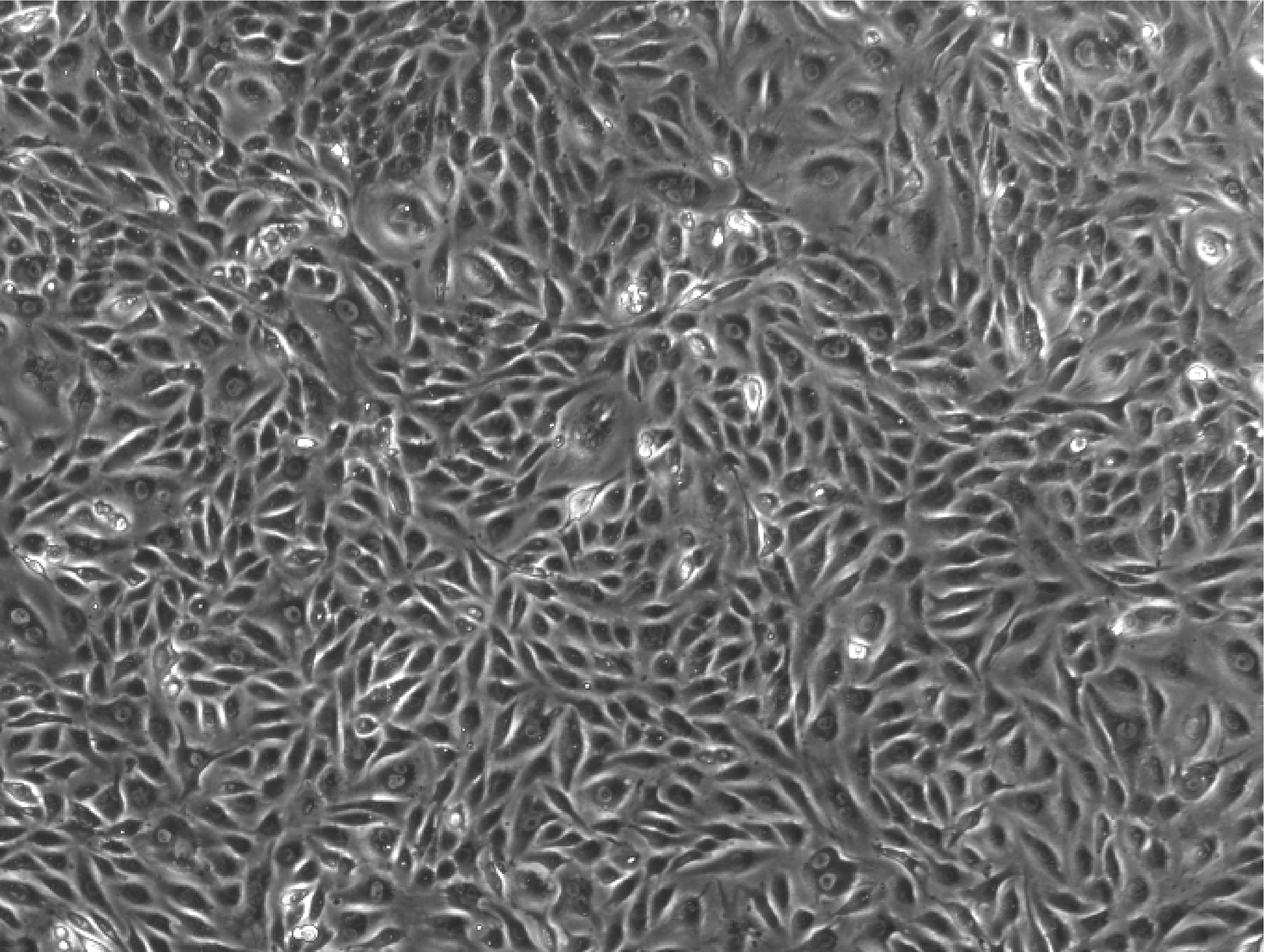

Supplement: S2 File — (ZIP) [file pone.0312791.s002.zip › Fig 5/Fig5 RP11 transfection efficiency/NC2 BF.tif]

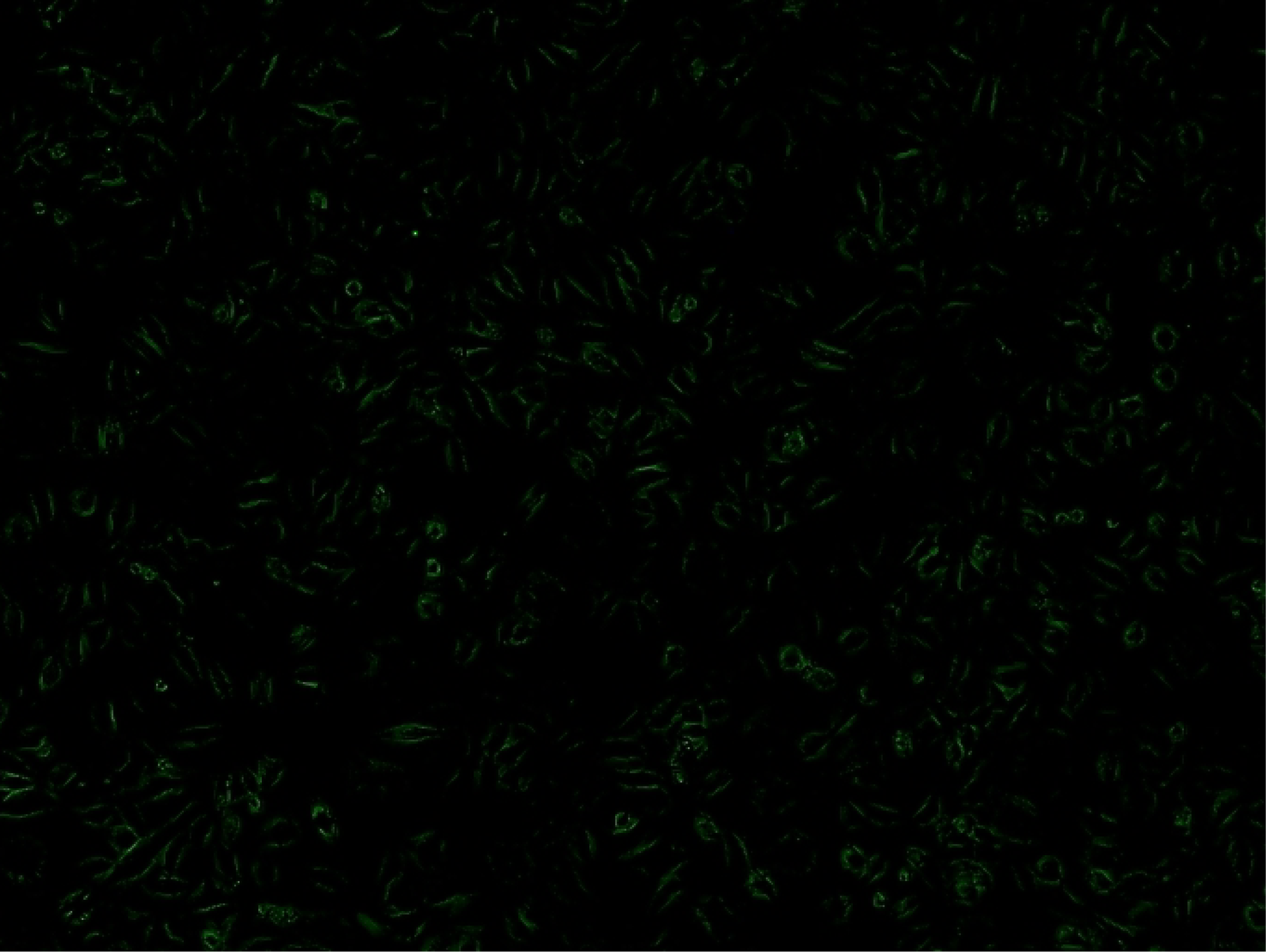

Supplement: S2 File — (ZIP) [file pone.0312791.s002.zip › Fig 5/Fig5 RP11 transfection efficiency/NC2 IF.tif]

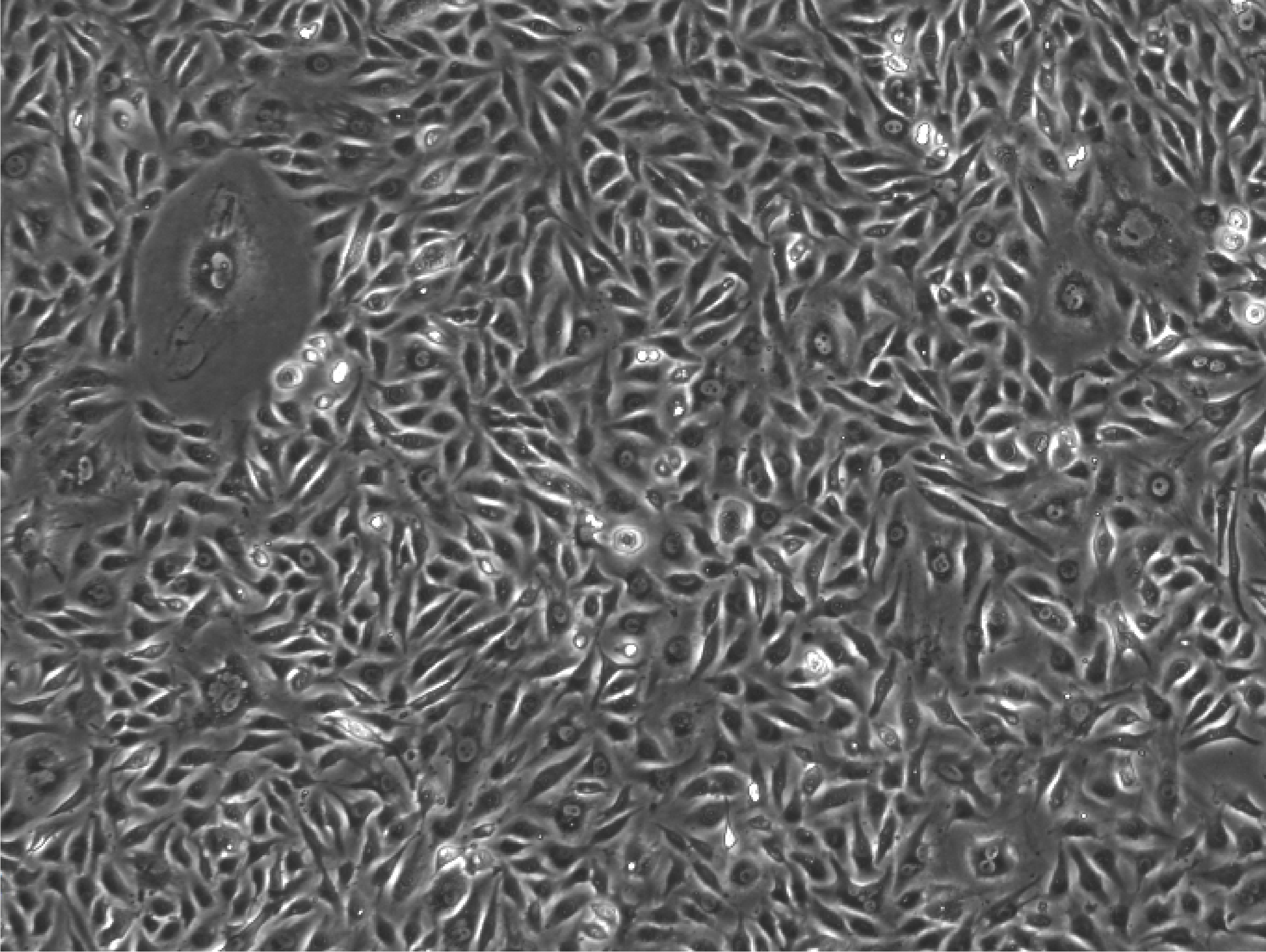

Supplement: S2 File — (ZIP) [file pone.0312791.s002.zip › Fig 5/Fig5 RP11 transfection efficiency/NC3 BF.tif]

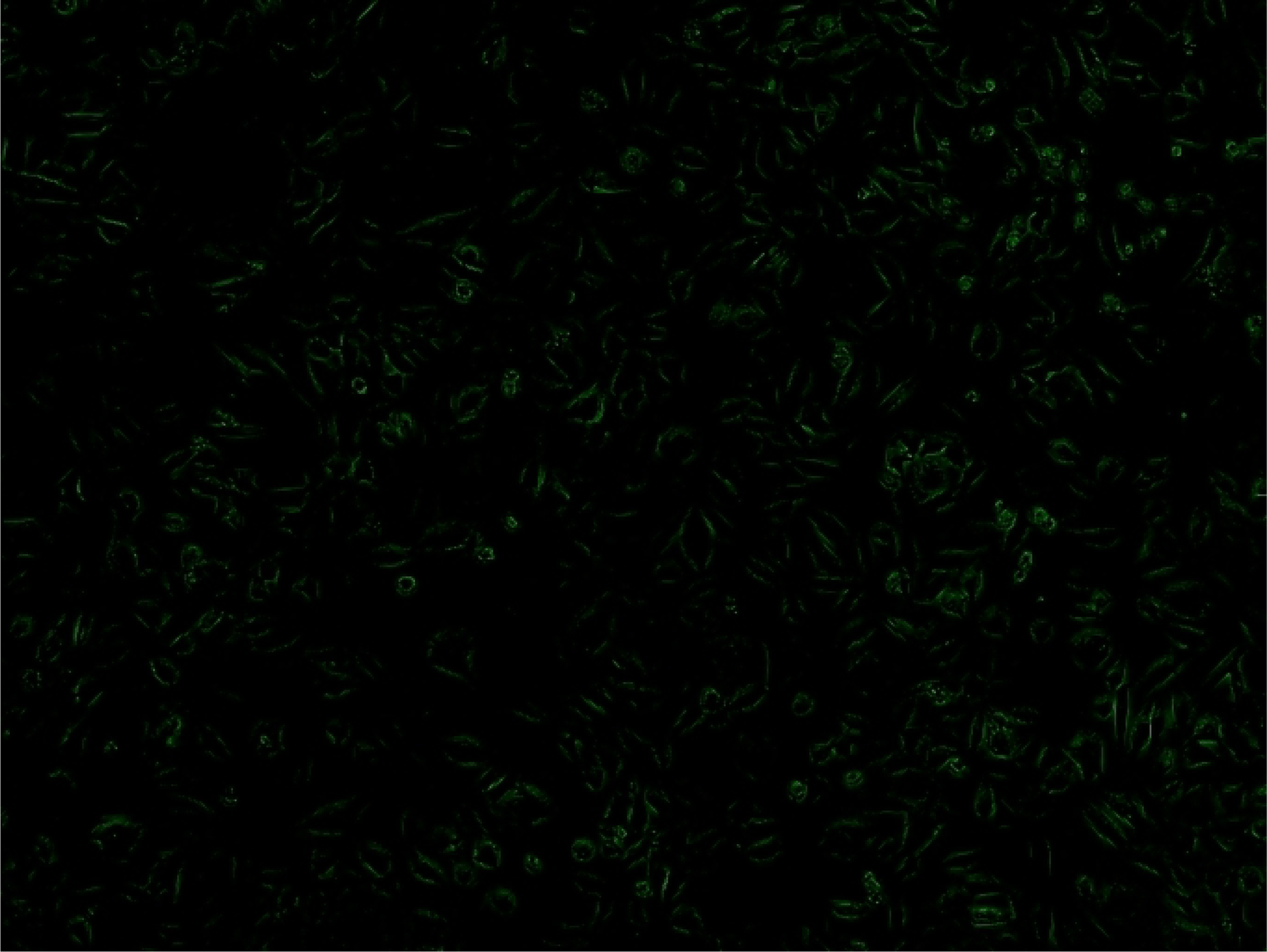

Supplement: S2 File — (ZIP) [file pone.0312791.s002.zip › Fig 5/Fig5 RP11 transfection efficiency/NC3 IF.tif]

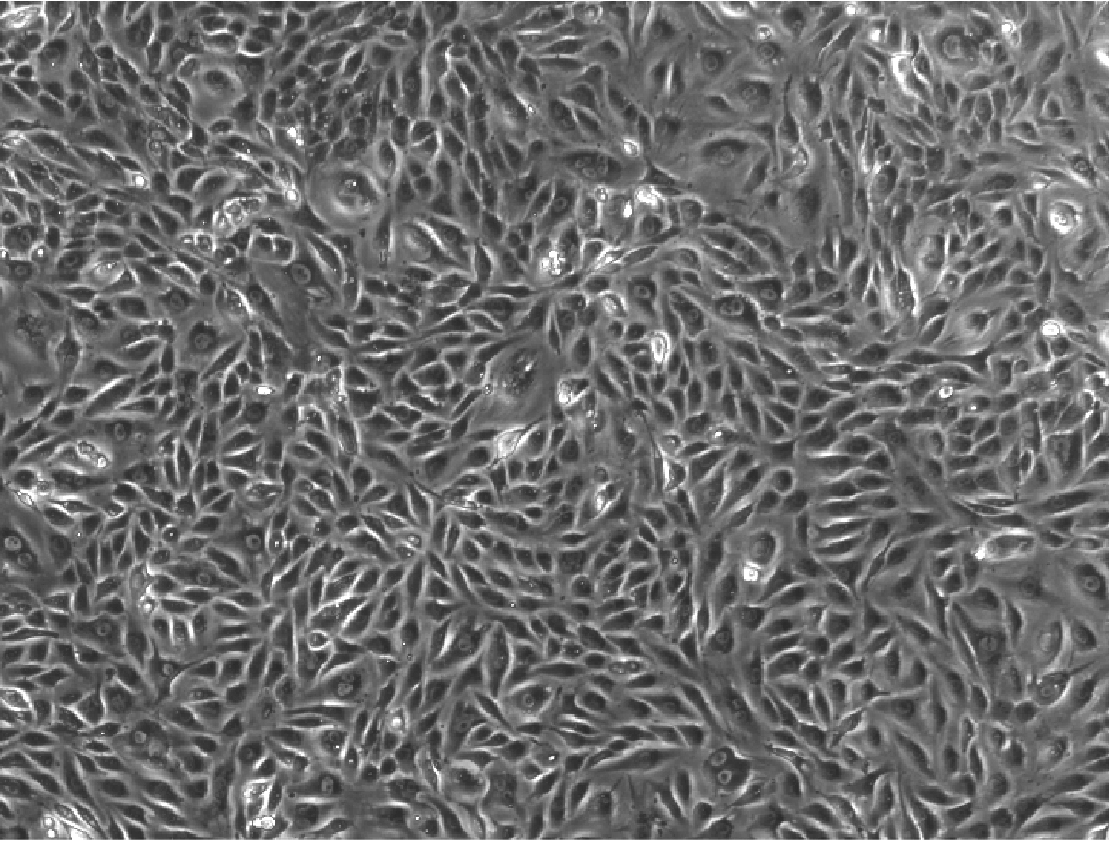

Supplement: S2 File — (ZIP) [file pone.0312791.s002.zip › Fig 5/Fig5 RP11 transfection efficiency/OE1 BF.tif]

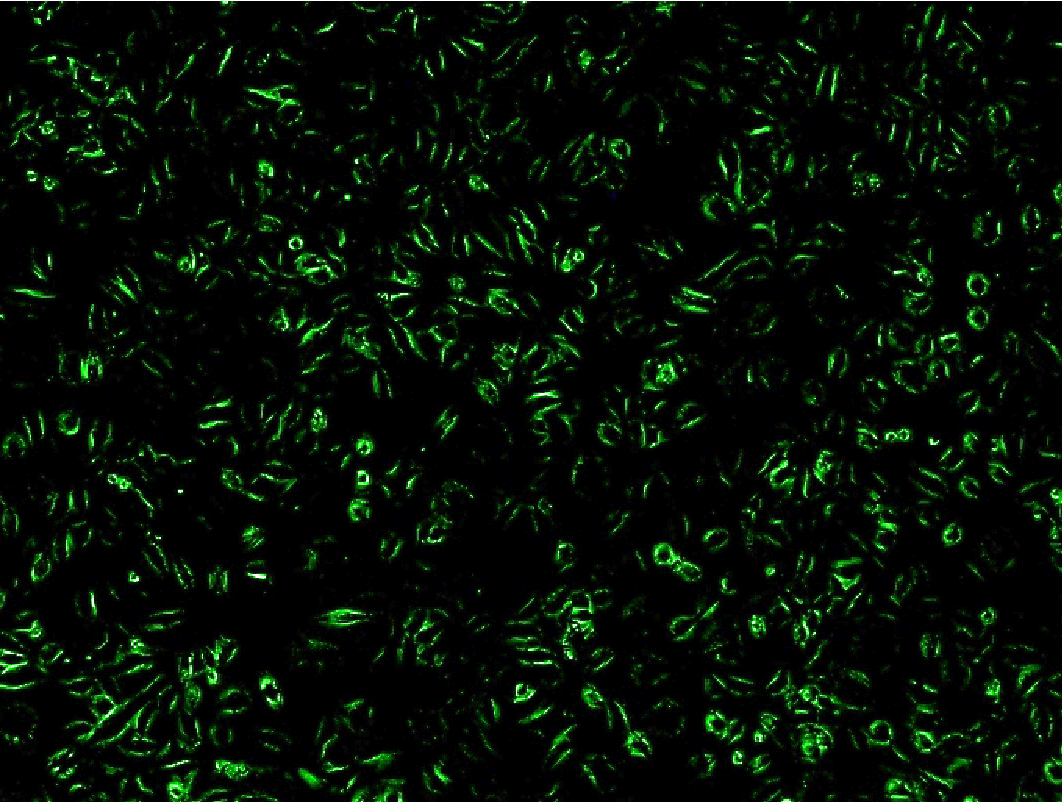

Supplement: S2 File — (ZIP) [file pone.0312791.s002.zip › Fig 5/Fig5 RP11 transfection efficiency/OE1 IF.tif]

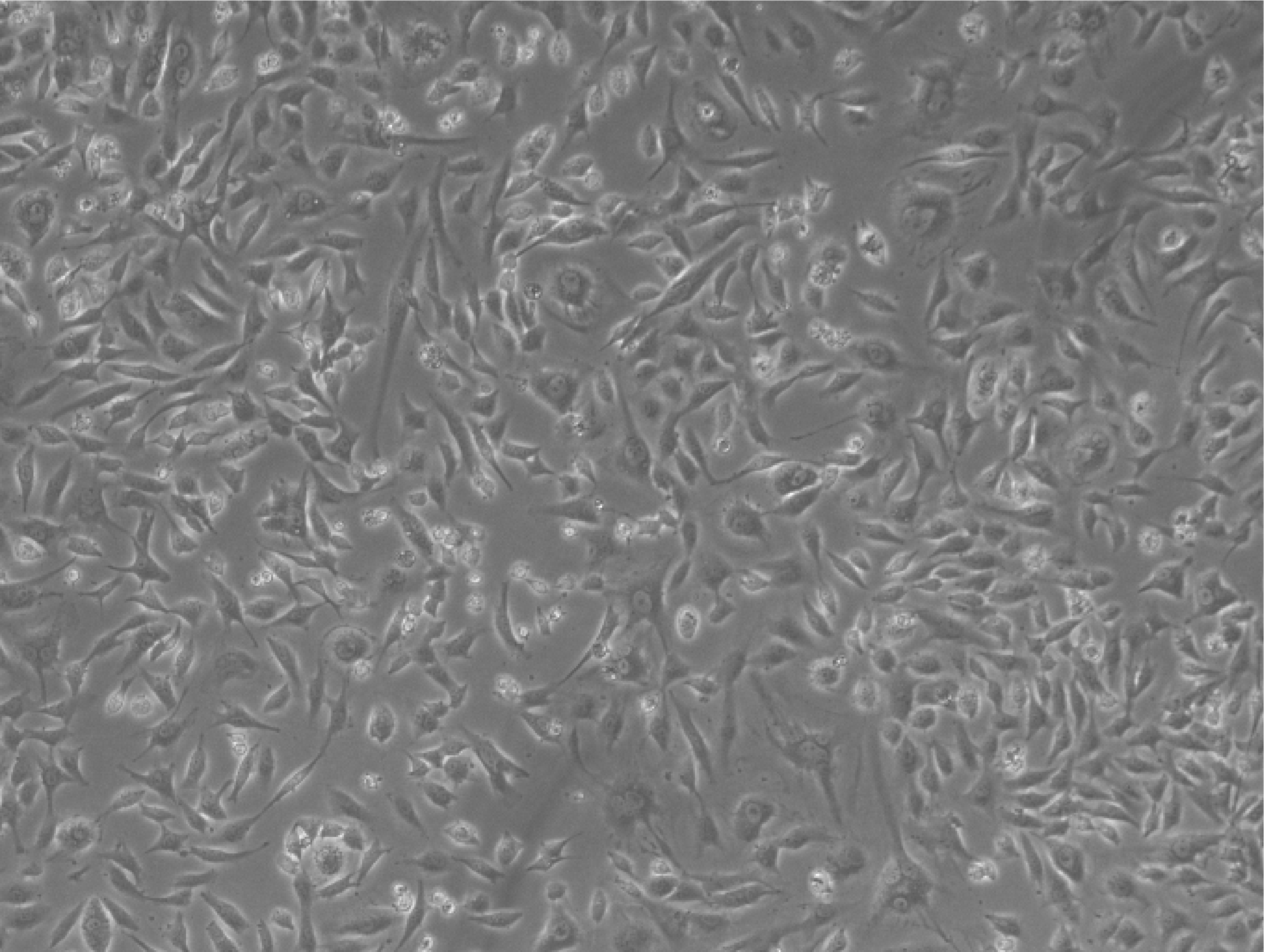

Supplement: S2 File — (ZIP) [file pone.0312791.s002.zip › Fig 5/Fig5 RP11 transfection efficiency/OE2 BF.tif]

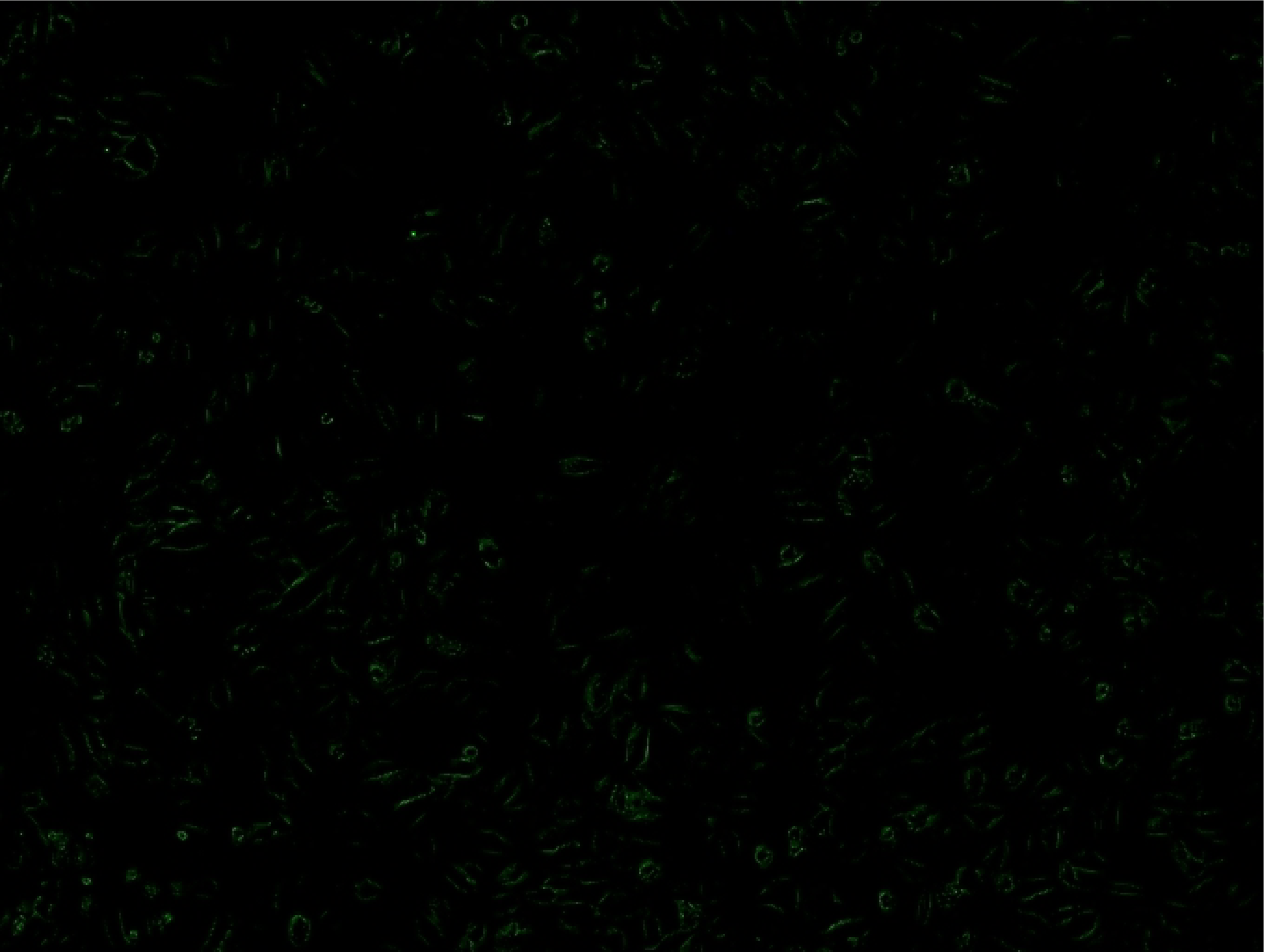

Supplement: S2 File — (ZIP) [file pone.0312791.s002.zip › Fig 5/Fig5 RP11 transfection efficiency/OE2 IF.tif]

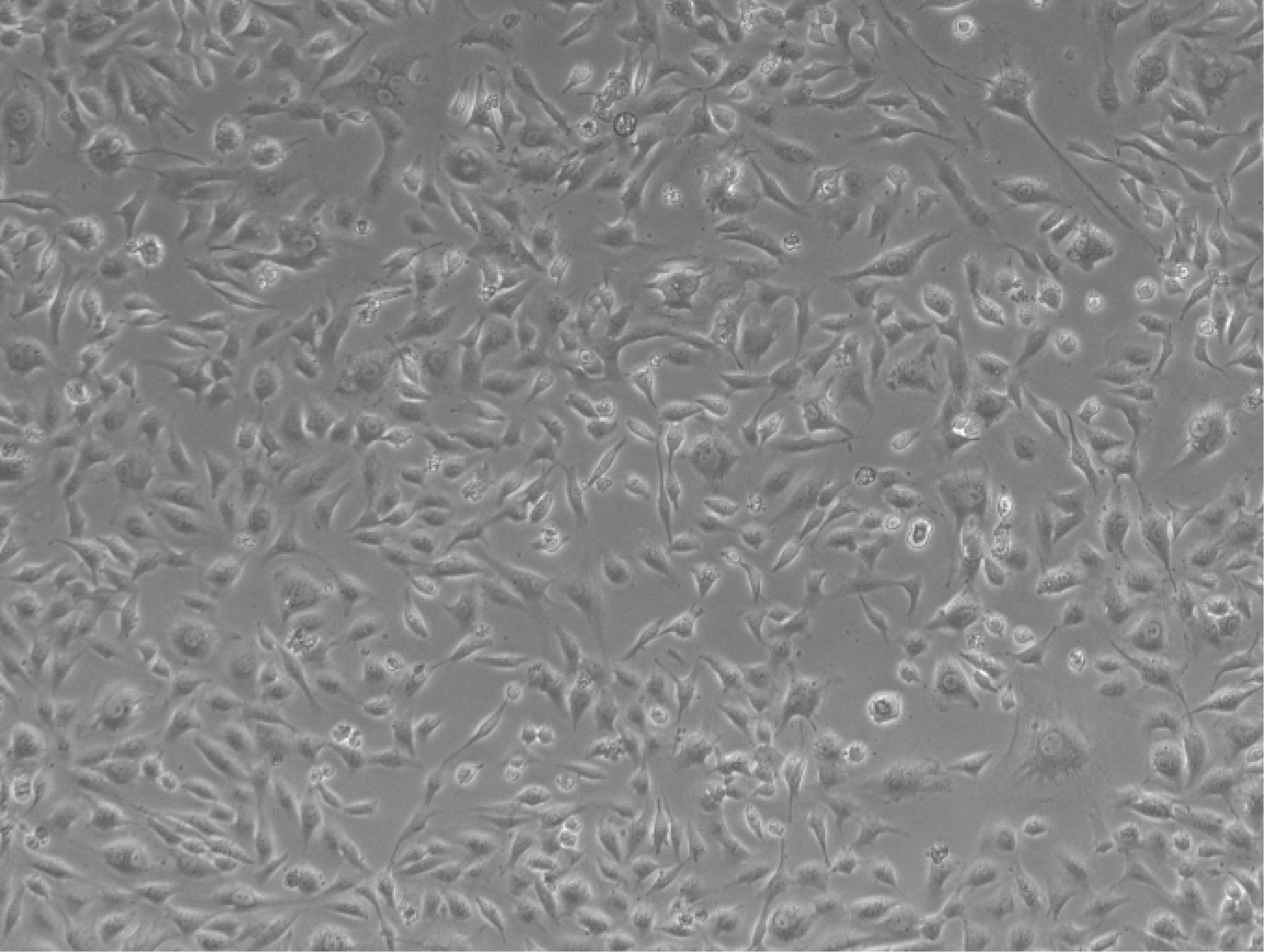

Supplement: S2 File — (ZIP) [file pone.0312791.s002.zip › Fig 5/Fig5 RP11 transfection efficiency/OE3 BF.tif]

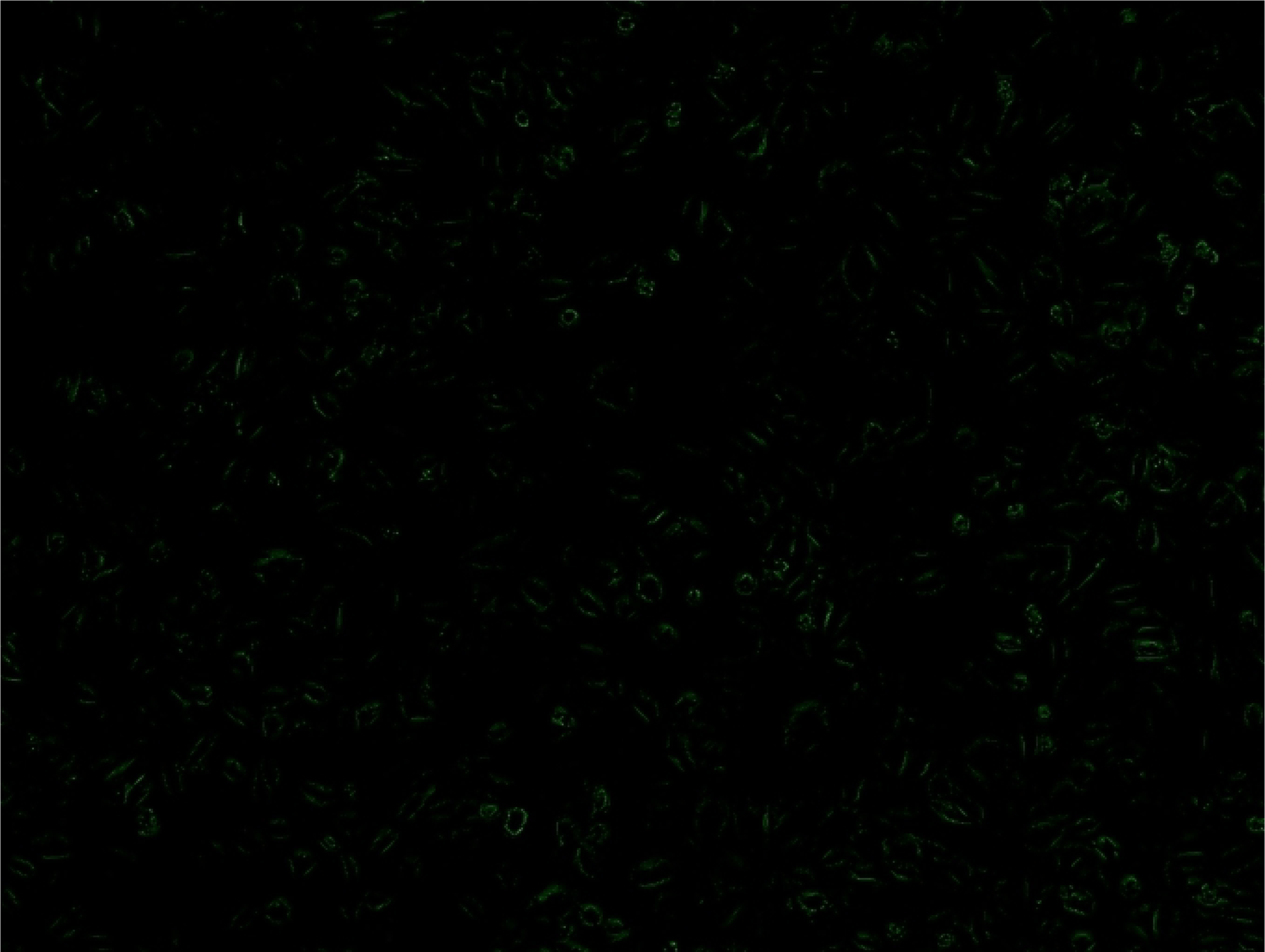

Supplement: S2 File — (ZIP) [file pone.0312791.s002.zip › Fig 5/Fig5 RP11 transfection efficiency/OE3 IF.tif]

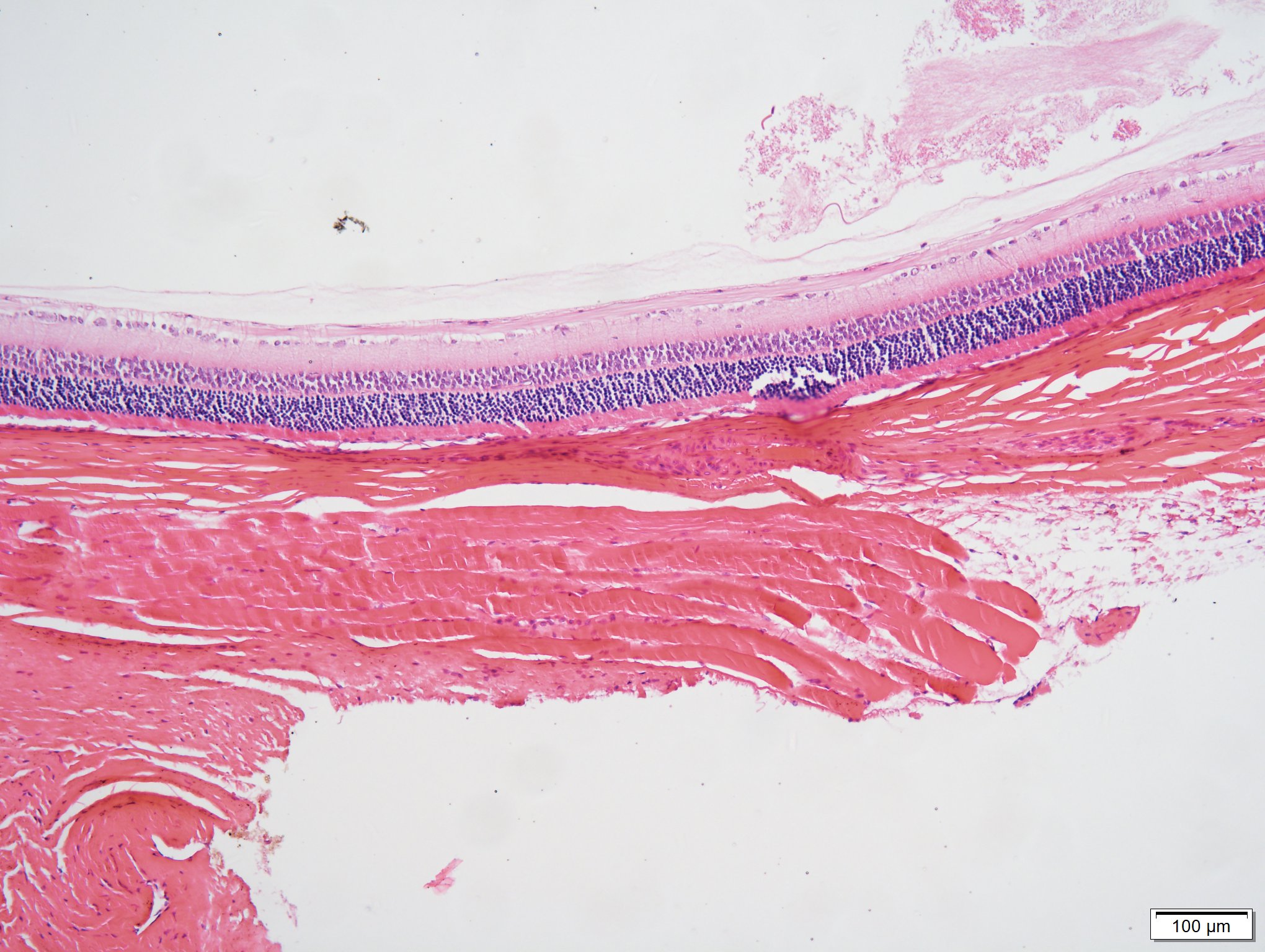

Supplement: S2 File — (ZIP) [file pone.0312791.s002.zip › Fig 6/Fig6 HE/DM-NC1 100um.jpg]

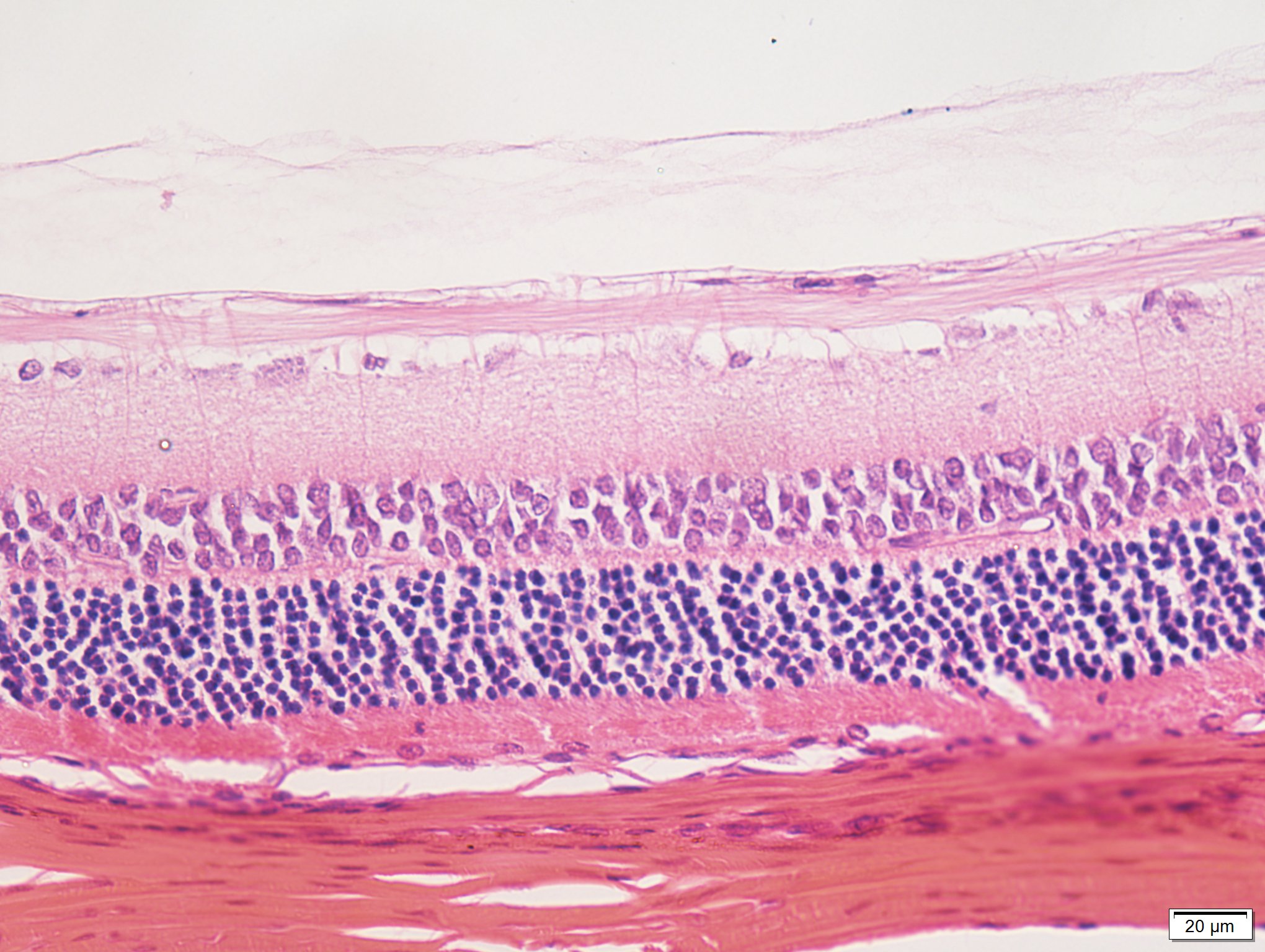

Supplement: S2 File — (ZIP) [file pone.0312791.s002.zip › Fig 6/Fig6 HE/DM-NC1 20um.jpg]

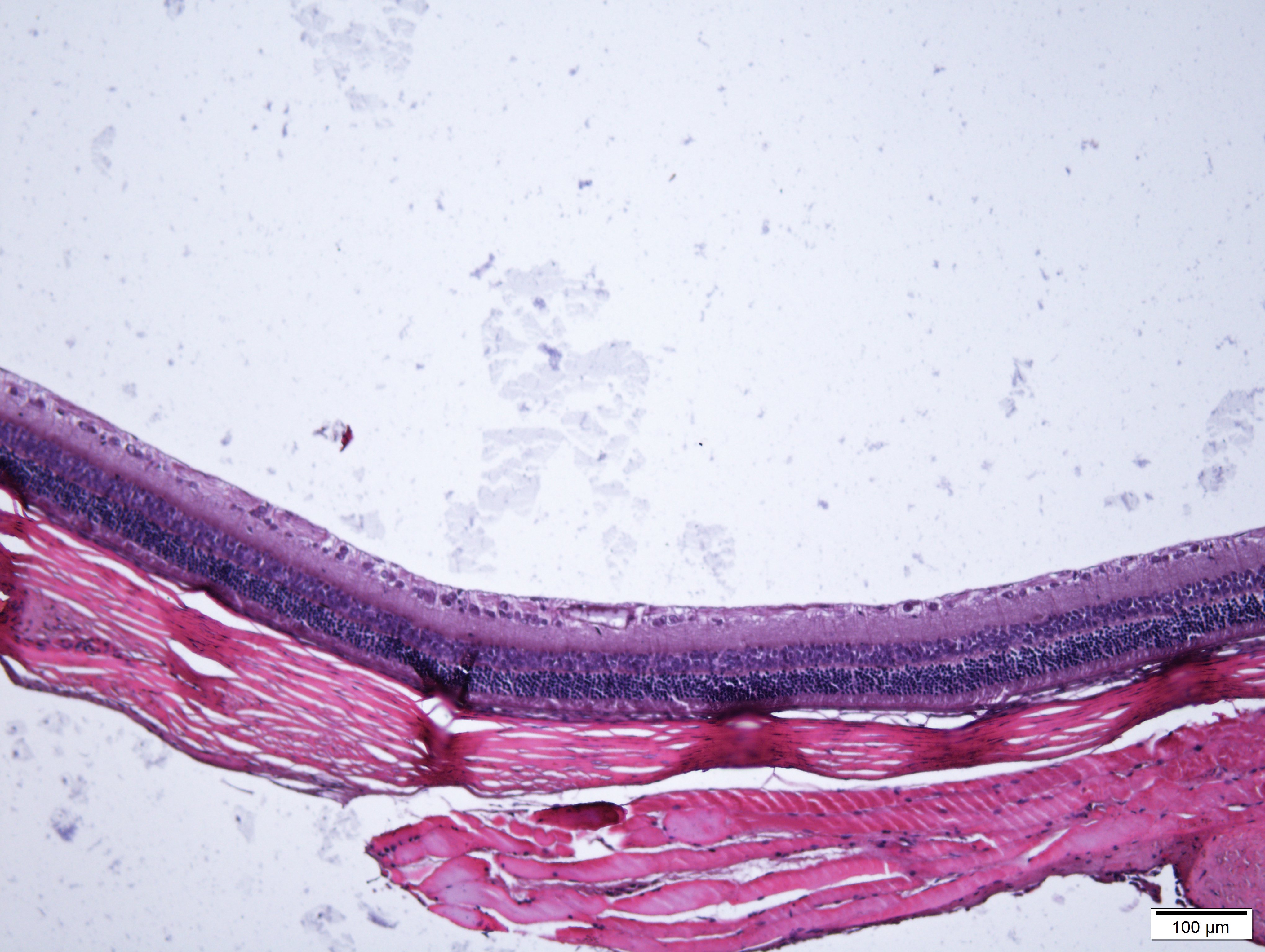

Supplement: S2 File — (ZIP) [file pone.0312791.s002.zip › Fig 6/Fig6 HE/DM-NC2 100um.jpg]

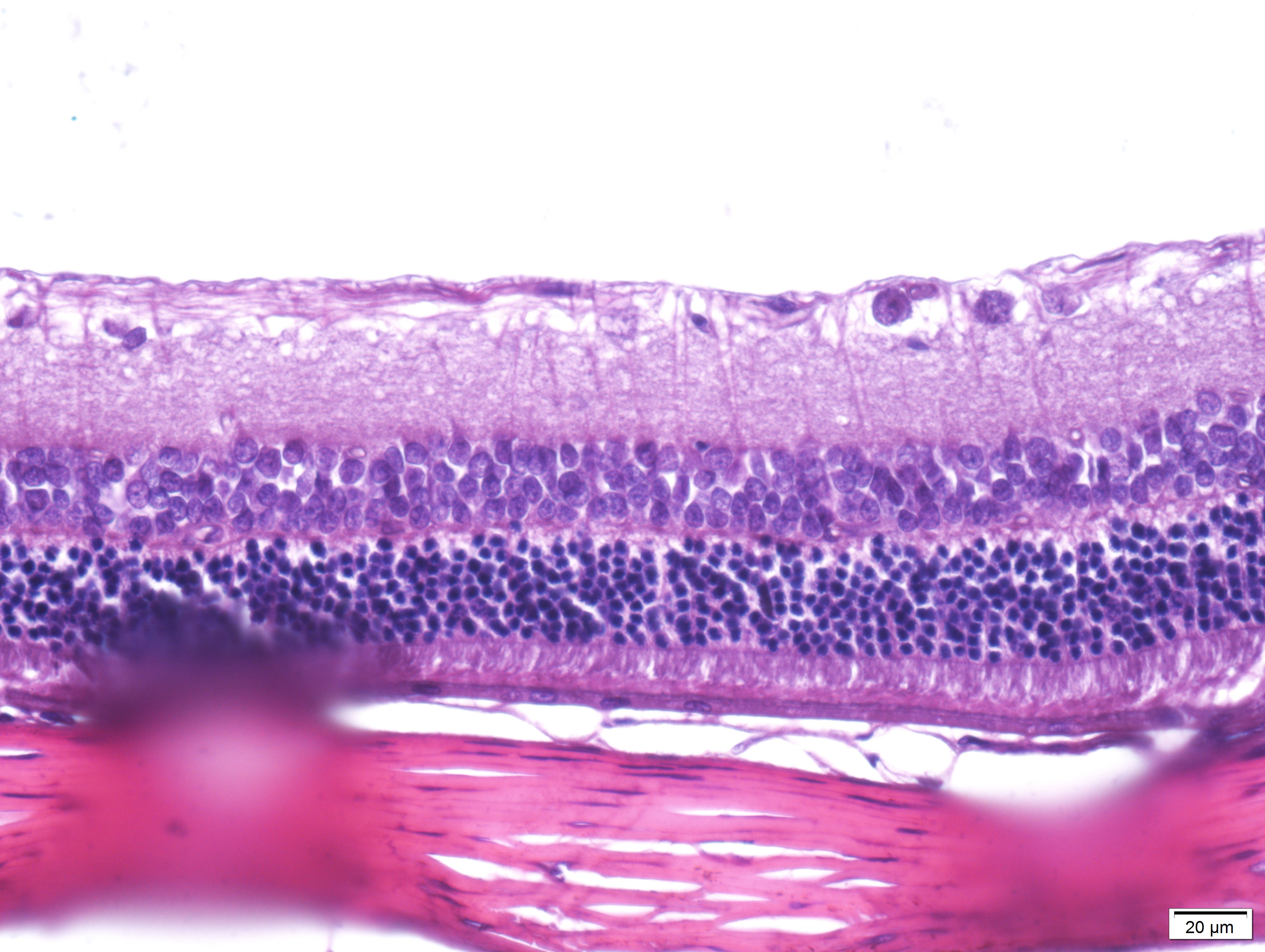

Supplement: S2 File — (ZIP) [file pone.0312791.s002.zip › Fig 6/Fig6 HE/DM-NC2 20um.jpg]

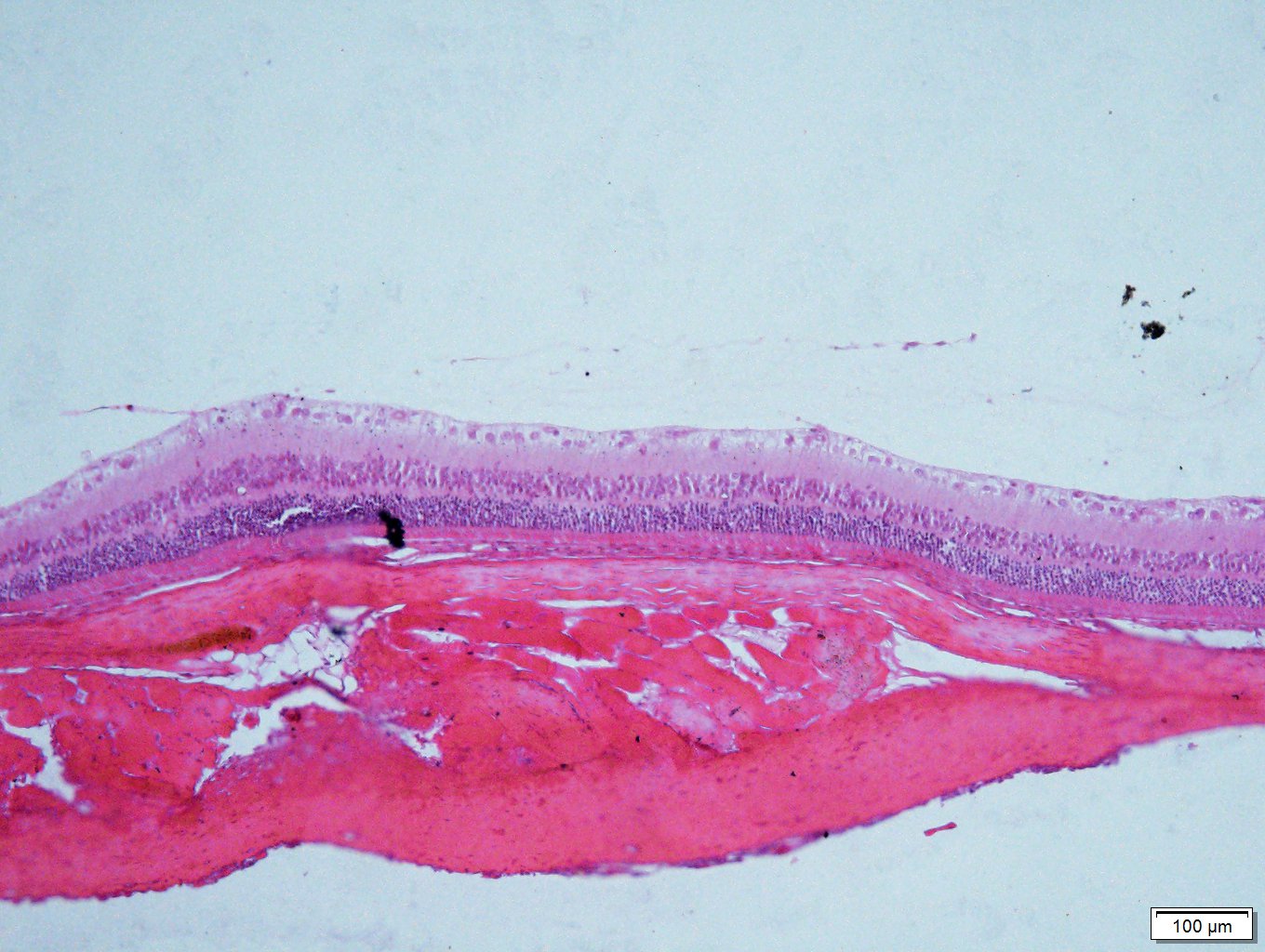

Supplement: S2 File — (ZIP) [file pone.0312791.s002.zip › Fig 6/Fig6 HE/DM-NC3 100um.jpg]

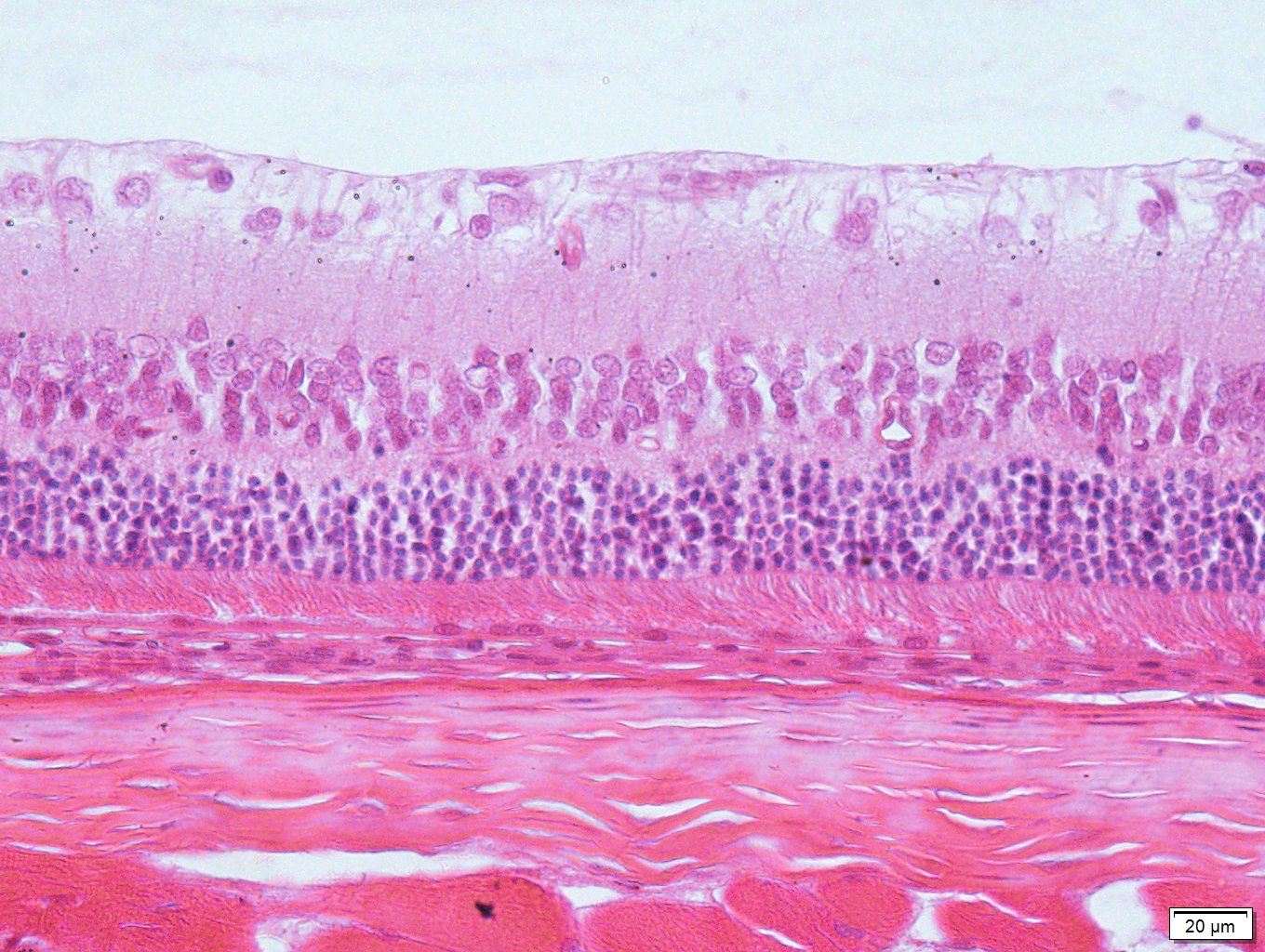

Supplement: S2 File — (ZIP) [file pone.0312791.s002.zip › Fig 6/Fig6 HE/DM-NC3 20um.jpg]

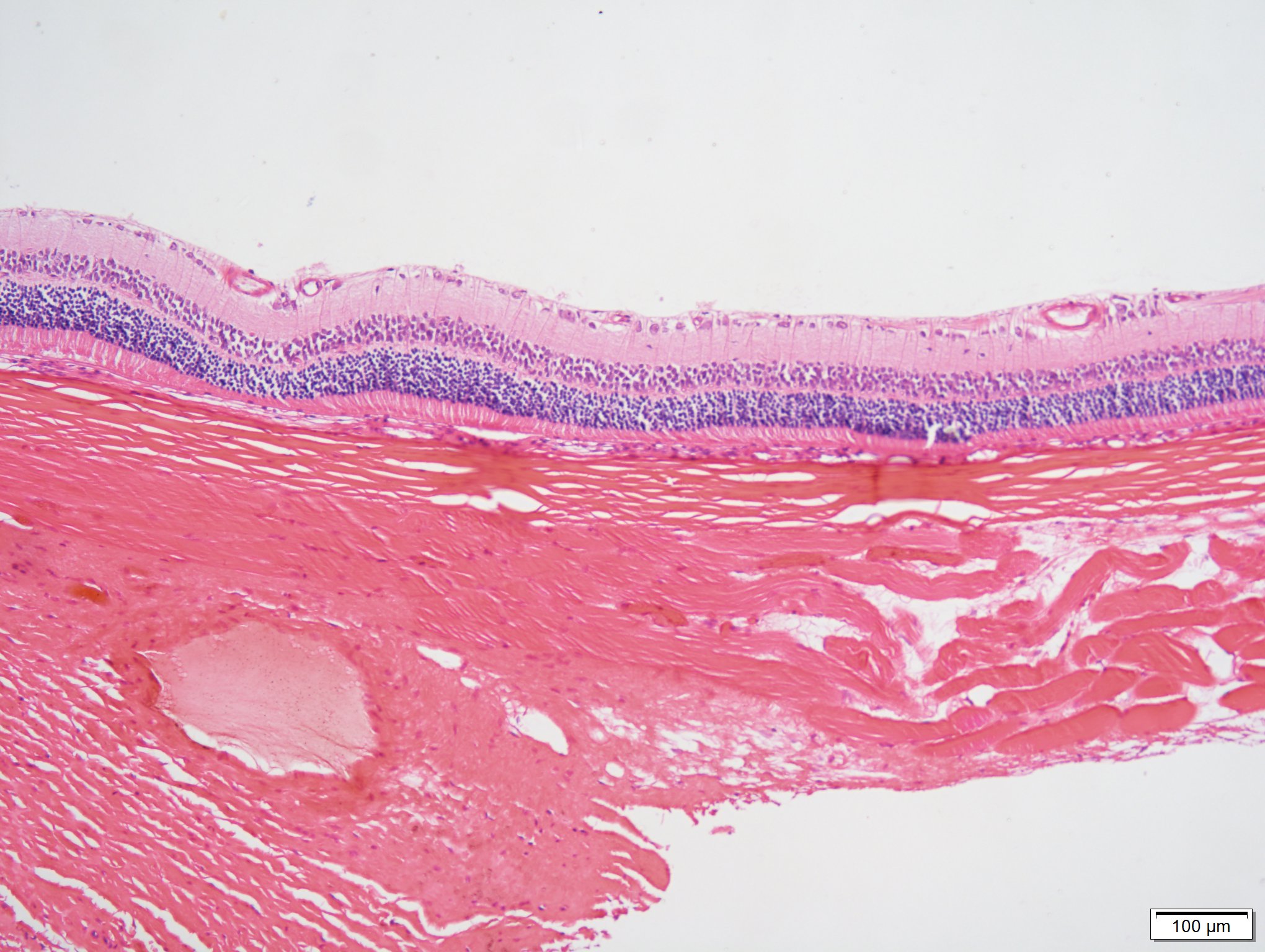

Supplement: S2 File — (ZIP) [file pone.0312791.s002.zip › Fig 6/Fig6 HE/DM-OE1 100um.jpg]

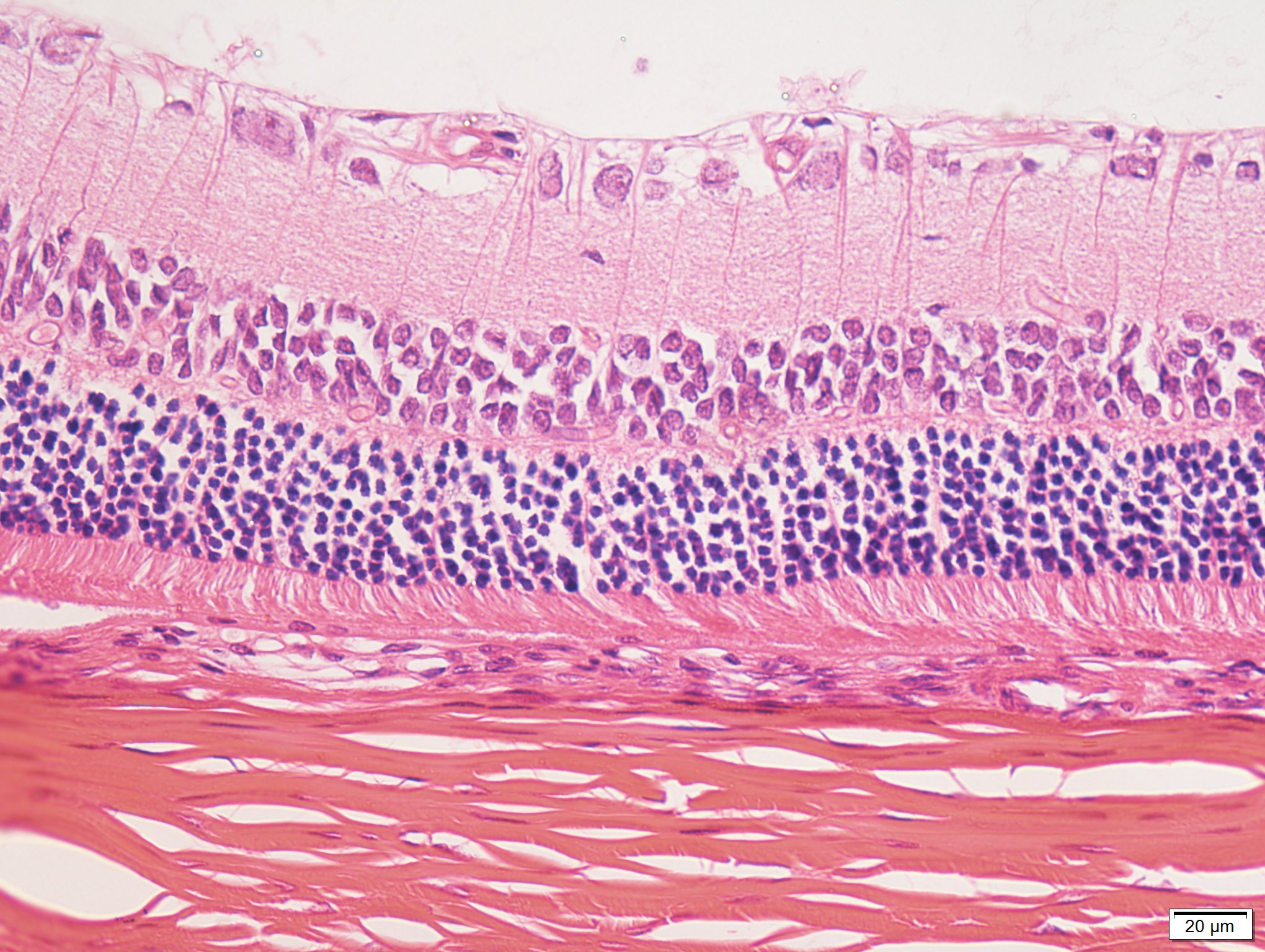

Supplement: S2 File — (ZIP) [file pone.0312791.s002.zip › Fig 6/Fig6 HE/DM-OE1 20um.jpg]

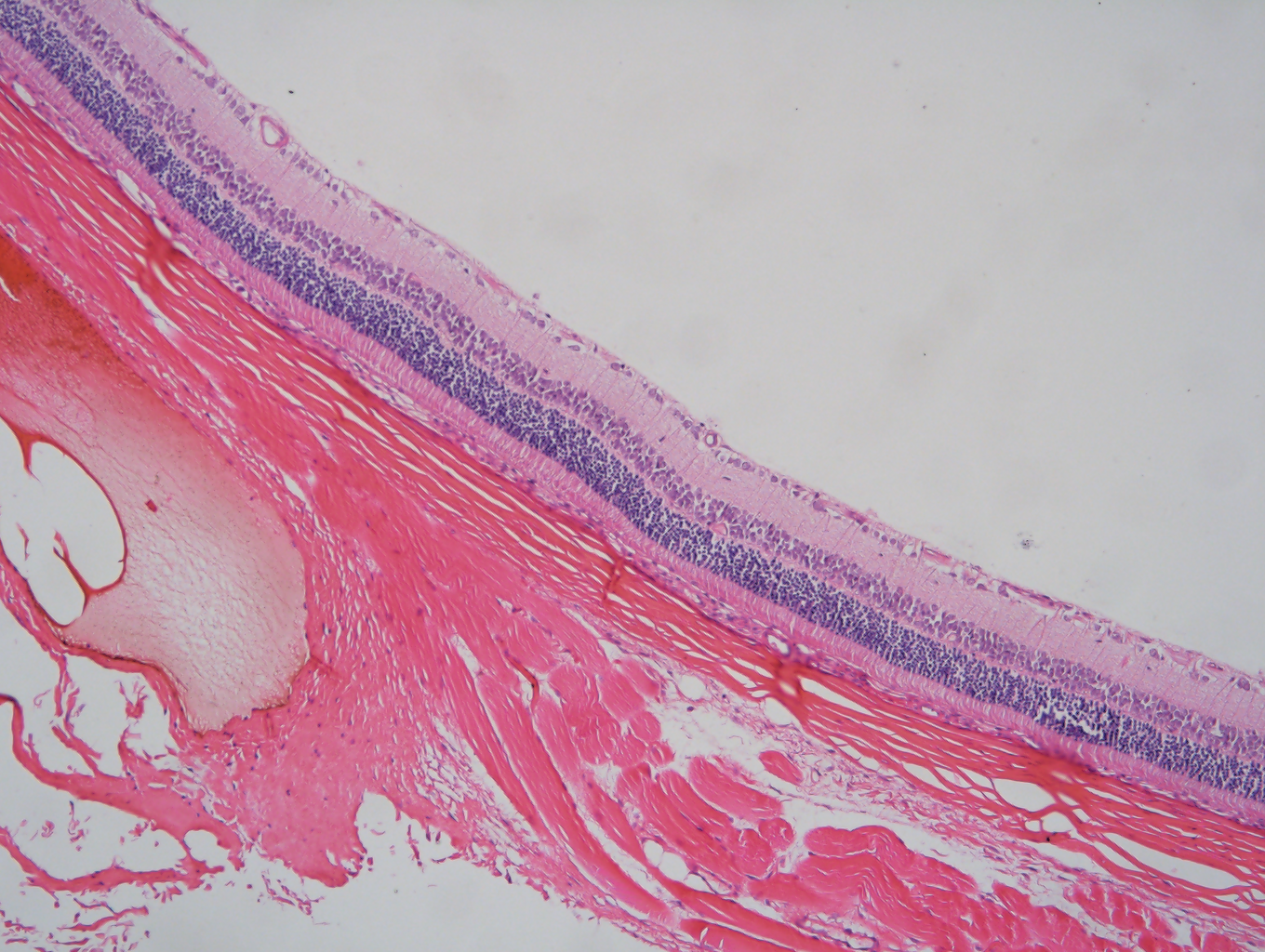

Supplement: S2 File — (ZIP) [file pone.0312791.s002.zip › Fig 6/Fig6 HE/DM-OE2 100um.tif]

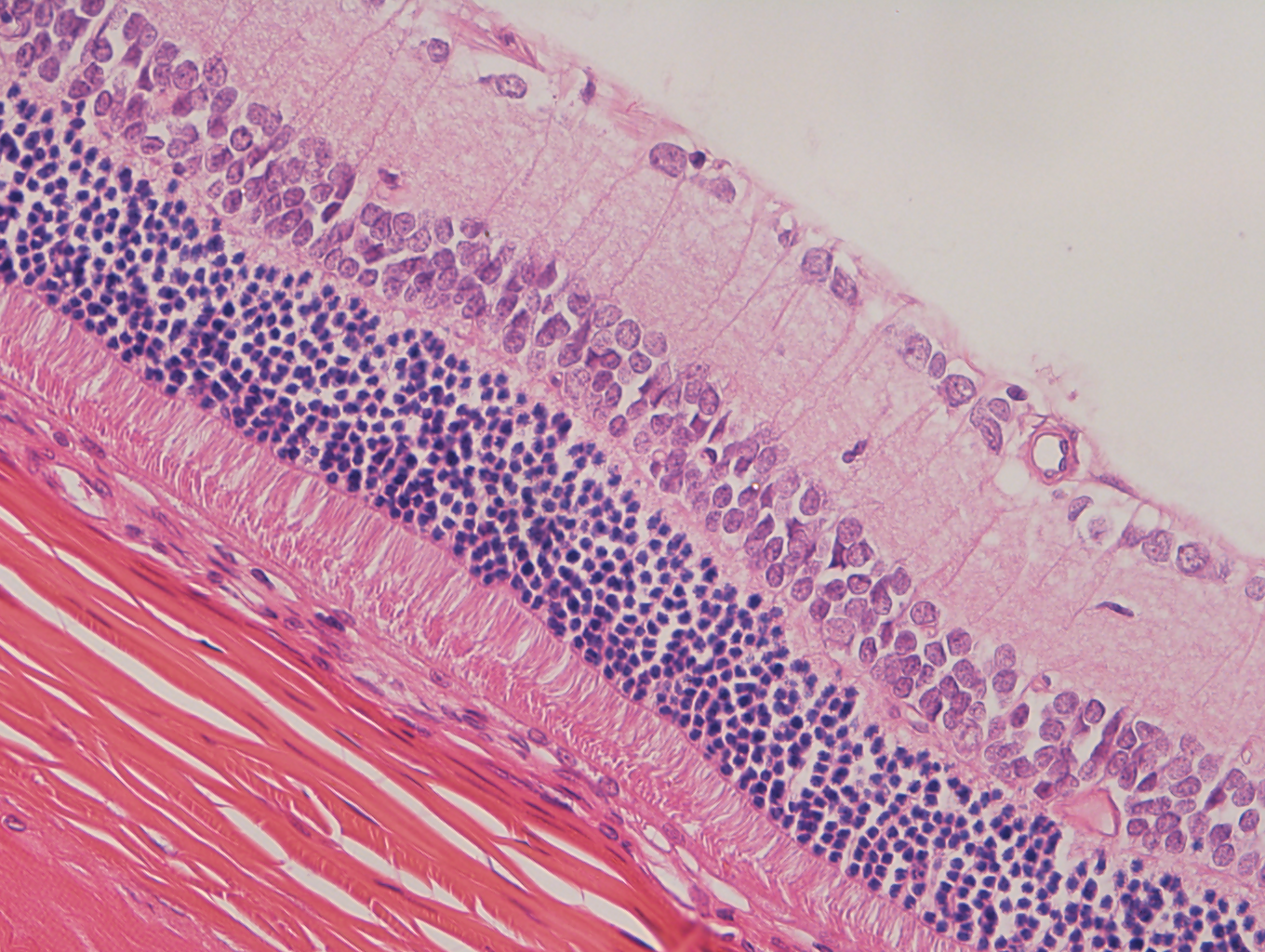

Supplement: S2 File — (ZIP) [file pone.0312791.s002.zip › Fig 6/Fig6 HE/DM-OE2 20um.tif]

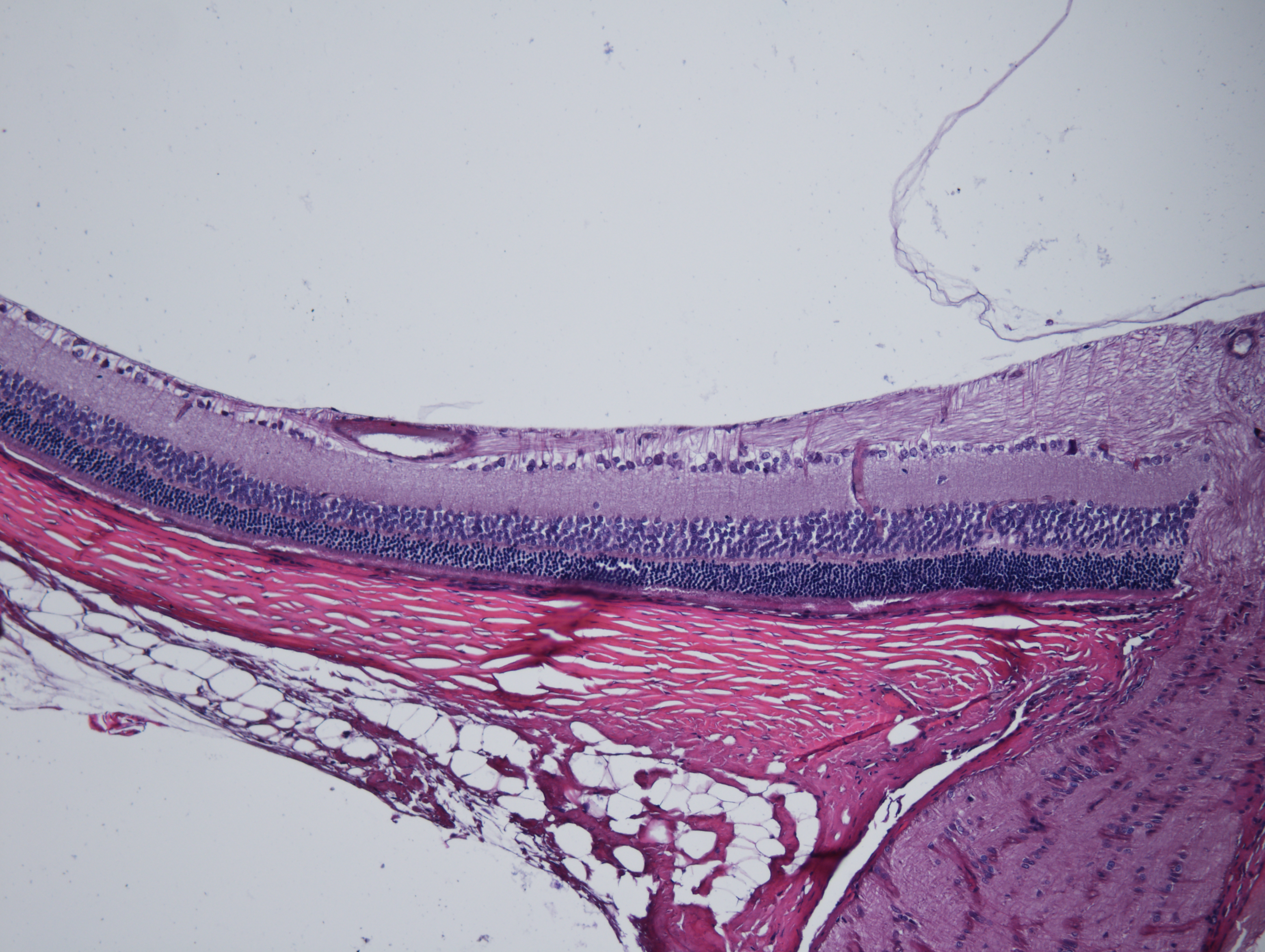

Supplement: S2 File — (ZIP) [file pone.0312791.s002.zip › Fig 6/Fig6 HE/DM-OE3 100um.tif]

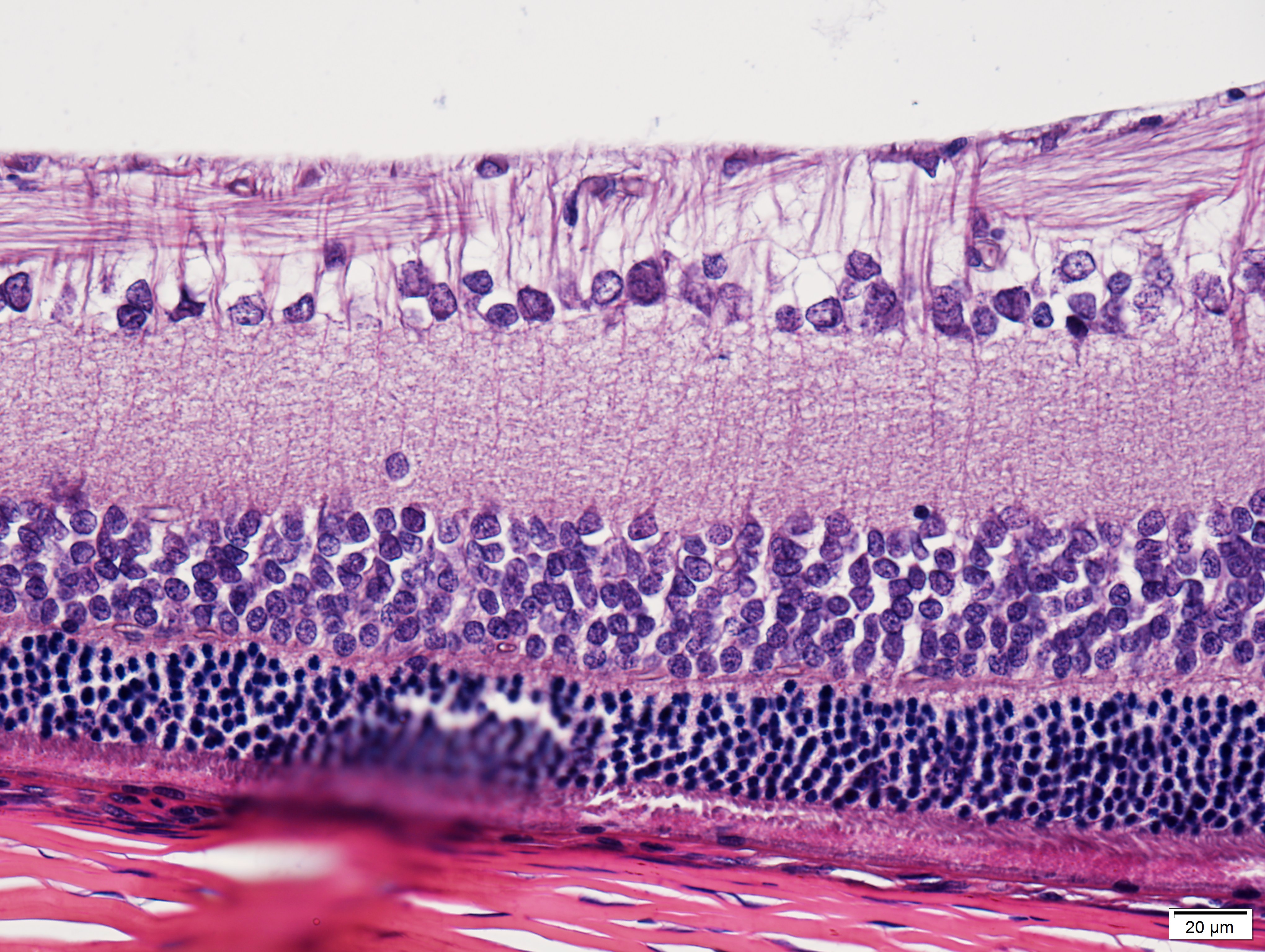

Supplement: S2 File — (ZIP) [file pone.0312791.s002.zip › Fig 6/Fig6 HE/DM-OE3 20um.jpg]

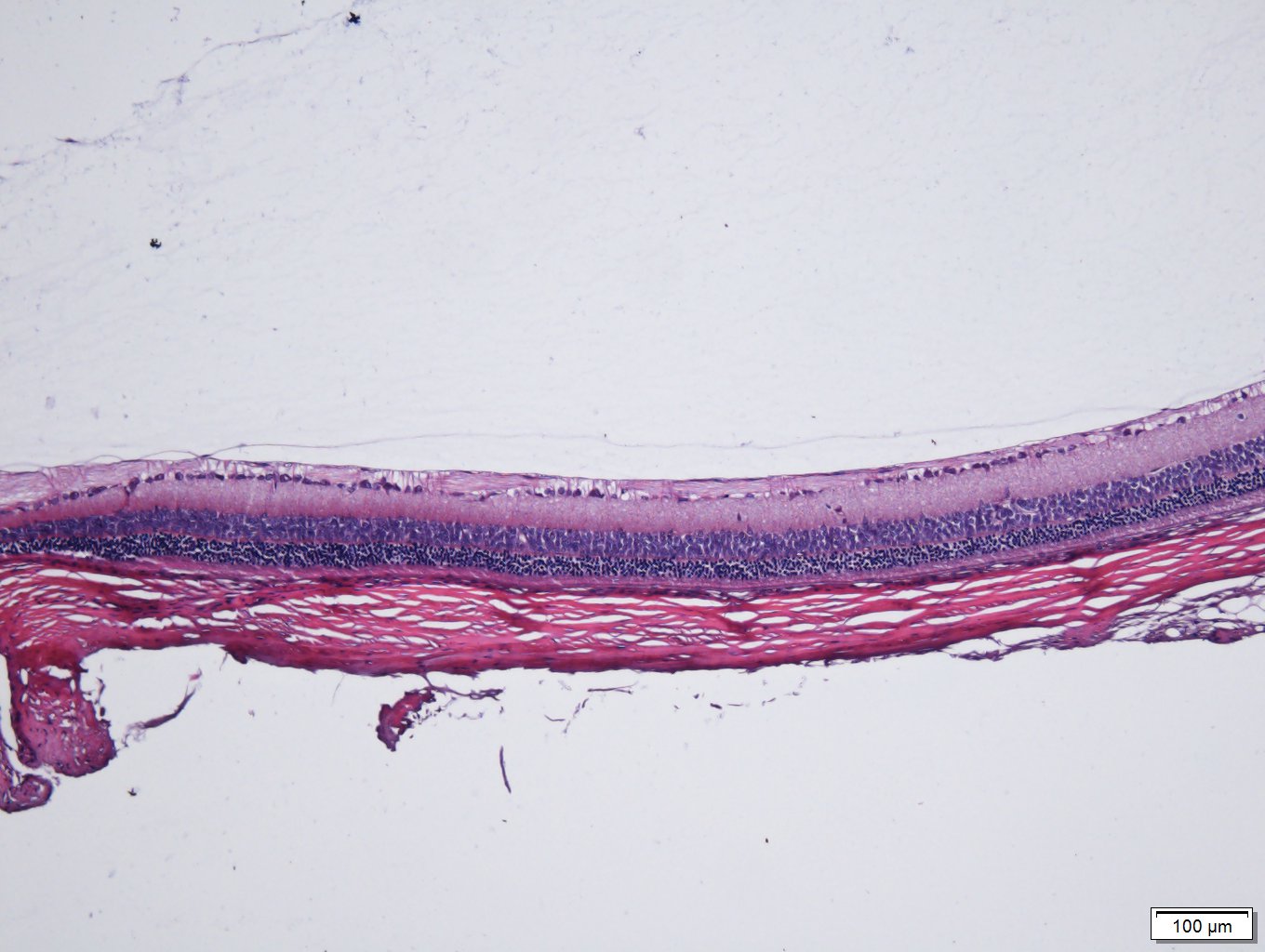

Supplement: S2 File — (ZIP) [file pone.0312791.s002.zip › Fig 6/Fig6 HE/DM2 100um.jpg]

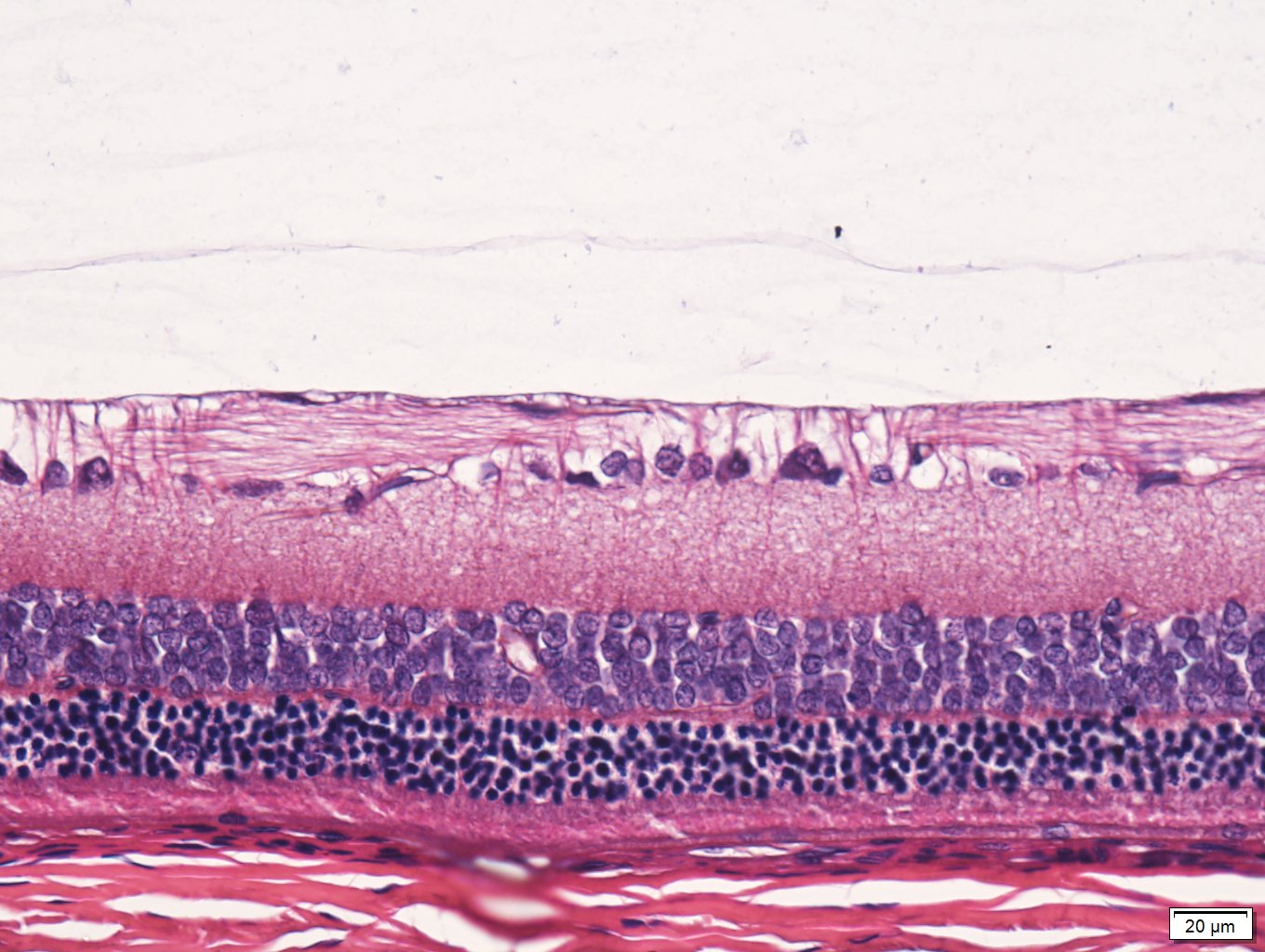

Supplement: S2 File — (ZIP) [file pone.0312791.s002.zip › Fig 6/Fig6 HE/DM2 20um.jpg]

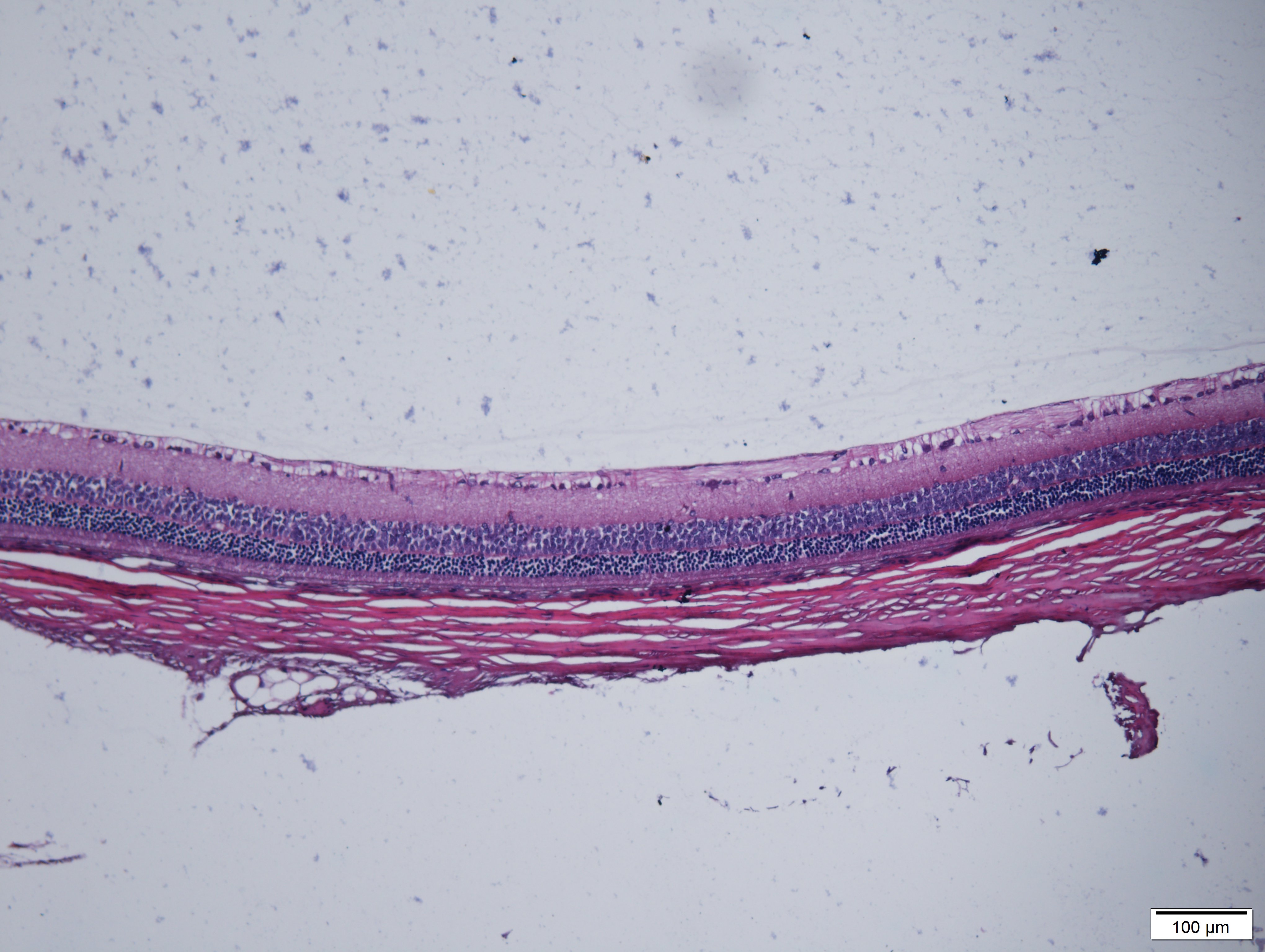

Supplement: S2 File — (ZIP) [file pone.0312791.s002.zip › Fig 6/Fig6 HE/DM3 100um.jpg]

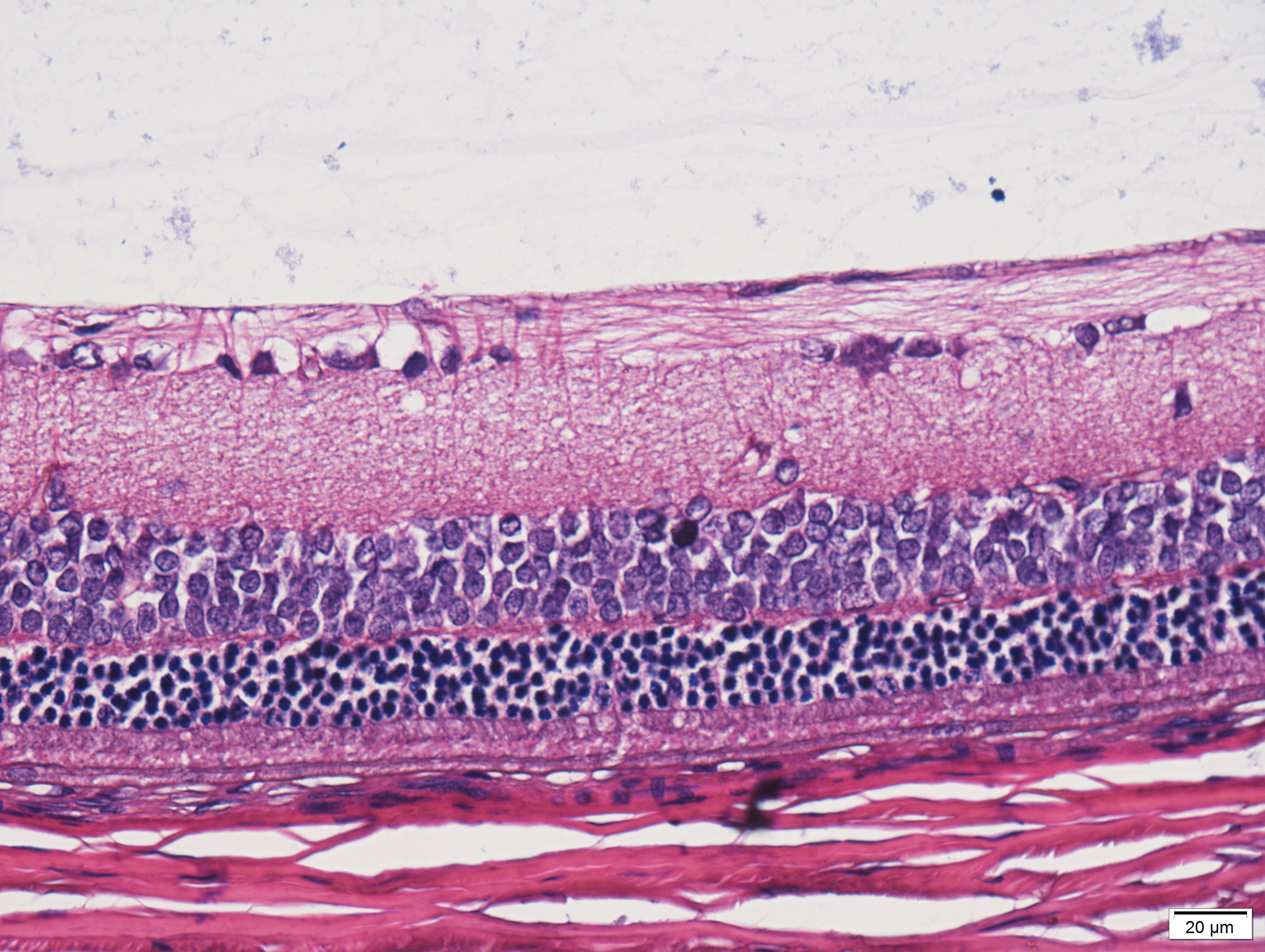

Supplement: S2 File — (ZIP) [file pone.0312791.s002.zip › Fig 6/Fig6 HE/DM3 20um.jpg]

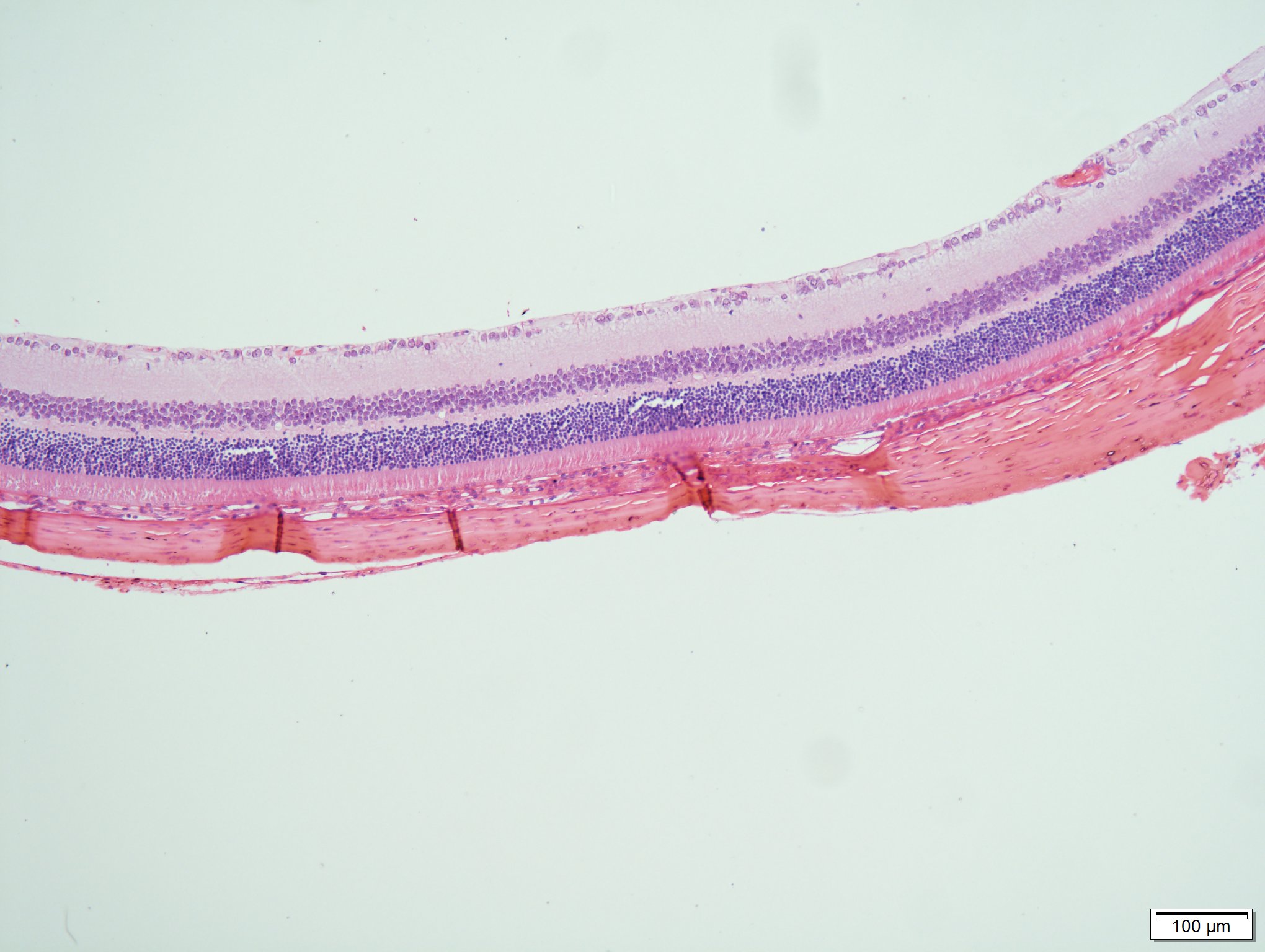

Supplement: S2 File — (ZIP) [file pone.0312791.s002.zip › Fig 6/Fig6 HE/WT1 100um.jpg]

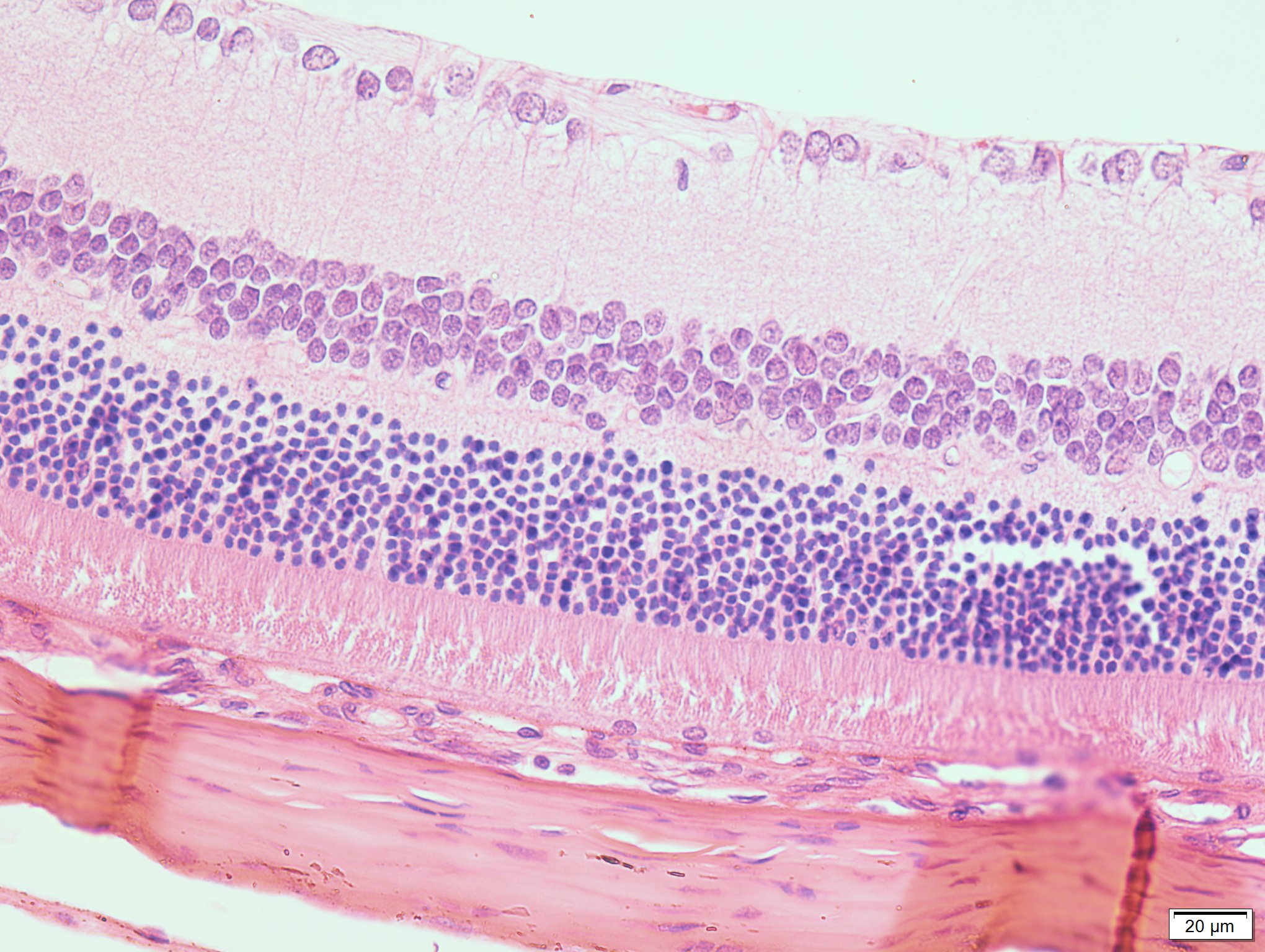

Supplement: S2 File — (ZIP) [file pone.0312791.s002.zip › Fig 6/Fig6 HE/WT1 20um.jpg]

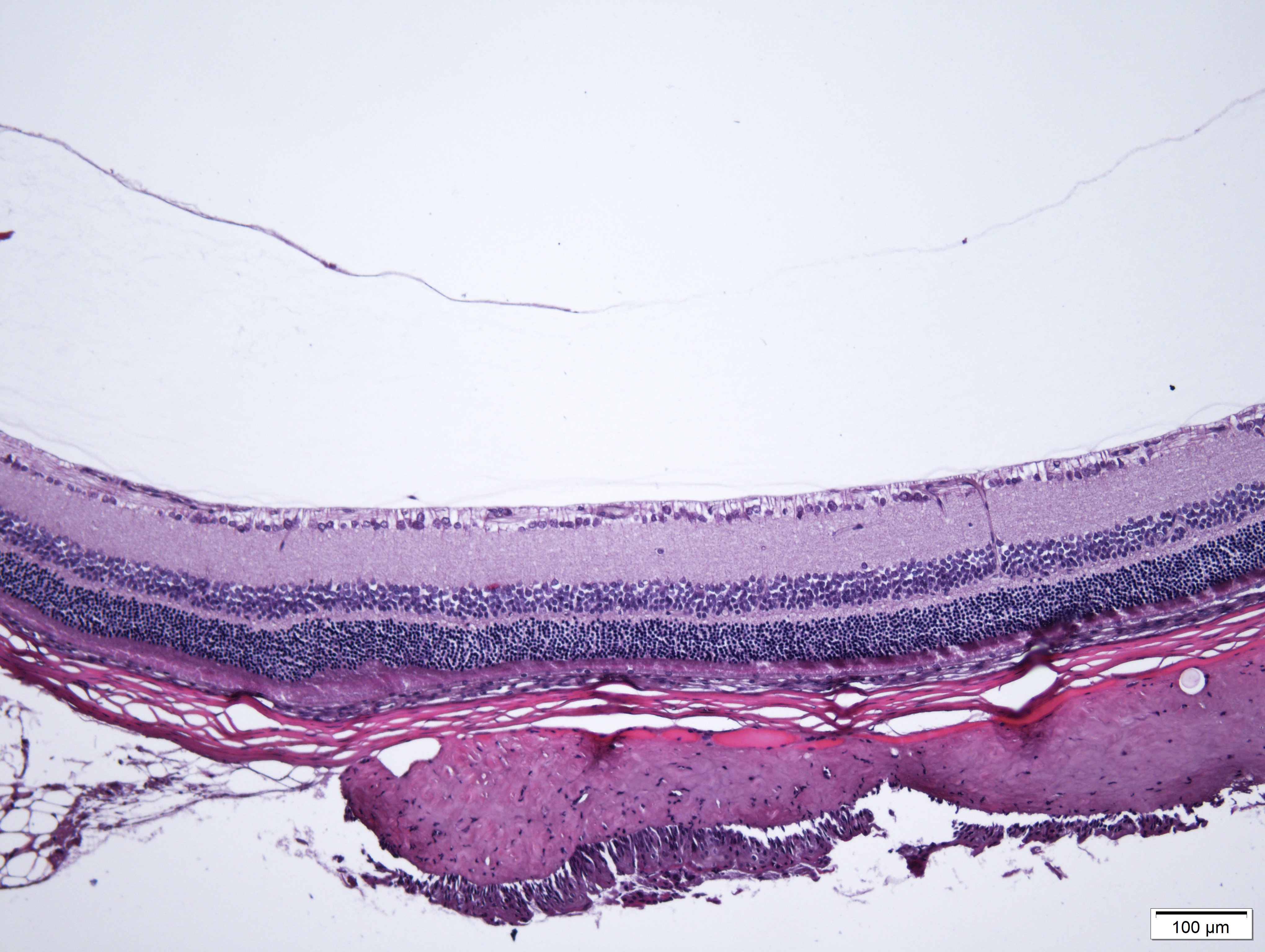

Supplement: S2 File — (ZIP) [file pone.0312791.s002.zip › Fig 6/Fig6 HE/WT2 100um.jpg]
