# Supplementary figures and images for: Targeting long non-coding RNA RP11-502I4.3 inhibits the trend of angiogenesis in diabetic retinopathy (part 2 of 2)
Source: PLoS One. 2025 May 14;20(5):e0312791. doi: 10.1371/journal.pone.0312791 (PMC12077687; doi:10.1371/journal.pone.0312791)

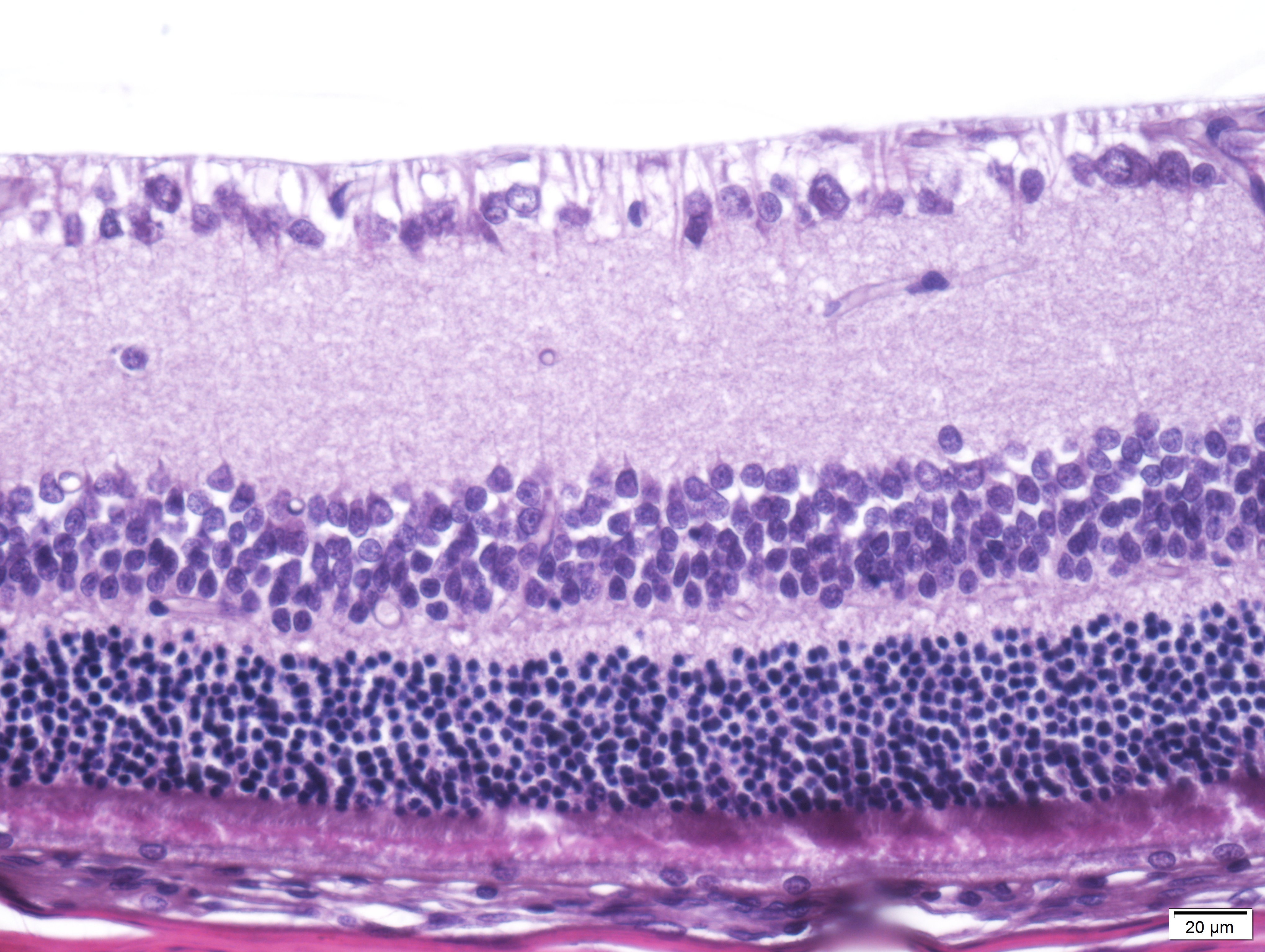

Supplement: S2 File — (ZIP) [file pone.0312791.s002.zip › Fig 6/Fig6 HE/WT2 20um.jpg]

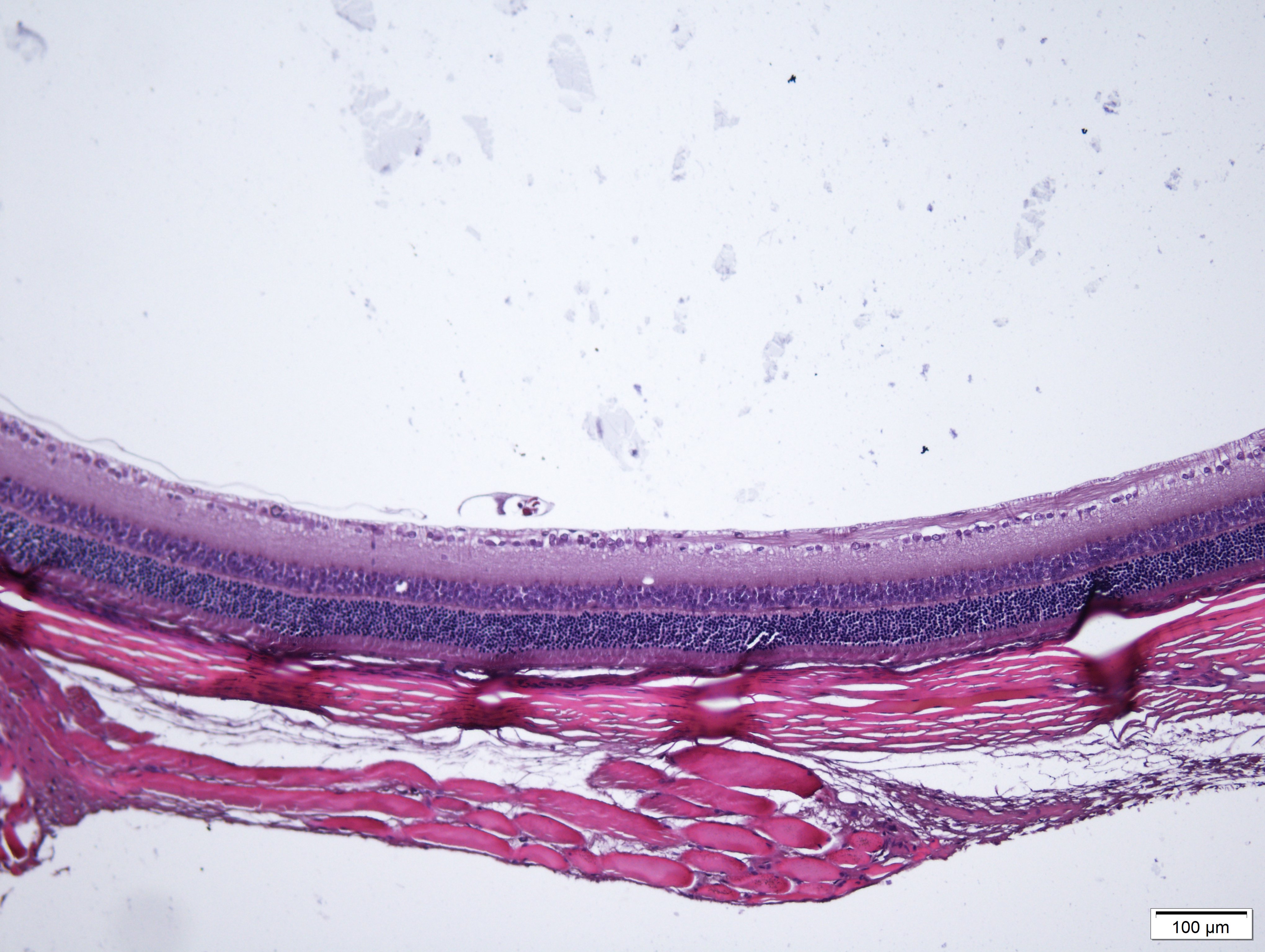

Supplement: S2 File — (ZIP) [file pone.0312791.s002.zip › Fig 6/Fig6 HE/WT3 100um.jpg]

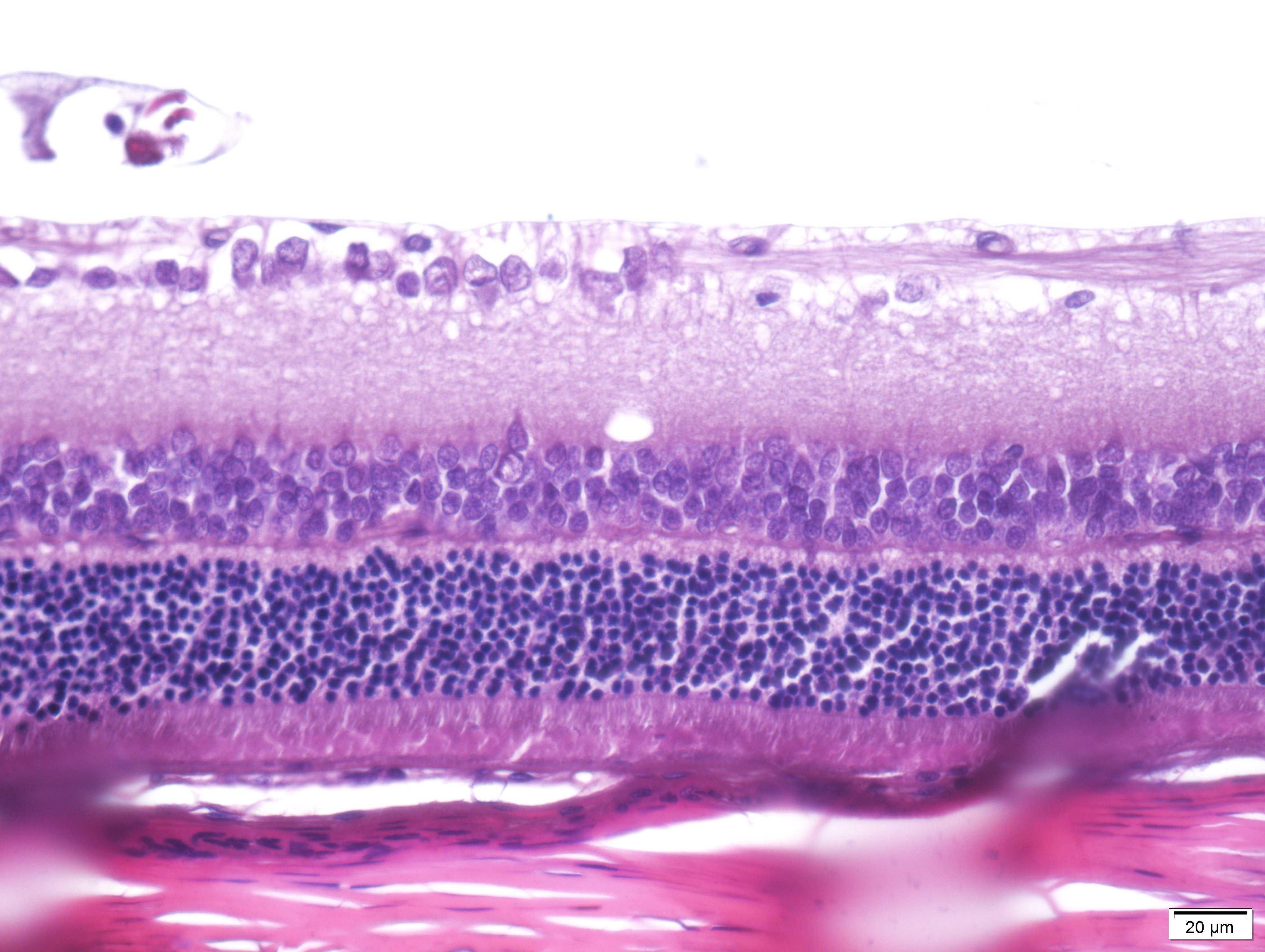

Supplement: S2 File — (ZIP) [file pone.0312791.s002.zip › Fig 6/Fig6 HE/WT3 20um.jpg]

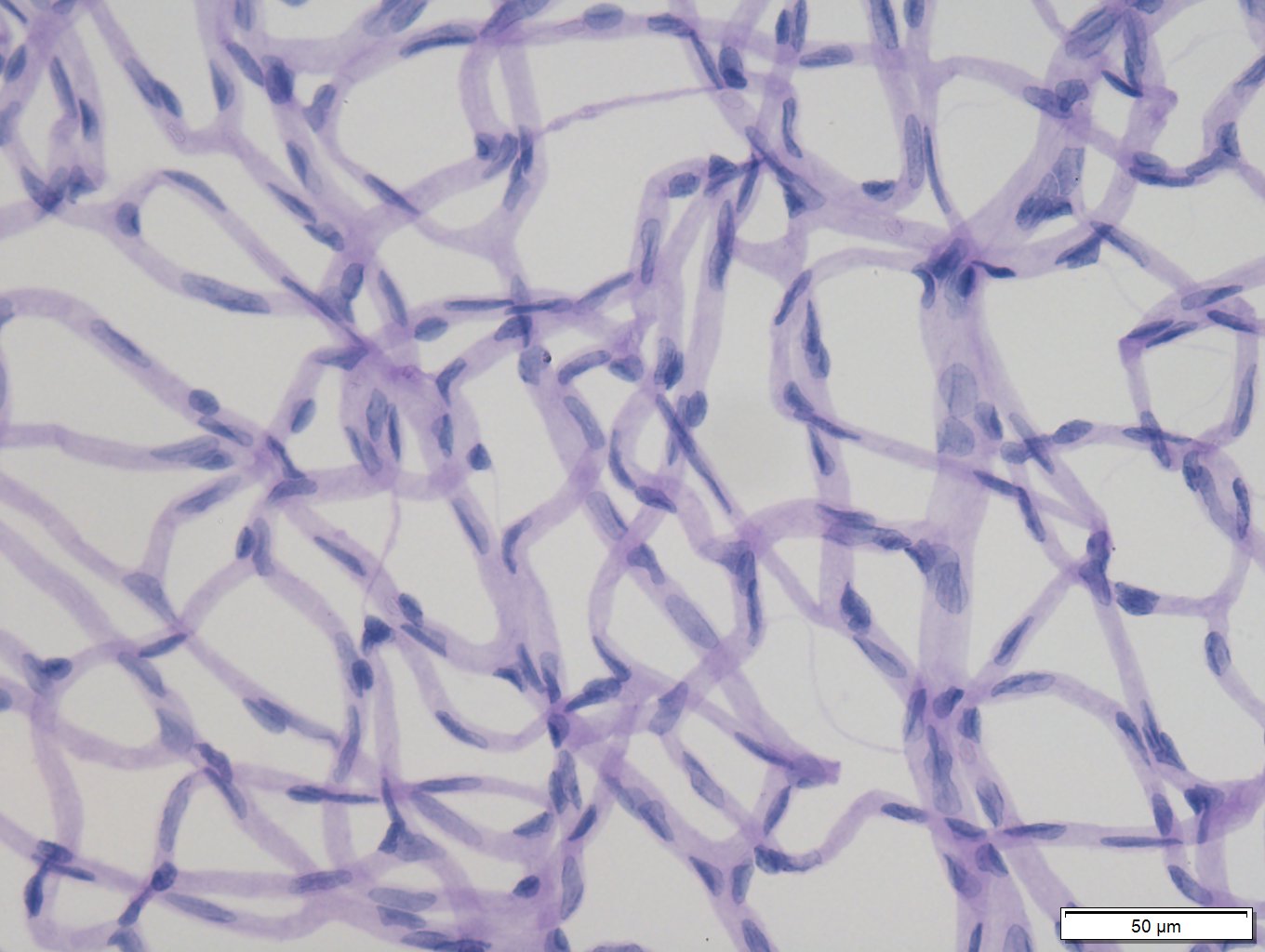

Supplement: S2 File — (ZIP) [file pone.0312791.s002.zip › Fig 6/Fig6 PAS/DM1.jpg]

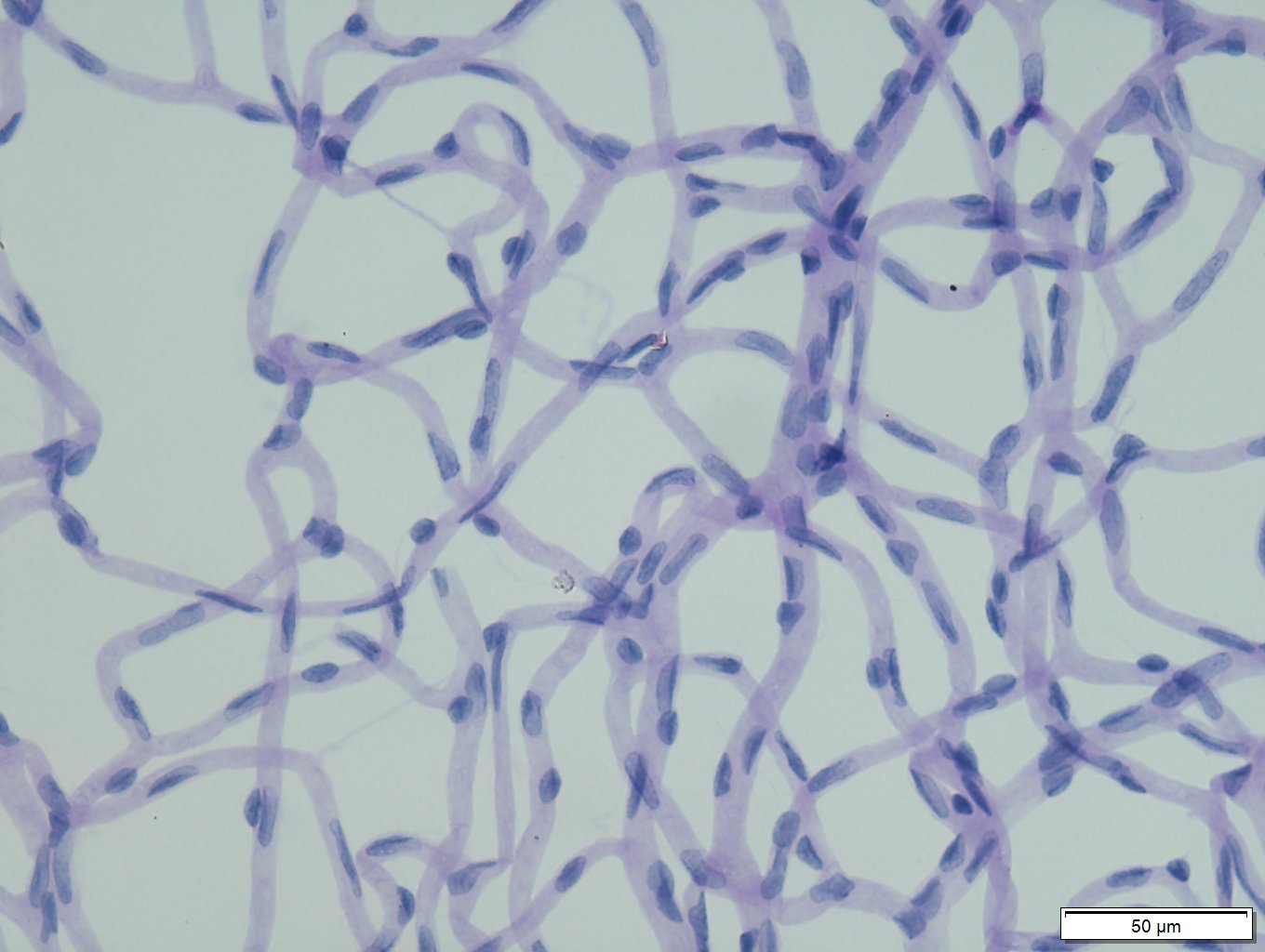

Supplement: S2 File — (ZIP) [file pone.0312791.s002.zip › Fig 6/Fig6 PAS/DM2.jpg]

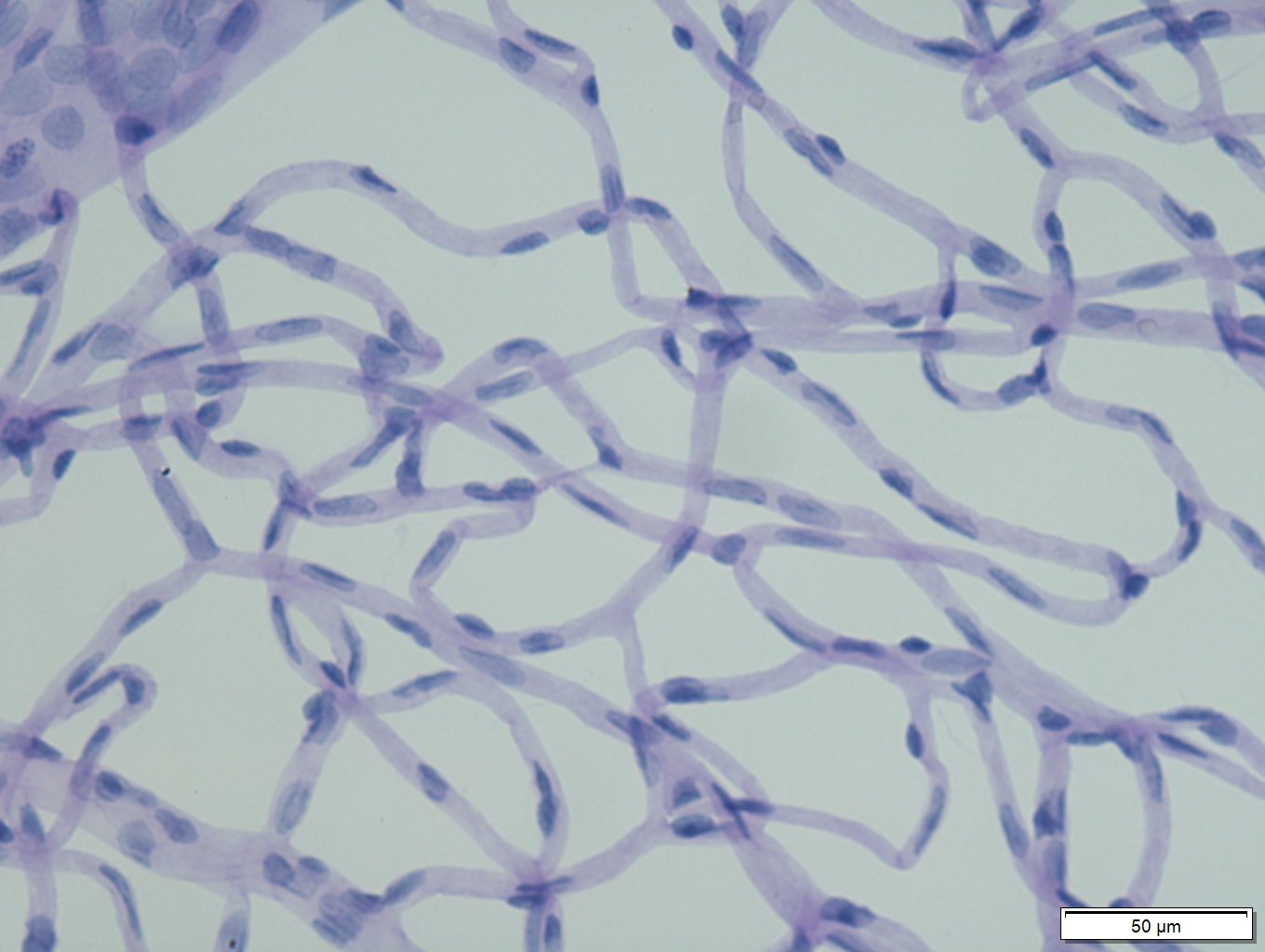

Supplement: S2 File — (ZIP) [file pone.0312791.s002.zip › Fig 6/Fig6 PAS/DM3.jpg]

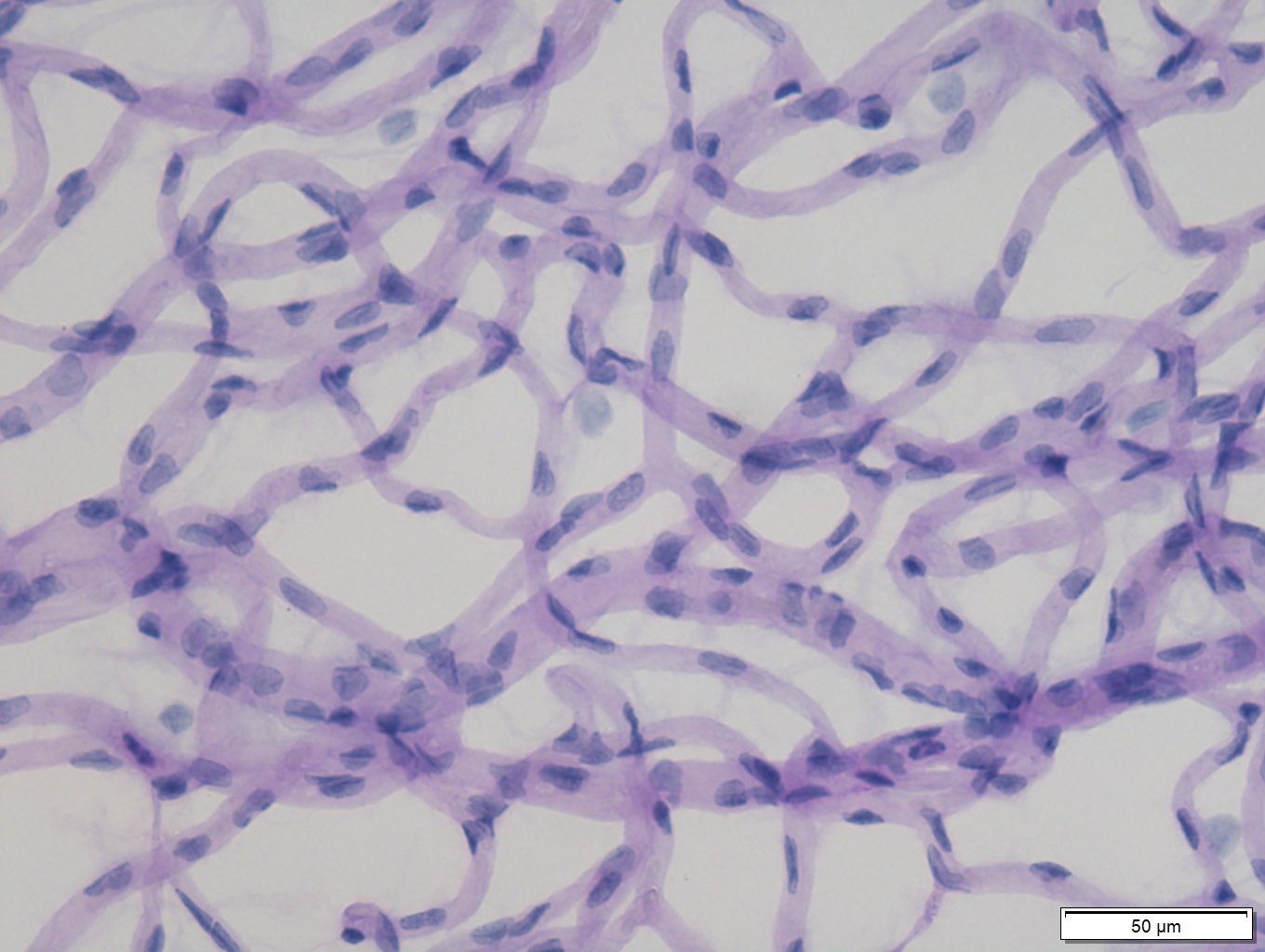

Supplement: S2 File — (ZIP) [file pone.0312791.s002.zip › Fig 6/Fig6 PAS/NC1.jpg]

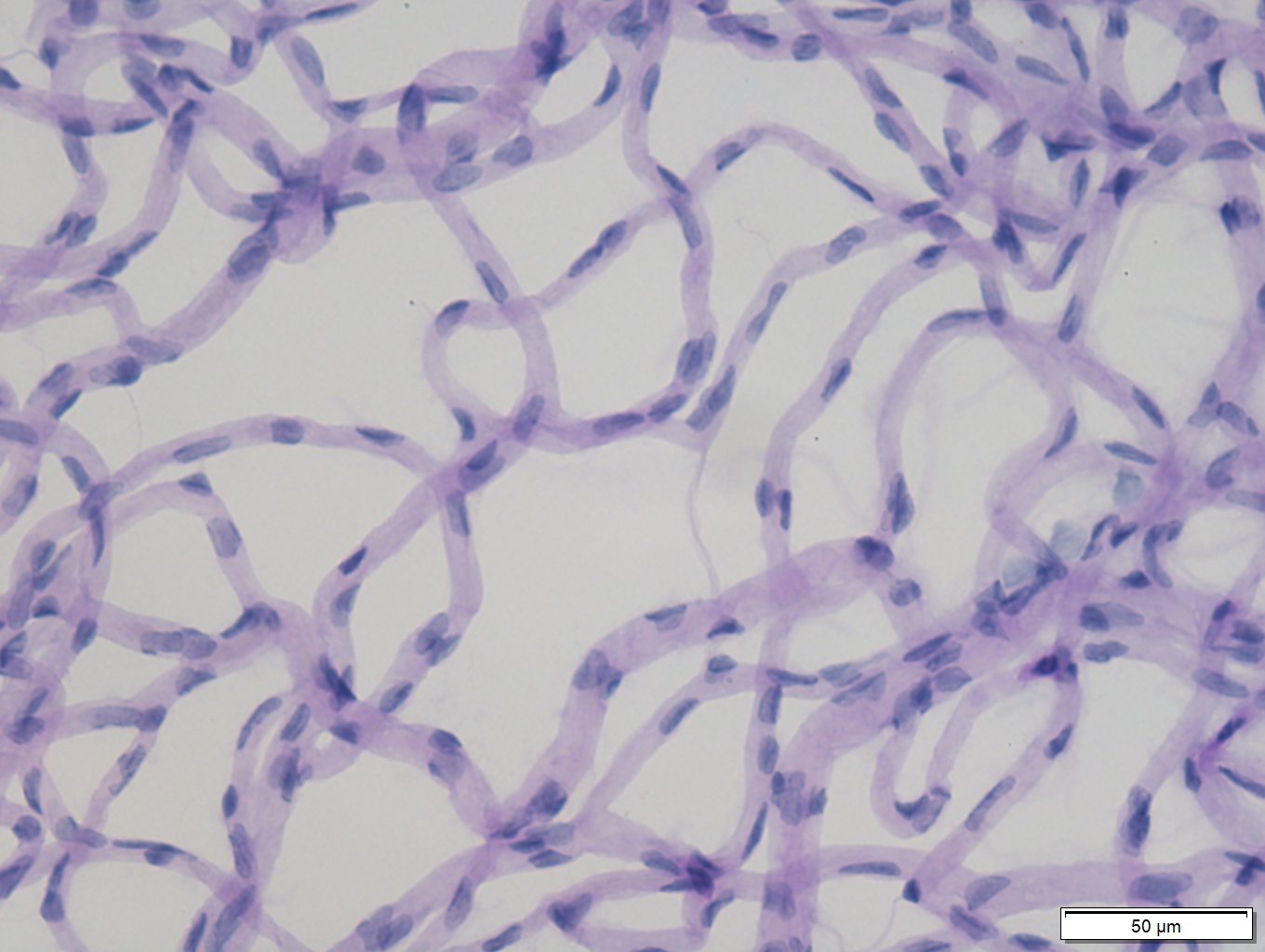

Supplement: S2 File — (ZIP) [file pone.0312791.s002.zip › Fig 6/Fig6 PAS/NC2.jpg]

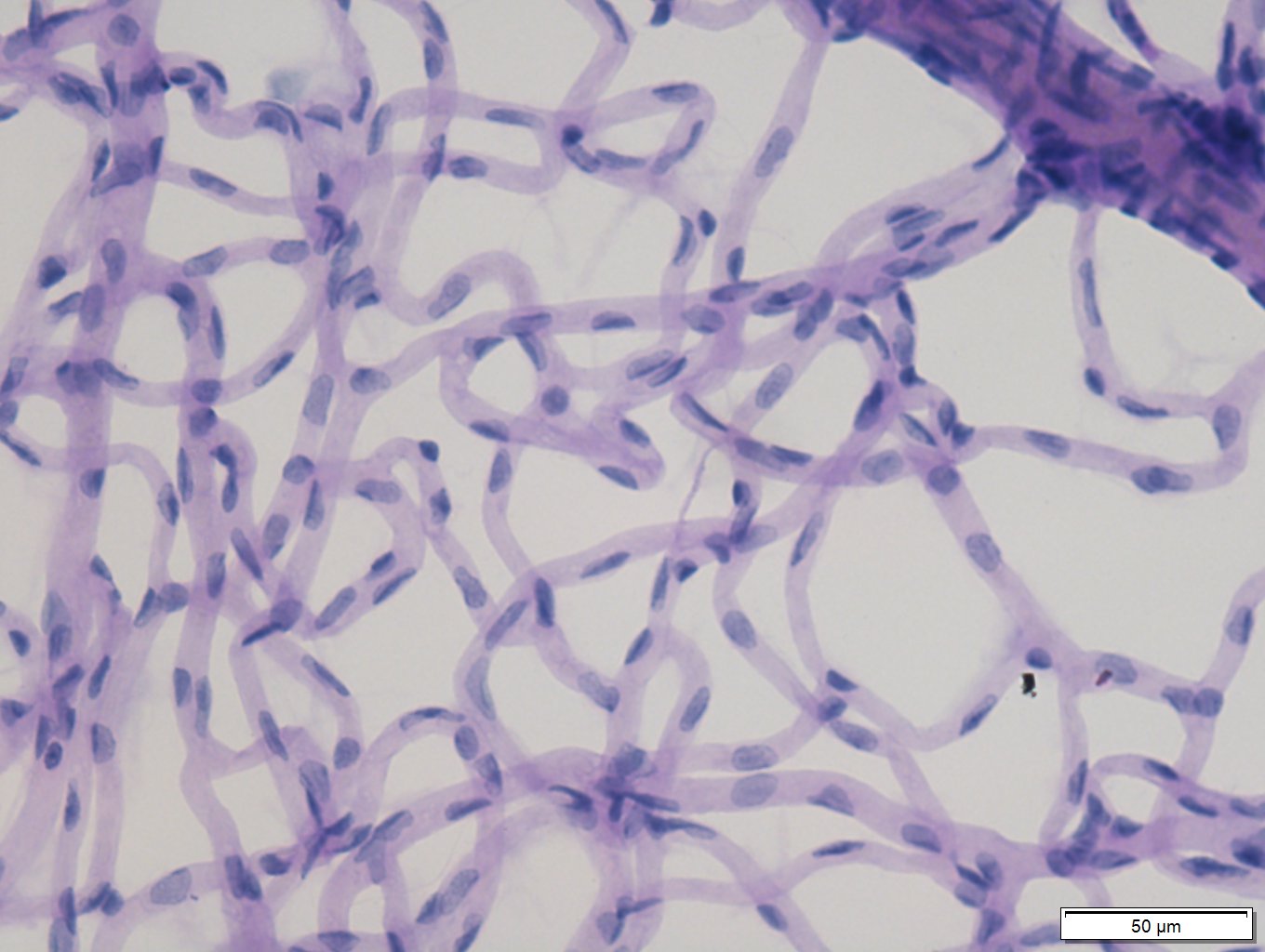

Supplement: S2 File — (ZIP) [file pone.0312791.s002.zip › Fig 6/Fig6 PAS/NC3.jpg]

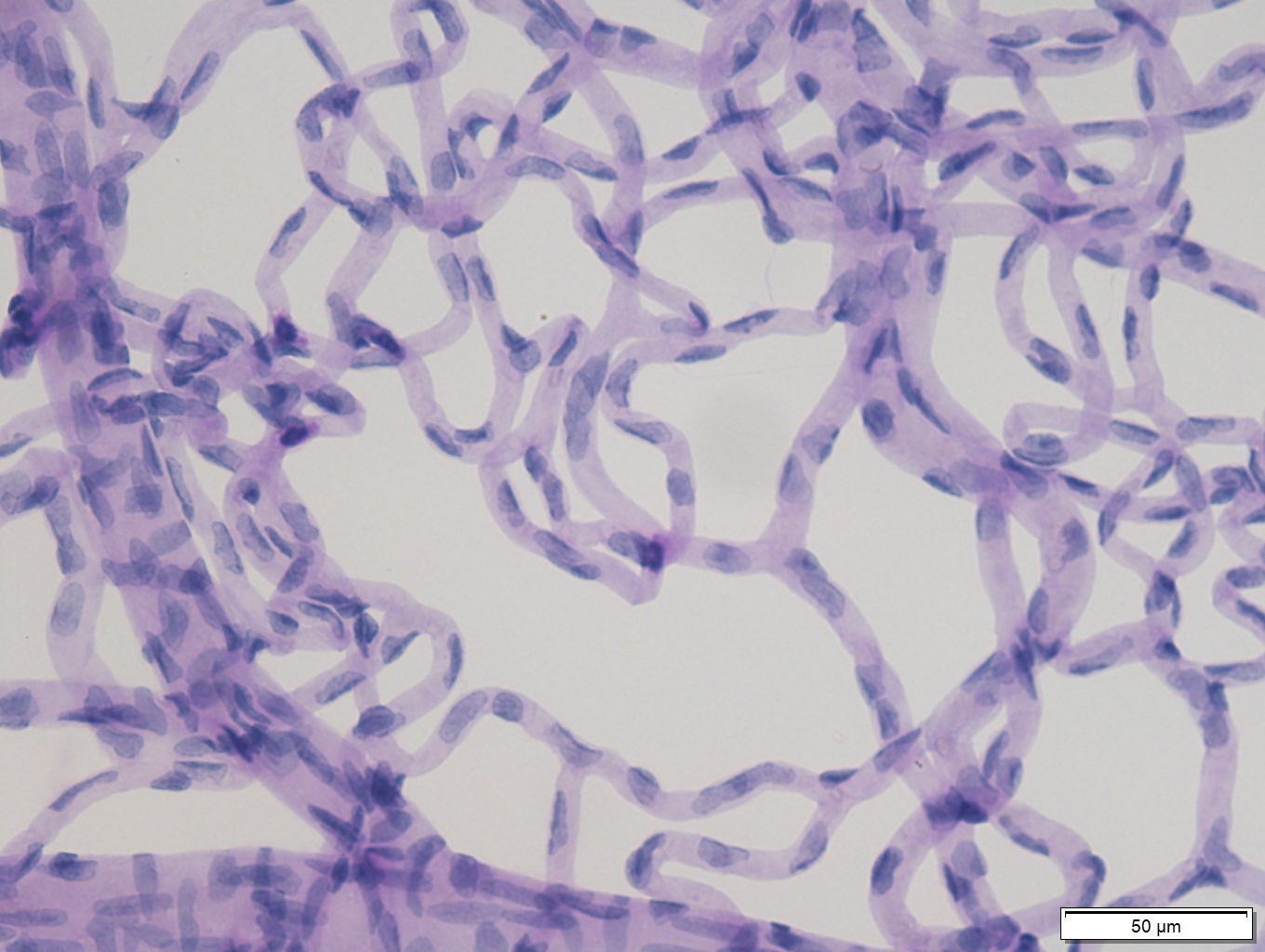

Supplement: S2 File — (ZIP) [file pone.0312791.s002.zip › Fig 6/Fig6 PAS/OE1.jpg]

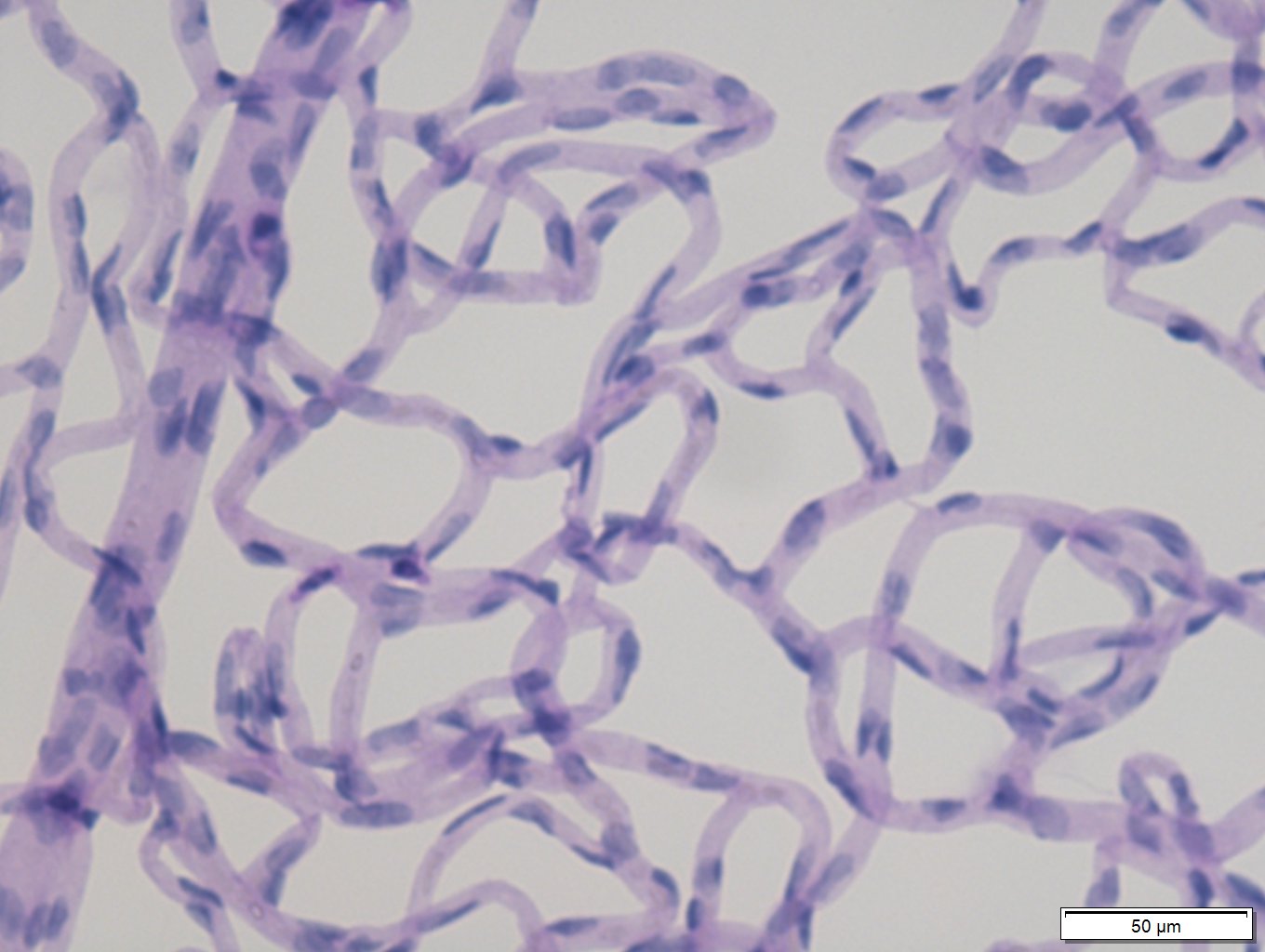

Supplement: S2 File — (ZIP) [file pone.0312791.s002.zip › Fig 6/Fig6 PAS/OE2.jpg]

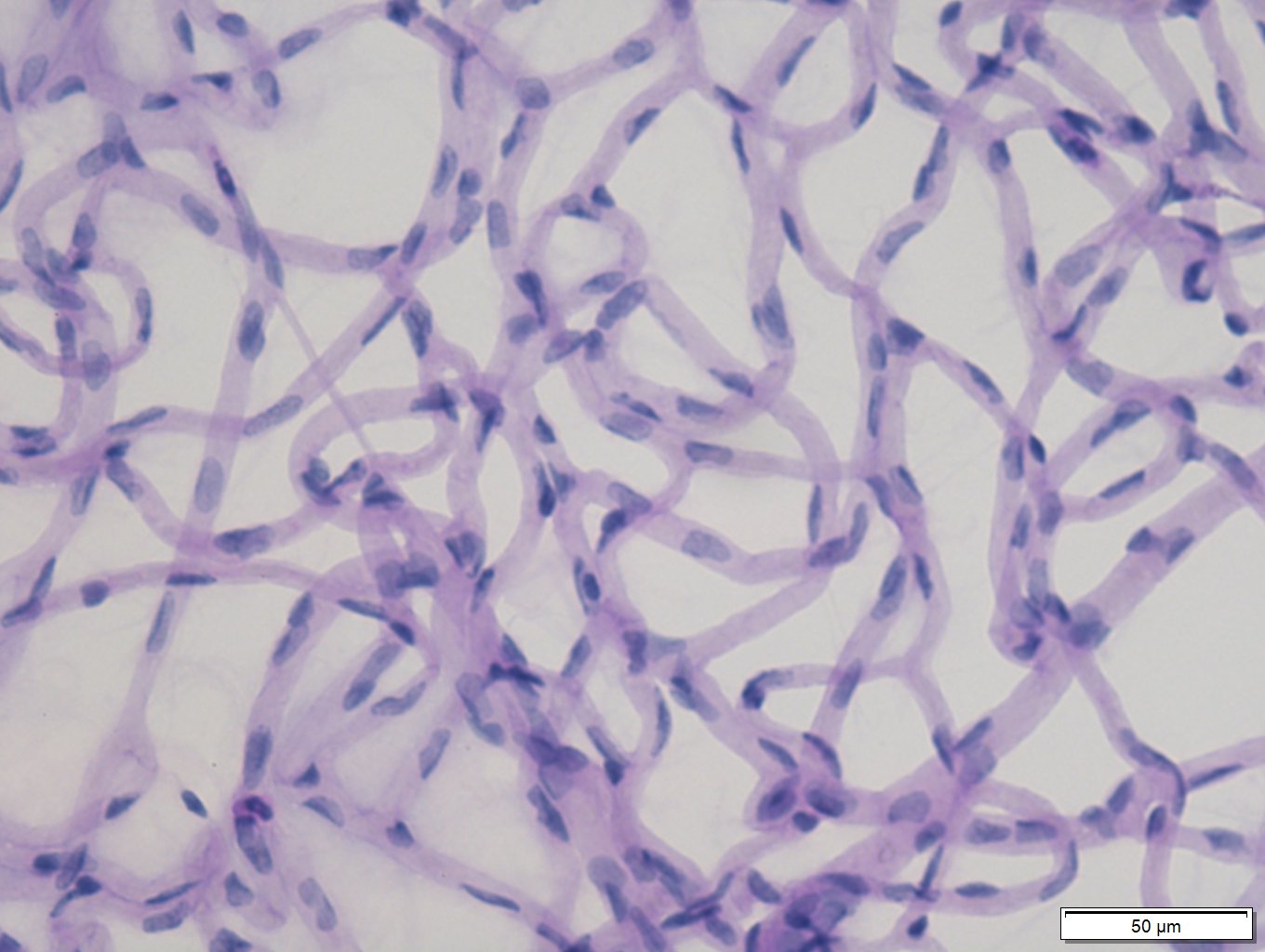

Supplement: S2 File — (ZIP) [file pone.0312791.s002.zip › Fig 6/Fig6 PAS/OE3.jpg]

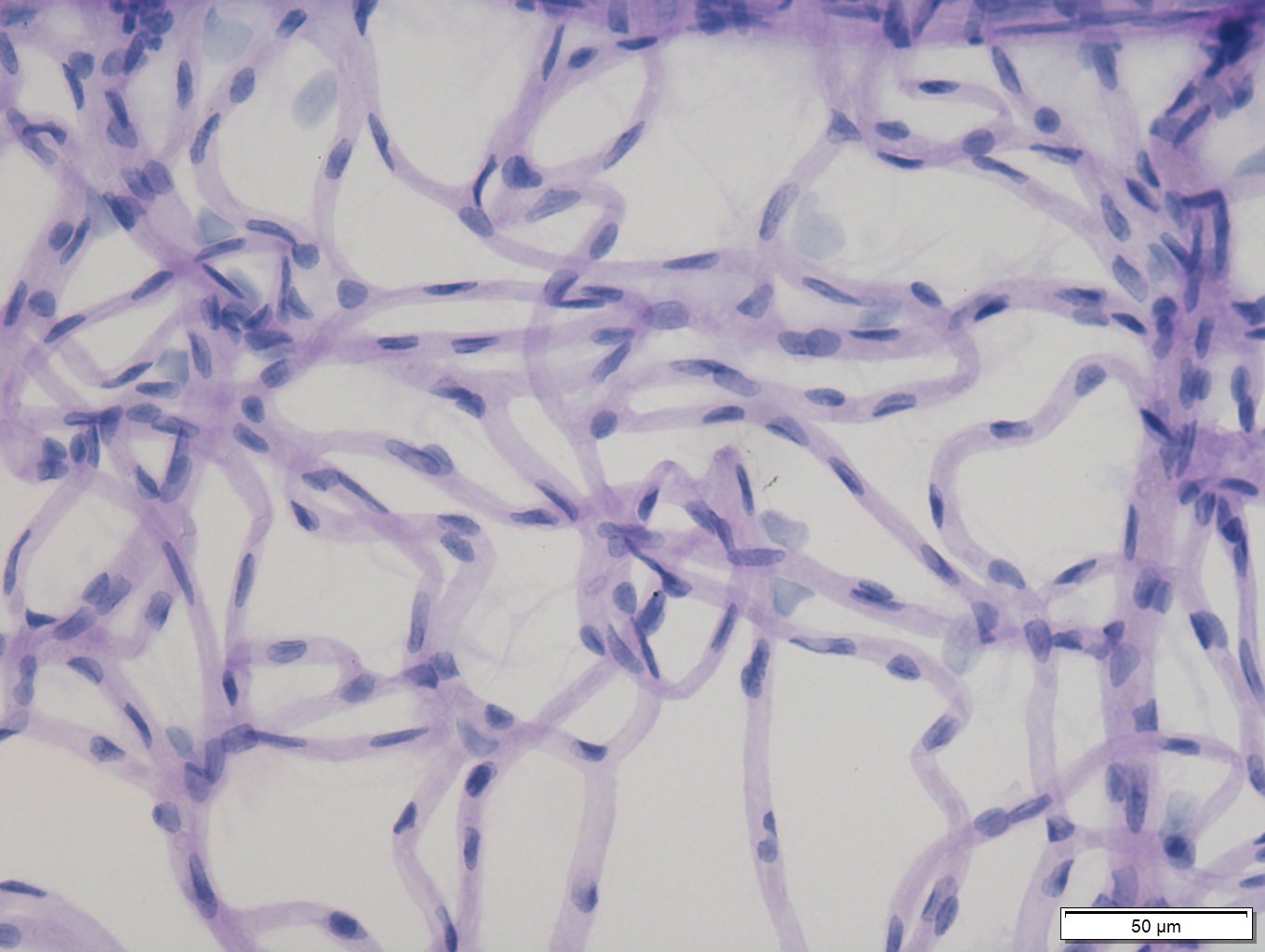

Supplement: S2 File — (ZIP) [file pone.0312791.s002.zip › Fig 6/Fig6 PAS/WT1.jpg]

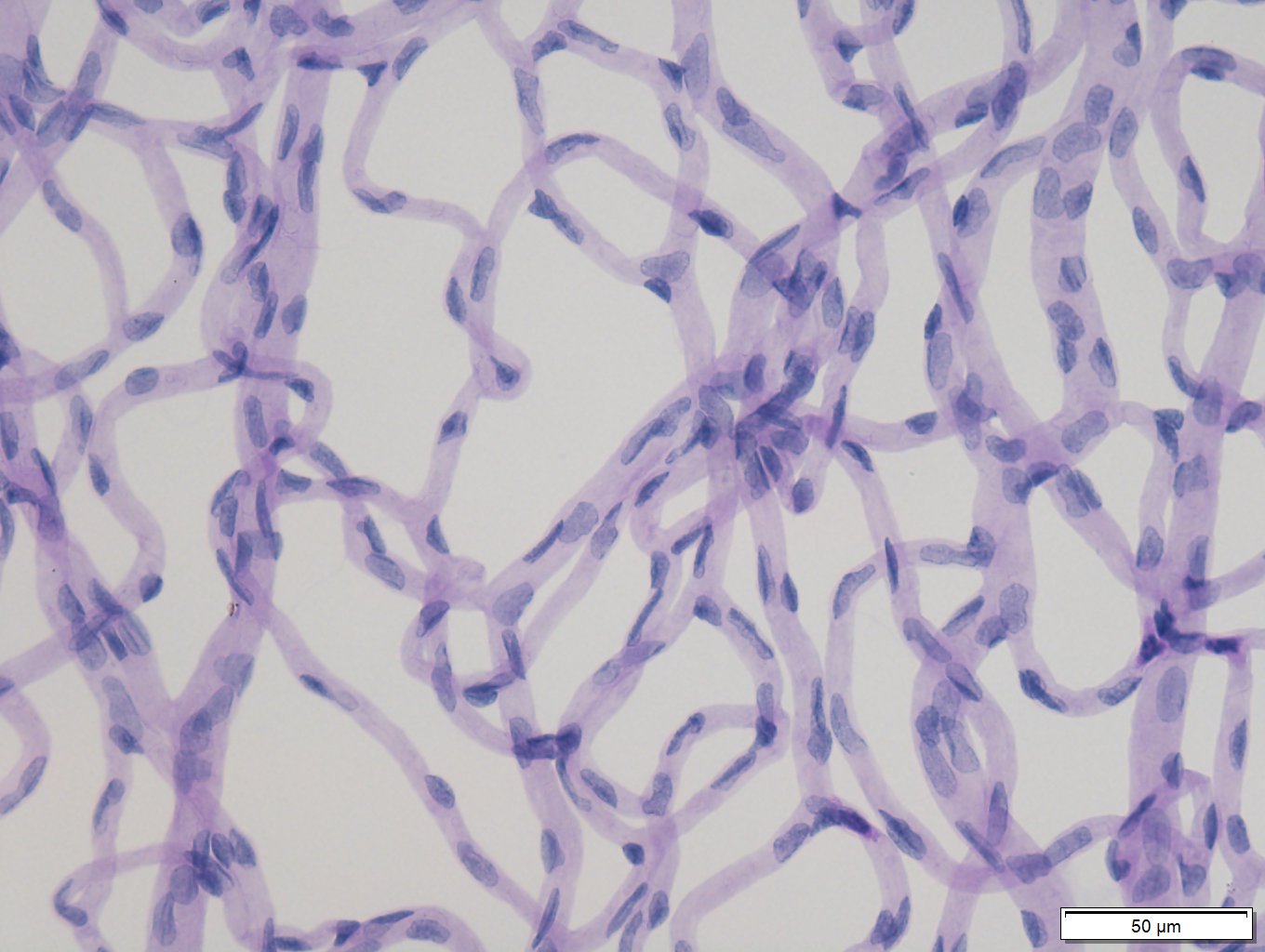

Supplement: S2 File — (ZIP) [file pone.0312791.s002.zip › Fig 6/Fig6 PAS/WT2.jpg]

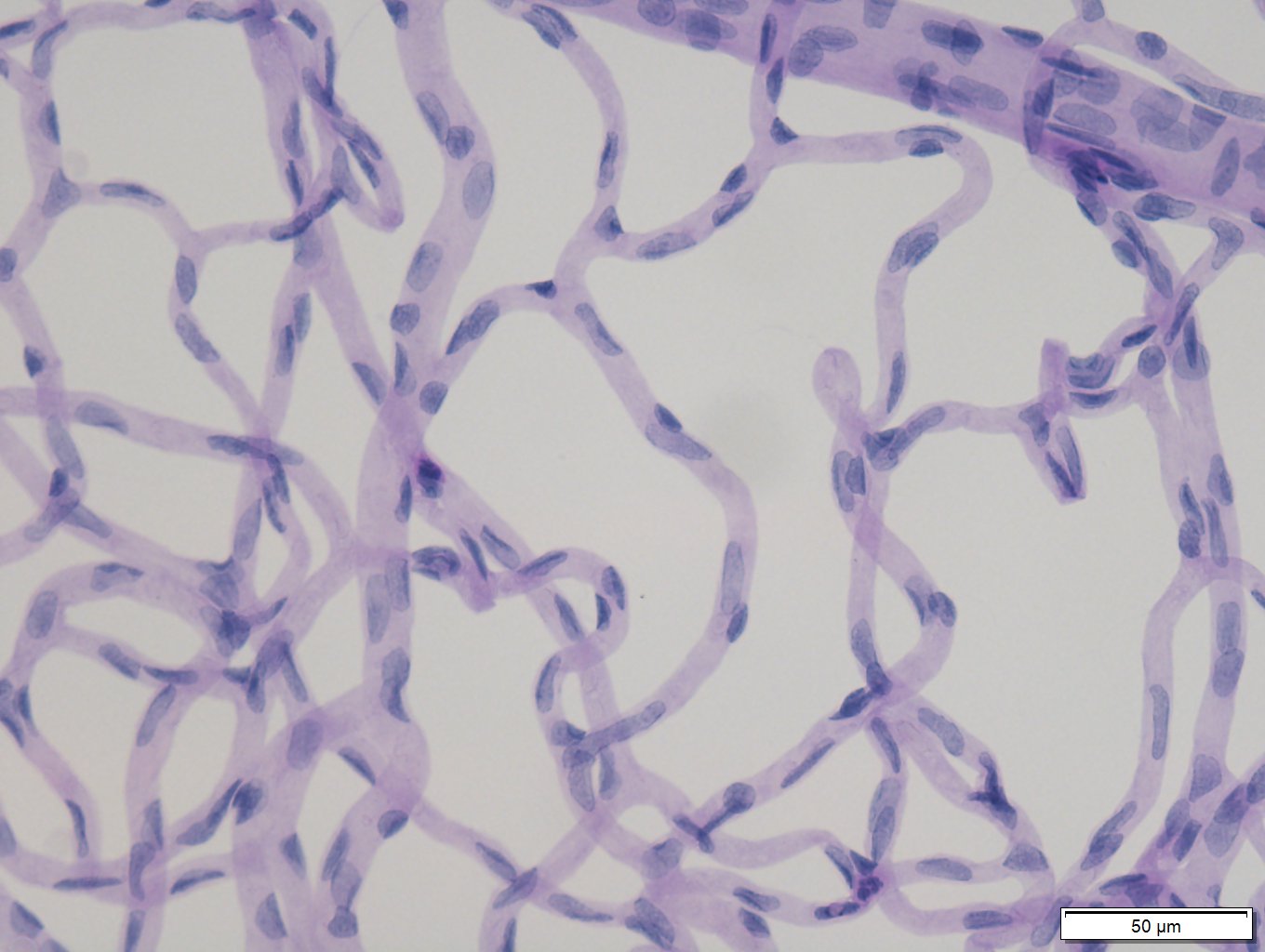

Supplement: S2 File — (ZIP) [file pone.0312791.s002.zip › Fig 6/Fig6 PAS/WT3.jpg]
